# Liverwort altitudinal patterns in East Kamchatka: dividing the indivisible

**DOI:** 10.3389/fpls.2026.1904286

**Published:** 2026-09-11

**Authors:** Vadim A. Bakalin, Daniil A. Bakalin, Seung Se Choi

**Affiliations:** 1 Laboratory of Cryptogamic Biota, Botanical Garden-Institute, Vladivostok, Russia; 2 Team of Ecosystem Survey, National Institute of Ecology, Seocheon, Republic of Korea

**Keywords:** altitudinal zonation, distribution patterns, East Kamchatka, Gaussian distribution model, hepaticae, isolation diagnostics, maximum likelihood estimation, occurrence records

## Abstract

**Background:**

Small cryptogamic plants, including liverworts, are confined in their occurrence to microhabitats and may be weakly associated with communities formed by dominant vascular plant species. This complicates the understanding of patterns in their elevational distribution in mountainous areas — a task that becomes doubly challenging under conditions of poorly expressed altitudinal zonation.

**Methods:**

Based on the concept of a Gaussian distribution model, modified in the present study to account for constraints imposed by the characteristics of the material, potential distribution curves and occurrence frequencies were calculated for each species using collections made in the study area in East Kamchatka.

**Results:**

Four elevational groups of liverworts were identified based on the occurrence-record adaptation of the range-density maximum likelihood estimation framework. The transition from one elevational group to the next is characterized by a pronounced trough in the positions of the elevational core centers of species and clearly defines each new group identified using the method applied herein. Species lists obtained using the abovementioned method were compared with taxonomic spectra for five elevational belts empirically recognized based on vascular plant characteristics. The groups identified under each approach partially coincide. However, the pyroclastic-field flora cannot be recognized as a distinct elevational group.

**Conclusion:**

The method allows the identification of elevational groups under conditions where gradual changes and deep interpenetration of adjacent communities along the elevational profile preclude the recognition of distinct vegetation belts. Even where elevational vegetation complexes can be recognized, the method applied here appears preferable to delimiting elevational groups based on dominant communities, since the distribution of liverworts is tied more closely to substrates than to communities formed by dominant vascular plant species. The applied method may be useful for incomplete data on altitudinal distribution, as the expected distribution is modeled using range–density maximum likelihood estimation.

## 1 Introduction

Mountain systems are unique natural laboratories where striking changes in plant formations occur over a small area. These formations deeply interpenetrate each other and coevolute both as plant communities and as elementary phylogenetic units for the taxa that comprise them (Silveira et al., 2019; Molina-Venegas et al., 2020; Dellinger et al., 2024). In this context, bryophytes, due to their poikilohydric nature, can be considered promising model organisms for studying altitudinal changes in taxonomic composition. For example, if a change in phanerophyte plants indicates a significant climatic shift, then, in turn, a change in the composition of epiphytic bryophytes on these same trees may signal earlier and subtler changes, such as fog frequency or air humidity (Maul et al., 2023; Mejia et al., 2020; Frahm and Gradstein, 1991).

The idiosyncratic nature of liverwort distribution patterns has long been recognized. What appears to be the first review of the geographic distribution patterns of liverworts was provided by Hübener (1834: p. XLII, translated from German by the authors), who described a remarkable pattern of their distribution: “We find that they [liverworts] are far more closely related to their habitats, their soil, and climatic altitude conditions than to geographical latitudes”. The following speculations further elucidate Hübener’s idea of ​​liverworts’ connection with specific substrates and reveal the altitudinal zonation of their distribution in the mountains of Europe. The author (Hübener, 1834: p. XLIV) provides sound examples in which high-mountain species are found in swamps at lower altitudes if the ecological conditions of the habitats are favorable for them. He further wrote (1834, p. XLV, translated by the authors): “Turning our attention from the general distribution to the specific occurrence of these species, we find them all strictly bound to landscape, habitat, and soil. Every type of soil, every similar air mass, supports its own species, indeed entire groups”. This idea was widely accepted. Later, in the classic monograph “Geographie der Moose” by Herzog (1926) (section “Substrat”, pp. 67-74), the author vividly describes the dependence of bryophyte species composition on substrate characteristics, contrasting acidic and alkaline substrates and devoting considerable space to the description of epiphyllous communities. This substrate confinement, coupled with the small size of plants, inspired the concept of microniches or the “ability to survive in microhabitats” (Schuster, 1983: 478). The above leads to a ‘blurring’ of the distribution patterns of bryophytes, compared with vascular plants. In general, however, their patterns are fundamentally similar to those of vascular plants (Herzog, 1926).

A natural problem arises: how to describe the altitudinal patterns of bryophyte distribution if bryophytes are tied more to the substrate and microniches than to community types (Mejia et al., 2020). For example, species descend along stream valleys in the mountains, much lower than the belts with which they might be regarded as naturally associated (Grishin, 2011; Neshataeva, 2009; Tsuyuzaki, 1991; Geissler and Velluti, 1995). Considerable effort has been devoted to describing this phenomenon. Bryophyte diversity has been described as following a unimodal pattern, where the highest diversity is achieved in the middle part of the altitudinal zone due to the mixing of species from adjacent belts and specific climatic conditions (Maul et al., 2023). Other patterns have also been described: 1) increasing diversity with altitude is due to an increasingly favorable climate in the upper belts and the availability of suitable substrates (Mejia et al., 2020). 2) Decreasing diversity toward higher elevations, where extreme cold temperatures, strong winds, long-lasting snow cover, and physiological dryness become the main limiting factors (Sun et al., 2013). 3) Bimodal distribution with two peaks (Song et al., 2024). 4) Idiosyncratic (region-specific) patterns that do not fit into general schemes (Sun et al., 2013). This emphasizes that the final shape of the diversity-altitude curve depends on many regional factors: geographic location (tropics or temperate latitudes, or other latitudinal zones), the degree of isolation of the mountain system (continental massifs or oceanic islands), altitudinal range, and the specific taxonomic group (liverworts are often more dependent on moisture than mosses) (Maul et al., 2023). Examples of these patterns can be found in studies conducted in various mountain systems, including the Andes (Mejia et al., 2023), the mountains of China (Sun et al., 2013), the Himalayas (Bahuguna et al., 2015), the Alps (Hanusch, 2025), and oceanic islands such as Réunion (Ah-Peng et al., 2007) and the Azores (Coelho et al., 2021; Gabriel et al., 2025).

The above and other studies provide an overview of the drivers (including through coupled interactions) that determine bryophyte distribution patterns along an altitudinal gradient, including mesoclimate (Maul et al., 2023), substrate (Mejia et al., 2020; Henriques et al., 2017; Coelho et al., 2021), forest structure (Mejia et al., 2020; Stehn et al., 2010), and interactions with vascular plants (Chen et al., 2017; Hanusch, 2025). In addition to the factors determining diversity changes with altitude, the question of whether bryophytes in the broad sense or liverworts in particular exhibit altitudinal zones and how they relate to altitudinal zones defined for vascular plants inevitably had to be addressed. The seminal paper in this regard is the study by Frahm and Gradstein (1991). Based on a study of the bryoflora of tropical rainforests, they proposed a scheme using bryophytes as indicators to distinguish five altitudinal zones: lowland forest, submontane forest, lower montane forest, upper montane forest, and subalpine forest. It should be noted that, as is evident from the names of the elevational belts of the bryophyte flora, they were biased toward the elevational structure of vegetation belts defined by phanerophytes, each element of which was subsequently assigned a bryofloristic characterization. The selected belts were characterized based on changes in projective cover, bryophyte phytomass, and the appearance or disappearance of characteristic indicator species and life forms. Due to regional specificities, this scheme is not universal. An important theoretical debate concerns the question of what better reflects reality: the identification of discrete ‘zones’ or an analysis of community change as a continuous function along a gradient. Although the concept of altitudinal vegetation belts is convenient for classification and mapping, modern ordination methods (e.g., canonical correspondence analysis, CCA) often show that species succession occurs gradually, without sharp boundaries (Mejia et al., 2020). The latter better reflects the gradual nature of changes in most environmental factors with altitude. It is likely that both approaches— ‘discrete’ and ‘continuous’ — are not mutually exclusive, but rather complementary tools for analyzing altitudinal zonation. There are relatively few studies examining altitudinal patterns in this sense. They consider changes in the species composition of bryophytes either across arbitrarily chosen altitudinal intervals (e.g. every 200 m in elevation) or link bryophyte diversity to *a priori* belts identified based on dominant vascular vegetation (Mejia et al., 2020; Grau et al., 2007; Ah-Peng et al., 2007, 2012).

The above studies seem to have two weaknesses: 1) they operate with de facto observed species distributions, although incomplete knowledge of the bryophyte flora is a ‘commonplace’ mentioned even in manuals (Schofield, 1985); 2) these studies record the fact of the presence or absence of a species, thereby obscuring information on the center of its abundance along the altitudinal gradient.

Meanwhile, starting with classical works, including Shelford’s law of tolerance and the concept of limiting factors (Odum, 1971), the distribution of species abundance is represented by a bell-shaped curve corresponding to a Gaussian (normal) distribution. More recent studies widely confirm this assumption (Begon et al., 2006). Whittaker (1967) clearly showed that the distribution of species along natural gradients (e.g., humidity or altitude) most often takes the form of symmetrical or slightly shifted bell-shaped curves. Gauch and Whittaker (1972) described the use of a Gaussian function to simulate the distribution of communities. Ter Braak (1986) directly uses the Gaussian response model to create algorithms that calculate a species ecological optimum from available data. Austin (2002) examines whether response curves should always be Gaussian and explains the theoretical underpinnings of using the normal distribution in modeling.

Therefore, when searching for patterns in the altitudinal distribution of bryophyte species (and liverworts in particular), it is theoretically possible to use concepts of a normal (Gaussian) distribution to calculate the estimated distribution of each species based on a limited amount of available data. Furthermore, obtaining such data for each species allows us to address the issue of whether they form belt-like structures and the extent to which these structures correspond to the defined vegetation zones. This approach is intended to reduce the influence of stochastic occurrences of species outside their typical altitudinal range. This issue was the objective of our previous study, devoted to the study of liverwort flora in the Southern Sikhote-Alin (Klimova et al., 2024). In describing the results obtained, we emphasized that both the applicability of the method itself and the need for its further modification would only be clarified by subsequent research. The present work modifies the method itself and attempts to apply it to the analysis of liverwort flora where the concept of altitudinal zones based on vascular vegetation is largely obscured by both the wide interpenetration of communities and the constant disturbance of existing communities as a result of volcanic activity.

Volcanism deserves special mention here: active volcanic processes lead to the constant creation of new habitats unoccupied by plants, the constant destruction of existing landscapes, and changes in the structure and characteristics of the substrate. All these events are a common reality in East Kamchatka (Neshataeva, 2009). The resulting picture of vegetation altitudinal distribution looks quite chaotic. Moreover, a number of studies demonstrate the influence of volcanism on speciation processes, or more precisely, the stimulation of this process (Craddock, 2016; Nogales et al., 2022). Thus, active volcanic processes, snowfall redistribution, temperature inversions, substrate drainage characteristics, and other factors are difficult to take into account together. This means that the liverwort assemblage of Nalychevo Park, at first glance, appears to be a simple continuum, making it very difficult to find clues to describe its altitudinal distribution using traditional methods of ranking species by community association. The same should be said about attempts to describe taxonomic shifts by fixed altitudinal intervals, due to the interpenetration of communities, because one fixed interval may include different vegetation types and does not reveal the potential altitudinal core of the species distribution. Accordingly, our analysis proceeded from the altitudinal distribution patterns of individual species, which were then combined into elevational groups. Describing the altitudinal patterns of liverwort distribution in East Kamchatka, an area within the Pacific Ring of Fire, was therefore the primary goal of this study.

Furthermore, we considered it necessary to examine the validity of the existing concept of landscape complexes (roughly equivalent to altitudinal zones, although it is difficult to call them ‘altitudinal’ due to the extensive interpenetration and transformations experienced under the influence of active volcanism) in terms of gradations in liverwort composition along the altitudinal profile. In other words, are the five altitudinal zones, the identification of which is described in the Materials and Methods section, valid for assessing altitudinal trends in flora changes, and to what extent will the changes associated with these zones correspond to the patterns identified based on the assumed Gaussian distribution of each species?

## 2 Materials and methods

### 2.1 Study area

Although liverwort distribution across Russian Asia as a whole, including its amphi-Pacific region, was studied in a rather fragmentary manner (Bakalin, 2013), this is less true for the Kamchatka Peninsula. A significant amount of data is available for the peninsula, and, according to available information, it is generally one of Russia’s taxonomically richest regions for liverworts (Bakalin, 2026). The vegetation cover of Kamchatka has been described repeatedly. Among the most important sources, one should note the literary description of vegetation provided by Komarov (1912) and the description according to strict geobotanical canons by Neshataeva (2009). With regard to liverworts, the features of the peninsula were described by Bakalin (2009) and recently briefly summarized by Bakalin (2026). The present account is based on the study of liverworts in Nalychevo Nature Park, whose natural conditions and their characteristics were described by Bakalin and Klimova (2018). We will mention here only the most important characteristics: 1) the nature of the vegetation as a whole is hemiarctic, with an unclear division into belts, due to frequent temperature inversion phenomena and abrupt interruptions due to the transition at the upper limit to alpine heaths and glaciers, 2) there are a number of active volcanoes in the park, which radically change the vegetation in small areas (in historical retrospect — totally) due to eruptive processes: the fall of scoria and the outpouring of lava, as well as thermal manifestations (travertine shields, thermal springs), 3) the virtually insular position of the peninsula as a whole, which hinders the migration of species from adjacent regions, especially conditionally “southern” species.

These drivers explain the altitudinal ‘indivisibility’ of the flora of the studied area, as 1) different communities interpenetrate extensively, 2) pyroclastic fields, which outwardly resemble high-mountain deserts, are formally a temporary phenomenon, but at the same time relatively persistent, due to the constancy of eruptions, 3) due to the large amount of precipitation, especially in solid form, such elements as migratory snowfields are found right up to the conventional belt of stone birch forests, thereby forming around them impoverished snowbed communities, formally located in the belt of birch forests, even at elevations of 400 or 500 m above sea level.

A more detailed description of the nature of the park is provided in **Supplementary Online Appendix 1**.

**Figure 1:**
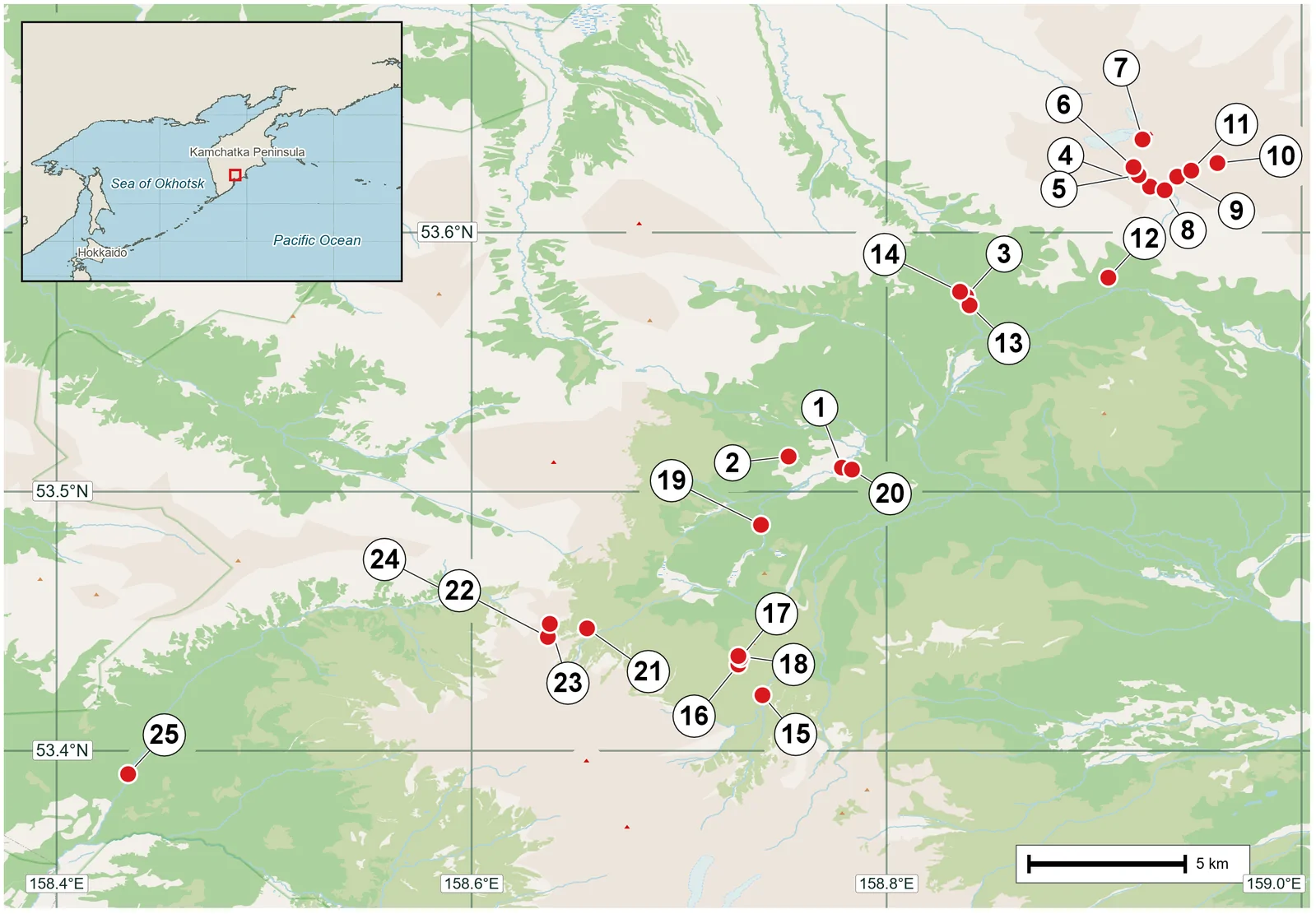
The collecting localities according to the **Supplementary Online Table 1**.

The liverwort collection sites for the sampling conducted in 2022 are situated within an altitudinal range from 239 to 2075 m a.s.l., thus covering the entire spectrum of plant communities represented in the park: from floodplain forests to high-mountain barren areas of the Dzendzur volcano crater crown. The descriptions of the collection sites and dates are provided in **Supplementary Table 1** in accordance with the numbering adopted on the map (**Figure 1**). All the specimens collected in 2022 were delivered alive to the Cryptogamic Biota Laboratory of the Botanical Garden-Institute in Vladivostok (international herbarium acronym VBGI), where they were examined with dissecting and compound microscopes while macro- and micromorphological features were photographed, including oil bodies in the leaf cells. The results of the identification were entered into a database, from which information was then output in the following fields: field numbers, geographic coordinates, altitude above sea level, habitat data, and associated species.

### 2.2 Material treatment

The material for this work was provided by specimens collected in 2001, 2015, and 2022. The specimens collected in 2001 and 2015 were previously summarized and described in the form of a checklist (Bakalin and Klimova, 2018). Here, we simply use the information available there in a completely different way. The collection made in 2022 was never published or evaluated. During the collection of both the previously described collection and the data summarized here for the first time, the following were noted: 1) coordinates and altitude above sea level (using a GPS receiver for the 2015 and 2022 collections and high-resolution topographic maps for the collection gathered in 2001), 2) community type and its general characteristics, 3) substrate, its illumination, and moisture content – ​​based on visual characteristics, without the use of special equipment. After collection, the specimens were examined using anatomical and morphological methods in the laboratory, and the species were identified. Since northern liverworts often grow intermixed within one cushion, the number of original identifications exceeds the number of collected specimens. The total number of identifications is 808, of which 496 are from the 2022 collection. The materials collected in 2001 are deposited in KPABG, with a few duplicates in VBGI. The 2015 and 2022 collections are entirely held in VBGI. The nomenclature in the paper follows the world checklist of liverworts (Söderström et al., 2016), with the exception of the genera 1) *Blepharostoma* (Dumort.) Dumort., *Frullania* Raddi, *Schistochilopsis* (Kitag.) Konstant. (species later described in Bakalin et al., 2020a, 2020b, Mamontov et al., 2020 are accepted), 2) *Marchantia* L. (due to tradition in far-eastern literature, we adopted a narrow species concept, the appropriateness of which, however, can be fairly questioned), 3) *Pseudolophozia* Konstant. et Vilnet, whose generic status is accepted, 4) the genera of *Solenostomataceae* are treated in a narrow sense (thus including *Plectocolea* (Mitt.) Mitt. and *Metasolenostoma* Bakalin et Vilnet following Bakalin et al., 2014).

### 2.3 Preparation of statistical analysis

Information on the newly obtained data (2022 collection) was summarized in a schematic table (**Supplementary Table 2**). This table was supplemented with information from previously published articles (Bakalin and Klimova, 2018) necessary for statistical analysis. The table structured the information as follows:

1. species name,
2. list of elevations without duplicates, for the 2022 collection only (regardless of how many times the species was collected at a given elevation),
3. list of collection points according to the additional coordinate table, in order from 1 to 25, distributed strictly from north to south, for the 2022 collection only,
4. description of habitats and community types, for the 2022 collection only,
5. species presence by presumed altitudinal vegetation complexes, with the designations BF – Betula forests, this belt also includes *Alnus hirsuta* (Spach) Rupr. tall stands in the valley, CF – Crooked forests (*Pinus pumila* (Pall.) Regel or *Alnus fruticosa* Rupr. dominating communities), TB – tundra belt, PF – pyroclastic fields (actually a temporary phenomenon, but acquiring regular features due to the regularity of eruptive processes in the region), AH – alpine heathlands (characterized by open vegetation cover, without dwarf shrubs), the presence of the species is marked by the number 1,
6. repeated altitudes for the 2022 collection; each occurrence record corresponds to one taxon identified in one collected specimen, so that repeated elevations of the same taxon always correspond to different specimens. Because northern liverworts frequently grow intermixed within one cushion, a single mixed specimen may yield several records, but these belong to different taxa. The repetitions therefore measure specimen-based detection frequency, which is used below to estimate the relative frequency of occurrence; they are not a count of individuals and not a measure of cover. That is, if a taxon was identified in three separate specimens collected at a given elevation, this elevation is repeated three times.
7. repeated altitudes for previously published data (2001 and 2015 collections); the same specimen-based definition of an occurrence record applies to all three collections,
8. field numbers, only for the 2022 collection,
9. associated species, only for the 2022 collection,

Thus, this table contains information on two parameters discussed in this paper: 1) the distribution of species across landscape complexes; 2) a list of altitudes at which the taxa were identified, each repeated elevation of a taxon representing a separate specimen-based occurrence record and thus a measure of relative frequency of occurrence rather than of organismal abundance.

### 2.4 Statistical analysis of altitudinal distribution

**Figure 2:**
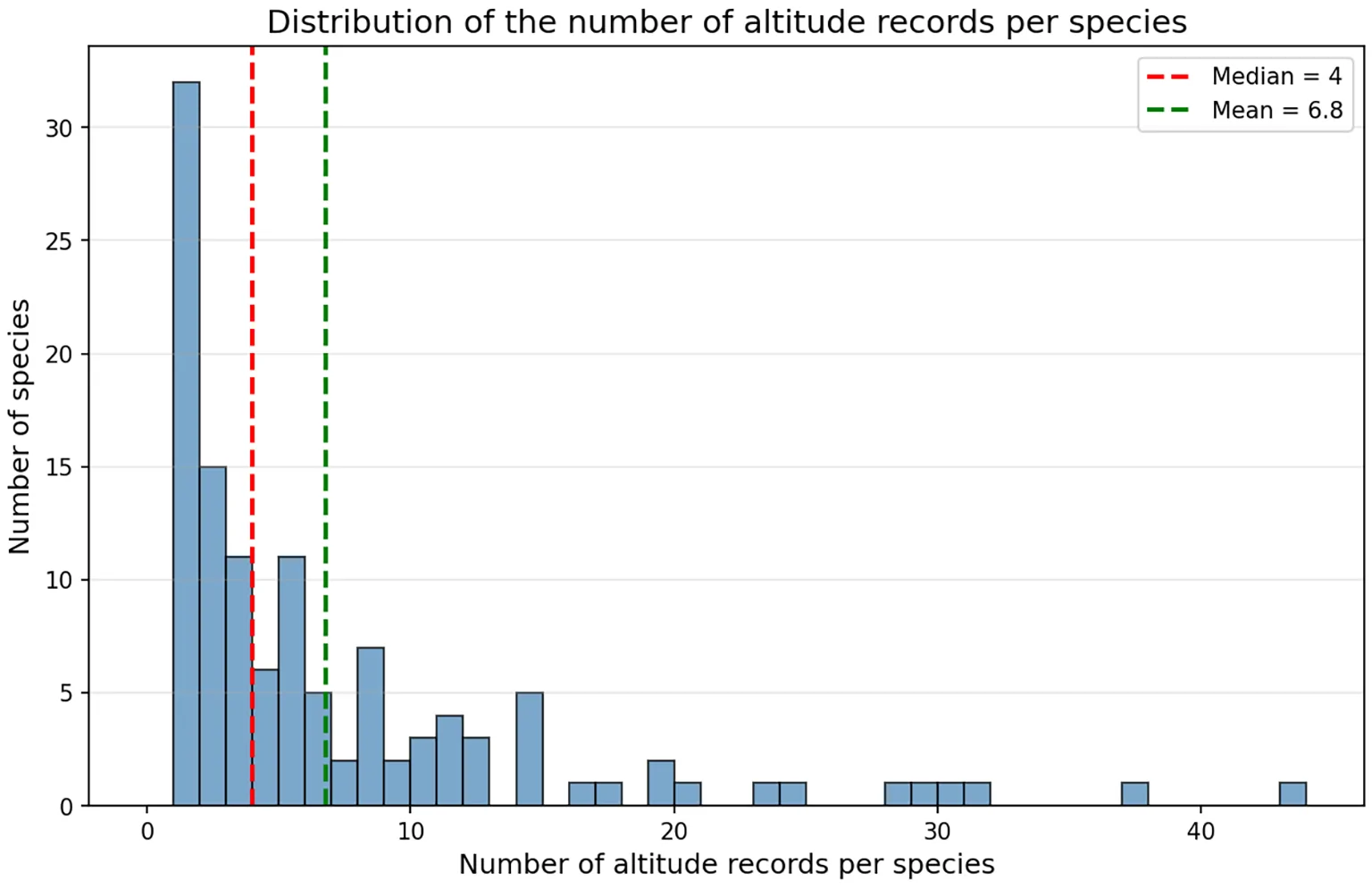
Distribution of the number of altitude records per species. Red dashed line: median = 4; green dashed line: mean = 6.8.

The statistical processing of the collected data was performed for 119 taxa (117 species and 2 varieties) with reliably identified collection localities, totaling 808 identification records with reliably determined elevations. The distribution of the number of altitude records per taxon is summarized in **Figure 2**. The actual elevations of the collection localities represented by these records range from 230 to 2075 m a.s.l.

For the statistical analysis, we employed the range-density Maximum Likelihood Estimation (MLE) framework described by Klimova et al. (2024), with the input data adapted from binary locality-level presence/absence data to occurrence-record frequencies. This method assumes that the altitudinal distribution of each taxon can be approximated by a Gaussian response curve characterized by the mean μ (the estimated center of altitudinal distribution) and standard deviation σ (characterizing the breadth of the altitudinal range). Unlike classical maximum likelihood estimation applied directly to raw elevations, the range-density approach accounts for uneven occurrence-record density across the elevation gradient, thereby correcting biases that arise when some elevation zones are sampled and identified more intensively than others.

In the present account, we retain the general range-density (RD) MLE framework of Klimova et al. (2024), but adapt its input from binary locality-level presence/absence data to repeated occurrence/identification records. We additionally introduce a *post hoc* diagnostic check for isolated altitudinal occurrences (Section 2.4.1.3), using the full window width 2d as the natural isolation threshold. Records that are observationally disconnected from the main cluster of a given taxon (separated by a gap > 2d) are excluded from the corresponding per-taxon core fit, but the range-density estimator itself is otherwise unchanged.

#### 2.4.1 Range–Density Maximum Likelihood Estimation (RD MLE)

Following the methodology described by Klimova et al. (2024), we assume that the relative occurrence intensity of a given taxon along an elevational gradient can be approximated by a Gaussian response curve:


$$\varphi \left(x;\;\mu ,\;\sigma \right)\;=\;\frac{1}{\sigma \sqrt{2\pi }}\cdot exp\left(\frac{-{\left(x\;-\;\mu \right)}^{2}}{2\sigma^{2}}\right)$$

where x represents elevation (m a.s.l.), μ is the distribution center, and σ is the standard deviation characterizing the spread of the distribution.

The key innovation of the range-density MLE approach in this account lies in the construction of the range-density function g(x), which accounts for the varying density of records at different elevations. In the present adaptation, H denotes the multiset of occurrence/identification records used in the analysis; each record h ∈ H has an elevation and a taxon identity. For each taxon t and elevation x, we define a subset of records within a window of radius d from the elevation x:


$$H_{x}^{\prime }\;=\;\left\{h\;\in \;H\;:\;\left|h\;-\;x\right|\;\leq \;d\right\}$$

The range-density function is then defined as the ratio of occurrence records belonging to the taxon to the total number of occurrence records within this subset:


$$g\left(x\right)\;=\;\frac{\sum_{H_{x}^{\prime }}f\left(t,\;h\right)}{\left|H_{x}^{\prime }\right|}$$

where f(t, h) is the indicator function equal to 1 if record h belongs to taxon t and 0 otherwise, and |H’_x| = n(x) is the number of occurrence records in the window. This function represents a moving estimate of the relative occurrence frequency of the taxon across the elevation gradient, implemented through a rectangular window function.

##### 2.4.1.1 Determination of the window radius d

The radius d of the window function is a critical parameter of the method. If d is too small — less than the typical elevation interval between consecutive records for a given taxon — the function g(x) fragments into isolated peaks separated by zero values, and fitting a smooth Gaussian response curve becomes unreliable. Conversely, if d is too large, the function g(x) becomes overly smoothed, and important details of the altitudinal distribution may be lost.

**Figure 3:**
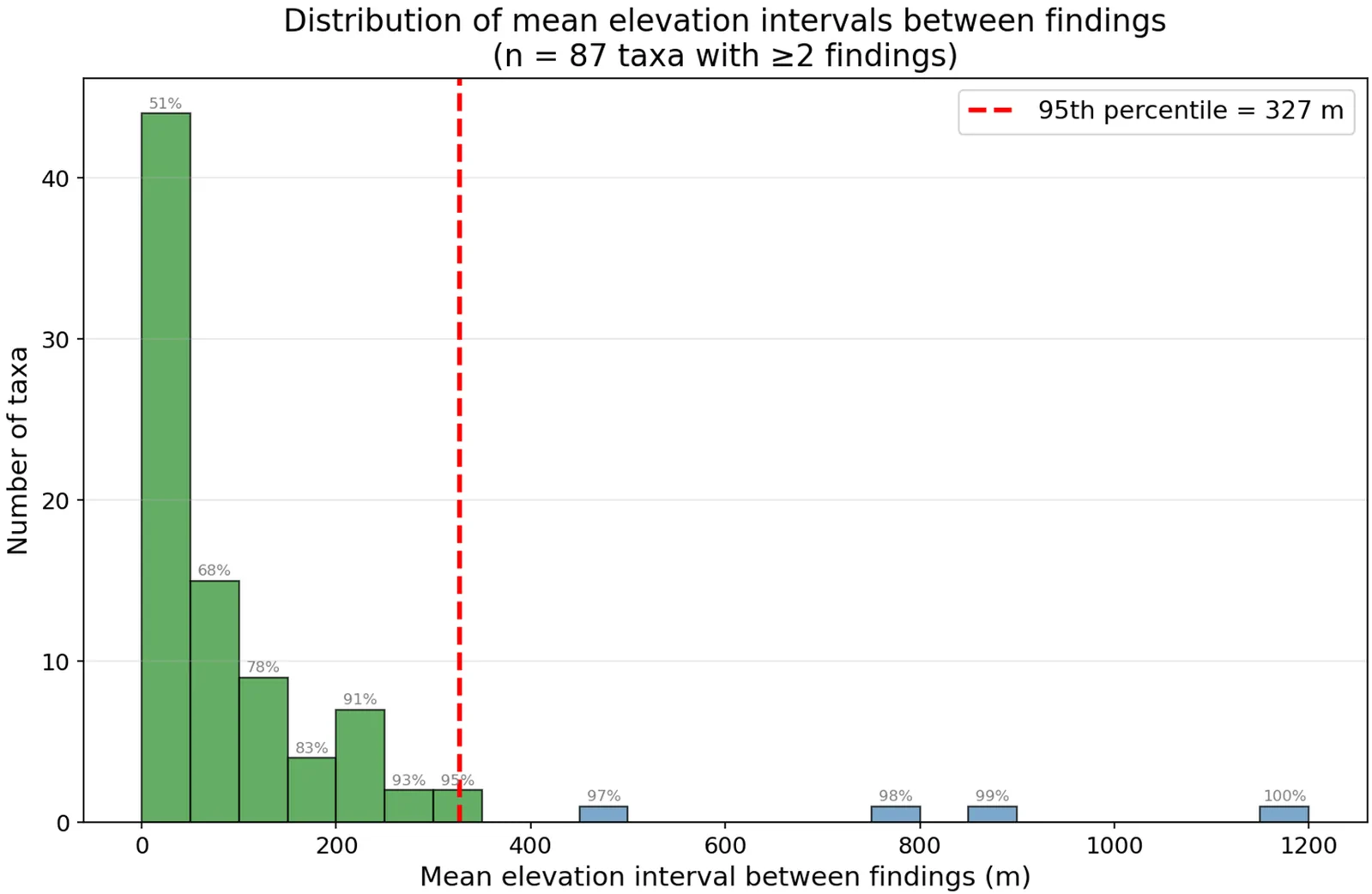
Distribution of mean elevation intervals for 87 taxa with two or more occurrence records. Red dashed line: 95th-percentile threshold d = 327 m.

To determine the appropriate value of d, we analyzed the elevation intervals between consecutive records for each taxon. For all taxa with two or more occurrence records (n = 87), we computed the mean elevation interval between consecutive sorted altitude values. The distribution of these mean intervals is shown in **Figure 3**. Repeated identical elevations of the same taxon contribute zero-length intervals to this calculation and therefore lower the resulting value of d slightly; this is a deliberate consequence of using occurrence records, rather than localities, as the unit of observation.

The histogram (**Figure 3**) shows a strongly right-skewed distribution. The majority of taxa have mean inter-finding intervals below 150 m, but a long tail extends to over 1000 m, corresponding to taxa with few, widely scattered records.

The value of d must be chosen so that the window function captures the local occurrence pattern for the vast majority of taxa while remaining robust against the few outlier taxa with anomalously large intervals. We set d equal to the 95th percentile of the distribution of mean inter-record intervals. This percentile was used as an empirical smoothing rule: it exceeds the mean inter-record interval for 95% of taxa while avoiding a window so broad that genuine distributional features would be obscured.

For the present dataset, the 95th percentile equals 326.7 m. Therefore, we set d = 326.7 m, which corresponds to a total window width of 2d = 653.3 m; both values are rounded to 327 m and 653 m elsewhere in the text and in the figures. This value captures the local occurrence pattern for most analyzed taxa while avoiding excessive smoothing.

Numerical fitting of the Gaussian response curve was performed in Python using the SciPy (2026) library.

##### 2.4.1.2 The boundary sampling problem

In the original formulation of the range-density MLE (Klimova et al., 2024), the parameters μ and σ are estimated by fitting a Gaussian curve to the normalized function g(x)/max(g). This approach works well when the denominator n(x) within the window is sufficiently large at all evaluated elevation spans. In the present occurrence-record dataset, however, the density of occurrence records is strongly asymmetric: the number of records falling inside the window, n(x), reaches a maximum of 558 near 690 m a.s.l., declines to 13 between 1800 and 1980 m, and falls to 6–9 above 2000 m, a variation of almost two orders of magnitude. It should be stressed that n(x) describes the density of the available occurrence records and does not, by itself, distinguish reduced field effort from the limited extent or availability of the corresponding habitats.

This denominator imbalance creates a systematic artifact in g(x). At elevations where n(x) is small, even a single finding produces a disproportionately large value of g(x) = k/n. For example, a single taxon occurrence among 9 total records yields g = 0.111, whereas the same single taxon record among 355 total records in a densely recorded part of the gradient yields g = 0.003. The normalization step g(x)/max(g) then amplifies these unstable estimates whenever the maximum of g(x) falls in a region with a small denominator.

This boundary effect manifests in two ways: (1) for taxa with isolated high-altitude occurrences, the fitted normal distribution may be pulled toward the sparse boundary, away from the main concentration of occurrences; and (2) near the edges of the sampled range, g(x) exhibits upward spikes that do not reflect genuine distributional patterns but merely the low denominator n(x). This is a well-known problem in binomial proportion estimation: Var(g) ≈ g(1−g)/n(x), so estimates at small n(x) are highly unreliable (Casella and Berger, 2002).

A further consequence of the same asymmetry concerns the admissible range of the fitted parameter. Because the range-density function is evaluated, and the response curve fitted, over the sampled interval, μ is constrained to [h_min, h_max] = [180, 2125] m, that is to the observed elevational range extended by 50 m at each end. A taxon whose range-density optimum would fall outside the sampled gradient therefore receives a fitted μ at the corresponding bound; in the present dataset this applies to 24 taxa at the lower bound and to five at the upper bound. The lowest and the highest altitudinal groups are consequently best understood as boundary groups, whose members are taxa whose fitted optima are limited by the ends of the profile, rather than as groups with precisely located centres. Their membership, however, is not an artefact of the constraint: refitting all taxa with the bounds widened to [0, 3000] m, the interval used by Klimova et al. (2024), and to [−2000, 5000] m reproduces exactly the same partition of the flora (24, 54, 35 and 6 taxa in groups A–D), the previously bounded estimates relaxing to μ ≤ 87 m and to μ = 2278–2551 m respectively. These relaxed values are extrapolations of the fitted response beyond the observed gradient, not estimates of actual optima; they show only that it is the constraint, and not a feature of the data, that fixes the numerical value at the bound. What the bounds censor is the numerical value of μ for these 29 taxa, not their assignment to a group.

##### 2.4.1.3 Diagnostic analysis of isolated altitudinal occurrences

An inherent limitation of the range-density MLE method becomes apparent when a taxon has occurrences distributed unevenly across the gradient: a single occurrence at an extreme elevation, separated from the main cluster by a large sampling gap, inflates g(x) near the range boundary and displaces the fitted distribution center (see Section 2.4.1.2). Such isolated occurrences are either genuinely rare extreme records of a euryzonal taxon, or taxonomic artifacts (morphologically similar but taxonomically distinct, possibly undescribed or misidentified species collected at widely separated elevations). In either case, they should be flagged so that they do not distort the core-fit estimate of the typical altitudinal range of the taxon.

To identify such cases, we introduce a *post-hoc* diagnostic check that uses the full window width (2d = 653 m) as the natural isolation threshold. The choice of 2d is not arbitrary: the window function in the range-density construction (Section 2.4.1) already defines 2d as the characteristic length of local connectedness — the distance over which two records are still jointly observable by at least one sliding-window position. If two occurrences of the same taxon are separated by a gap exceeding one full window width, no sliding-window position can simultaneously contain both of them, and the two occurrence groups are observationally disconnected in the g(x) calculation. Using 2d therefore keeps the diagnostic consistent with the core method and introduces no new tunable parameters.

For each taxon, the sorted sequence of observed altitudes is examined for consecutive gaps. If no gap exceeds 2d, all records form one connected cluster. If at least one gap exceeds 2d, the sequence is partitioned into contiguous segments; the segment containing the most records is designated the main cluster, and records in other segments are considered isolated. Five diagnostic status categories are defined:

OK: all records form one connected cluster (no gap > 2d). The RD-MLE estimate is considered as reliable.

Questionable: exactly one record is isolated from the main cluster by a gap > 2d. The isolated record is excluded from the core fit, and the result requires attention before ecological interpretation.

Needs taxonomic check: two or more records are isolated from the main cluster. All isolated records are excluded from the core fit; the pattern itself suggests that morphologically similar but taxonomically distinct taxa may have been collected at widely separated elevations, and the original specimens warrant critical taxonomic review.

Single record: the taxon is represented by only one occurrence record; isolation cannot be assessed. Such taxa are kept in the analysis with their single datum but carry no internal cross-check.

Two-record unresolved: the taxon is represented by two records separated by a gap > 2d. Such cases are flagged, but no record is excluded because the data do not define a unique main cluster.

Crucially, isolated records in the Questionable and Needs taxonomic check categories are not used in the MLE and RD-MLE fits for the corresponding taxon: the parameters μ and σ reflect only the main cluster. In Two record unresolved cases no record is excluded, because the data do not define a unique main cluster. The occurrence-record density denominator n(x) is kept unchanged, because all records, including those excluded from the fit of a particular taxon, remain part of the empirical record-density profile along the elevation gradient.

**Figure 4:**
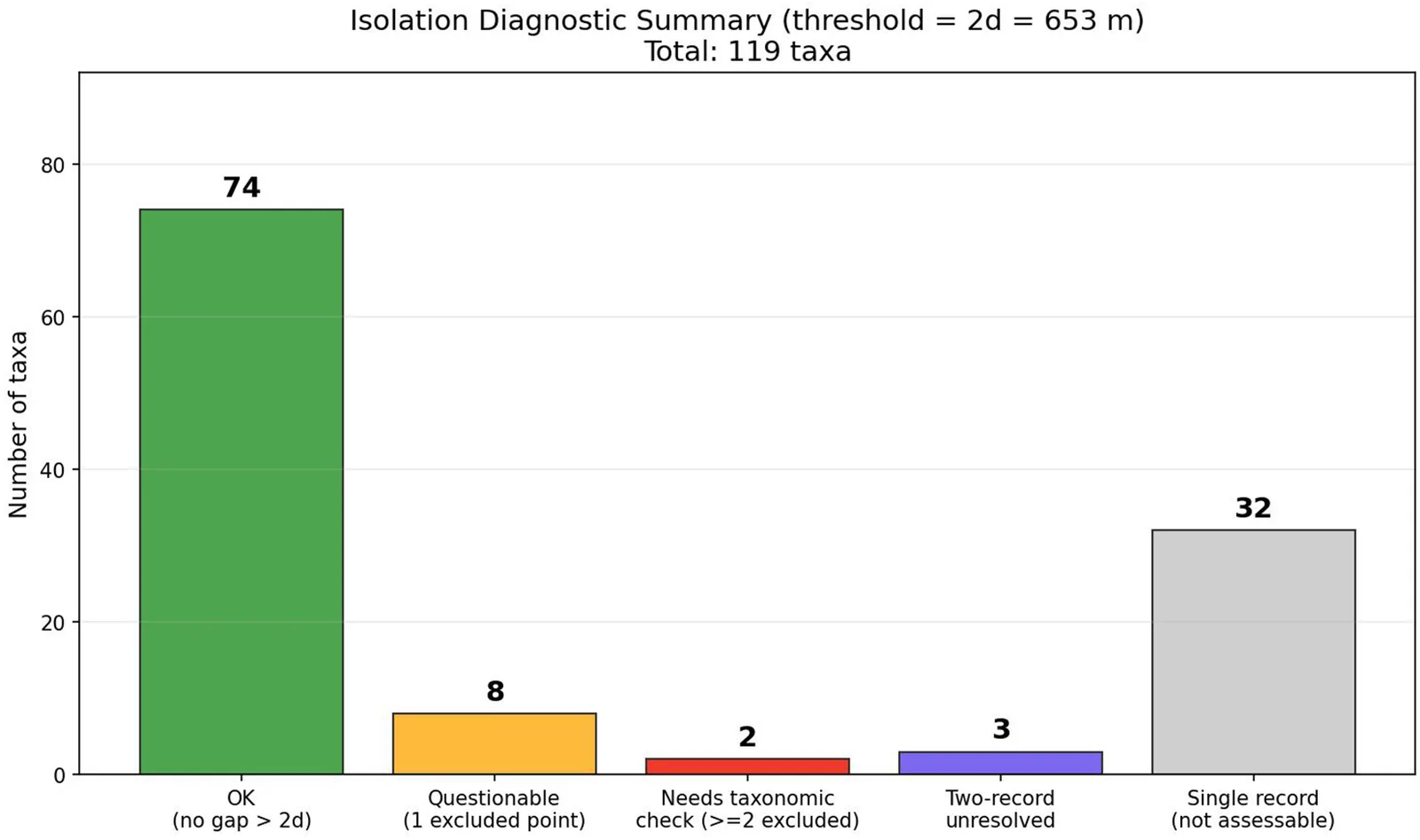
Isolation diagnostic summary for all 119 analyzed taxa. The isolation threshold equals one full window width (2d = 653 m) — the same length scale that defines local connectedness in the RD-MLE g(x). A consecutive altitude gap exceeding 2d separates isolated occurrences from the main cluster; such records are excluded from the per-taxon core fit except in two-record unresolved cases, where no unique main cluster can be defined.

Of the 119 analyzed taxa, 74 received OK status, 8 Questionable, 2 Needs taxonomic check, 3 Two record unresolved, and 32 were single record taxa. In total, 13 isolated occurrence points were excluded from the corresponding per-taxon core fits; three additional two-record cases were flagged but not reduced. The distribution of statuses is shown in **Table 4**; **Figure 4**, and the complete list of affected taxa is given in Section 2.4.1.6.

In the species distribution plots (**Online Appendix 2**), isolated occurrence points are drawn with a red × marker; they are shown for transparency but do not contribute to the plotted curves or the reported μ and σ values.

##### 2.4.1.4 Illustrative example

**Figure 5:**
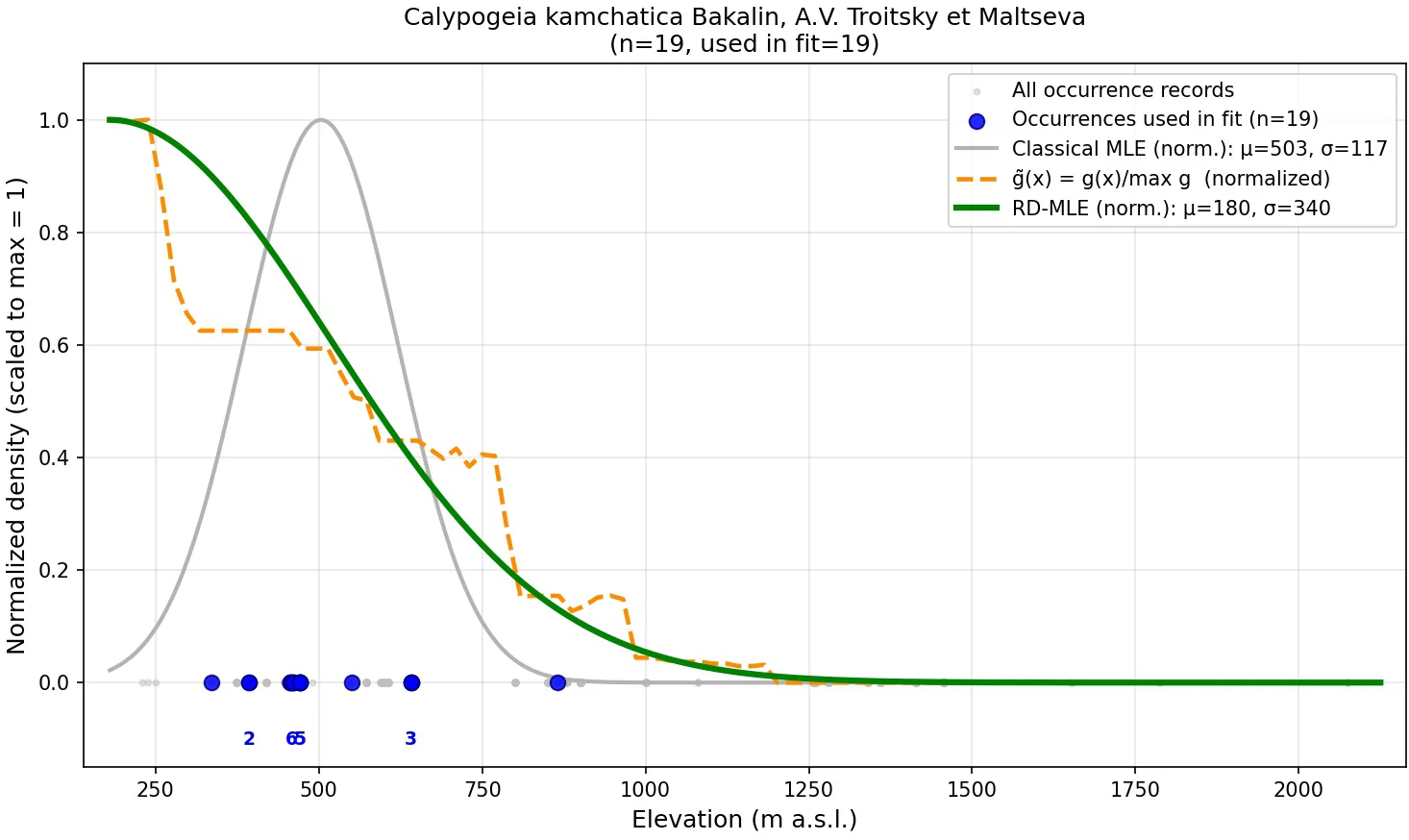
Illustrative example: Calypogeia kamchatica Bakalin, A.V. Troitsky et Maltseva (ID 10, n = 19, used in fit 19). Gray: classical MLE (μ = 503 m, σ = 117 m); orange dashed: $\tilde{g}\left(\text{x}\right)$; green: RD-MLE (μ = 180 m, σ = 340 m).

**Figure 5** illustrates the method for *Calypogeia kamchatica* Bakalin, A.V. Troitsky et Maltseva (ID 10, n = 19 occurrence records, 19 used in the fit after the isolation diagnostic). The plot shows three elements corresponding to successive stages of the analysis:

The gray curve is the classical MLE fit (μ = 503 m, σ = 117 m), obtained by fitting a normal distribution directly to the main-cluster elevation data. This estimate does not account for the varying density of occurrence records and may be biased near range boundaries.

The orange dashed curve is the normalized range-density function $\tilde{g}\left(\text{x}\right)$ = g(x)/max g, where g(x) = k(x)/n(x) is the ratio of the number of taxon records k(x) to the total number of occurrence records n(x) within a window at the current elevation. Boundary-related oversampling of rare records is therefore explicitly visible in the range-density function.

The green solid curve is the final result — the Gaussian response curve fitted to g(x) by the range-density MLE procedure (μ = 180 m, σ = 340 m). Where multiple occurrence records fall within 5 m, the blue numbers below the axis indicate the count of overlapping points.

**Figure 6:**
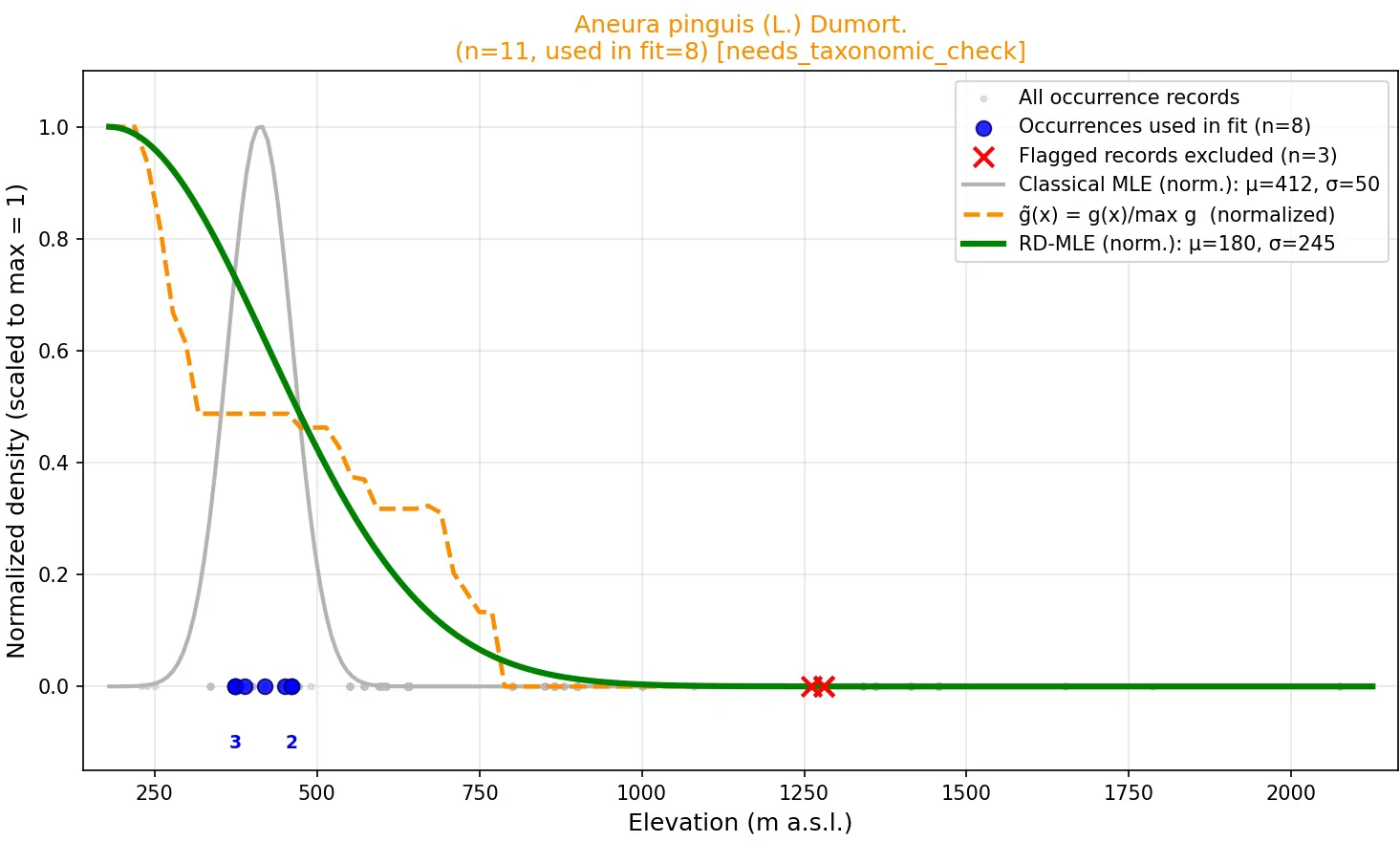
Aneura pinguis (L.) Dumort. (ID 2, n = 11, used in fit = 8). Red ×: excluded isolated records; diagnostic status: Needs taxonomic check. Gray: classical MLE (μ = 412 m, σ = 50 m); orange dashed: $\tilde{g}\left(\text{x}\right)$; green: RD-MLE (μ = 180 m, σ = 245 m).

For comparison, **Figure 6** shows the analogous plot for *Aneura pinguis* (L.) Dumort. (ID 2, n = 11, used in fit = 8 after the isolation diagnostic). Classical MLE: μ = 412 m, σ = 50 m; RD-MLE: μ = 180 m, σ = 244 m. Three isolated records are excluded from the fit and marked with a red ×; the taxon is assigned the status Needs taxonomic check. Analogous plots for all analyzed taxa are presented in **Online Appendix 2**.

##### 2.4.1.5 Diagnostic reliability check: case study

**Figure 7:**
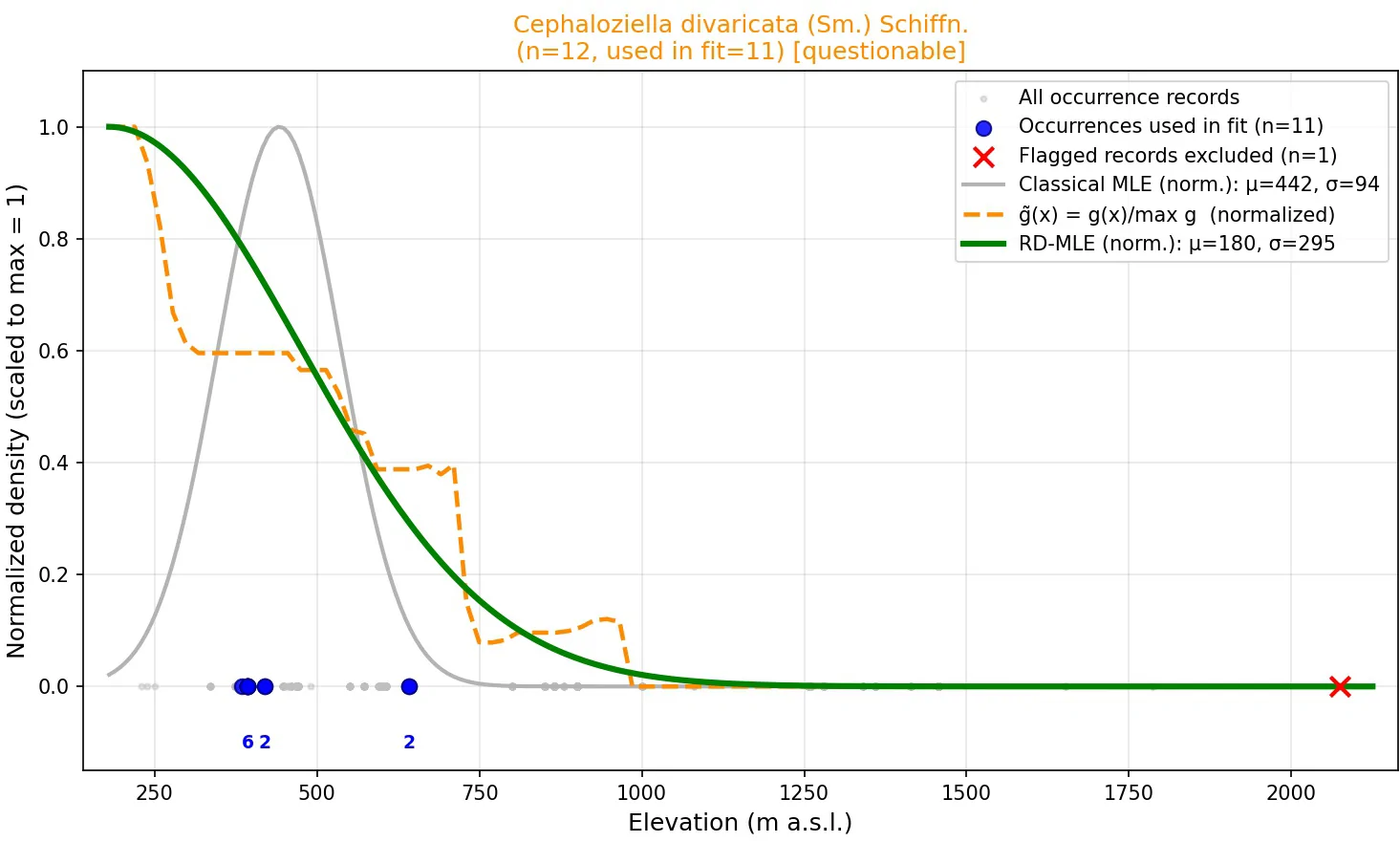
Taxon No. 15 (Cephaloziella divaricata (Sm.) Schiffn.), n = 12 records, 11 retained after the isolation diagnostic. Red ×: excluded record at 2075 m (gap ~1434 m > 2d). Curves are fitted to the main cluster only; the resulting RD-MLE estimate is μ = 180 m, σ = 295 m. Diagnostic status: Questionable.

The isolation diagnostic is most clearly illustrated by taxon No. 15, *Cephaloziella divaricata* (Sm.) Schiffn. (n = 12) (**Figure 7**). The majority of occurrence records are concentrated between 384 and 641 m a.s.l. A single occurrence at 2075 m is separated from this cluster by a gap of approximately 1434 m — more than twice the full window width (2d = 653 m), and therefore unambiguously isolated by the criterion of Section 2.4.1.3.

Before the diagnostic, the 2075 m record disproportionately inflated g(x) near the upper range boundary (where the density of occurrence records is very low, n(x) is approximately 6-9) and pulled the fitted curve toward the summit. After excluding this single isolated record, the fit is recomputed on the remaining 11 records of the main cluster and yields μ = 180 m, σ = 295 m — an ecologically plausible center of distribution for a lower-montane taxon.

Two interpretations of the excluded record remain possible: (1) the taxon genuinely occupies an unusually wide altitudinal span, with this 2075 m collection being a rare but authentic extreme record of a euryzonal species; or (2) the high-altitude specimen represents a morphologically similar but taxonomically distinct alpine taxon, making it a potential taxonomic artifact. In both cases, the ecological “typical” position of the taxon is better represented by the main cluster, and the original specimen is recommended for critical taxonomic review.

This case illustrates the general reasoning behind the diagnostic: under a strongly uneven density of occurrence records, the boundary inflation of g(x) is a structural property of the estimator rather than a sign of ecological reality. By excluding records that are observationally disconnected from the main cluster (gap > 2d) from the core fit, while retaining them in the raw occurrence dataset, we preserve the principled form of RD-MLE while preventing it from being driven by single outliers.

##### 2.4.1.6 List of flagged taxa

**Table 1** lists all 13 taxa flagged by the isolation diagnostic described in Section 2.4.1.3. For each taxon, the table gives the full number of records (n), the number of records retained in the main cluster and used for the fit (n_used), the largest altitudinal gap found in the sorted records (max_gap, in metres), the excluded or flagged altitudes, and the diagnostic status. Ten taxa have μ and σ estimates in **Supplementary Table 3** based on a reduced subset of records; three two-record cases are flagged as unresolved but retain both records in the fit.

**Table 1:** The 13 taxa flagged by the isolation diagnostic (Section 2.4.1.3).

ID	Taxon	n	n_used	max_gap (m)	Excluded altitudes (m)	Status
2	Aneura pinguis (L.) Dumort.	11	8	801	1261, 1280, 1280	needs taxonomic check
15	Cephaloziella divaricata (Sm.) Schiffn.	12	11	1434	2075	questionable
21	Cryptocoleopsis imbricata Amak.	16	15	683	597	questionable
22	Diplophyllum albicans (L.) Dumort.	7	6	707	573	questionable
30	Gymnocolea inflata (Huds.) Dumort.	8	6	767	420, 573	needs taxonomic check
51	Lophozia murmanica Kaal.	2	2	892	flagged; not excluded	two-record unresolved
60	Marsupella apertifolia Steph.	2	2	787	flagged; not excluded	two-record unresolved
62	Marsupella boeckii (Austin) Lindb. ex Kaal.	3	2	659	1256	questionable
65	Marsupella tubulosa Steph.	5	4	683	1280	questionable
73	Nardia harae Amakawa	5	4	851	1457	questionable
74	Nardia insecta Lindb.	6	5	860	1457	questionable
100	Scapania obcordata (Berggr.) S.W. Arnell	2	2	1175	flagged; not excluded	two-record unresolved
117	Sphenolobus minutus (Schreb.) Berggr.	8	7	790	471	questionable

“Excluded or flagged altitudes” are records that lie in segments other than the main cluster, separated by one or more gaps > 2d. In two-record unresolved cases, both records are retained in the fit.

#### 2.4.2 Robustness and sensitivity analyses

Sensitivity of the altitudinal grouping to the histogram. Because the altitudinal groups are read from a histogram of μ, we assessed how far this reading depends on the histogram itself. The histogram was reconstructed for bin widths of 100, 150, 200, 250, 300 and 400 m and, because the apparent position of a trough depends also on where the bins begin, for three bin origins (0, 50 and 100 m), giving 18 configurations. For each configuration the interior local minima of the counts were recorded, and each adopted cutpoint was checked against them under two criteria: a strict one, counting a cutpoint as recovered when it lies inside a minimum bin, and a tolerant one, counting it as recovered when a minimum bin lies within 100 m of it; both counts are reported in **Supplementary Table 4**. In addition, a bin-free Gaussian kernel density estimate of the μ distribution was computed at bandwidths of 0.10–0.30 and its modes and minima located, and the ordered μ series was examined directly for empty intervals. Full results are given in **Supplementary Table 4** and **Supplementary Figure 1**.

Exclusion of single-record taxa. The effect of the 32 taxa represented by a single occurrence record was assessed at two levels. First, the group tabulation was repeated for the 87 taxa with two or more records, retaining the μ estimates of the primary analysis and the boundaries A: μ< 300 m; B: 300 ≤ μ ≤ 1100 m; C: 1100< μ ≤ 1900 m; D: μ > 1900 m. Second, because the denominator n(x) of the range-density function is built from all occurrence records, the 32 records of the single-record taxa were also removed from H, n(x) was rebuilt from the remaining 776 records, and μ and σ were re-estimated for all 87 retained taxa. The window radius d is estimated only from taxa with two or more records and is therefore unaffected by this removal. Taxon-level values and group assignments under both procedures are listed in **Supplementary Table 5**.

Goodness of fit of the Gaussian response. Because a single Gaussian curve cannot represent an asymmetric or locally multimodal empirical response, goodness of fit was quantified for every taxon with two or more records. The coefficient of determination R^2^ was computed between the normalized empirical range-density function g(x)/max(g) and the fitted curve over the evaluated elevation grid. R^2^ is used here as a descriptive measure of curve shape and not as a formal test, because neighbouring points of g(x) are strongly autocorrelated: consecutive grid positions share most of the records inside the window of radius d. Values for all taxa are given in **Supplementary Table 6**, and the empirical and fitted curves for every taxon are shown in **Supplementary Online Appendix 2**.

Filtered versus unfiltered fits. To quantify the influence of the isolation diagnostic, the ten taxa from which isolated records had been removed were refitted on their complete record sets. Filtered and unfiltered μ estimates, group assignments and aggregate group sizes were then compared taxon by taxon (**Supplementary Table 6**).

Dependence-preserving comparison of vegetation belts. Because the same taxon may be recorded in more than one vegetation belt, the belt-level taxon sets overlap and are not independent samples: of the 119 taxa, 57 occur in one belt, 36 in two, 19 in three and 7 in four, and CF and TB share 41 taxa. Intervals calculated from Student’s t-distribution were therefore retained only as marginal descriptive summaries of individual belts and are not used for pairwise comparison. Belts were instead compared by a dependence-preserving taxon-level bootstrap: in each of 10,000 replicates, 119 taxa were drawn with replacement from the taxon list, and every resampled taxon contributed its μ simultaneously to all belts in which it had been recorded, so that the covariance induced by shared taxa is reproduced. Percentile intervals (2.5th and 97.5th) were computed for belt means and for pairwise differences of belt means; the two-sided tail probability reported below is twice the smaller of the proportions of replicates in which the difference was at most zero and at least zero. To compare the elevational proximity of PF to TB and to CF directly, the distance contrast |mean μ(PF) - mean μ(CF)| - |mean μ(PF) - mean μ(TB)| was additionally evaluated over the same replicates; positive values indicate that PF lies closer to TB than to CF. All resampling used a fixed random seed (12345, NumPy default_rng); the analysis scripts are available from the authors.

Compositional similarity of belt floras. Floristic similarity among the belt floras was quantified from taxon presence/absence using the Jaccard index J = |A ∩ B|/|A ∪ B| and the Sørensen index S = 2|A ∩ B|/(|A| + |B|); AH was excluded from this comparison because only nine taxa were recorded in it. Both indices are symmetric and are therefore constrained by differences in flora size: with 23 taxa in PF against 64 in CF and 66 in TB, even complete inclusion of the PF flora within one of them could not raise J above 0.35. For this reason the overlap coefficient |A ∩ B|/min(|A|, |B|), which expresses the proportion of the smaller flora that is shared, is reported in the text for the PF comparisons, and the belt floras were additionally compared by their composition in terms of the altitudinal groups A–D. Because the belt floras share taxa and several expected cell counts are small, a contingency-table test is not appropriate here; instead, the L1 distance between group-composition profiles was computed, and the contrast L1(PF, CF) − L1(PF, TB) was evaluated with the same taxon-level bootstrap, each resampled taxon retaining its group assignment together with all its belt memberships.

## 3 Results

### 3.1 Taxonomic diversity in the Nalychevo Nature Park

A total of 283 specimens were collected and 494 identifications were made from the collection gathered in 2022. After the identification results were summarized, a species list was obtained that includes 94 species. Comparison of the obtained list with the previously published one showed that we identified 35 species in the park for the first time, whereas 25 of those on the previously published list were not found in the course of the research conducted in 2022. The total diversity in the park reached 119 taxa (117 species and two varieties).

Anatomical and morphological analysis of the collection yielded one species new for the Kamchatka flora: *Aneura maxima* (Schiffn.) Steph. However, the actual meaning of this name in this context remains unclear. It was previously shown (Bączkiewicz et al., 2017) that this species, as traditionally circumscribed, is clearly paraphyletic and distributed among several clades of cryptic species within the *Aneura pinguis* (L.) Dumort group. Formal descriptions for these cryptic taxa have not yet been provided, so by using this name we are simply referring to another (as yet undescribed) species. Information on the specimen collection localities is provided in **Supplementary Tables 1** and **2**. *Radula prolifera* Arnell is excluded from the Nalychevo Park flora. The species was reported based on four specimens in Bakalin and Klimova (2018) (K-66-2-15, K-66-8-15, K-66-14-15, K-66-17-15); in the course of the preparation of the present account we re-examined all of them and found that they belonged to *R. polyclada*. Indeed, the two taxa are somewhat similar due to numerous small-leaved branches. However, as discussed in Bakalin and Klimova (2020), branches in *R. polyclada* are larger and always bear leaves pronounced size difference between the ventral and dorsal lobes, while in *R. prolifera* lobes are subequal. The newly reported taxa are highlighted in blue in the checklist provided in **Supplementary Table 2** as a table with the structure described in the Materials and Methods chapter.

### 3.2 Distribution parameters of the analyzed taxa

**Figure 8:**
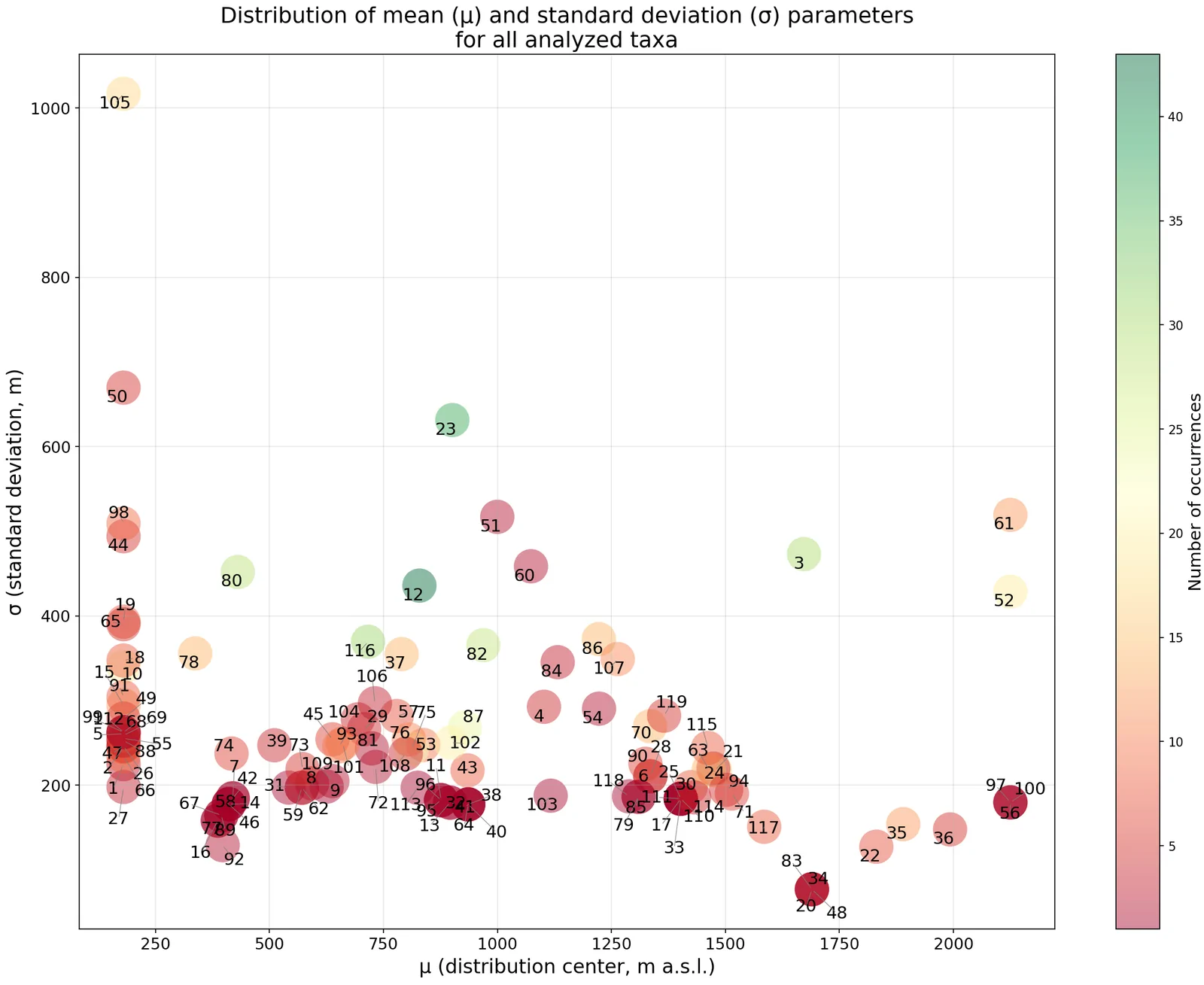
Distribution of μ and σ for all 119 analyzed taxa (RD-MLE).

The parameters μ and σ were estimated for all 119 analyzed taxa using the range-density MLE method. **Figure 8** shows the distribution of these parameters in the μ-σ coordinate plane. The abscissa represents the estimated center of the altitudinal distribution (μ), while the ordinate characterizes the breadth of the distribution (σ). The color of each marker indicates the number of occurrence records for the taxon, ranging from single records (red) to a maximum of 43 records (green).

Several important patterns are visible in **Figure 8**. Taxa in the lower part of the diagram (low σ values) exhibit a narrow altitudinal range, meaning they are confined to a relatively narrow elevation band. Conversely, taxa in the upper part (high σ values) show a broad, more uniform distribution across the entire studied altitudinal gradient. The majority of the analyzed taxa have σ values below 400 m, indicating relatively specialized altitudinal preferences.

**Figure 9:**
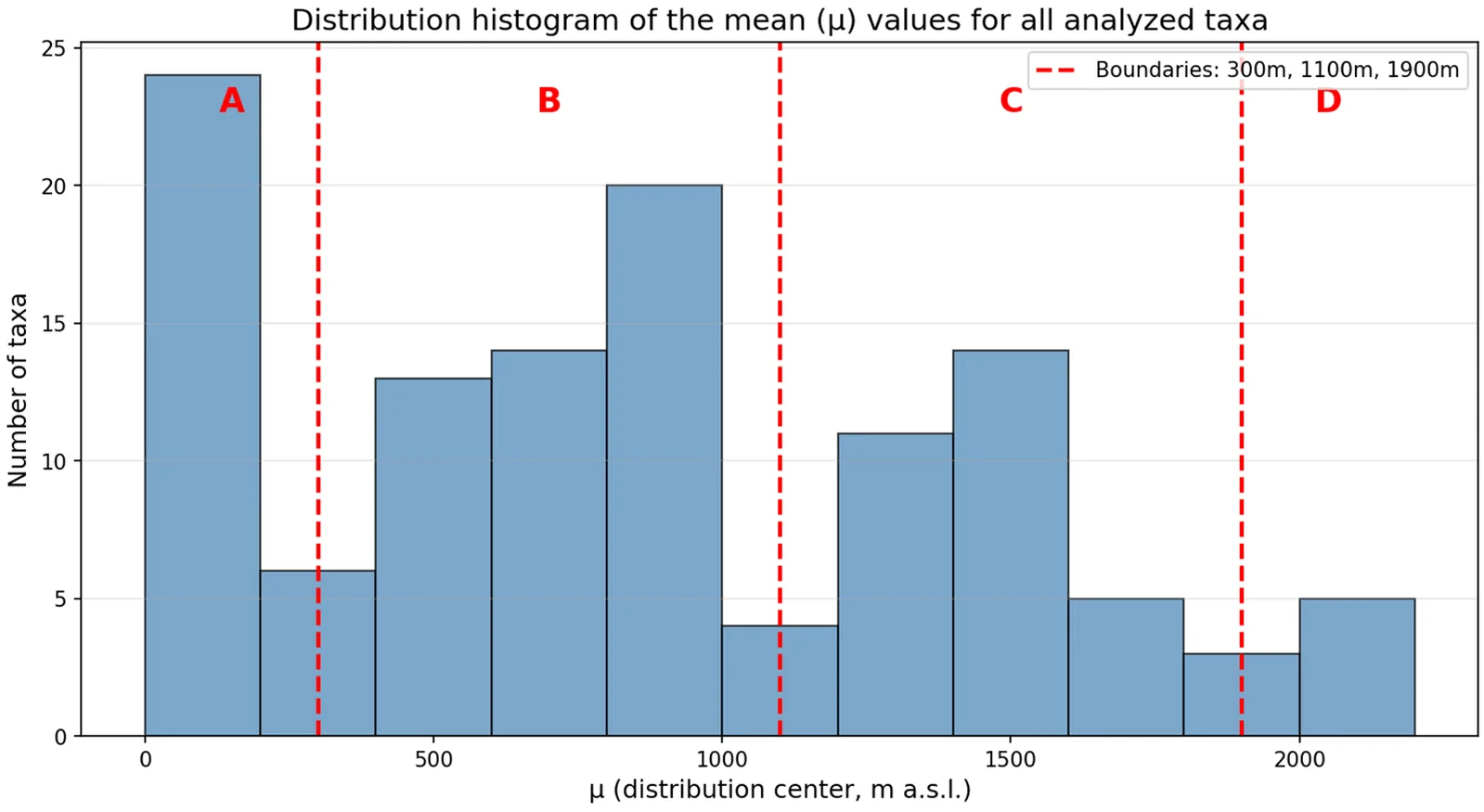
Histogram of μ values (RD-MLE). Red dashed lines: group boundaries. **(A–D)** Altitudinal groups.

The horizontal distribution of points reveals a non-uniform pattern: there are clearly identifiable clusters of taxa with similar distribution centers. This observation suggests the existence of distinct altitudinal groups, which can be identified more precisely from the histogram of μ values (**Figure 9**).

#### 3.2.1 Goodness of fit of the Gaussian response

The empirical range-density curves are described well by a single Gaussian response for the great majority of taxa. Across all 87 taxa with two or more occurrence records the median R^2^ was 0.81, with 70% of taxa exceeding 0.70 and 85% exceeding 0.50. Among the 13 most fully represented taxa (n ≥ 15) R^2^ ranged from 0.44 to 0.96 with a median of 0.82: Pellia neesiana 0.96, Scapania paludosa 0.94, Calypogeia kamchatica 0.92, Solenostoma subellipticum 0.90, Anthelia juratzkana 0.87, Nardia japonica 0.86, Pleurocladula albescens 0.82, Cryptocoleopsis imbricata 0.78, Cephalozia bicuspidata 0.76, Pseudolophozia sudetica 0.76, Lophozia savicziae 0.76, Scapania subalpina 0.61 and Diplophyllum taxifolium 0.44. Only the last of these shows a broad, irregular response that a single symmetric curve does not reproduce; it is discussed in Section 4.2. Values for all taxa are given in **Supplementary Table 6**.

### 3.3 Division into altitudinal groups

To identify the altitudinal grouping of taxa, we constructed a histogram of the μ values for all analyzed taxa with a bin width of 200 m (**Figure 9**). The histogram was also reconstructed at the alternative bin widths and bin origins, and by kernel density estimation, as described in Section 2.4.2.

The histogram reveals four clearly distinct groups separated by pronounced minima in the distribution. The sensitivity analysis shows that this reading holds over a broad, although not unlimited, range of resolutions, and that the two internal boundaries are supported in different ways. The B/C trough is the most robust feature: an interior histogram minimum containing 1100 m appeared in 17 of the 18 combinations of bin width and bin origin, two adjacent minima bracketed it in the remaining one, and the bin-free kernel density estimate places this minimum at 1129–1157 m at every bandwidth from 0.15 to 0.30. The C/D trough corresponds to the empty interval of the μ series between 1890 and 1993 m: it is resolved as a distinct histogram minimum at fine and moderate resolutions, merges with neighbouring features at the coarsest widths (300–400 m), and is placed at 1913–1928 m by the kernel estimate at bandwidths of 0.15–0.25, the two upper modes merging at 0.30. The kernel estimate resolves four modes at bandwidths of 0.20–0.25; at 0.15 it additionally separates a small cluster of taxa centred at 390–430 m from the rest of group B (minima at 341 and 537 m), that is, it subdivides group B without displacing any adopted boundary. The lowest cutpoint falls inside the largest empty interval of the ordered μ series, no taxon having an estimated center between 180 and 337 m. The number of groups and the approximate position of the internal minima are therefore stable over a broad range of resolutions, whereas the exact cutpoint values are defined only to within about one bin width, and we retain 300, 1100 and 1900 m as operational cutpoints. The alternative histograms and the kernel density estimates are shown in **Supplementary Figure 1**. On this basis we define four altitudinal groups:

Group A (lower belt, μ < 300 m) comprises 24 taxa. Group B (middle belt, 300–1100 m) is the most numerous, comprising 54 taxa. Group C (upper belt, 1100–1900 m) includes 35 taxa. Group D (alpine belt, μ > 1900 m) contains 6 taxa. Excluding the 32 taxa represented by a single occurrence record leaves the same four occupied groups. Retabulating the 87 remaining taxa under the same boundaries gives 24, 34, 25 and 4 taxa in groups A–D; refitting them after the 32 records have also been removed from the range-density denominator gives 24, 34, 23 and 6 taxa, the median change in μ being 0.3 m, with two taxa moving from group C to group D (Anthelia juratzkana, n = 30, and Gymnomitrion concinnatum, n = 12). No single-record taxon belongs to group A. Group D again comprises six taxa after the complete refitting, although not the same six: its four multi-record members are retained, its two single-record members are excluded by construction, and the two taxa named above enter it from group C. Repeating the kernel density analysis on the refitted μ values of the 87 taxa again resolves four modes at every bandwidth from 0.15 to 0.30, with internal minima at 1123–1158 m and 1808–1917 m. The single-record taxa therefore determine neither the number of groups nor the position of the internal minima, although the upper cutpoint is located less precisely without them. Taxon-level values are given in **Supplementary Table 5**.

It should be noted that this division into four groups differs from the three-group classification obtained for the Zov Tigra National Park by Klimova et al. (2024). The emergence of a distinct fourth group in the alpine zone is likely attributable to the greater altitudinal range of the study area on the Kamchatka Peninsula (up to 2075 m a.s.l.) compared with Sikhote-Alin (up to 1839 m a.s.l.), as well as to the more pronounced physiognomic differences between vegetation communities at high elevations.

### 3.4 Correspondence with vegetation belts

An important question is how the statistically derived altitudinal groups correspond to the recognized vegetation belts of the study area. Five vegetation belt-like structures were designated above: BF, CF, TB, PF and AH.

**Figure 10:**
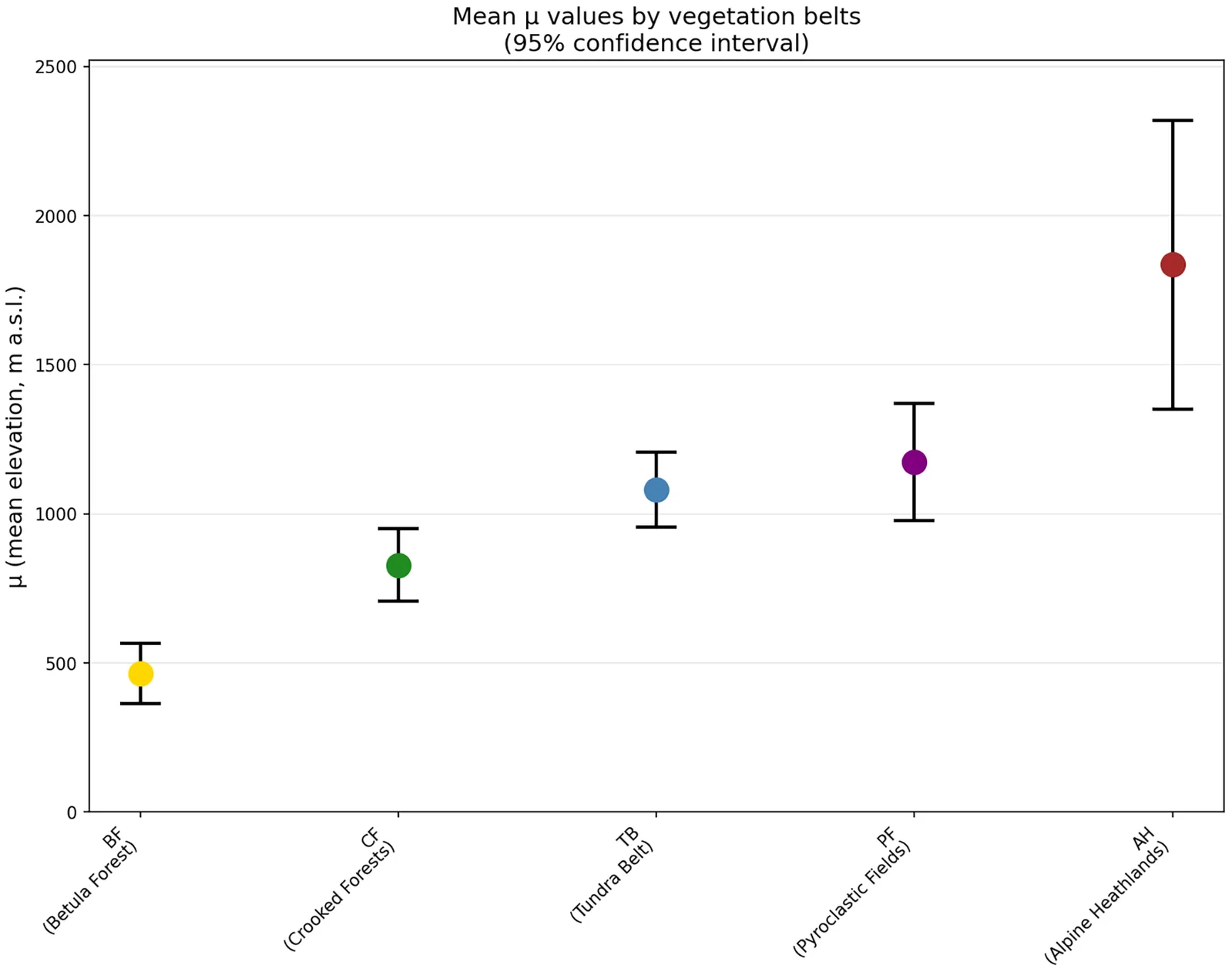
Mean μ by vegetation belt with descriptive 95% intervals. Because the belt floras share taxa, these intervals summarize individual belts and are not used for comparison between belts (Sections 2.4.2, 3.6).

**Figure 11:**
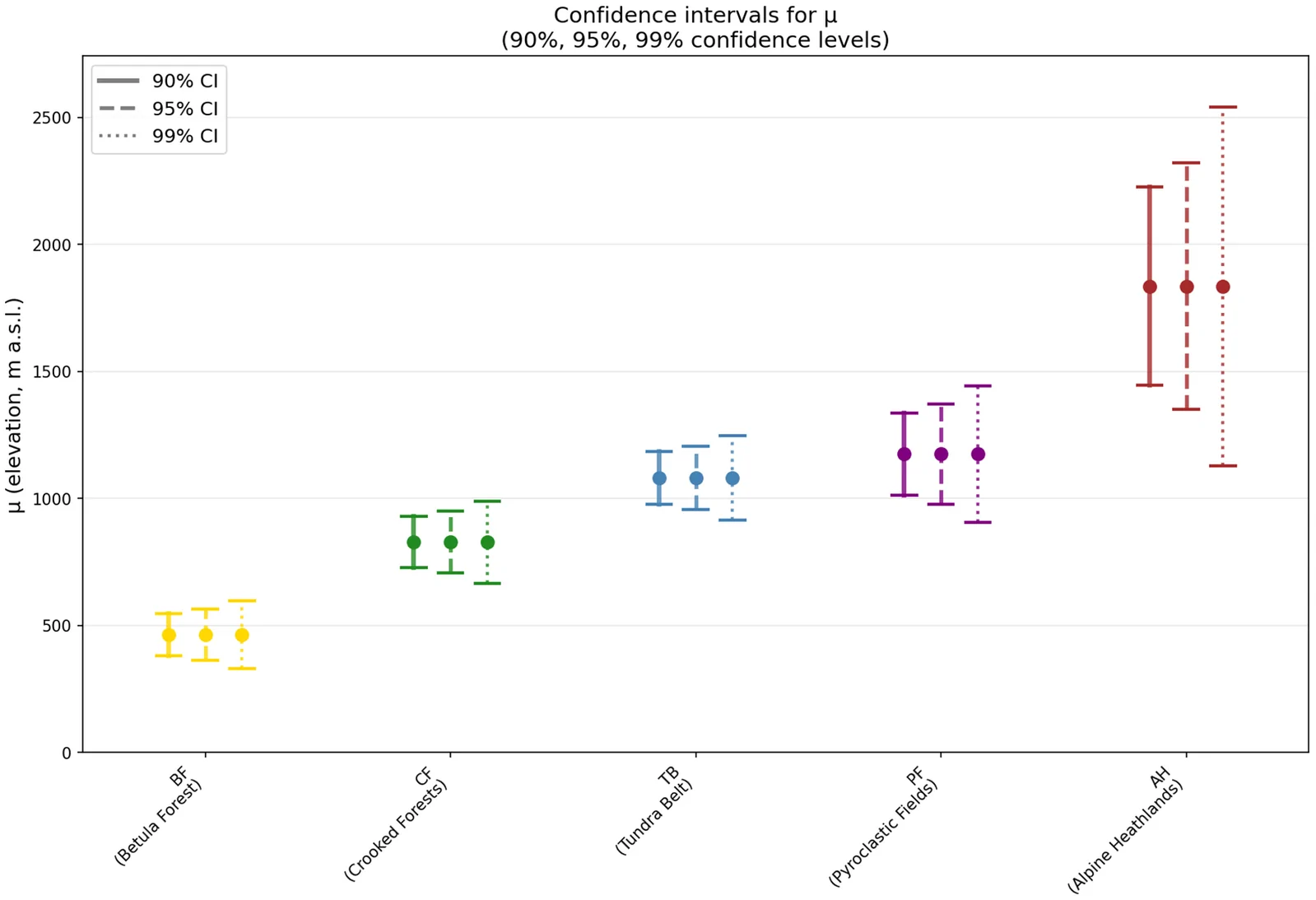
Descriptive intervals for mean μ by belt at the 90%, 95% and 99% levels. Because the belt floras share taxa, these intervals summarize individual belts and are not used for pairwise comparison (Sections 2.4.2, 3.6).


For each vegetation belt, we computed the mean value of μ across all taxa recorded in that belt, together with the standard deviation (SD), which describes the dispersion of taxon-level distribution centers within the belt. Because the same taxon can be recorded in several belts, the belt-level taxon sets overlap and are not independent samples. The 95% intervals calculated from Student’s t-distribution and shown in **Figures 10**; **11** are therefore marginal descriptive summaries of individual belts rather than inferential intervals for comparing them; the belts are compared by means of the dependence-preserving taxon-level bootstrap described in Section 2.4.2.

$$CI\;=\;\left[\bar{\mu }\;\pm \;t_{\alpha /2,\;n-1}\cdot \frac{s}{\sqrt{n}}\right]$$

where $\bar{\mu }$ is the sample mean, s is the sample standard deviation, n is the number of taxa, and t is the critical value of a Student’s t-distribution at the chosen significance level.

BF (Betula Forest): 52 taxa, mean μ = 463 m, SD = 363 m, 95% CI = 362–564 m.

CF (Crooked Forests): 64 taxa, mean μ = 828 m, SD = 487 m, 95% CI = 706–949 m.

TB (Tundra Belt): 66 taxa, mean μ = 1081 m, SD = 509 m, 95% CI = 955–1206 m.

PF (Pyroclastic Fields): 23 taxa, mean μ = 1174 m, SD = 456 m, 95% CI = 977–1371 m.

AH (Alpine Heathlands): 9 taxa, mean μ = 1835 m, SD = 631 m, 95% CI = 1350–2321 m.

The results show a clear altitudinal gradient in the mean distribution centers across vegetation belts. The Betula Forest belt has the lowest mean μ value, reflecting its position at the lower elevations. The Crooked Forests and Tundra Belt occupy intermediate positions, while the Alpine Heathlands have the highest mean μ, as expected from their topographic position.

**Figure 12:**
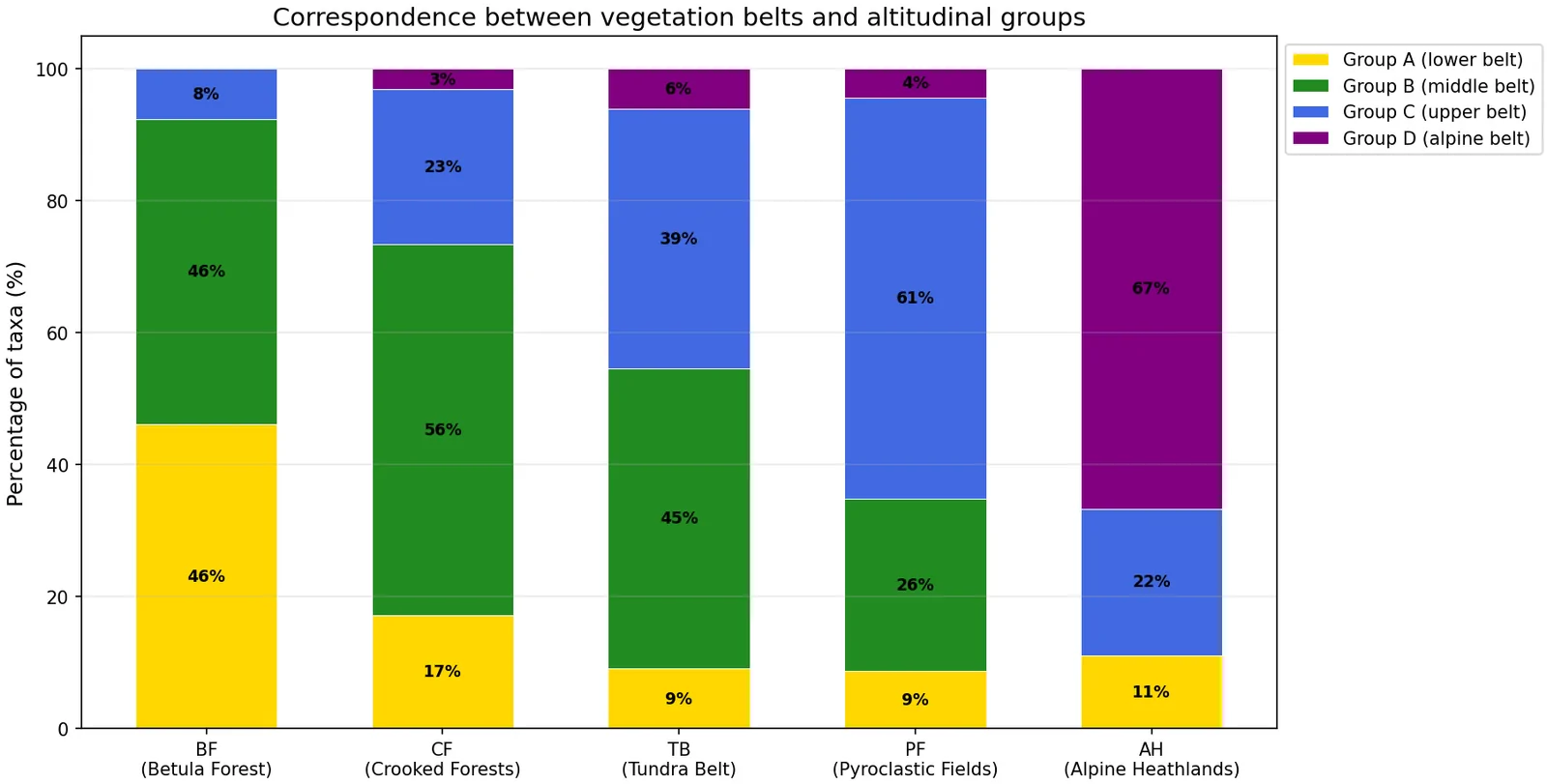
Correspondence between vegetation belts and altitudinal groups.

The correspondence is generally strong but not absolute. The Betula Forest belt is dominated by taxa from groups A and B, consistent with its lower-elevation position. The Crooked Forests show a predominance of group B taxa with a notable contribution from group C. The Tundra Belt has a mixed composition with groups B and C as the most prominent, and Pyroclastic Fields are dominated by group C but retain contributions from groups B and D. The Alpine Heathlands are dominated by group D taxa, with a smaller contribution from group C and one lower-elevation taxon, supporting their high-elevation character while also reflecting the small number of taxa in this belt. The composition of each revealed vegetation belt by elevation groups is shown in **Figure 12**.

### 3.5 Status of pyroclastic fields

Pyroclastic Fields (PF) represent an azonal phenomenon characteristic of the volcanic landscapes of the Kamchatka Peninsula. These areas consist of volcanic scoria, ash deposits, and lava clinkers, where the vegetation cover is sparse and dominated by cryptogams, including liverworts. Despite their distinctive physiognomy, PF do not emerge as a separate elevational unit. They are compared below with the Tundra Belt (TB) and with the Crooked Forests (CF) in three ways: by the mean elevational position of their constituent taxa, by their composition in terms of the altitudinal groups, and by the taxon lists themselves.

The mean μ values for PF and TB are close: 1174 m and 1081 m, respectively, and their descriptive 95% intervals overlap substantially (**Figure 11**). In the taxon-level bootstrap the PF–TB difference is 94 m (95% percentile interval −89 to 265 m; two-sided tail probability 0.29), whereas the PF–CF difference is 346 m (95% percentile interval 167 to 519 m, excluding zero). Because the presence of one difference and the absence of the other do not by themselves compare the two distances, the distance contrast defined in Section 2.4.2 was evaluated directly: |mean μ(PF) - mean μ(CF)| exceeds |mean μ(PF) - mean μ(TB)| by 253 m (95% percentile interval 90 to 354 m), with 0.6% of replicates at or below zero. The mean elevational position of PF is thus closer to that of TB than to that of CF as a direct positive result, not as an inference from the pattern of interval coverage.

The same conclusion follows from the composition of the belt floras in terms of the altitudinal groups. Group C accounts for 60.9% of the PF flora, against 39.4% in TB and 23.4% in CF, while the share of group A is almost identical in PF and TB (8.7% and 9.1%) and about twice as high in CF (17.2%). The L1 distance between the group-composition profiles of PF and TB is 0.43, against 0.77 between PF and CF and 1.15 between PF and BF. In the taxon-level bootstrap the contrast L1(PF, CF) − L1(PF, TB) equals 0.34 with a 95% percentile interval of 0.09 to 0.52; the interval excludes zero, and only 0.6% of the replicates reverse its sign, so the profile of PF is closer to that of TB than to that of CF by more than resampling variation can account for. In terms of altitudinal-group composition the pyroclastic fields thus follow the tundra belt and not the crooked forests (**Figure 12**).

Direct comparison of the taxon lists (**Table 2**) is less discriminating, for the reason given in Section 2.4.2. The highest values in the matrix are those of the CF–TB pair (J = 0.461; S = 0.631), which reflects both the floristic continuity of the two upper belts and their comparable sizes. PF shares 14 of its 23 taxa with CF (J = 0.192; S = 0.322) and 13 with TB (J = 0.171; S = 0.292), that is 60.9% and 56.5% of the PF flora respectively; the difference of a single taxon carries no statistical weight (exact binomial test on the discordant taxa, p = 1.00). Moreover, 11 of the 13 taxa shared with TB are shared with CF as well, so that the two intersections are largely the same set of taxa, and 16 of the 23 PF taxa (69.6%) belong to the combined CF–TB pool. The symmetric indices therefore place PF inside the connected upper-elevation complex without discriminating between its two components.

**Table 2:** Pairwise compositional similarity of the vegetation-belt floras.

Belt	BF	CF	TB	PF
BF	—	0.466	0.339	0.213
CF	0.303	—	0.631	0.322
TB	0.204	0.461	—	0.292
PF	0.119	0.192	0.171	—

Sørensen indices are given above the diagonal and Jaccard indices below it; AH is excluded because only nine taxa were recorded in it. Flora sizes: BF 52, CF 64, TB 66 and PF 23 taxa. Numbers of shared taxa: BF–CF 27, BF–TB 20, BF–PF 8, CF–TB 41, CF–PF 14 and TB–PF 13.

This conclusion has important phytogeographical implications. While PF is easily distinguishable in the field as a distinct landscape element — areas of bare black volcanic rocks and fine deposits with scattered pioneer vegetation — its liverwort flora belongs to the same elevational domain as that of the mountain tundra. The apparent distinctiveness of PF is therefore a substrate-driven physiognomic phenomenon rather than a reflection of genuinely different altitudinal or climatic conditions. This does not mean that the two floras are identical. Seven of the 23 PF taxa — Cephaloziella varians, Endogemma caespiticia, Gymnomitrion alpinum, both varieties of Schljakovianthus quadrilobus, Solenostoma rossicum and Tetralophozia setiformis — were recorded nowhere else in the park, and all of them belong to group C, with μ between 1289 and 1431 m. The pyroclastic fields are thus a substrate-filtered assemblage within the tundra elevational domain: the substrate selects a particular set of taxa, but it does not shift the assemblage to a different position along the elevational gradient.

### 3.6 Confidence intervals

To evaluate uncertainty in belt-level mean μ, we constructed confidence intervals at three confidence levels: 90%, 95%, and 99% (**Figure 11**).

The descriptive 95% intervals separate the lower forest belt from the crooked forest belt (BF: 362–564 m; CF: 706–949 m), while the intervals of CF and TB approach one another, in agreement with a gradual transition from elfin woodland to alpine tundra, and the intervals of TB and PF overlap substantially. Because these intervals cannot be compared with each other directly, the belts were also compared by the taxon-level bootstrap (**Table 3**). The bootstrap supports the ordered elevational shift BF → CF → TB → AH, all three successive differences excluding zero (CF − BF = 364 m, 241–495 m; TB − CF = 253 m, 150–368 m; AH − TB = 755 m, 279–1083 m). PF is displaced above CF (346 m, 167–519 m) and lies below AH (AH − PF = 661 m, 156–1024 m), whereas the PF − TB difference (94 m, −89 to 265 m) includes zero; the direct distance contrast of Section 3.5 places the mean elevational position of PF closer to that of TB than to that of CF (difference in distances 253 m, 95% interval 90–354 m). The ordering of the vegetation belts along the elevational gradient is therefore not an artefact of the taxa shared between them, and the pyroclastic fields occupy the same elevational domain as the tundra belt.

**Table 3:** Differences between belt-level mean μ, estimated by a taxon-level bootstrap of 10,000 replicates in which each resampled taxon retains all of its vegetation-belt memberships (Section 2.4.2).

Comparison	Difference of means (m)	95% bootstrap interval (m)	Two-sided tail probability
CF − BF	364	241 to 495	< 0.001
TB − CF	253	150 to 368	< 0.001
PF − CF	346	167 to 519	< 0.001
PF − TB	94	−89 to 265	0.29
AH − PF	661	156 to 1024	0.018
AH − TB	755	279 to 1083	0.006

Positive values indicate that the first belt lies higher.

**Table 4:** Effect of the isolation diagnostic: filtered versus unfiltered range-density fits for the ten taxa from which isolated records were removed.

Taxon	n	n used	Excluded elevations (m)	μ filtered (m)	μ unfiltered (m)	Group
Aneura pinguis	11	8	1261, 1280, 1280	180	180	A/A
Cephaloziella divaricata	12	11	2075	180	2125	A/D
Cryptocoleopsis imbricata	16	15	597	1477	1476	C/C
Diplophyllum albicans	7	6	573	1831	1831	C/C
Gymnocolea inflata	8	6	420, 573	1424	1423	C/C
Marsupella boeckii	3	2	1256	571	879	B/B
Marsupella tubulosa	5	4	1280	180	180	A/A
Nardia harae	5	4	1457	573	577	B/B
Nardia insecta	6	5	1457	417	416	B/B
Sphenolobus minutus	8	7	471	1585	1585	C/C

Aggregate group sizes are 24, 54, 35 and 6 taxa in groups A–D with filtering and 23, 54, 35 and 7 without it.

## 4 Discussion

### 4.1 Floristic overview

The flora of the studied area exists under the strong influence of volcanic activity, evidenced by the ‘looseness’ and high drainage capacity of all fine-grained pyroclastic-based soils, as well as the periodic destruction of ground habitats by ashfalls. Indeed, in the Avacha-Koryaksky group of volcanoes, 217 explosive eruptions have occurred in the last 13,500 years alone, covering the surface with ash of varying thickness (Krasheninnikov et al., 2020). The Zhupanovskaya Sopka volcano is less active, but even here at least 350 km^3^ have been erupted since the Upper Pleistocene (http://repo.kscnet.ru/1180/1/Zhupan.pdf). The last notable explosive eruption of this volcano occurred in 2014. In addition to ashfalls, a special feature of the landscapes of Nalychevo Park is various thermal outputs in the form of fumaroles, solfataras and various thermal springs. Often, the activity of thermal springs leads to the formation of travertine shields, sometimes of great thickness and extent. For example, the large swamp in the valley of the Levaya Nalycheva River is entirely developed on an old giant travertine shield. This and similar swamps provide opportunities for basiphilous species to grow in the study area (realized at least by *Mesoptychia rutheana* (Limpr.) L. Söderstr. & Váňa). Small sedge-moss mires, developed near thermal springs and the banks of thermal streams, are also a special type of habitat, leading to the appearance here of either facultative thermophiles (such as *Calypogeia kamchatica*) or species that, in principle, prefer habitats rich in sulfur (*Metasolenostoma orientale* Bakalin et Vilnet).

Among the 35 species newly recorded in the park, many were found at several localities. Moreover, at each site, they were found often in several habitat types, so that their substrate limitation does not seem clearly expressed. At the highest point, the Dzendzur volcano crater crown (2075 m above sea level), only four species were collected, three of which, however, were new to the park: Gymnomitrion concinnatum (Lightf.) Corda (found in the park at several other localities), *Lophoziopsis polaris* (R.M. Schust.) Konstant. & Vilnet. (found only there), and *Scapania degenii* Schiffn. ex Müll.Frib. (found only there). It should be noted that of the 35 new species, 22 were found (only, or including) in the *Betula ermanii* forests and in the crooked forest belts, which may suggest insufficient attention to these belts in the course pf previous explorations.

Of the previously known species, 24 were not collected during the 2022 survey (plus *Radula prolifera*, the 25th taxon that was excluded from the flora as misidentified). In some cases, this can be explained by insufficiently careful searching, but probably not in all cases. In some cases, one of the authors (Bakalin) clearly remembered the places where certain species were collected in 2015, but these species were not found when the same sites were revisited in 2022. For instance, *Cephaloziella varians* (Gottsche) Steph. and *Schljakovianthus quadrilobus* (Lindb.) Konstant. & Vilnet occupied vast areas on the moist gentle slopes below the Dzendzur fumarole in 2015. However, in 2022, we were unable to find these habitats there at all. They may have been destroyed during the constant movements of weakly fixed, waterlogged ash deposits, which cover all the gentle slopes in the alpine belt of the park. On the other hand, such almost ‘inevitable’ components of the Northeast Asian hemiarctic as *Gymnomitrion concinnatum* and *Marsupella apiculata* Schiffn., which were not found despite special searches in 2015 and 2001, were unexpectedly discovered (each from several localities) during the survey in 2022. Despite precisely measured coordinates, *Frullania subarctica* Vilnet, Borovichev & Bakalin, which occupied a significant area on the rocks near the Pinachevsky Pass in 2015, was not found again at the same place. We are inclined to explain the surprising rarity of species such as *Tetralophozia setiformis* (Ehrh.) Schljakov, *Schljakovia kunzeana* (Huebener) Konstant. & Vilnet (both discovered for the first time, and each from only one locality), by volcanic activity (primarily disturbances of the ground cover as a result of ashfalls). Thus, we assume that the area occupied by the vast majority of the taxa is extremely flexible over time, and taxa that may seem common during studies in one year may not be found in a few years later. Species that would be tied to some ‘typically volcanic’ habitats are generally few in the world’s floras. In the flora of the Nalychevo Nature Park, there is only one such species — *Cryptocoleopsis imbricata* Amakawa, inhabiting ash deposits or andesite rocks only in areas of active volcanism in the Kamchatka-Japanese macro-region along a chain of active volcanoes.

One of the most interesting records is *Gymnomitrion adustum* Nees, which was previously known from the Kuril Islands and South Kamchatka (Bakalin et al., 2022b) in Russian Asia. The presented locality is the first in East Kamchatka and, apparently, the northernmost in Pacific Asia. The distribution pattern of this species may be described as an oro-boreal in Pacific Asia, although in Europe it is rather an alpine-subalpine species (Paton, 1999). The issue of the identity of the Pacific and European populations, currently attributed to the same species, remains unresolved. Examination of the lectotype of the species (STR, s.n.) did not reveal significant differences in morphology between it and the amphi-Pacific plants, which, however, does not confirm the genetic identity of the materials.

Regarding the problem of the identity of plants from the *locus classicus* and material from East Kamchatka, another such example was *Jungermannia pumila* L. Throughout its species range, this taxon is characterized by non-biconcentric oil bodies, whereas in Pacific Asia the plants have biconcentric oil bodies in leaf cells at least in the vast majority of populations. The current taxonomic status of these populations has shown that this is a genetically well-distinct subspecies (subsp. *orientale*) (Konstantinova et al., 2025). *Nardia harae* Amakawa and *Gymnocolea marginata* (Steph.) S. Hatt. should also be mentioned among the species with dubious status. We previously expressed similar doubts when describing liverworts from South Kamchatka (Bakalin et al., 2022b), and these issues have not yet been resolved. Given the general trend toward the adoption of narrower ranges (and, accordingly, species splitting) in a number of bryophyte groups, these species (or morphological variants, which is not clear) at least deserve particular attention until these issues are resolved on larger datasets.

The conducted studies (both reported now and done before) may inspire some ideas on the most important sites for the liverwort protection inside the park. First, this is a complex of thermal swamps and a stream in the area of ​​the Kraevedcheskie hot springs (locality number 3 on **Supplementary Table 1**, the same springs are often called Talovskie). This place is the original locality for *Calypogeia kamchatica* (Bakalin et al., 2022a), as well as a distribution site of the rare Kamchatka-Kuril (distribution in Japan is not obvious) *Metasolenostoma orientale* (Bakalin et al., 2014). This place is under severe anthropogenic impact since it is located near the forestry house in an area often visited by tourists in the immediate vicinity of a thermal pool, widely used for recreational purposes.

Second, this is a large swamp massif on a travertine shield in the area of ​​the central estate (locality 1 in **Supplementary Table 1**). This is the only known locality in Kamchatka for Aneura maxima, as well as the Kamchatka-rare *Moerckia flotoviana* (Nees) Schiffn, *Mesoptychia rutheana* and some other species. Since on one side of the massif there is a complex of buildings belonging to the central estate of the park (including accommodation for tourists), and on the other (eastern) side there is a terminus used by heavy off-road vehicles, there is substantial movement of people and goods through the swamp, leading to damage to the vegetation cover and degradation of the peat deposit. In this area, it is necessary to construct a permanent trail with a boardwalk.

Third, this is a small swamp massif, apparently receiving inflowing water with a neutral or alkaline reaction, near the upper tourist house in the upper reaches of the Pinacheva River (locality 24 in **Supplementary Table 1**). This is the habitat of *Riccardia chamedryfolia* (With.) Grolle, *Saccobasis polita* (Nees) H.Buch, which are rare in Kamchatka, and the only location of *Schljakovia kunzeana* in the park. The tourist trail here goes right through the swamp, since movement on other sides is hampered by thickets of shrubby Salix. Due to the latter, the cover is heavily disturbed, gullies form and degradation of the peat deposit occurs. If actions are not taken in the near future (for example, through the construction of a permanent boardwalk with railings), this valuable habitat may be completely destroyed.

Taken together, the three localities can be described within the Pressure–State–Response scheme, applied here qualitatively as a way of translating local threats into management actions; the same scheme has recently been combined with multi-criteria weighting for regional conservation prioritisation by Karadeniz et al. (2026). The pressures are tourist access and trampling at all three sites and, at the central estate mire, the passage of heavy off-road vehicles. The state indicators are damage to the liverwort cover, gully formation and degradation of the peat deposit; the conservation value of the sites derives from the presence of taxa known in the park from a single locality, such as Aneura maxima, Schljakovia kunzeana and Metasolenostoma orientale. The corresponding responses are the construction of boardwalks at the two mire sites, with railings in the upper reaches of the Pinacheva River where the trail crosses the mire and cannot be diverted because of dense Salix thickets, the regulation of visitor access around the Kraevedcheskie thermal pool, and repeated monitoring of the condition of the cover. Of the three localities the upper Pinacheva mire is the most urgent, since erosion of the peat deposit is already in progress and continued degradation may destroy the habitat entirely. We use the scheme descriptively and do not reproduce the multi-criteria weighting procedure of Karadeniz et al. (2026), which would require a separate dataset.

The identified total of 118 species and one subspecies is quite consistent with our assumptions made in the previous work (Bakalin and Klimova, 2018), but is still not exhaustive. Considering that during the 2022 exploration 35 species were identified for the park for the first time, the situation with the state of knowledge has not yet reached a plateau in the knowledge curve. We expect an increase in the list composition by at least another 20–30 species in the course of studying both 1) the lower elevational belts (especially in the middle and lower reaches of the Nalycheva River), where both forest taxa and a number of species from oligotrophic bog complexes can be identified, and 2) the upper limits — alpine wastelands on the Koryak-Avachinskaya and Zhupanovskaya groups of volcanoes.

### 4.2 Individual species spectra from the Range–Density (RD) MLE

Regardless of the number of occurrence records known for a particular taxon, the method places all taxa in a common elevational parameter space. Estimates based on very few records, especially single records, should be interpreted as low-confidence constraints rather than full reconstructions of the taxon’s distribution; excluding all 32 single-record taxa and refitting the remaining flora nevertheless leaves all four groups occupied (Section 3.3). The range-density correction normalizes the records of a taxon by the local density of all occurrence records, so that μ and σ describe the observed altitudinal concentration of records after correction for the uneven density of the record set. The isolation diagnostic introduced in Section 2.4.1.3 provides an additional safeguard against single outliers driving the inferred center of distribution.

The single Gaussian response is used deliberately as a common descriptor that places every taxon, irrespective of the number of records available for it, within one elevational parameter space; it is not a claim that each taxon has exactly one symmetric ecological optimum. Across the flora it performs well (median R^2^ = 0.81; Section 3.2.1), and the fit diagnostics identify the exceptions that require individual interpretation. Microhabitat dependence can indeed generate asymmetric or locally bimodal empirical curves. In the study area, however, the two situations that would most obviously produce a second peak do not divide a taxon between two elevational types. Mires here are intrazonal and grade directly into mountain-tundra habitats, so that a taxon occupying both is occupying one continuous ecological setting rather than two distant ones; and montane taxa may descend along road margins, stream valleys and other disturbed corridors far below the belt with which they are normally associated. Occurrences of this kind broaden or skew the response curve without relocating the elevational affinity of the taxon. This is also what the data show: of the ten taxa in which the isolation diagnostic detected a second, observationally disconnected segment of records — the closest observable counterpart of a second elevational peak in records of this density — only one changed its altitudinal group when those records were restored (Section 4.6).

### 4.3 Distribution of taxa among altitudinal groups

From a phytogeographical point of view the four altitudinal groups delineated in Section 3 are ecologically coherent. Group A (n = 24, μ < 300 m) is dominated by boreal taxa that are common in floodplain and lower-slope habitats. Group B (n = 54, 300–1100 m) is the most diverse and mixes boreal and hemiarctic elements typical of the Betula ermanii belt. Group C (n = 35, 1100–1900 m) encompasses generally hemiarctic and arctic-montane taxa, while Group D (n = 6, μ > 1900 m) is restricted to the alpine heathlands near and above the vegetation limit. The boundaries between the groups correspond to minima of the μ distribution that remain identifiable over a broad, although not unlimited, range of histogram resolutions and bin origins and are reproduced by bin-free kernel density estimation (Section 3.3); support is strongest for the B/C boundary, whereas the C/D minimum merges at the coarsest resolutions. The four groups are therefore not an arbitrary partition of a continuum but reflect ecological discontinuities associated with elevational discontinuities, even though the numerical value of each cutpoint is defined only to within about one bin width.

Groups A and D differ from groups B and C in one further respect that is worth stating explicitly. Both are boundary groups: their fitted μ values accumulate at the limits of the evaluated interval, indicating an affinity for the ends of the sampled profile whose exact centre the data cannot locate (Section 2.4.1.2). Boundary-limited fits are the situation for which the original formulation of the method retained wide parameter bounds (Klimova et al., 2024) rather than a defect of the approach, and the pattern is ecologically consistent with the truncated lower part of the transect, which does not reach the middle and lower course of the Nalycheva River, and with the restricted extent of the habitats at its upper end. For these two groups the direction of the ecological affinity is well determined and the group membership is stable, whereas the exact position of the distribution centre is not resolved by the present data.

### 4.4 Altitudinal groups versus floras of vegetation belts

The statistically derived altitudinal groups and the classical floras-by-belt are not equivalent: an individual taxon with μ in group B can nonetheless occur in BF, CF, or TB, because belt-by-belt floras include every taxon found in a given belt, irrespective of where its centre of distribution lies. The altitudinal group, by contrast, is a property of the taxon itself, not of the belt. This conceptual difference explains why group counts and belt species counts do not coincide and why a comparison between the two provides complementary information about the structure of the flora. This distinction also determines the statistical treatment of the belt means. Because belt floras are overlapping sets of taxa, the intervals shown in **Figures 10** and **11** are used descriptively, and the comparisons between belts rest on the taxon-level bootstrap, in which every resampled taxon carries all of its belt memberships (Sections 2.4.2 and 3.6).

An important consequence of the analysis is the demonstration that Pyroclastic Fields (PF) do not constitute a distinct altitudinal zone in terms of the elevational affinities of their liverwort flora. Although PF are physiognomically conspicuous and occupy a specific altitudinal band, the direct bootstrap distance contrast places the mean elevational position of their taxa closer to that of the surrounding Tundra Belt than to that of the Crooked Forests (difference in distances 253 m, 95% interval 90–354 m), and the group-level composition of PF is closer to that of TB than to that of CF by more than resampling variation can account for (Section 3.5; **Figures 10**, **11**; **Supplementary Table 4**). PF should therefore be regarded as a substrate-driven facies of the tundra belt rather than an independent vegetation belt sensu stricto. The direct comparison of taxon lists (**Supplementary Table 5**) is consistent with this, but does not by itself discriminate between the two upper belts, because the symmetric similarity indices are strongly constrained by the small size of the PF flora: PF shares 60.9% of its taxa with CF and 56.5% with TB, a difference of one taxon, and most of the taxa involved are shared with both. What the taxon lists do add is that the PF assemblage is not simply a subset of the tundra flora, since seven of its 23 taxa, all with μ between 1289 and 1431 m, were found nowhere else in the park. This substrate filtering accounts for the compositional distinctiveness of PF without displacing it along the elevational gradient.

The Alpine Heathlands (AH) are separated in mean elevational position both from the tundra belt (755 m, 95% interval 279–1083 m) and from the pyroclastic fields (661 m, 156–1024 m) in the taxon-level bootstrap. AH is dominated by Group D taxa, and these taxa occupy the highest part of the μ–σ plane (**Figure 8**), with high distribution centres and relatively narrow altitudinal widths. Thus, AH is a small but well-defined high-elevation assemblage, supported by the concentration of Group D taxa. Group D itself is likewise supported rather than merely suspected: it remains a six-taxon group when the single-record taxa are removed and the model is refitted — its four multi-record members persist and two well-sampled taxa join it from group C — and two of its members are represented by 19 and 12 occurrence records. According to field observations, the corresponding habitats are spatially restricted and absent from much of the park; since the survey covered the elevational gradient up to 2075 m a.s.l., the small size of the group is interpreted as reflecting habitat availability at least as much as collecting intensity. What remains poorly resolved is not the existence of the upper component but the exact position of its centre, because five of the six group D fits are boundary-limited at the upper end of the evaluated interval, so that the data constrain the direction of the affinity of these taxa but not the position of their centres.

### 4.5 Comparison with previous results

When comparing the present results for the Kamchatka Peninsula with those obtained for the Zov Tigra National Park in Sikhote-Alin (Klimova et al., 2024), several noteworthy differences emerge. First, the division into altitudinal groups is more pronounced: four groups are clearly distinguishable compared with three in Sikhote-Alin. This phenomenon is difficult to explain; it may be related to the zonal position of the studied region, where such shifts occur more abruptly. Of particular importance is the observation that the greater portion of the elevational transect in the study area lies above the timber line, whereas in Zov Tigra National Park only a narrow, weakly differentiated elevational belt, caused by mesoclimatic fluctuations, exists above the tree-line. This assumption finds indirect support in the fact that avian species composition in the Andes undergoes a 91% turnover above the forest zone (Pineda-López et al., 2020).

### 4.6 Significance and applicability of the method

The principal value of the occurrence-record adaptation of the range-density (RD) MLE method, together with the isolation diagnostic introduced here, is that it allows one to distinguish between a random admixture of taxa produced by substrate preferences or chance collecting events and consistently observed altitudinal concentrations. Without such a method, any attempt to describe a taxon’s typical altitudinal position from raw collection records will be biased by the uneven density of the occurrence records themselves — an especially acute problem in volcanic landscapes, where in the present dataset the local record density n(x) falls from 558 near 690 m a.s.l. to 13 between 1800 and 1980 m and to 6–9 above 2000 m, that is by almost two orders of magnitude between a trail-accessible valley and a high summit. By normalizing taxon records by occurrence-record density and, where necessary, excluding observationally disconnected records from the core fit, the method yields μ and σ estimates that reflect the observed altitudinal structure of the dataset rather than the geography of past expeditions alone. The comparison of filtered and unfiltered fits shows how far this correction and the accompanying diagnostic actually reach (**Supplementary Table 6**). Restoring the isolated records to the ten taxa from which they had been removed changes the aggregate group sizes from 24, 54, 35 and 6 to 23, 54, 35 and 7 taxa, and alters the assignment of exactly one taxon out of 119: Cephaloziella divaricata, whose single record at 2075 m, separated from the rest of its range by a gap of 1434 m, moves the fitted centre from 180 m to 2125 m and the taxon from group A to group D. Eight of the ten taxa shift by less than 5 m, the median shift being 0.6 m. The overall structure is thus robust to the diagnostic, while individual taxa can be affected decisively. The records removed from a core fit are not thereby declared erroneous: they remain in the dataset, are plotted in **Supplementary Online Appendix 2**, and may represent genuine disjunct occurrences deserving taxonomic and field verification rather than automatic rejection.

It is worth noting that the qualitative phenomenon addressed here — the stratification of the liverwort flora into altitudinally distinct groups — was already recognized in the early nineteenth century. Hübener’s Hepaticologia Germanica (Hübener, 1838) describes altitudinal differences in Central European liverwort assemblages in vivid qualitative terms, but without a means of quantitatively separating genuine elevational preference from the geography of collecting. The present approach brings that long-standing qualitative observation into a quantitative framework: Hübener’s intuitive altitudinal “belts” in Europe are consonant with the statistically defined groups A–D in East Kamchatka, and his specific high-alpine finds correspond to the records now flagged by the isolation diagnostic. In this sense, the method described here gives a formal, reproducible expression to an ecological pattern that was informally recognized almost two centuries ago.

## 5 Conclusions

First, the range-density (RD) MLE framework, originally developed for southern Sikhote-Alin (Klimova et al., 2024), was adapted here from binary locality-level presence/absence data to occurrence-record frequencies and yielded four clearly interpretable altitudinal fractions, including a lower and an upper boundary group. Sensitivity analyses support this structure over a broad range of resolutions: the trough at 1100 m is recovered in 17 of the 18 histogram configurations, bracketed by two adjacent minima in the remaining one, and by kernel density estimation at bandwidth factors 0.15–0.30, the trough at 1900 m corresponds to an empty interval of the μ series (1890–1993 m) and is resolved at fine and moderate resolutions, and removal of the 32 single-record taxa followed by complete refitting leaves all four groups occupied and reproduces the four-mode structure. The exact cutpoint values are defined to within about one bin width. Second, the introduction of a *post-hoc* isolation diagnostic with a threshold of 2d (one full RD-MLE window width) allows 13 taxa with observationally disconnected records (potential extreme occurrences or taxonomic ambiguities) to be flagged without any additional tunable parameters; in 10 of these taxa, isolated records are excluded from the core fit, whereas three two-record cases remain unresolved and retain both records. Third, a dependence-preserving taxon-level bootstrap, which does not treat taxa shared between belts as independent observations, supports the ordered elevational shift from Betula forests through crooked forests and tundra to alpine heathlands, and, through the direct distance contrast, shows that the mean elevational position of the Pyroclastic Fields is closer to that of the Tundra Belt than to that of the Crooked Forests (253 m, 95% interval 90–354 m); together with the closeness of their altitudinal-group composition to that of the tundra, this identifies PF as a substrate-filtered facies of the tundra elevational domain rather than an independent belt, distinctive in composition, since seven of its 23 taxa occur nowhere else in the park, but not in elevational position. Alpine Heathlands and Group D form a small high-elevation component that is separated from the tundra in mean elevational position and survives the removal of all single-record taxa, its small size being consistent with the restricted extent of suitable habitats documented by field observation. Fourth, a comparison with the Sikhote-Alin dataset (Klimova et al., 2024) shows a sharper separation of altitudinal groups in Kamchatka, which is consistent with the stronger environmental gradient imposed by volcanism and the altitudinally truncated lower floristic belts. Finally, the combined RD-MLE and isolation-diagnostic framework provides a reproducible quantitative estimator of the dominant elevational affinity of a taxon under a strongly uneven density of occurrence records, together with an explicit diagnostic for observationally disconnected records; its operational group cutpoints remain resolution-dependent, whereas the number of groups and the approximate position of the internal minima are stable over a broad range of resolutions. The framework is applicable to other mountain systems and to other groups of cryptogams.

## Data Availability

The original contributions presented in the study are included in the article/**Supplementary Material**. Further inquiries can be directed to the corresponding authors.

## Supplementary material

Supplementary Figure 1 Sensitivity of the altitudinal grouping to the resolution of the μ histogram. **(A)** The histogram at the adopted 200-m resolution, overlaid with bin-free kernel density estimates at three bandwidths. **(B)** The same μ values at six bin widths.

Supplementary Table 1 The list of collecting localities from the 2022 expedition.

Supplementary Table 2 The checklist of liverwort flora in the Nalychevo Nature Park. The species name filled with yellow color is new report for Kamchatka, the species names filled with green color are new records for the park.

Supplementary Table 3 The complete results of the RD-MLE analysis for all 119 taxa, including the diagnostic isolation status for each taxon.

Supplementary Table 4 Sensitivity of the μ histogram: interior local minima for six bin widths and three bin origins, and the modes and minima of the bin-free kernel density estimate.

Supplementary Table 5 Taxon-level results after exclusion of the 32 taxa represented by a single occurrence record: n, μ and altitudinal-group assignment for the 87 retained taxa, both for the retabulation under the primary estimates and for the complete refitting after removal of the 32 records from the range-density denominator.

Supplementary Table 6 Coefficient of determination R^2^ between the empirical range-density function and the fitted Gaussian response, for all 87 taxa with two or more occurrence records.

## References

[B1] Ah-Peng C. Chuah-Petiot M. Descamps-Julien B. Bardat J. Stamenoff P. Strasberg D. (2007). Bryophyte diversity and distribution along an altitudinal gradient on a lava flow in La Réunion. Divers. Distrib. 13, 654–662. doi: 10.1111/j.1472-4642.2007.00393.x40046247

[B2] Ah-Peng C. Wilding N. Kluge J. Descamps-Julien B. Bardat J. Chuah-Petiot M. . (2012). Bryophyte diversity and range size distribution along two altitudinal gradients: continent vs. island. Acta Oecol. 42, 58–65. doi: 10.1016/j.actao.2012.04.01042574925

[B3] Austin M. P. (2002). Spatial prediction of species distribution: an interface between ecological theory and statistical modelling. Ecol. Modell. 157, 101–118. doi: 10.1016/S0304-3800(02)00205-3

[B4] Bączkiewicz A. Szczecińska M. Sawicki J. Stebel A. Buczkowska K. (2017). DNA barcoding, ecology and geography of the cryptic species of Aneura pinguis and their relationships with Aneura maxima and Aneura mirabilis (Metzgeriales, Marchantiophyta). PloS One 12, e0188837. doi: 10.1371/journal.pone.0188837 29206876 PMC5716573

[B5] Bahuguna Y. M. Gairola S. Uniyal P. L. Bhatt A. B. (2015). Moss flora of kedarnath wildlife sanctuary (KWLS), garhwal himalaya, India. Proc. Natl. Acad. Sci. India Sect. B Biol. Sci. 86, 931–943. doi: 10.1007/s40011-015-0531-z30311153

[B6] Bakalin V. A. (2009). “ Flora and phytogeography of liverworts of Kamchatka and adjacent islands,” in Flora and Phytogeography of Liverworts of Kamchatka and Adjacent Islands ( KMK Scientific Press, Moscow).

[B7] Bakalin V. A. (2013). Hepatic diversity patterns in the Russian Far East. Bot. Pac. 2, 35–42. doi: 10.17581/bp.2013.02104

[B8] Bakalin V. A. (2026). A contribution to the liverwort flora of Kamchatka (Northwest Pacific). Novosti Sist. Nizsh. Rast. 60, 1–10. doi: 10.31111/nsnr/2026.60.1.b137078554

[B9] Bakalin V. A. Klimova K. G. (2018). “ Flora pechenochnikov (Hepaticae) prirodnogo parka “Nalychevo” (poluostrov Kamchatka),” in Komarovskie Chteniya (Vladivostok), 65, 29–54.

[B10] Bakalin V. A. Fedosov V. E. Maltseva Y. D. Milyutina I. A. Klimova K. G. Nguyen H. M. . (2020b). Overview of Schistochilopsis (Hepaticae) in Pacific Asia with the description Protochilopsis gen. nov. Plants 9, 850. doi: 10.3390/plants9070850 32640627 PMC7412342

[B11] Bakalin V. A. Klimova K. G. (2020). A review of Radulaceae (Marchantiophyta) in the Russian far east. Bot. Pac. 9, 133–153. doi: 10.17581/bp.2020.09204

[B12] Bakalin V. A. Klimova K. G. Karpov E. A. Bakalin D. A. Choi S. S. (2022b). Liverworts of the South Kamchatka Nature Park: survival in active volcanism land. Diversity 14, 722. doi: 10.3390/d1409072230654563

[B13] Bakalin V. A. Maltseva Y. D. Müller F. Klimova K. G. Nguyen V. S. Choi S. S. . (2022a). Calypogeia (Calypogeiaceae, Marchantiophyta) in Pacific Asia: updates from molecular revision with particular attention to the genus in North IndoChina. Plants 11, 983. doi: 10.3390/plants11070983 35406962 PMC9002642

[B14] Bakalin V. A. Vilnet A. A. Choi S. S. Nguyen V. S. (2020a). Blepharostoma trichophyllum s.l. (Marchantiophyta): the complex of sibling species and hybrids. Plants 9, 1423. doi: 10.3390/plants9111423 33114166 PMC7716226

[B15] Bakalin V. A. Vilnet A. A. Furuki T. Katagiri T. (2014). Taxonomic novelties in Solenostoma-Plectocolea complex (Solenostomataceae, Hepaticae) in East Asia. Bot. Pac. 3, 3–18. doi: 10.17581/bp.2014.03201

[B16] Begon M. Townsend C. R. Harper J. L. (2006). Ecology: From Individuals to Ecosystems. 4th Edn (Oxford: Blackwell Publishing), 752.

[B17] Casella G. Berger R. L. (2002). Statistical Inference. 2nd Edn (Pacific Grove, CA: Duxbury), 660.

[B18] Chen Y. Niu S. Li P. Jia H. Wang H. Ye Y. . (2017). Stand structure and substrate diversity as two major drivers for bryophyte distribution in a temperate montane ecosystem. Front. Plant Sci. 8, 874. doi: 10.3389/fpls.2017.00874 28603535 PMC5445162

[B19] Coelho M. C. M. Gabriel R. Hespanhol H. Borges P. A. V. Ah-Peng C. (2021). Bryophyte diversity along an elevational gradient on Pico Island (Azores, Portugal). Diversity 13, 162. doi: 10.3390/d1304016230654563

[B20] Craddock E. M. (2016). Profuse evolutionary diversification and speciation on volcanic islands: transposon instability and amplification bursts explain the genetic paradox. Biol. Direct 11, 44. doi: 10.1186/s13062-016-0146-1 27600528 PMC5012101

[B21] Dellinger A. S. Lagomarsino L. Michelangeli F. Dullinger S. Smith S. D. (2024). The sequential direct and indirect effects of mountain uplift, climatic niche, and floral trait evolution on diversification dynamics in an Andean plant clade. Syst. Biol. 73, 594–612. doi: 10.1093/sysbio/syae011 38554255 PMC11377192

[B22] Frahm J. P. Gradstein S. R. (1991). An altitudinal zonation of tropical rain forests using bryophytes. J. Biogeogr. 18, 669–678. doi: 10.2307/2845548

[B23] Gabriel R. Morgado L. N. Poponessi S. Henriques D. S. G. Coelho M. C. M. Silveira G. M. . (2025). Diversity and distribution of bryophytes along an altitudinal gradient on Flores Island (Azores, Portugal). Plants 14, 3766. doi: 10.3390/plants14243766 41470649 PMC12736622

[B24] Gauch H. G. Whittaker R. H. (1972). Coenocline simulation. Ecology 53, 446–451. doi: 10.2307/1934231

[B25] Geissler P. Velluti C. (1995). Ecology of alpine bryophytes. Giorn. Bot. Ital. 129, 199–204. doi: 10.1080/1126350950943612137339054

[B26] Grau O. Grytnes J. A. Birks H. J. B. (2007). A comparison of altitudinal species richness patterns of bryophytes with other plant groups in Nepal, Central Himalaya. J. Biogeogr. 34, 1907–1915. doi: 10.1111/j.1365-2699.2007.01745.x40046247

[B27] Grishin S. Y. (2011). Vegetation of the Volcanic Landscapes of the Kuril Islands and Kamchatka (Vladivostok: Dalnauka), 164.

[B28] Hanusch M. (2025). Vegetation structural complexity uniquely captures competition between vascular plants and bryophytes over succession. J. Ecol. 113, 2515–2525. doi: 10.1111/1365-2745.7011040046247

[B29] Henriques D. S. G. Borges P. A. V. Gabriel R. (2017). Regional processes drive bryophyte diversity and community composition in a small oceanic island. Community Ecol. 18, 193–202. doi: 10.1556/168.2017.18.2.9

[B30] Herzog T. (1926). Geographie Der Moose (Jena: Gustav Fischer), 439.

[B31] Hübener J. W. P. (1834). Hepaticologia Germanica, Oder Beschreibung Der Deutschen Lebermoose (Mannheim: Schwan- und Götz'sche Hofbuchhandlung), 314.

[B32] Karadeniz E. Sengun M. T. Sunbul F. (2026). From local pressures to global priorities: a PSR-guided model for spatial biodiversity conservation. Biodivers. Conserv. 35, 81. doi: 10.1007/s10531-026-03293-030311153

[B33] Klimova K. G. Bakalin V. A. Bakalin D. A. Choi S. S. (2024). A model of southern Sikhote-Alin liverwort flora and a new approach to analyze the altitudinal distribution patterns in the Zov Tigra National Park (South of the Russian Far East, Temperate Pacific Asia). Diversity 16, 752. doi: 10.3390/d1612075230654563

[B34] Komarov V. L. (1912). Puteshestvie Po Kamchatke V 1908-1909 Gg. [a Journey Through Kamchatka in 1908-1909] (Moscow: P. P. Ryabushinsky Printing House), 428.

[B35] Konstantinova N. A. Vilnet A. A. Long D. G. Mamontov Y. S. (2025). Hidden diversity and the treatment of Jungermannia pumila s.l. with description of a new subspecies from the Russian Far East. Arctoa 34, 133–151. doi: 10.15298/arctoa.34.11

[B36] Krasheninnikov S. P. Bazanova L. I. Ponomareva V. V. Portnyagin M. V. (2020). Detailed tephrochronology and composition of major Holocene eruptions from Avachinsky, Kozelsky, and Koryaksky volcanoes in Kamchatka. J. Volcanol. Geotherm. Res. 408, 107088. doi: 10.1016/j.jvolgeores.2020.10708842574925

[B37] Mamontov Y. S. Vilnet A. A. Atwood J. J. Konstantinova N. A. (2020). Molecular phylogenetic study of Frullania subsect. Inflatae (Frullaniaceae, Marchantiophyta) in the Holarctic with description of a new subgenus and three new species. Nova Hedwigia Beih. 150, 201–242. doi: 10.1127/nova-suppl/2020/20139861660

[B38] Maul K. Wei Y. M. Iskandar E. A. P. Chantanaorrapint S. Ho B. C. Quandt D. . (2023). Liverworts show a globally consistent mid-elevation richness peak. Ecol. Evol. 13, e9862. doi: 10.1002/ece3.9862 36969936 PMC10034488

[B39] Mejia A. Castro V. Peralta D. F. Moncada B. (2020). Altitudinal zonation of mosses in west of the Sierra Nevada of Cocuy, Boyacá, Colombia. Hoehnea 47, e162020. doi: 10.1590/2236-8906-16/2020

[B40] Molina-Venegas R. Fischer M. Hemp A. (2020). Plant evolutionary assembly along elevational belts at Mt. Kilimanjaro: using phylogenetics to asses biodiversity threats under climate change. Environ. Exp. Bot. 170, 103853. doi: 10.1016/j.envexpbot.2019.10385342574925

[B41] Neshataeva V. Y. (2009). The Vegetation of the Kamchatka Peninsula (Moscow: KMK Scientific Press).

[B42] Nogales M. Izquierdo I. Martín J. L. Rando J. C. García-Olivares A. Oromí P. (2022). The fate of terrestrial biodiversity during an oceanic island volcanic eruption. Sci. Rep. 12, 19344. doi: 10.1038/s41598-022-22863-0 36369519 PMC9652411

[B43] Odum E. P. (1971). Fundamentals of Ecology. 3rd Edn (Philadelphia: W.B. Saunders Company), xiv, 574.

[B44] Paton J. A. (1999). The Liverwort Flora of the British Isles (Colchester: Harley Books), 626.

[B45] Pineda-López R. Tepos-Ramírez M. Ocaña Feregrino A. Altamirano T. A. (2020). Treeline ecotones shape the distribution of avian species richness and functional diversity in south temperate mountains. Sci. Rep. 10, 18428. doi: 10.1038/s41598-020-75470-2 33116173 PMC7595238

[B46] Schofield W. B. (1985). Introduction to Bryology (New York: Macmillan; London: Collier Macmillan), 431.

[B47] Schuster R. M. (1984). “ Phytogeography of the bryophyta,” in New Manual of Bryology, vol. 1 . Ed. Schuster R. M. ( Hattori Botanical Laboratory, NiChinan), 463–626.

[B48] SciPy (2026). Open Source Scientific Tools for Python. Available online at: https://docs.scipy.org/ (Accessed June 5, 2026).

[B49] Silveira F. A. O. Barbosa M. Beiroz W. Callisto M. Macedo D. R. Morellato L. P. C. . (2019). Tropical mountains as natural laboratories to study global changes: a long-term ecological research project in a megadiverse biodiversity hotspot. Perspect. Plant Ecol. Evol. Syst. 38, 64–73. doi: 10.1016/j.ppees.2019.04.00142574925

[B50] Söderström L. Hagborg A. von Konrat M. Bartholomew-Began S. Bell D. Briscoe L. . (2016). World checklist of hornworts and liverworts. PhytoKeys 59, 1–828. doi: 10.3897/phytokeys.59.6261 26929706 PMC4758082

[B51] Song X. Gu J. Ye Y. Li W. Liao Y. Wang R. . (2024). The diversity and community pattern of liverworts on Sygera Mountain, Tibet. Forests 15, 48. doi: 10.3390/f1501004830654563

[B52] Stehn S. E. Webster C. R. Glime J. M. Jenkins M. A. (2010). Elevational gradients of bryophyte diversity, life forms, and community assemblage in the southern Appalachian Mountains. Can. J. For. Res. 40, 2164–2174. doi: 10.1139/X10-15634819996

[B53] Sun S.-Q. Wu Y.-H. Wang G.-X. Zhou J. Yu D. Bing H.-J. . (2013). Bryophyte species richness and composition along an altitudinal gradient in Gongga Mountain, China. PloS One 8, e58131. doi: 10.1371/journal.pone.0058131 23472146 PMC3589371

[B54] Ter Braak C. J. F. (1986). Canonical correspondence analysis: a new eigenvector technique for multivariate direct gradient analysis. Ecology 67, 1167–1179. doi: 10.2307/1938672

[B55] Tsuyuzaki S. (1991). Species turnover and diversity during early stages of vegetation recovery on the volcano Usu, Japan. J. Veg. Sci. 2, 301–306. doi: 10.2307/3235920

[B56] Whittaker R. H. (1967). Gradient analysis of vegetation. Biol. Rev. 42, 207–264. doi: 10.1111/j.1469-185X.1967.tb01419.x 4859903

