# Supplementary material for: Liverwort altitudinal patterns in East Kamchatka: dividing the indivisible
Source: Front Plant Sci. 2026 Sep 11;17:1904286. doi: 10.3389/fpls.2026.1904286 (PMC13612434; doi:10.3389/fpls.2026.1904286)
Supplement: Supplementary file 1 [file DataSheet1.pdf]

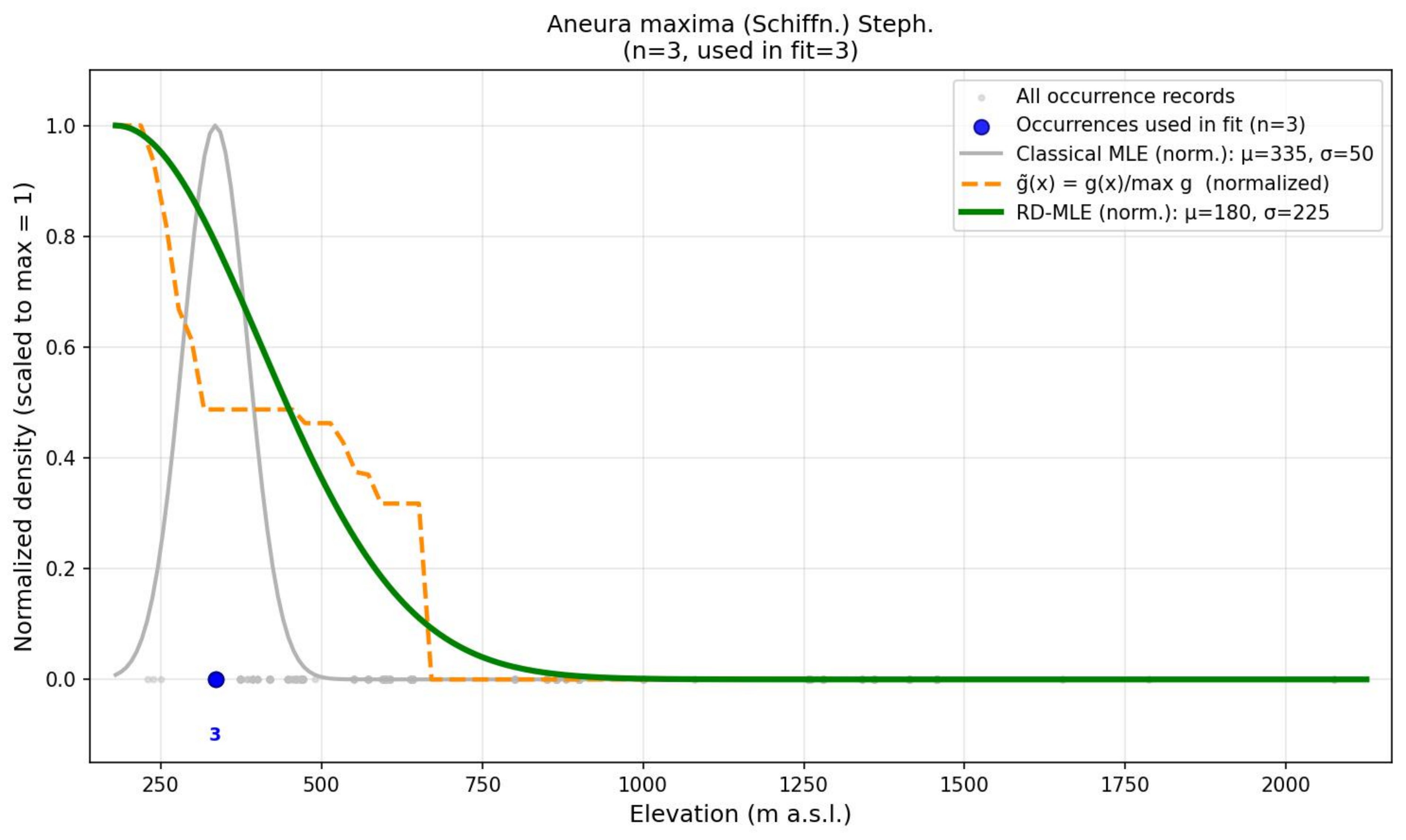

Aneura maxima (Schiffn.) Steph.
(n=3, used in fit=3)
All occurrence records
Occurrences used in fit (n=3)
Classical MLE (norm.): μ=335, σ=50
g̃(x) = g(x)/max g (normalized)
RD-MLE (norm.): μ=180, σ=225
Normalized density (scaled to max = 1)
Elevation (m a.s.l.)
1.0
0.8
0.6
0.4
0.2
0.0
250
500
750
1000
1250
1500
1750
2000
3

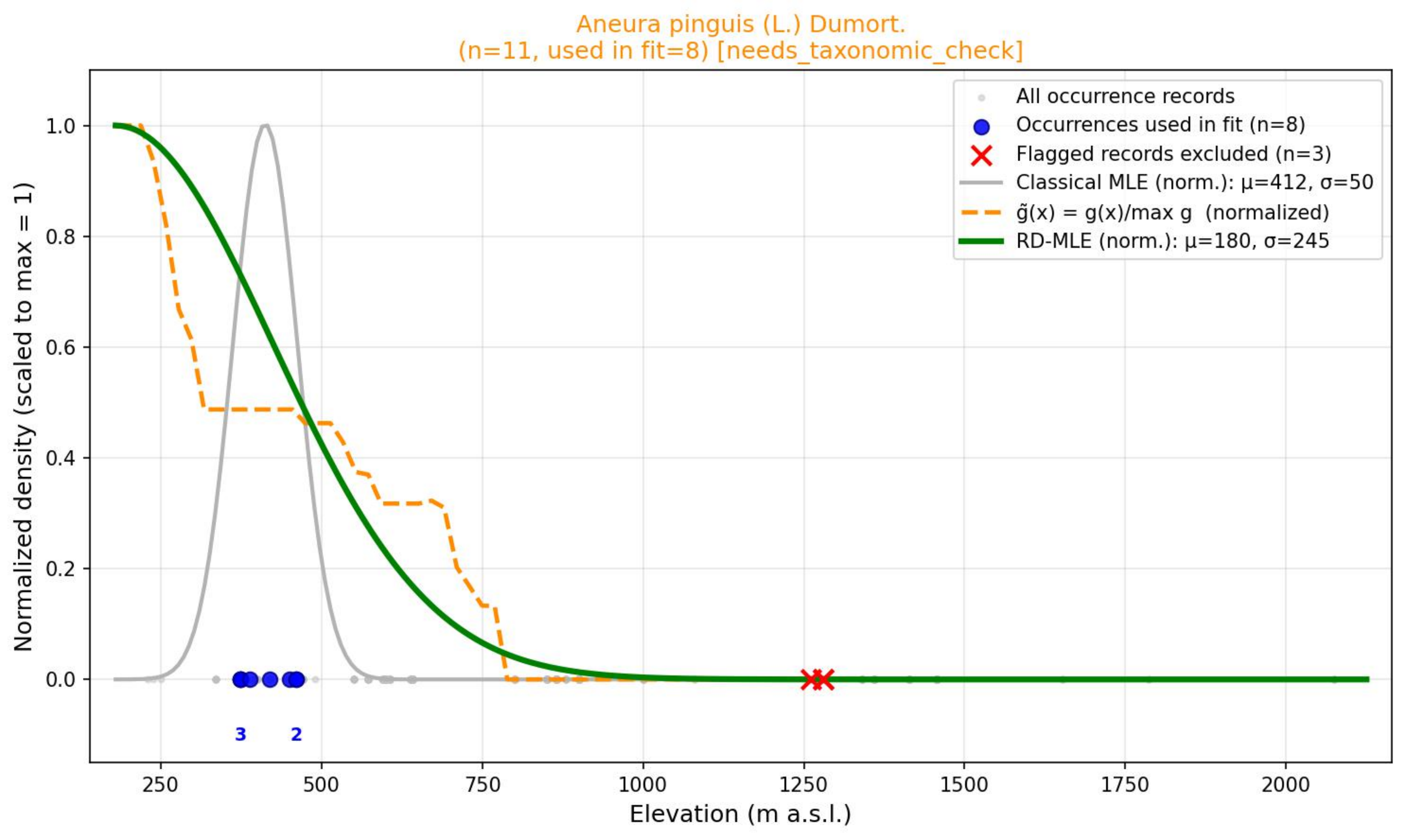
Aneura pinguis (L.) Dumort.
(n=11, used in fit=8) [needs_taxonomic_check]
All occurrence records
Occurrences used in fit (n=8)
Flagged records excluded (n=3)
Classical MLE (norm.): μ=412, σ=50
g̃(x) = g(x)/max g (normalized)
RD-MLE (norm.): μ=180, σ=245
Normalized density (scaled to max = 1)
Elevation (m a.s.l.)
0.0
0.2
0.4
0.6
0.8
1.0
250
500
750
1000
1250
1500
1750
2000
3
2

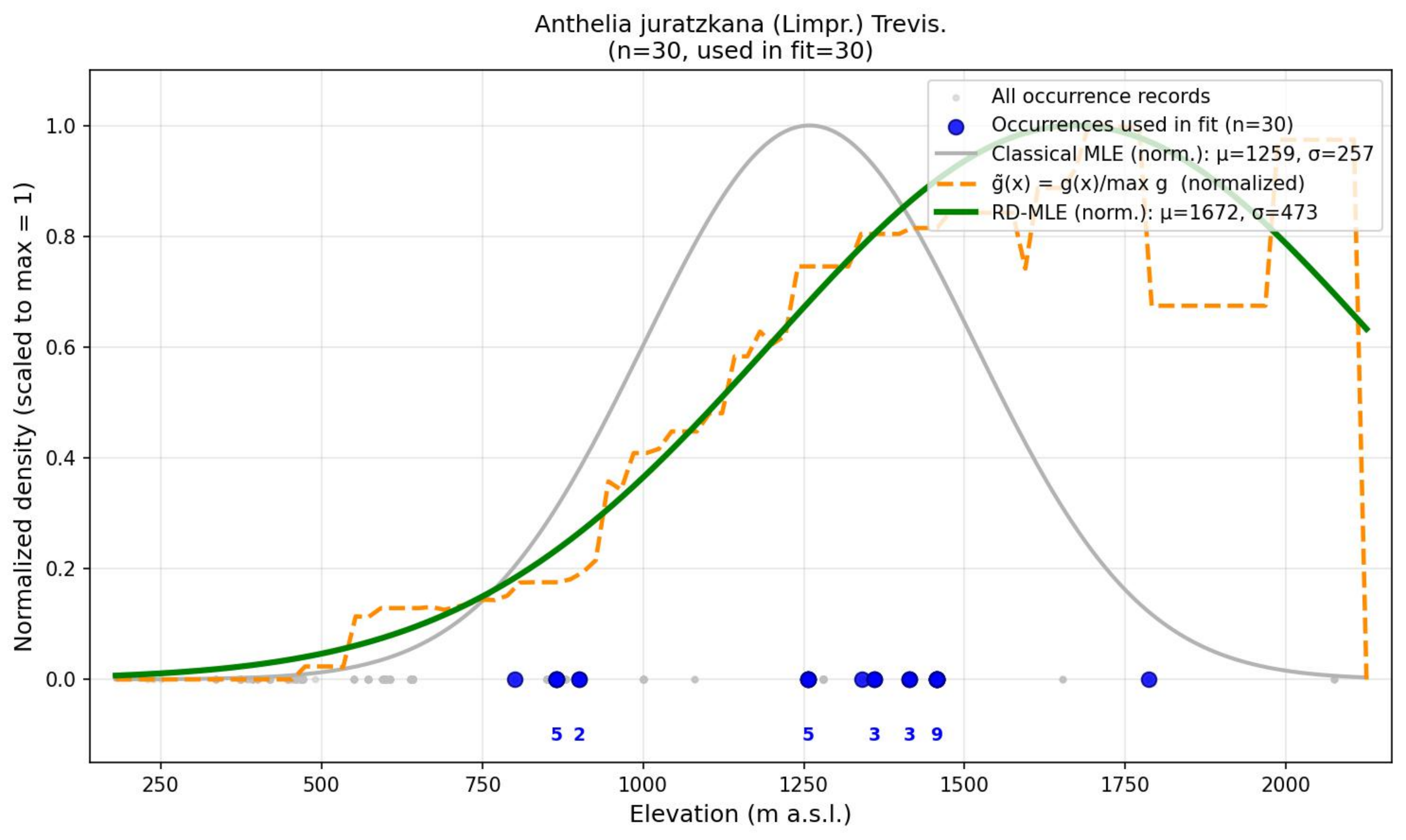
Anthelia juratzkana (Limpr.) Trevis.
(n=30, used in fit=30)
All occurrence records
Occurrences used in fit (n=30)
Classical MLE (norm.): μ=1259, σ=257
g̃(x) = g(x)/max g (normalized)
RD-MLE (norm.): μ=1672, σ=473
Normalized density (scaled to max = 1)
Elevation (m a.s.l.)
5
2
5
3
3
9

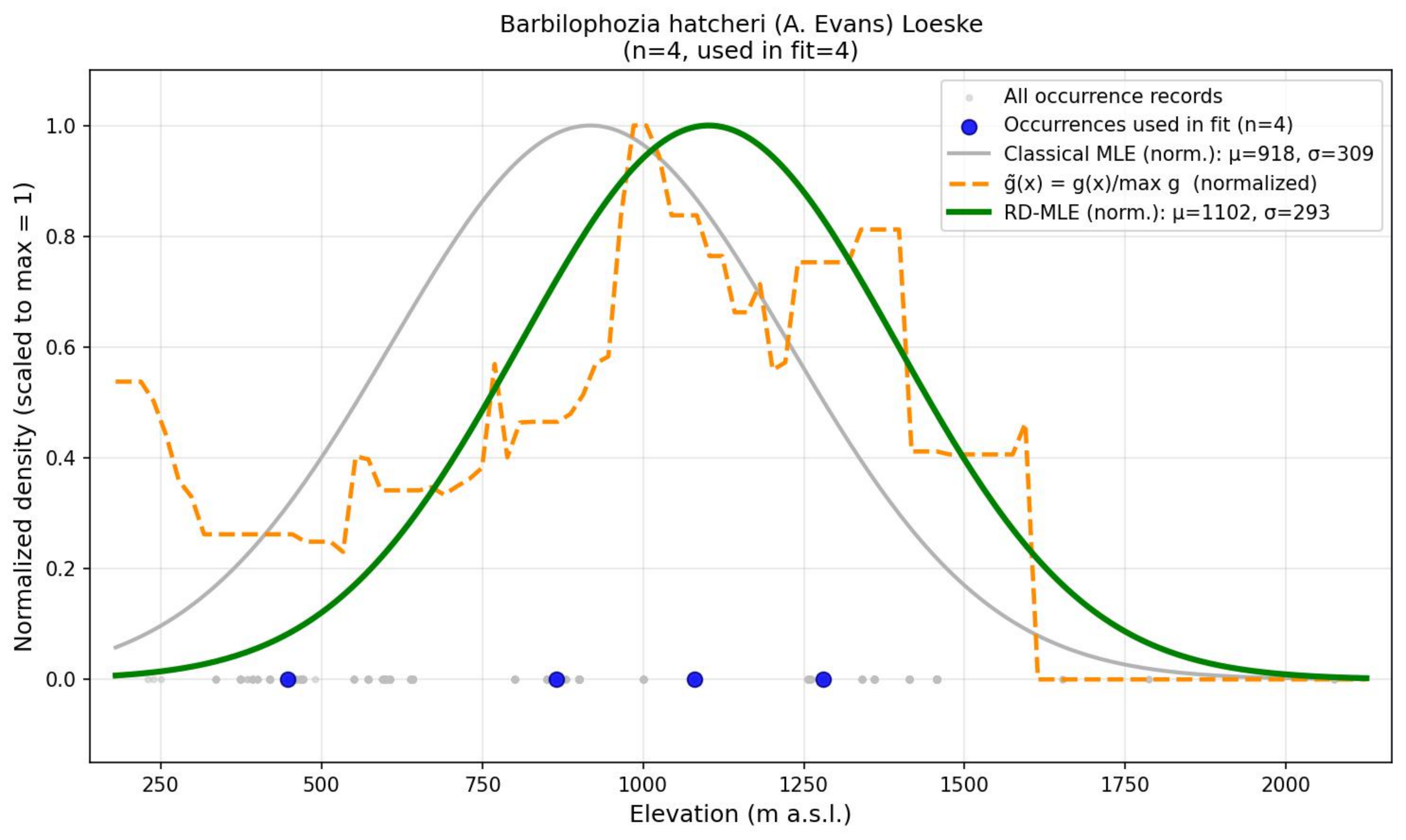
Barbilophozia hatcheri (A. Evans) Loeske
(n=4, used in fit=4)
All occurrence records
Occurrences used in fit (n=4)
Classical MLE (norm.): μ=918, σ=309
g̃(x) = g(x)/max g (normalized)
RD-MLE (norm.): μ=1102, σ=293
Normalized density (scaled to max = 1)
Elevation (m a.s.l.)
0.0
0.2
0.4
0.6
0.8
1.0
250
500
750
1000
1250
1500
1750
2000

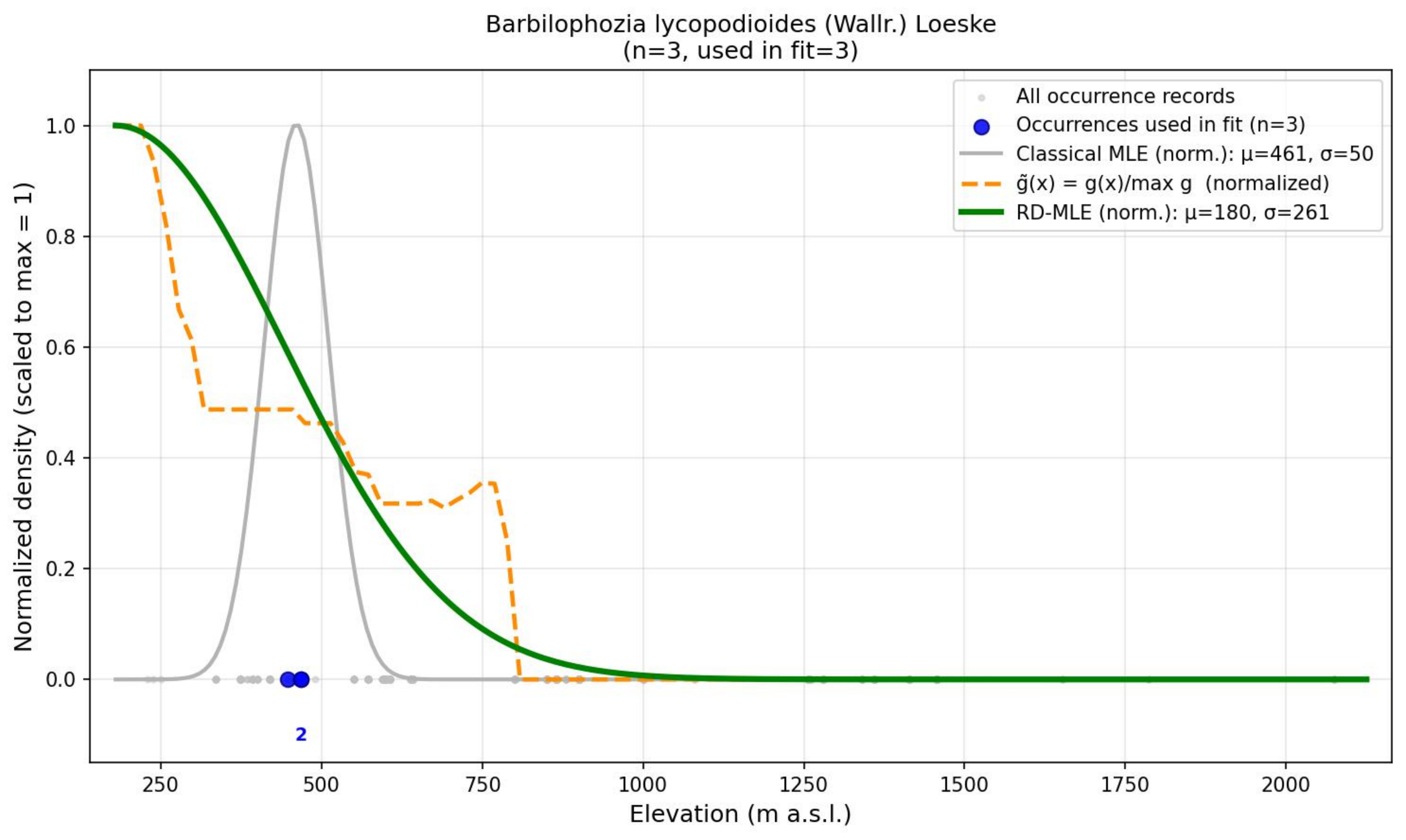
Barbilophozia lycopodioides (Wallr.) Loeske
(n=3, used in fit=3)
All occurrence records
Occurrences used in fit (n=3)
Classical MLE (norm.): μ=461, σ=50
g̃(x) = g(x)/max g (normalized)
RD-MLE (norm.): μ=180, σ=261
Normalized density (scaled to max = 1)
1.0
0.8
0.6
0.4
0.2
0.0
2
250
500
750
1000
1250
1500
1750
2000
Elevation (m a.s.l.)

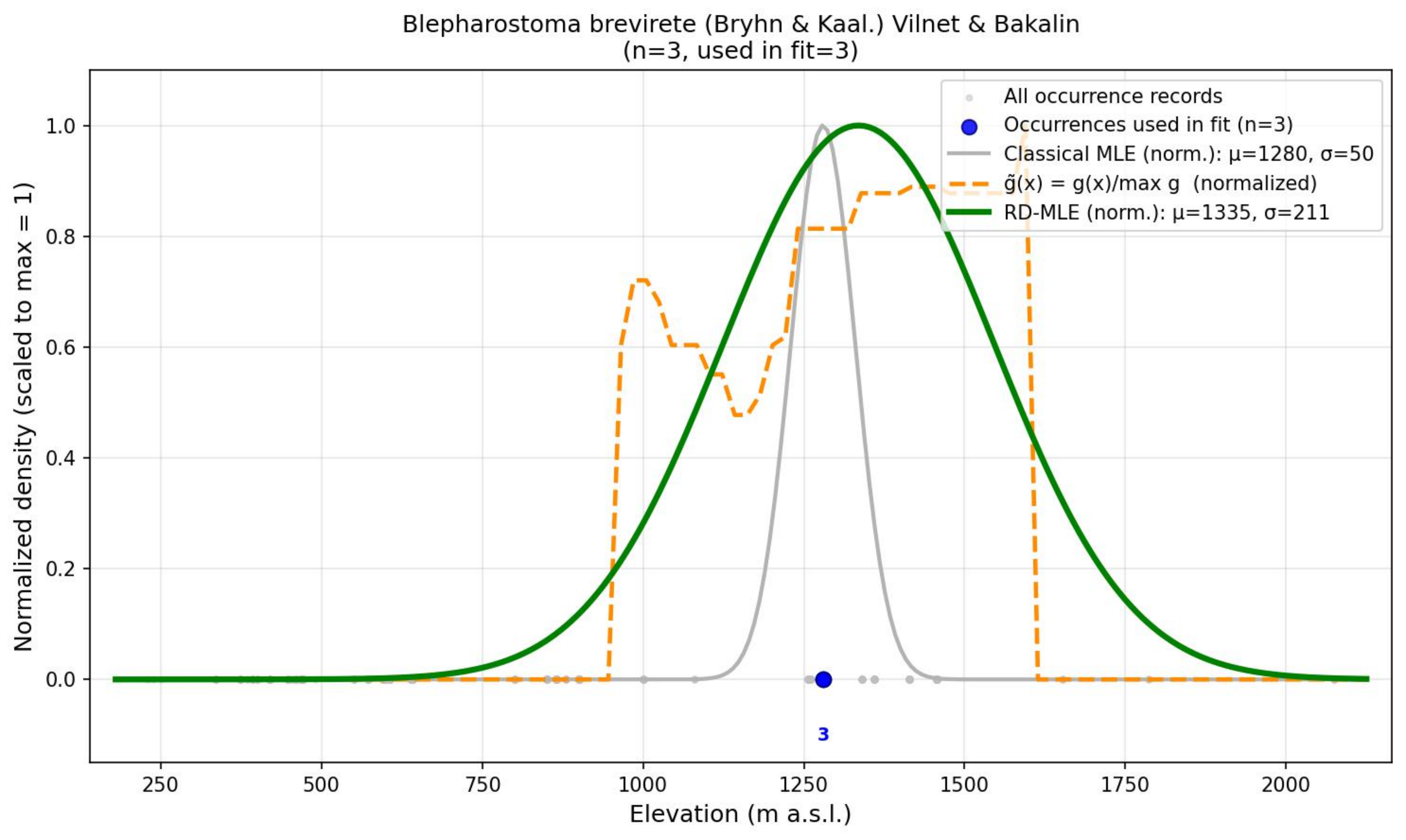

Blepharostoma brevirete (Bryhn & Kaal.) Vilnet & Bakalin
(n=3, used in fit=3)
All occurrence records
Occurrences used in fit (n=3)
Classical MLE (norm.): μ=1280, σ=50
g̃(x) = g(x)/max g (normalized)
RD-MLE (norm.): μ=1335, σ=211
Normalized density (scaled to max = 1)
Elevation (m a.s.l.)
0.0
0.2
0.4
0.6
0.8
1.0
250
500
750
1000
1250
1500
1750
2000
3

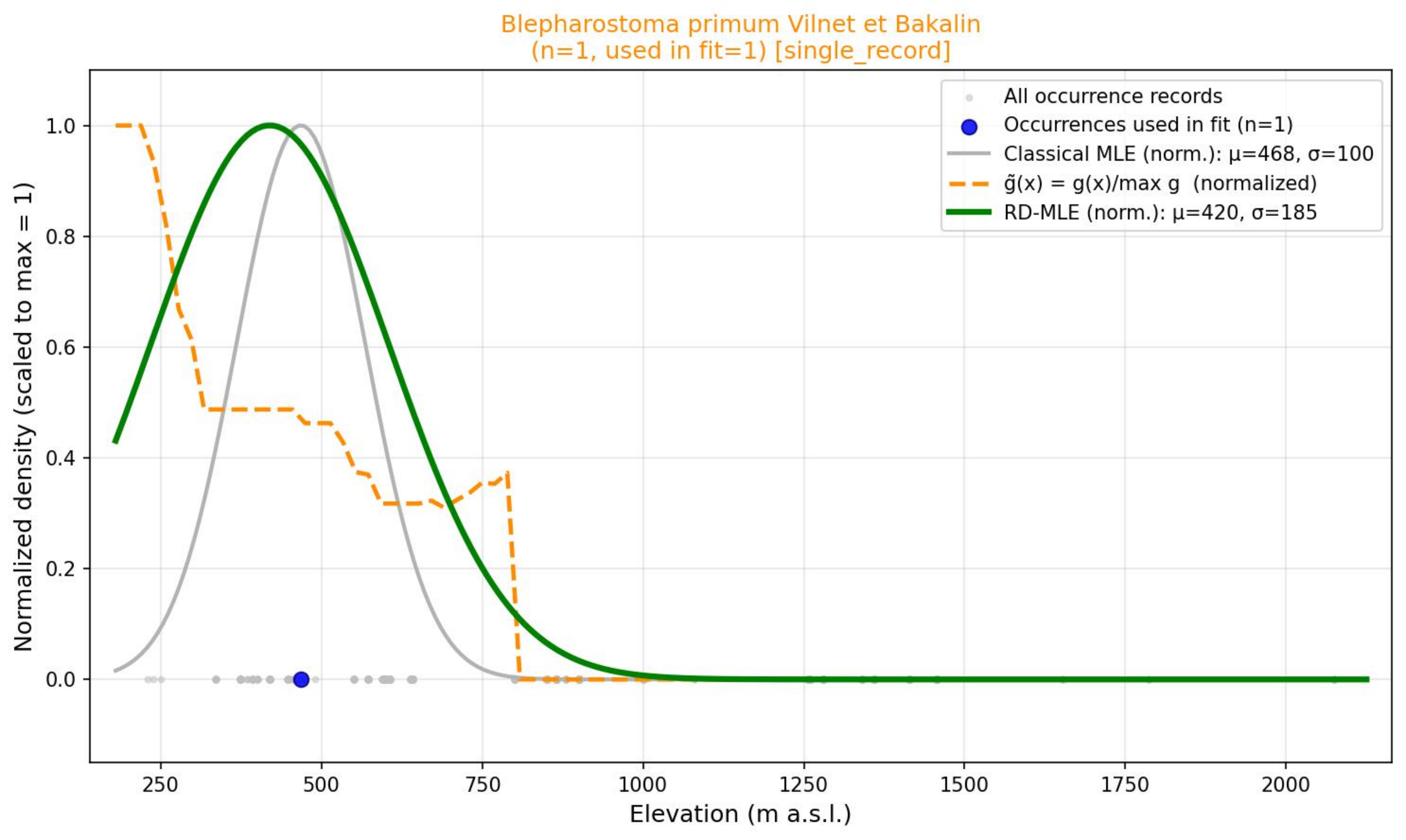

Blepharostoma primum Vilnet et Bakalin
(n=1, used in fit=1) [single_record]
All occurrence records
Occurrences used in fit (n=1)
Classical MLE (norm.): μ=468, σ=100
g̃(x) = g(x)/max g (normalized)
RD-MLE (norm.): μ=420, σ=185
Normalized density (scaled to max = 1)
Elevation (m a.s.l.)
0.0
0.2
0.4
0.6
0.8
1.0
250
500
750
1000
1250
1500
1750
2000

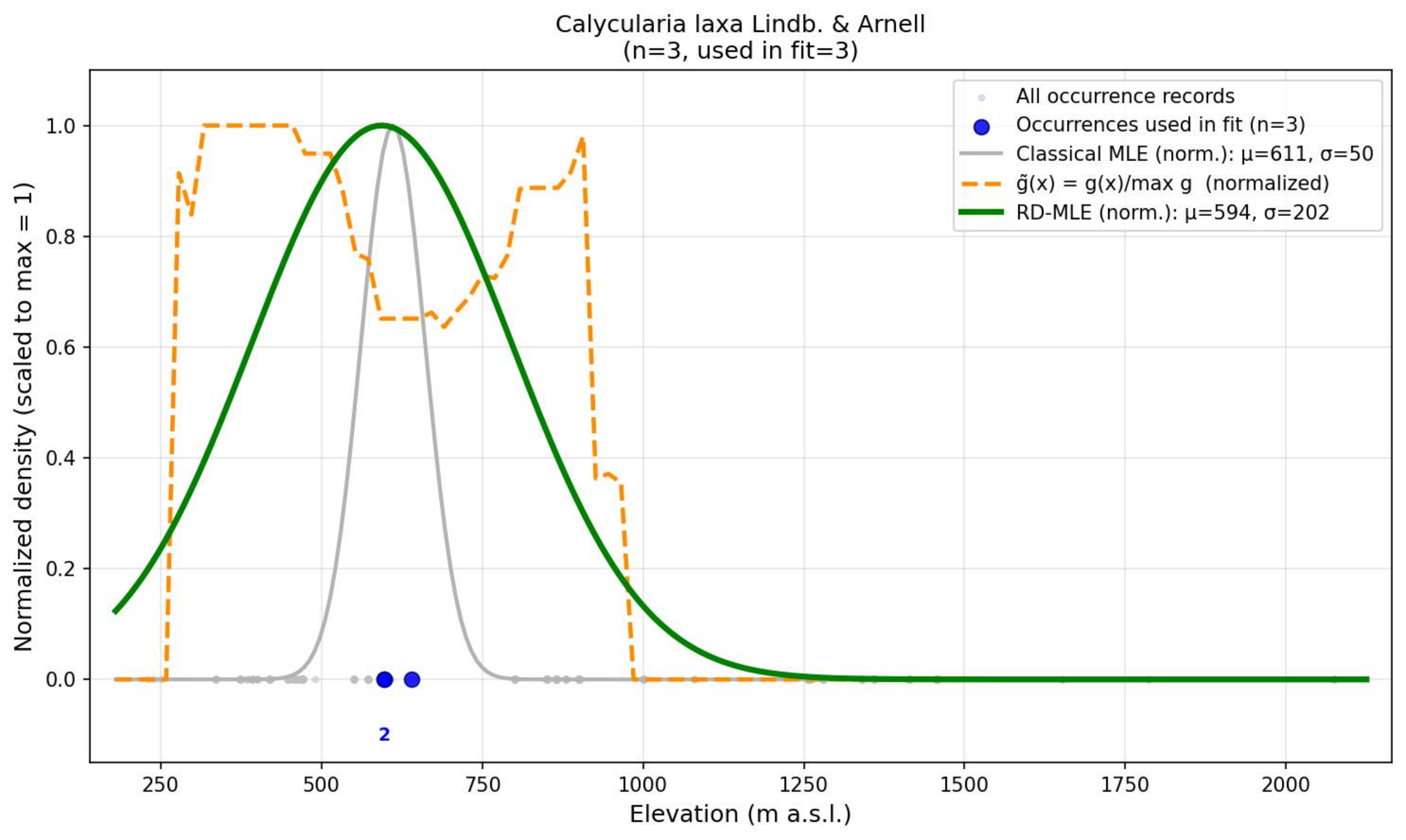
Calycularia laxa Lindb. & Arnell
(n=3, used in fit=3)
All occurrence records
Occurrences used in fit (n=3)
Classical MLE (norm.): μ=611, σ=50
g̃(x) = g(x)/max g (normalized)
RD-MLE (norm.): μ=594, σ=202
Normalized density (scaled to max = 1)
1.0
0.8
0.6
0.4
0.2
0.0
2
250
500
750
1000
1250
1500
1750
2000
Elevation (m a.s.l.)

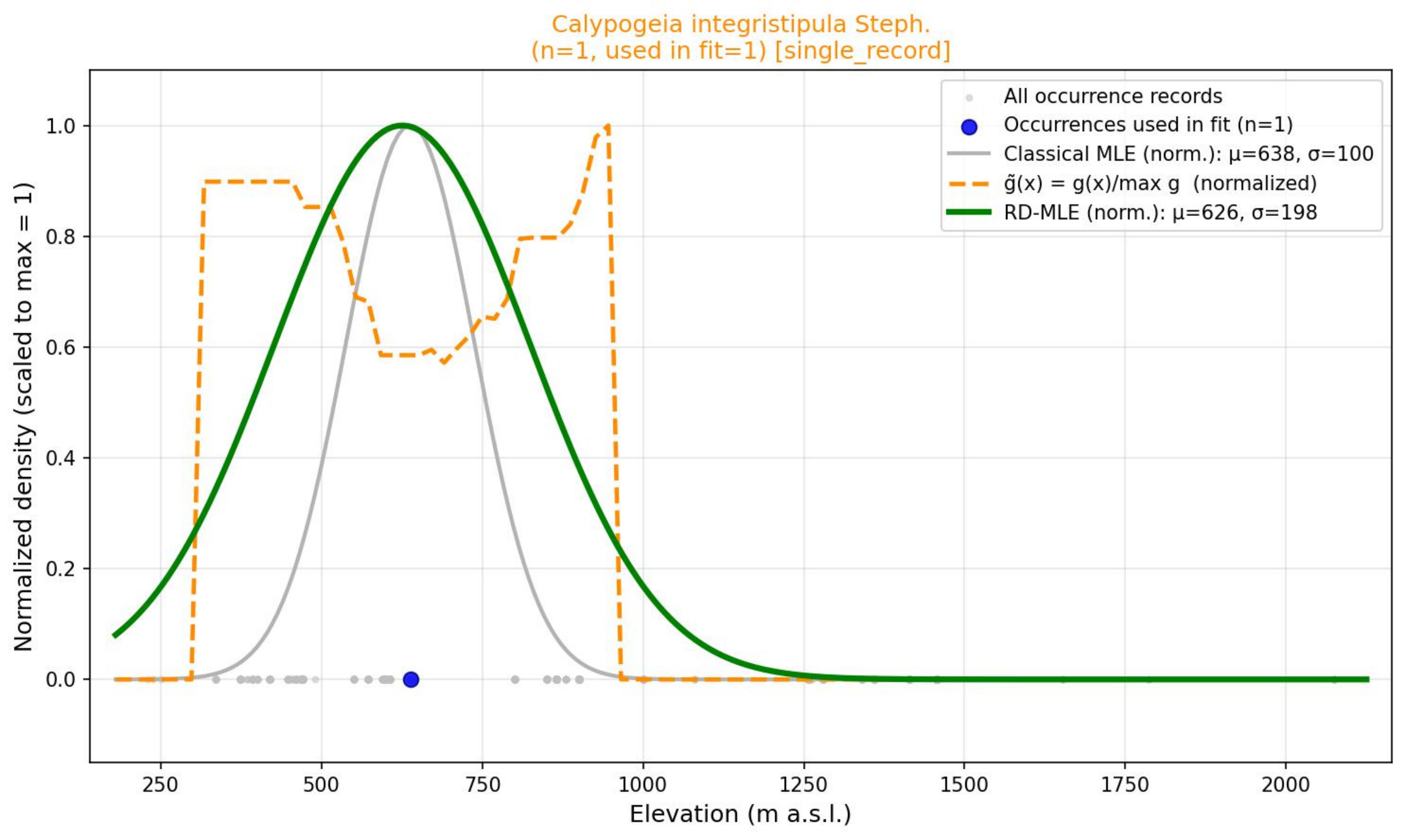
Calypogeia integristipula Steph.
(n=1, used in fit=1) [single_record]
All occurrence records
Occurrences used in fit (n=1)
Classical MLE (norm.): μ=638, σ=100
g̃(x) = g(x)/max g (normalized)
RD-MLE (norm.): μ=626, σ=198
Normalized density (scaled to max = 1)
Elevation (m a.s.l.)
0.0
0.2
0.4
0.6
0.8
1.0
250
500
750
1000
1250
1500
1750
2000

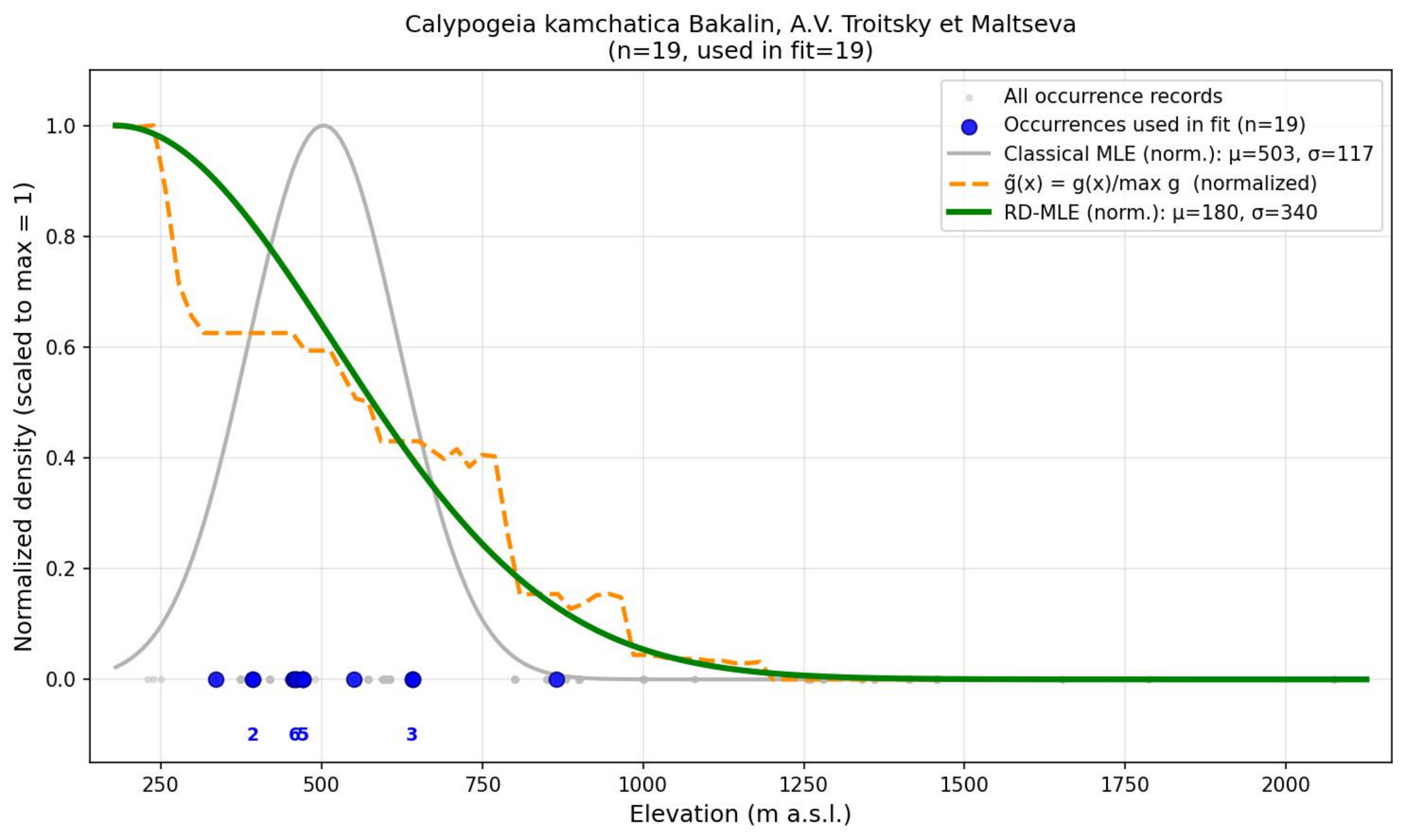

Calypogeia kamchatica Bakalin, A.V. Troitsky et Maltseva
(n=19, used in fit=19)
All occurrence records
Occurrences used in fit (n=19)
Classical MLE (norm.): μ=503, σ=117
g̃(x) = g(x)/max g (normalized)
RD-MLE (norm.): μ=180, σ=340
Normalized density (scaled to max = 1)
Elevation (m a.s.l.)
1.0
0.8
0.6
0.4
0.2
0.0
250
500
750
1000
1250
1500
1750
2000
2
65
3

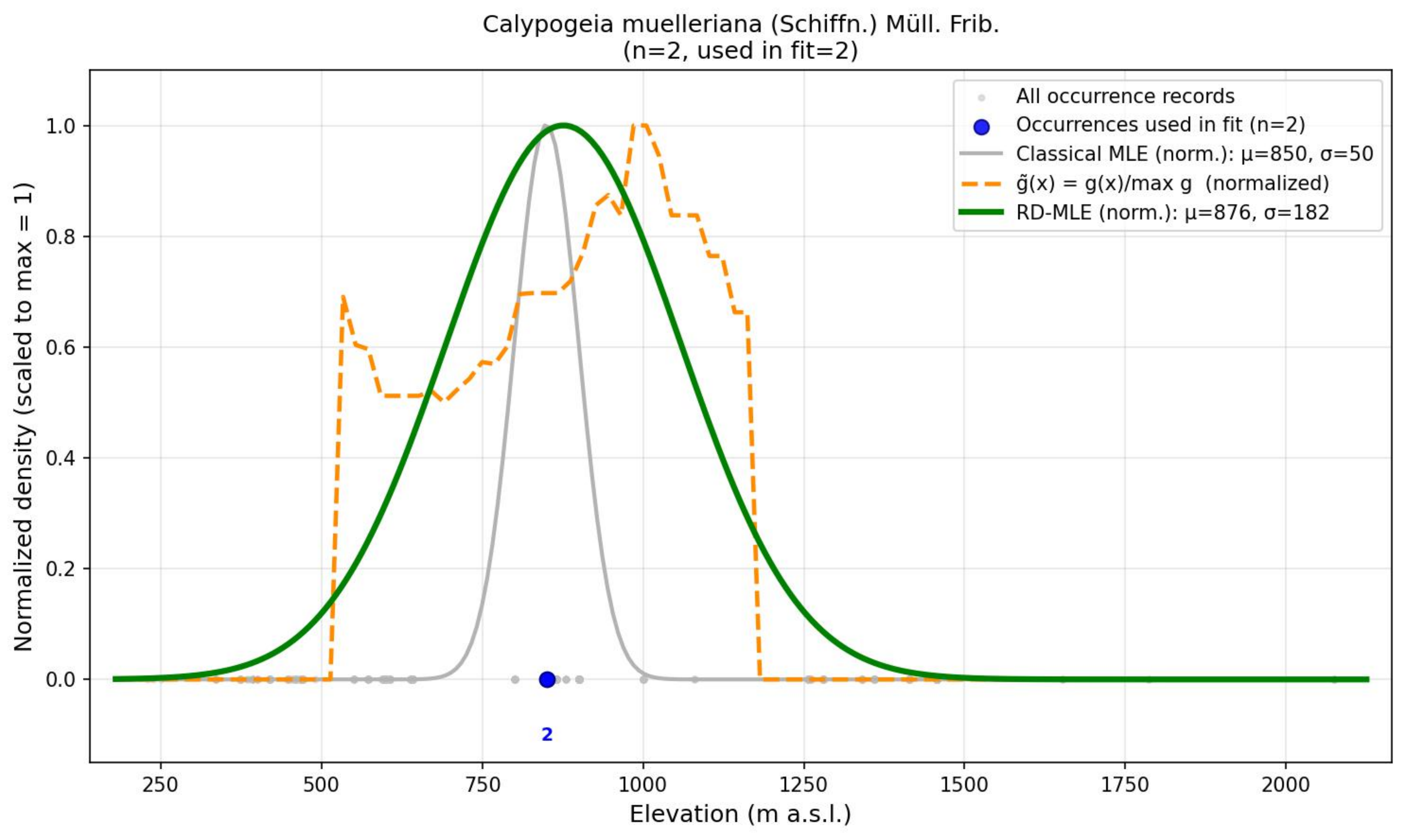
Calypogeia muelleriana (Schiffn.) Müll. Frib.
(n=2, used in fit=2)
All occurrence records
Occurrences used in fit (n=2)
Classical MLE (norm.): μ=850, σ=50
g̃(x) = g(x)/max g (normalized)
RD-MLE (norm.): μ=876, σ=182
Normalized density (scaled to max = 1)
1.0
0.8
0.6
0.4
0.2
0.0
2
250
500
750
1000
1250
1500
1750
2000
Elevation (m a.s.l.)

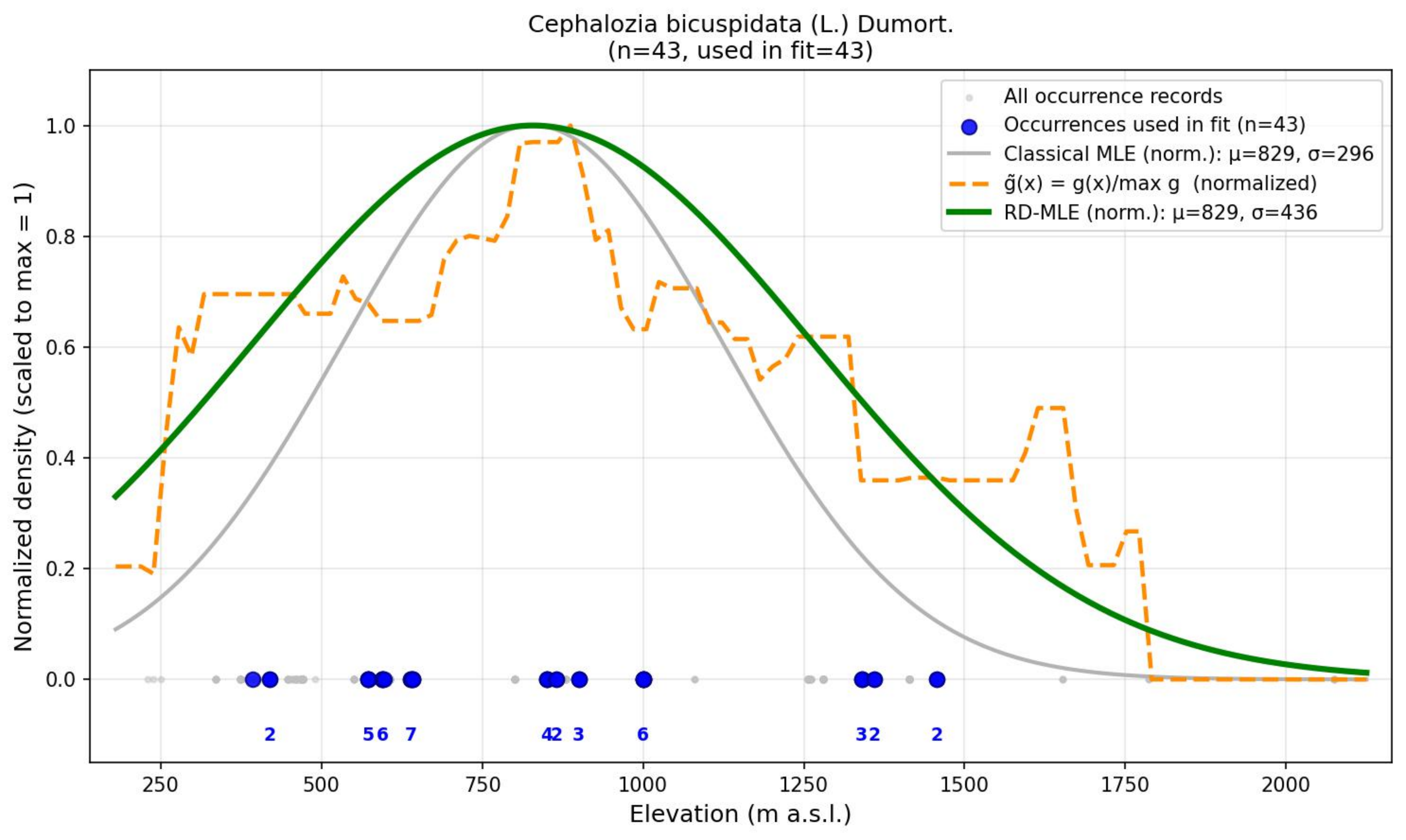
Cephalozia bicuspidata (L.) Dumort.
(n=43, used in fit=43)
All occurrence records
Occurrences used in fit (n=43)
Classical MLE (norm.): μ=829, σ=296
g̃(x) = g(x)/max g (normalized)
RD-MLE (norm.): μ=829, σ=436
Normalized density (scaled to max = 1)
Elevation (m a.s.l.)
1.0
0.8
0.6
0.4
0.2
0.0
250
500
750
1000
1250
1500
1750
2000
2
5
6
7
4
2
3
6
3
2
2

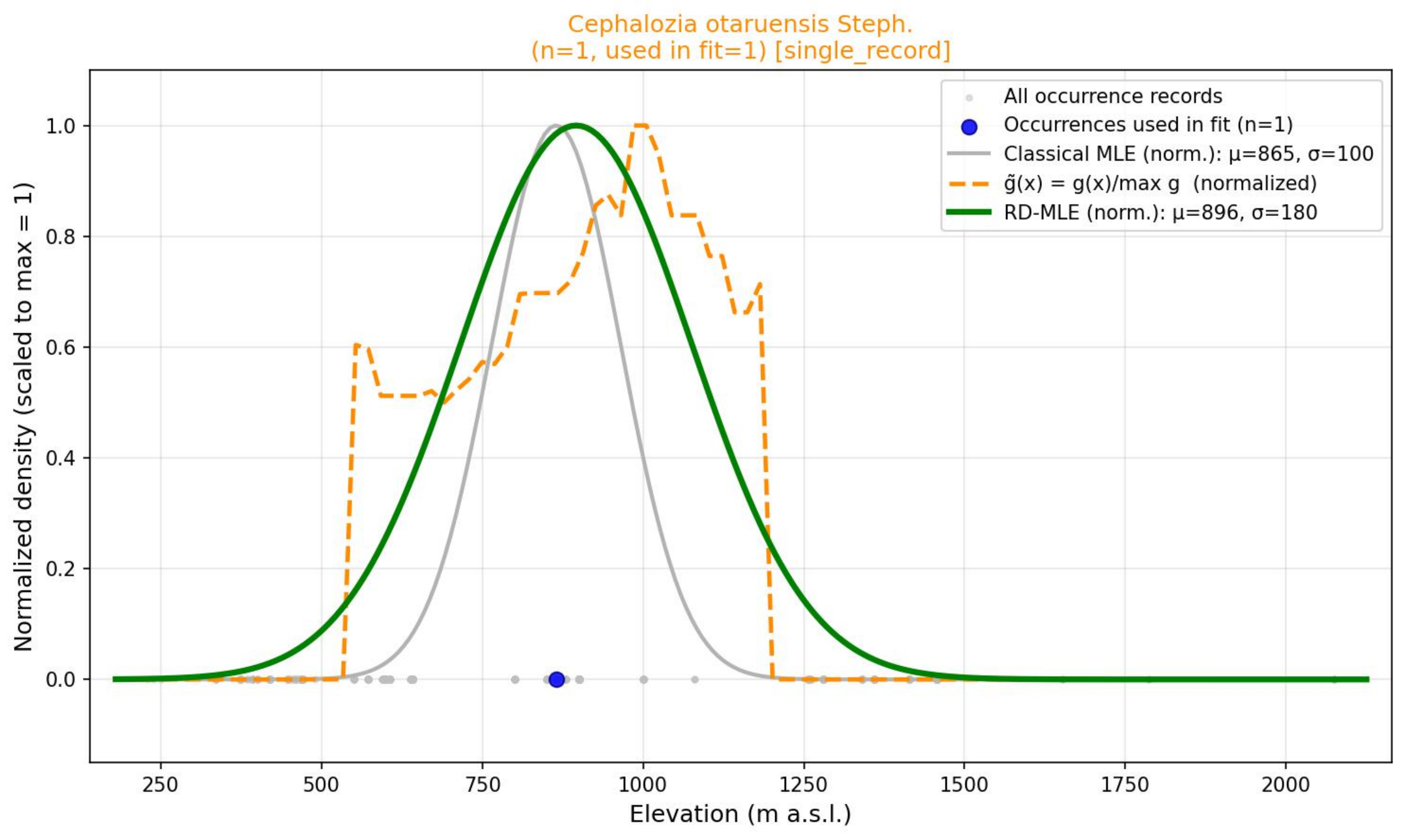
Cephalozia otaruensis Steph.
(n=1, used in fit=1) [single_record]
All occurrence records
Occurrences used in fit (n=1)
Classical MLE (norm.): μ=865, σ=100
g̃(x) = g(x)/max g (normalized)
RD-MLE (norm.): μ=896, σ=180
Normalized density (scaled to max = 1)
Elevation (m a.s.l.)
0.0
0.2
0.4
0.6
0.8
1.0
250
500
750
1000
1250
1500
1750
2000

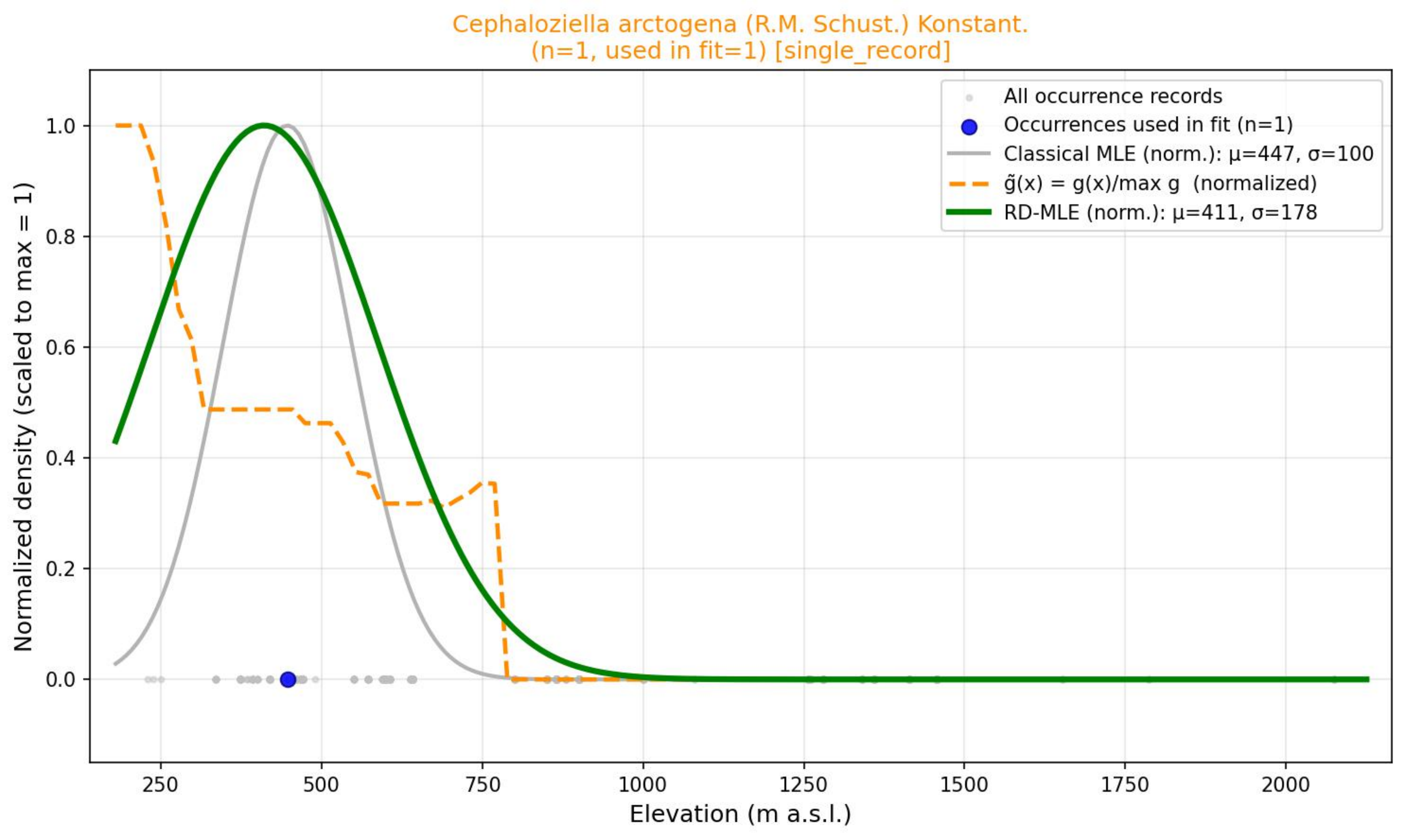
Cephaloziella arctogena (R.M. Schust.) Konstant.
(n=1, used in fit=1) [single_record]
All occurrence records
Occurrences used in fit (n=1)
Classical MLE (norm.): μ=447, σ=100
g̃(x) = g(x)/max g (normalized)
RD-MLE (norm.): μ=411, σ=178
Normalized density (scaled to max = 1)
1.0
0.8
0.6
0.4
0.2
0.0
250
500
750
1000
1250
1500
1750
2000
Elevation (m a.s.l.)

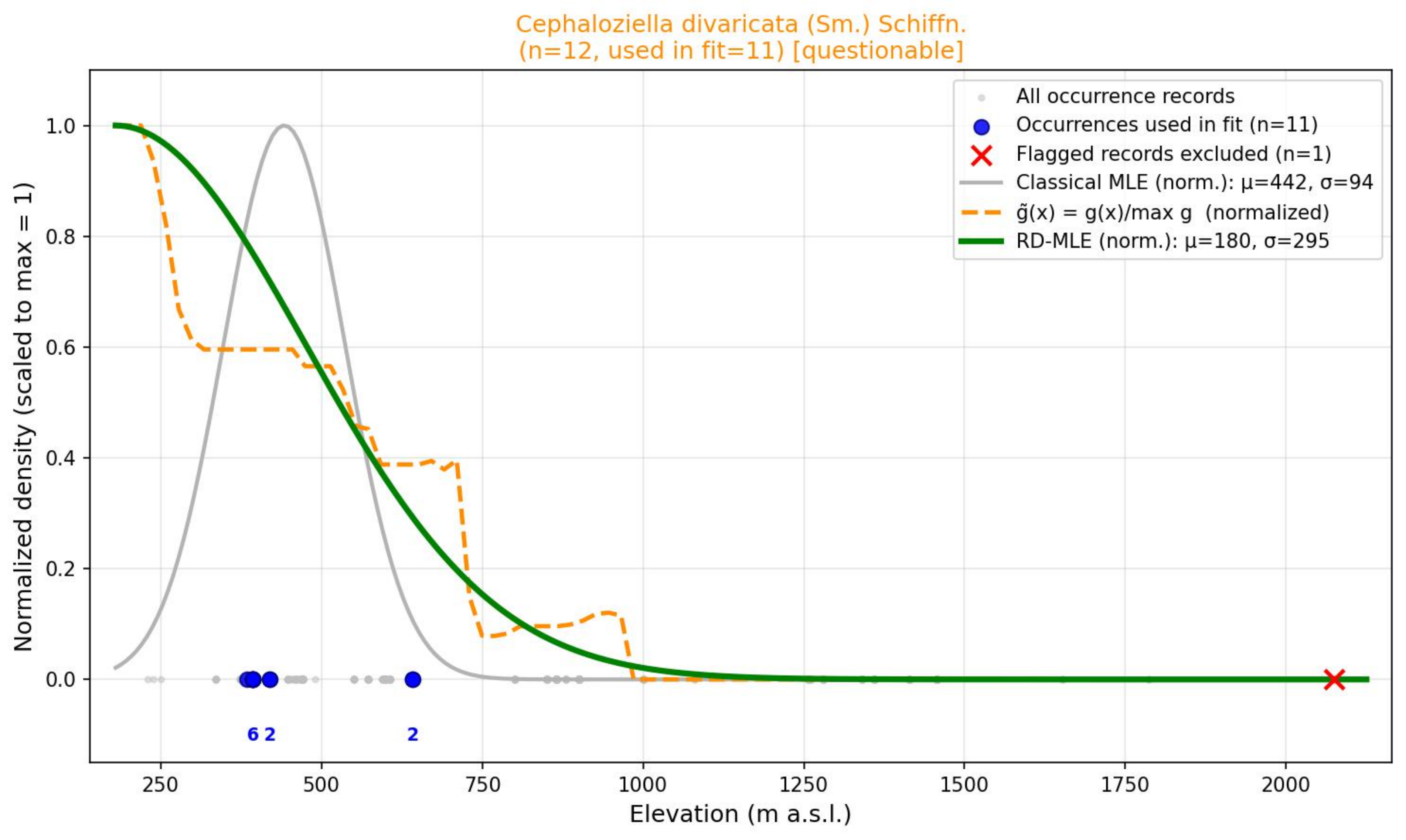
Cephaloziella divaricata (Sm.) Schiffn.
(n=12, used in fit=11) [questionable]
All occurrence records
Occurrences used in fit (n=11)
Flagged records excluded (n=1)
Classical MLE (norm.): μ=442, σ=94
g̃(x) = g(x)/max g (normalized)
RD-MLE (norm.): μ=180, σ=295
Normalized density (scaled to max = 1)
Elevation (m a.s.l.)
1.0
0.8
0.6
0.4
0.2
0.0
250
500
750
1000
1250
1500
1750
2000
6
2
2

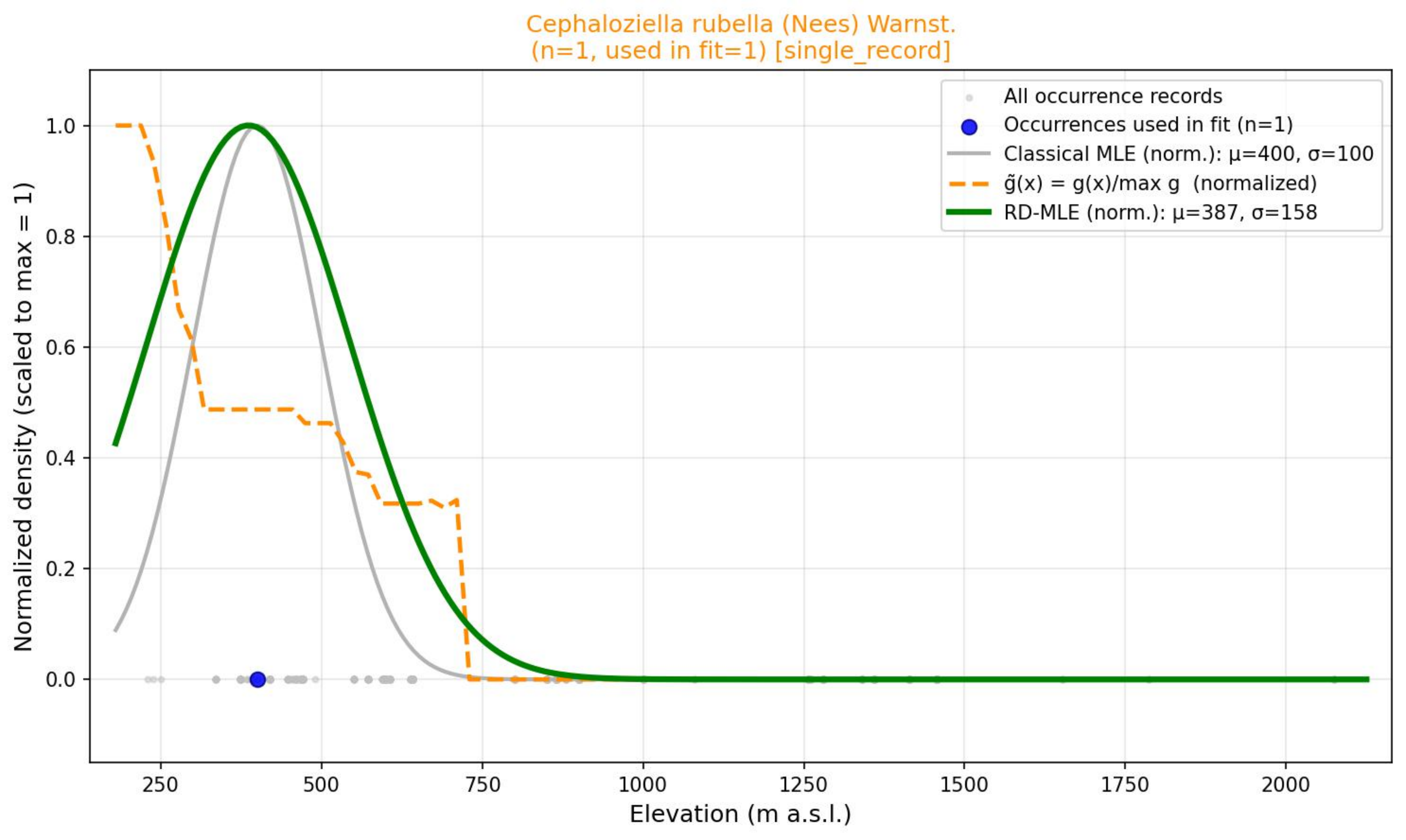
Cephaloziella rubella (Nees) Warnst.
(n=1, used in fit=1) [single_record]
All occurrence records
Occurrences used in fit (n=1)
Classical MLE (norm.): μ=400, σ=100
g̃(x) = g(x)/max g (normalized)
RD-MLE (norm.): μ=387, σ=158
Normalized density (scaled to max = 1)
Elevation (m a.s.l.)
1.0
0.8
0.6
0.4
0.2
0.0
250
500
750
1000
1250
1500
1750
2000

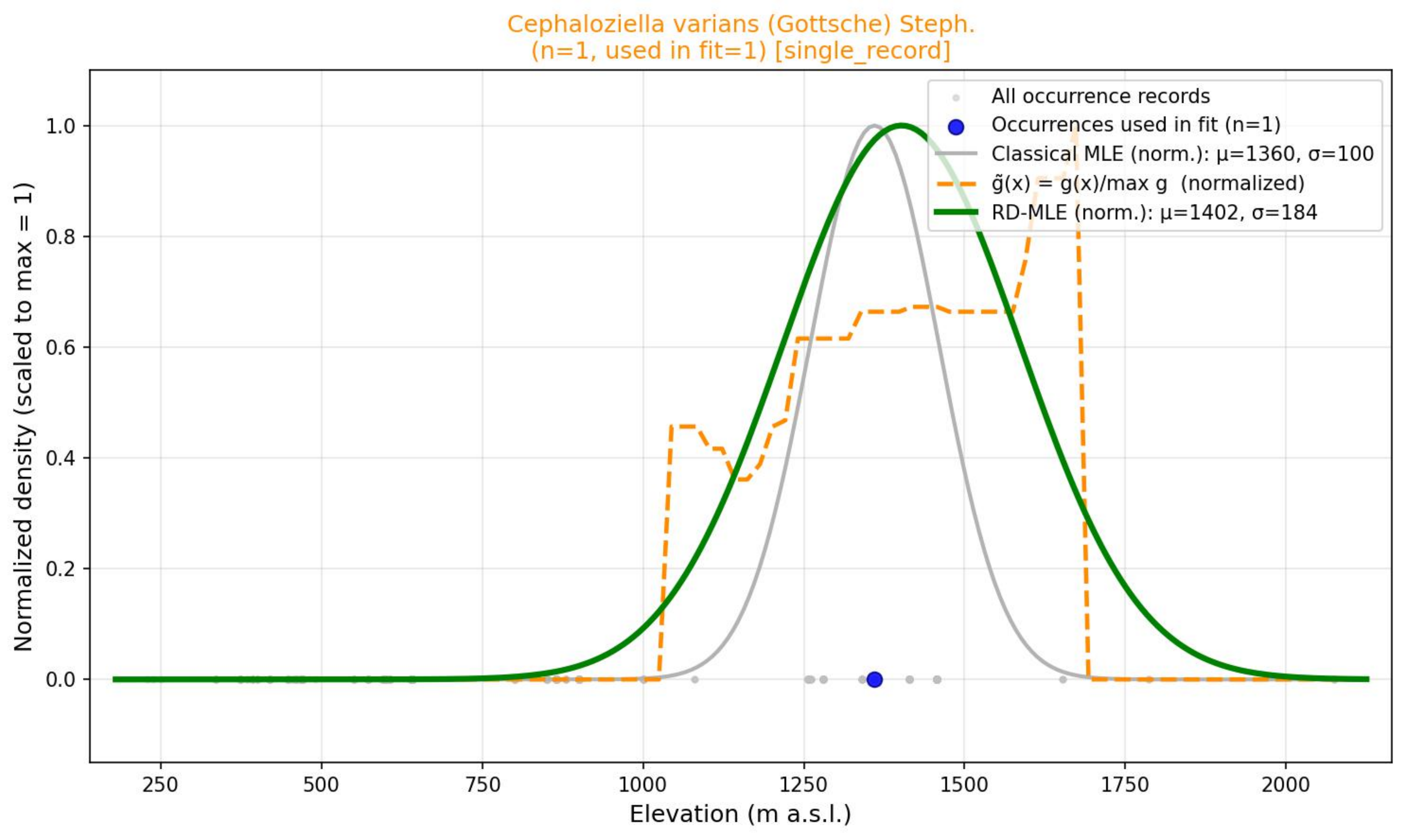

Cephaloziella varians (Gottsche) Steph.
(n=1, used in fit=1) [single_record]
All occurrence records
Occurrences used in fit (n=1)
Classical MLE (norm.): μ=1360, σ=100
g̃(x) = g(x)/max g (normalized)
RD-MLE (norm.): μ=1402, σ=184
Normalized density (scaled to max = 1)
Elevation (m a.s.l.)
0.0
0.2
0.4
0.6
0.8
1.0
250
500
750
1000
1250
1500
1750
2000

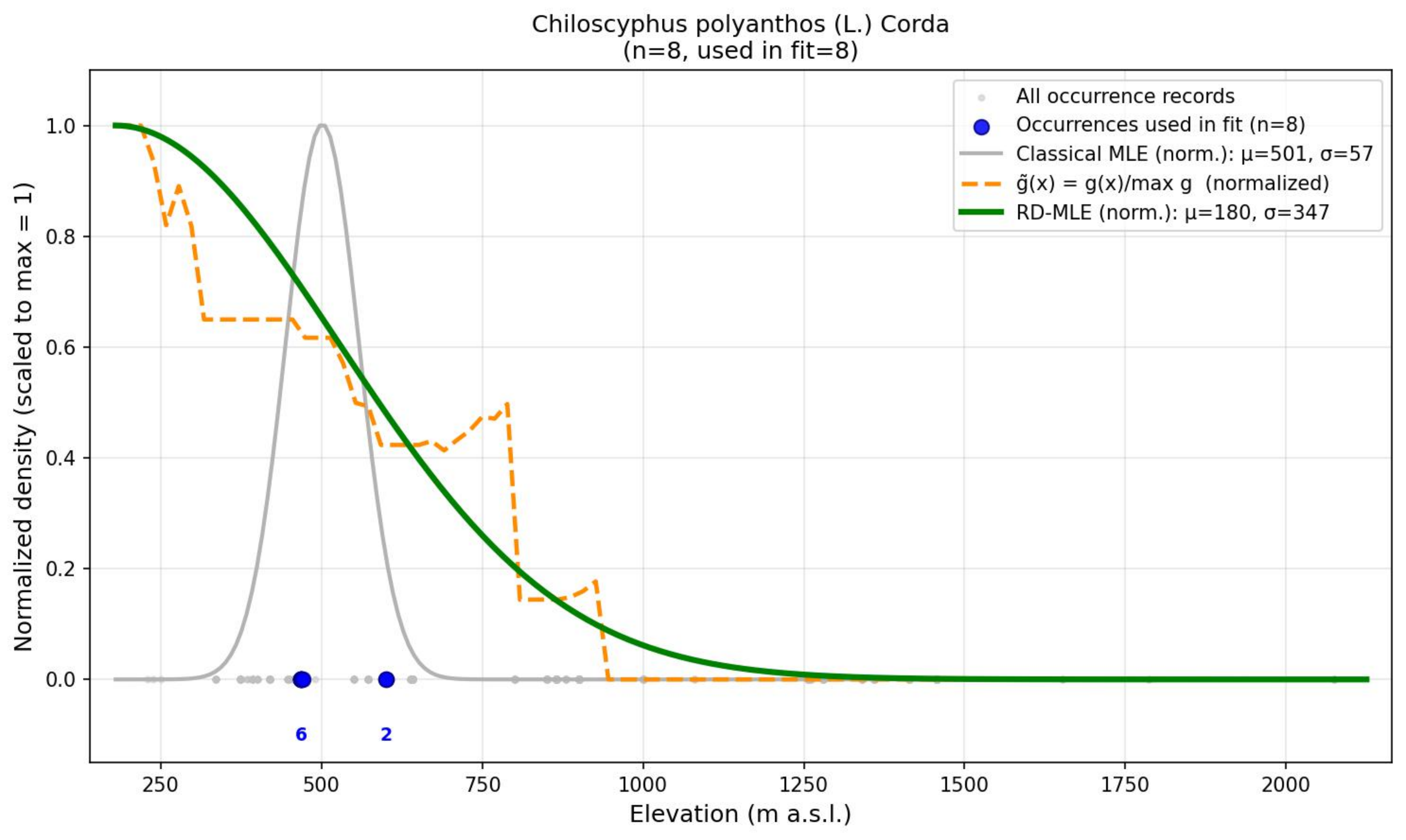
Chiloscyphus polyanthos (L.) Corda
(n=8, used in fit=8)
All occurrence records
Occurrences used in fit (n=8)
Classical MLE (norm.): μ=501, σ=57
g̃(x) = g(x)/max g (normalized)
RD-MLE (norm.): μ=180, σ=347
Normalized density (scaled to max = 1)
Elevation (m a.s.l.)
6
2
0.0
0.2
0.4
0.6
0.8
1.0
250
500
750
1000
1250
1500
1750
2000

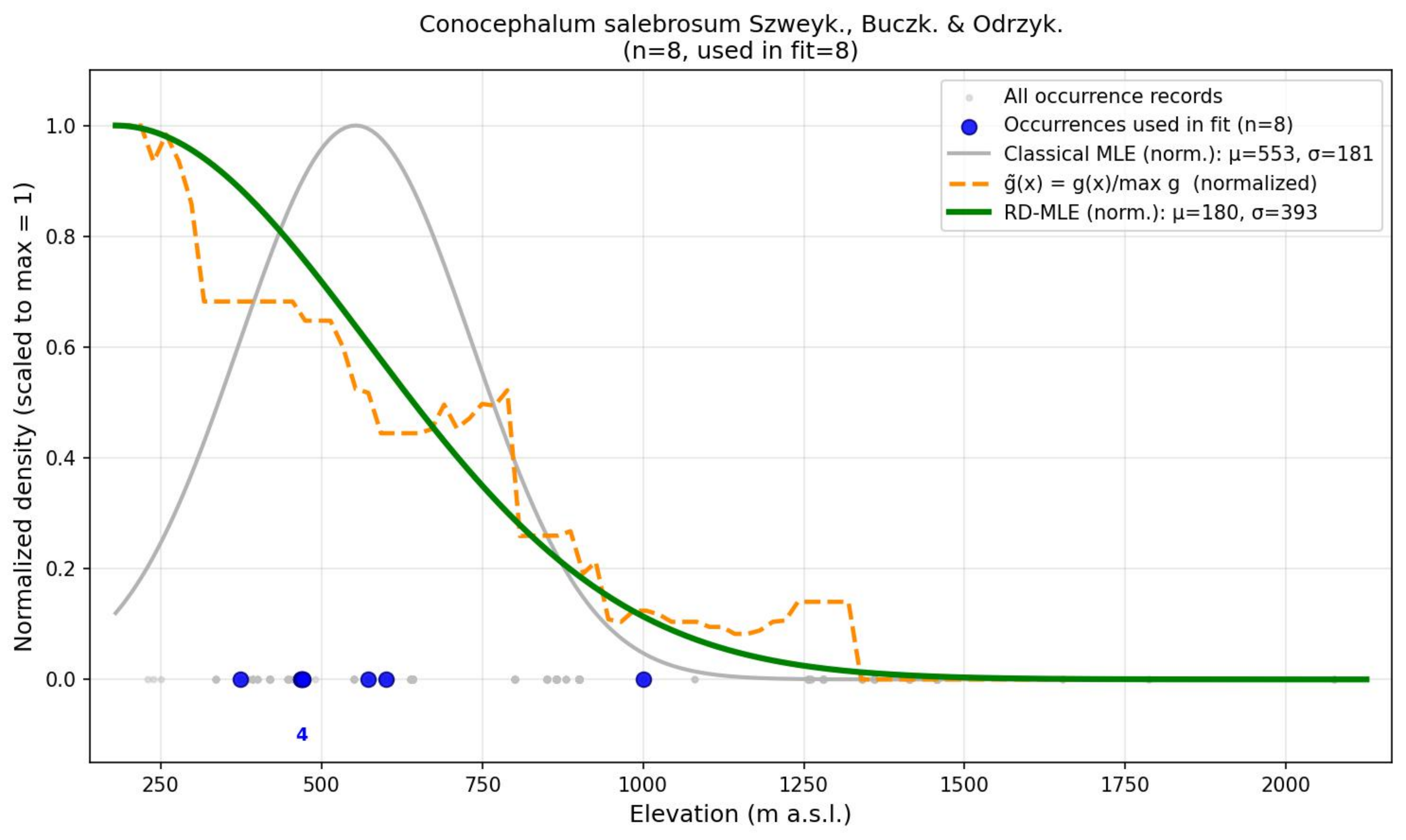

Conocephalum salebrosum Szweyk., Buczk. & Odrzyk.
(n=8, used in fit=8)
All occurrence records
Occurrences used in fit (n=8)
Classical MLE (norm.): μ=553, σ=181
g̃(x) = g(x)/max g (normalized)
RD-MLE (norm.): μ=180, σ=393
Normalized density (scaled to max = 1)
1.0
0.8
0.6
0.4
0.2
0.0
4
250
500
750
1000
1250
1500
1750
2000
Elevation (m a.s.l.)

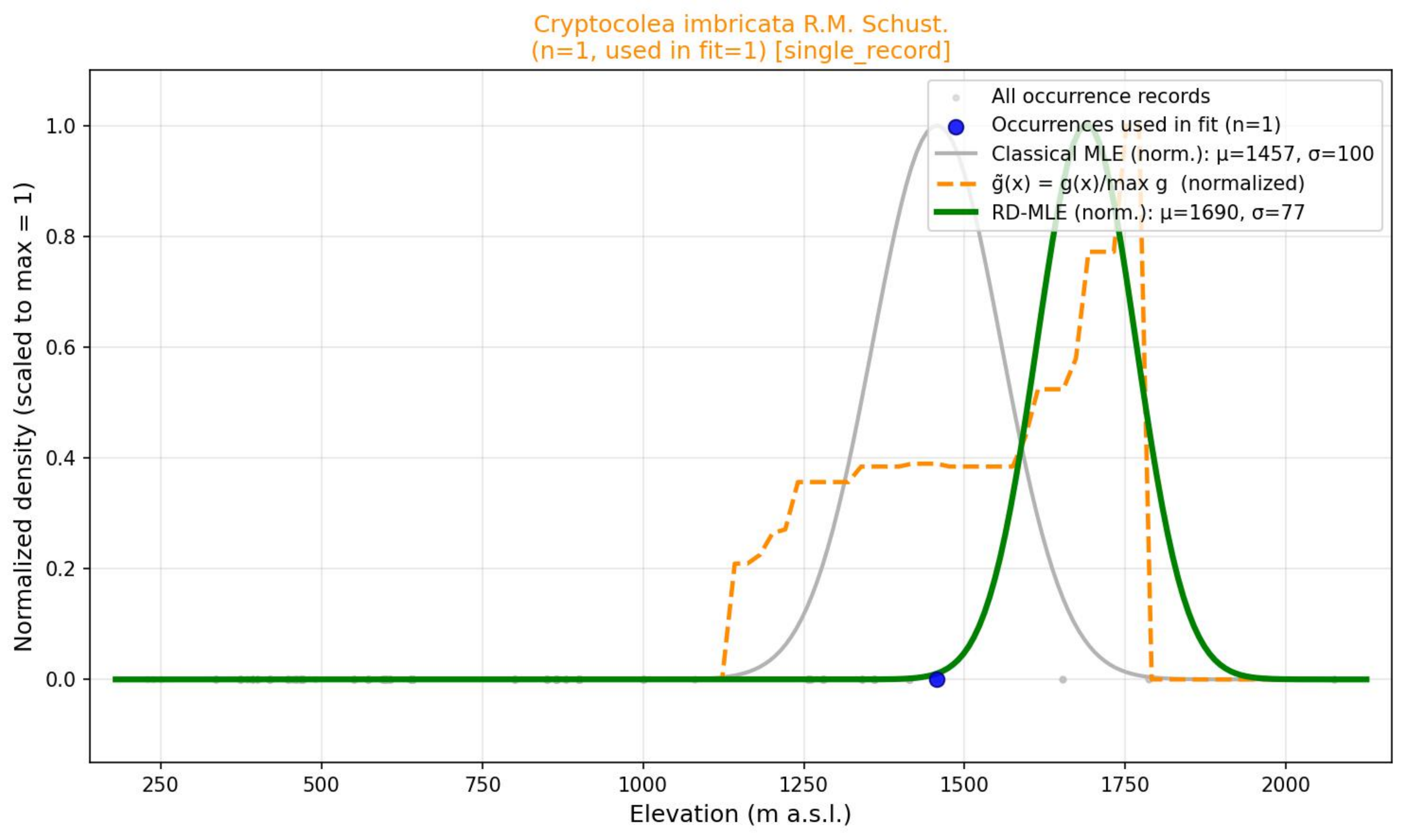

Cryptocolea imbricata R.M. Schust.
(n=1, used in fit=1) [single_record]
All occurrence records
Occurrences used in fit (n=1)
Classical MLE (norm.): μ=1457, σ=100
g̃(x) = g(x)/max g (normalized)
RD-MLE (norm.): μ=1690, σ=77
Normalized density (scaled to max = 1)
Elevation (m a.s.l.)
0.0
0.2
0.4
0.6
0.8
1.0
250
500
750
1000
1250
1500
1750
2000

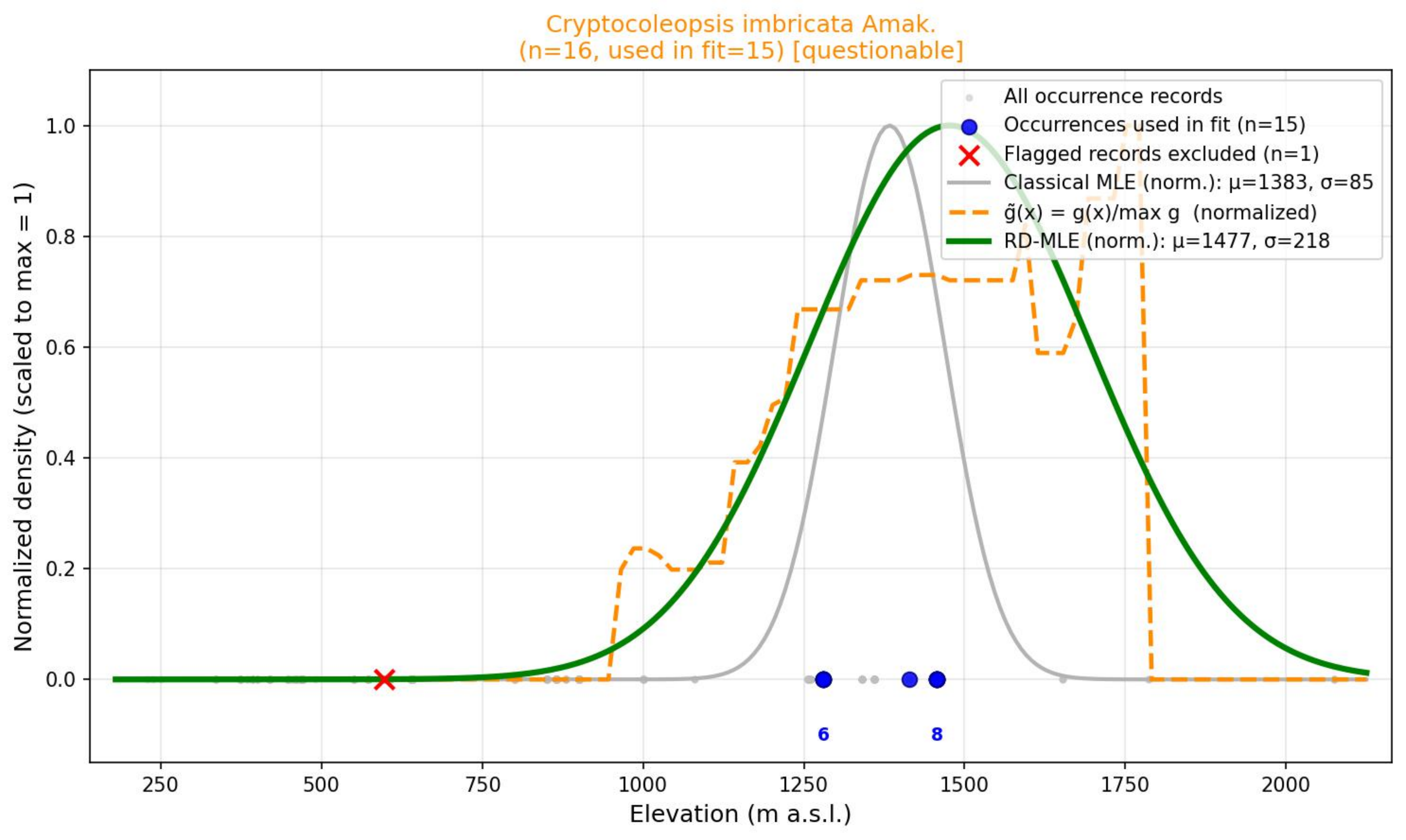

Cryptocoleopsis imbricata Amak.
(n=16, used in fit=15) [questionable]
All occurrence records
Occurrences used in fit (n=15)
Flagged records excluded (n=1)
Classical MLE (norm.): μ=1383, σ=85
g̃(x) = g(x)/max g (normalized)
RD-MLE (norm.): μ=1477, σ=218
Normalized density (scaled to max = 1)
1.0
0.8
0.6
0.4
0.2
0.0
6
8
250
500
750
1000
1250
1500
1750
2000
Elevation (m a.s.l.)

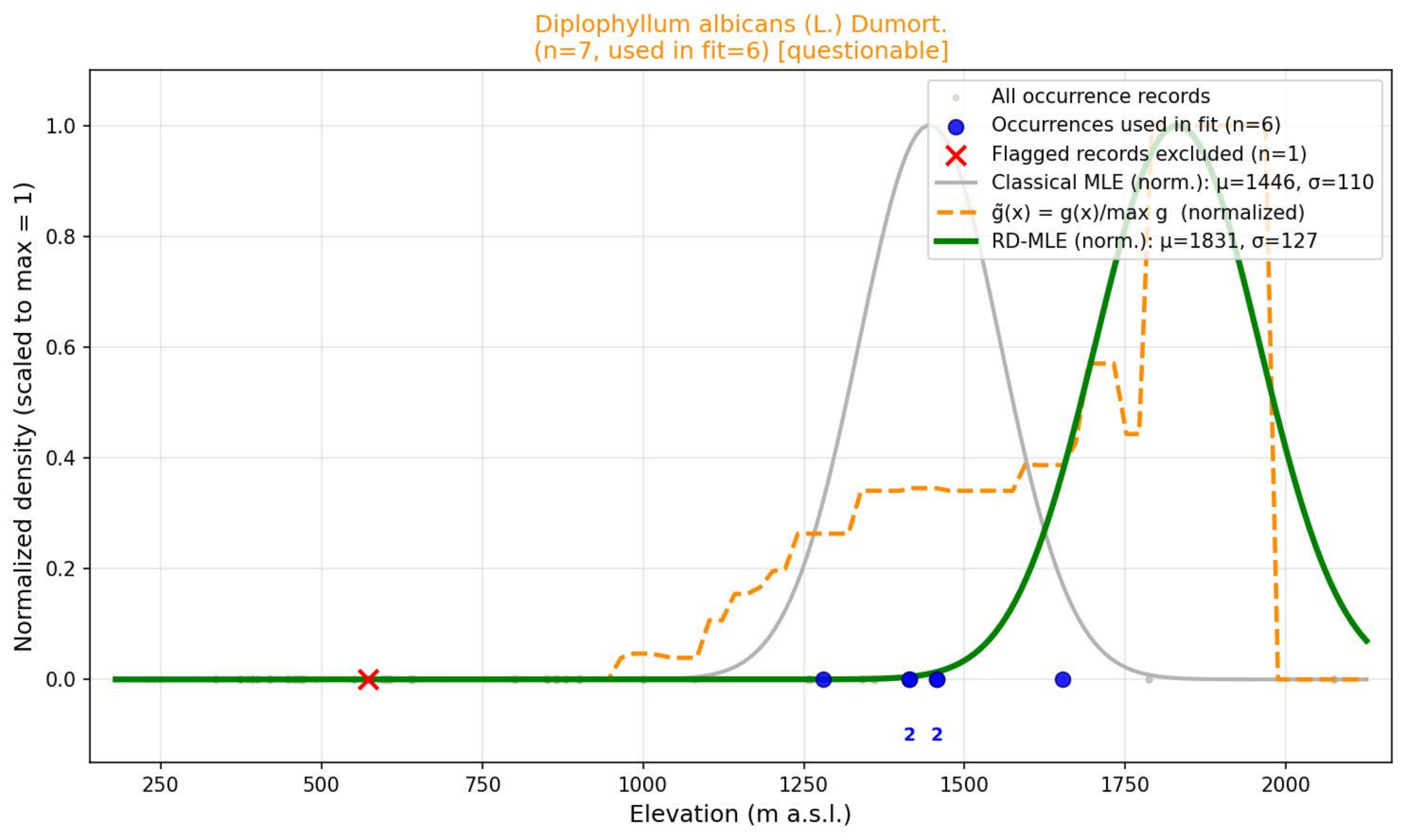

Diplophyllum albicans (L.) Dumort.
(n=7, used in fit=6) [questionable]
All occurrence records
Occurrences used in fit (n=6)
Flagged records excluded (n=1)
Classical MLE (norm.): μ=1446, σ=110
g̃(x) = g(x)/max g (normalized)
RD-MLE (norm.): μ=1831, σ=127
Normalized density (scaled to max = 1)
0.0
0.2
0.4
0.6
0.8
1.0
2
2
250
500
750
1000
1250
1500
1750
2000
Elevation (m a.s.l.)

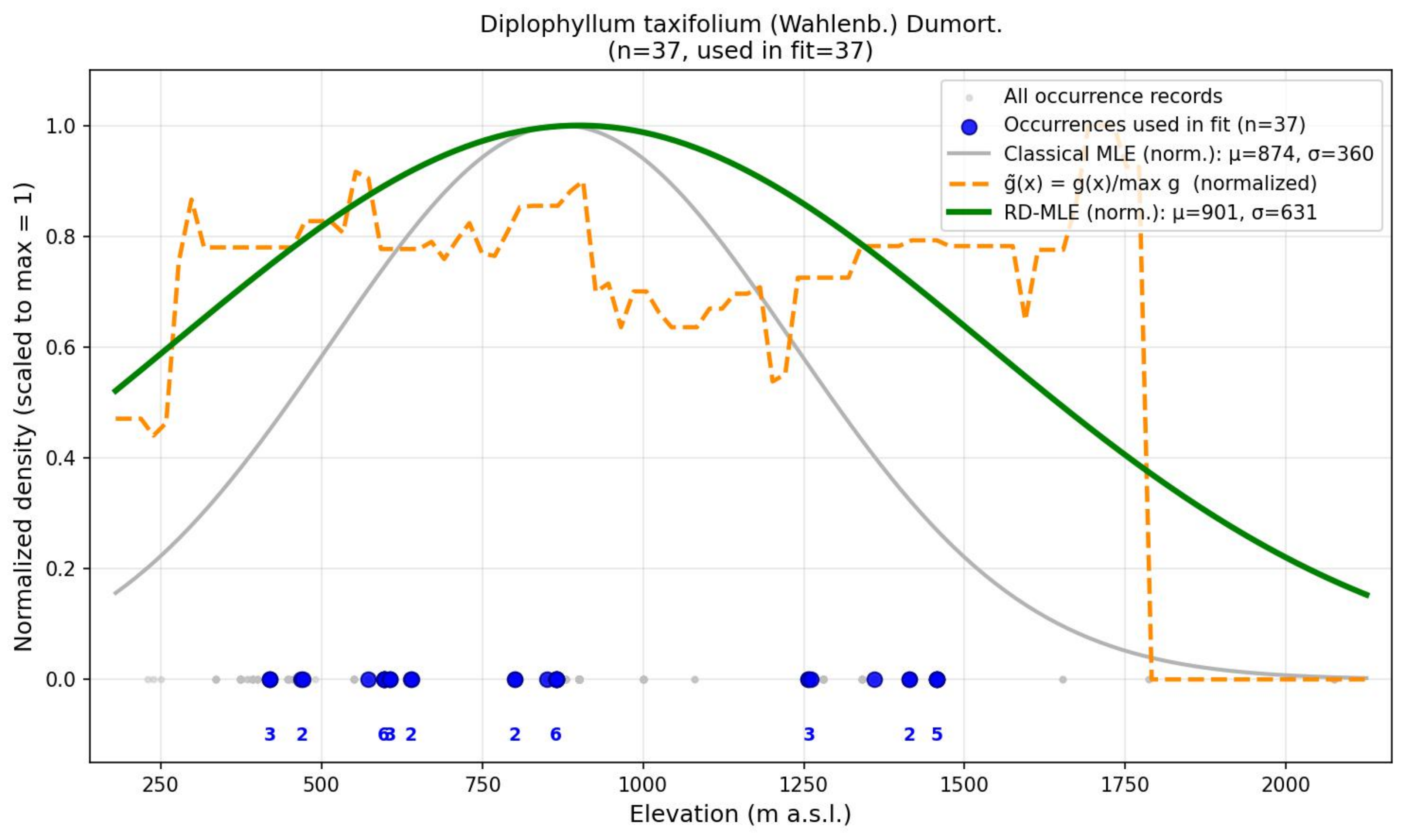

Diplophyllum taxifolium (Wahlenb.) Dumort.
(n=37, used in fit=37)
All occurrence records
Occurrences used in fit (n=37)
Classical MLE (norm.): μ=874, σ=360
g̃(x) = g(x)/max g (normalized)
RD-MLE (norm.): μ=901, σ=631
Normalized density (scaled to max = 1)
Elevation (m a.s.l.)
0.0
0.2
0.4
0.6
0.8
1.0
250
500
750
1000
1250
1500
1750
2000
3
2
6
3
2
2
6
3
2
5

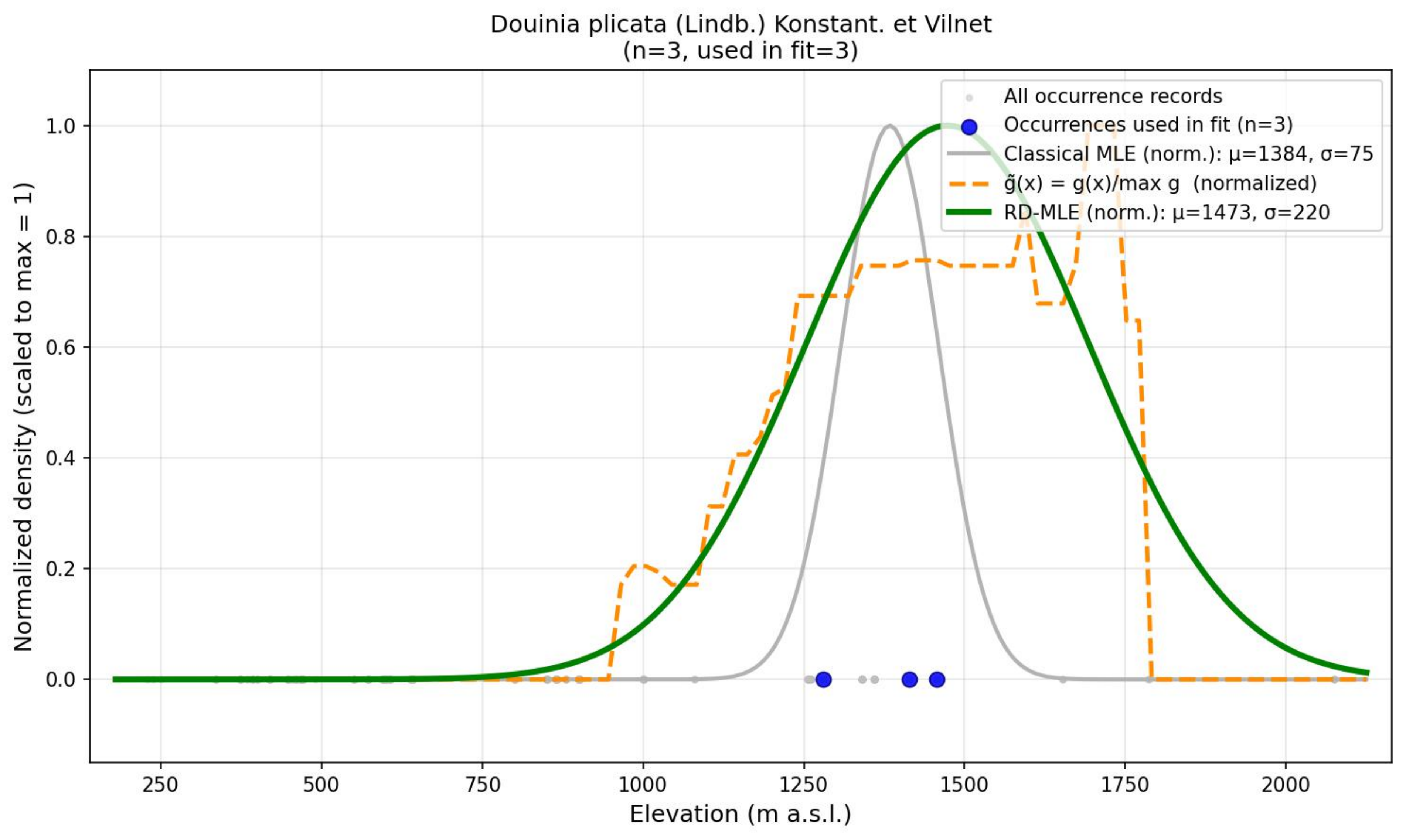

Douinia plicata (Lindb.) Konstant. et Vilnet
(n=3, used in fit=3)
All occurrence records
Occurrences used in fit (n=3)
Classical MLE (norm.): μ=1384, σ=75
g̃(x) = g(x)/max g (normalized)
RD-MLE (norm.): μ=1473, σ=220
Normalized density (scaled to max = 1)
Elevation (m a.s.l.)
1.0
0.8
0.6
0.4
0.2
0.0
250
500
750
1000
1250
1500
1750
2000

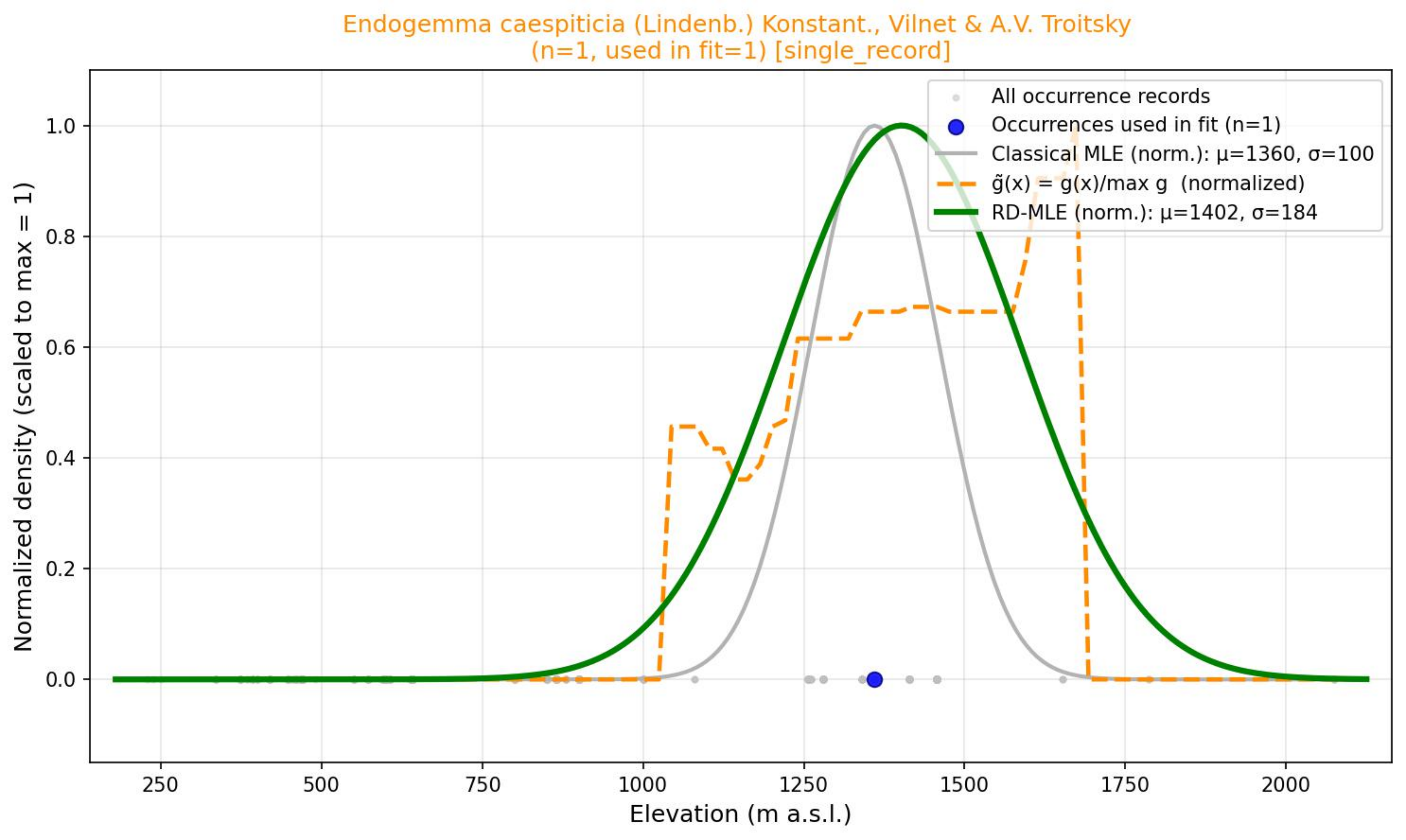

Endogemma caespiticia (Lindenb.) Konstant., Vilnet & A.V. Troitsky
(n=1, used in fit=1) [single_record]
All occurrence records
Occurrences used in fit (n=1)
Classical MLE (norm.): μ=1360, σ=100
g̃(x) = g(x)/max g (normalized)
RD-MLE (norm.): μ=1402, σ=184
Normalized density (scaled to max = 1)
Elevation (m a.s.l.)
0.0
0.2
0.4
0.6
0.8
1.0
250
500
750
1000
1250
1500
1750
2000

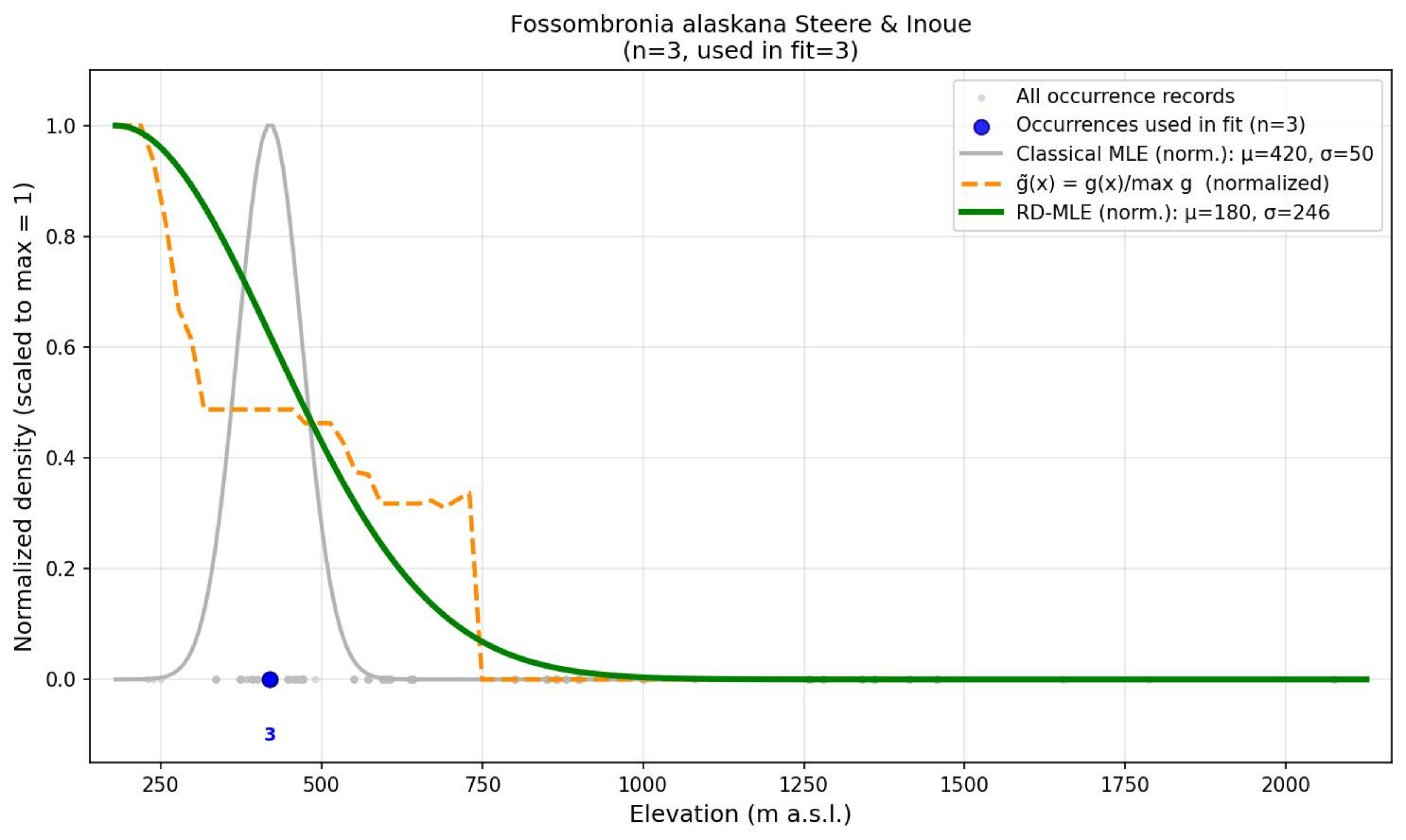

Fossombronia alaskana Steere & Inoue
(n=3, used in fit=3)
All occurrence records
Occurrences used in fit (n=3)
Classical MLE (norm.): μ=420, σ=50
g̃(x) = g(x)/max g (normalized)
RD-MLE (norm.): μ=180, σ=246
Normalized density (scaled to max = 1)
1.0
0.8
0.6
0.4
0.2
0.0
3
250
500
750
1000
1250
1500
1750
2000
Elevation (m a.s.l.)

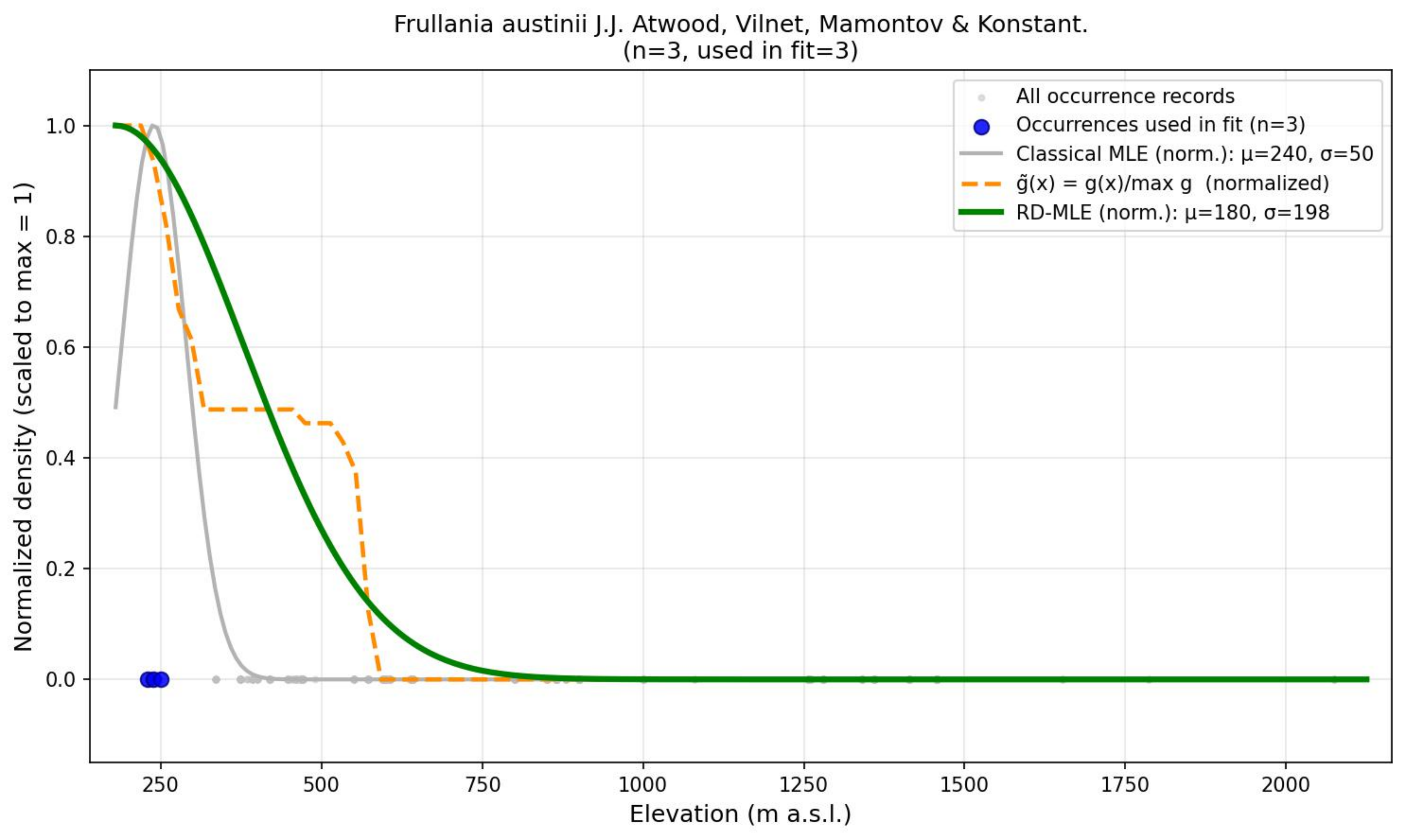
Frullania austinii J.J. Atwood, Vilnet, Mamontov & Konstant.
(n=3, used in fit=3)
All occurrence records
Occurrences used in fit (n=3)
Classical MLE (norm.): μ=240, σ=50
g̃(x) = g(x)/max g (normalized)
RD-MLE (norm.): μ=180, σ=198
Normalized density (scaled to max = 1)
Elevation (m a.s.l.)
1.0
0.8
0.6
0.4
0.2
0.0
250
500
750
1000
1250
1500
1750
2000

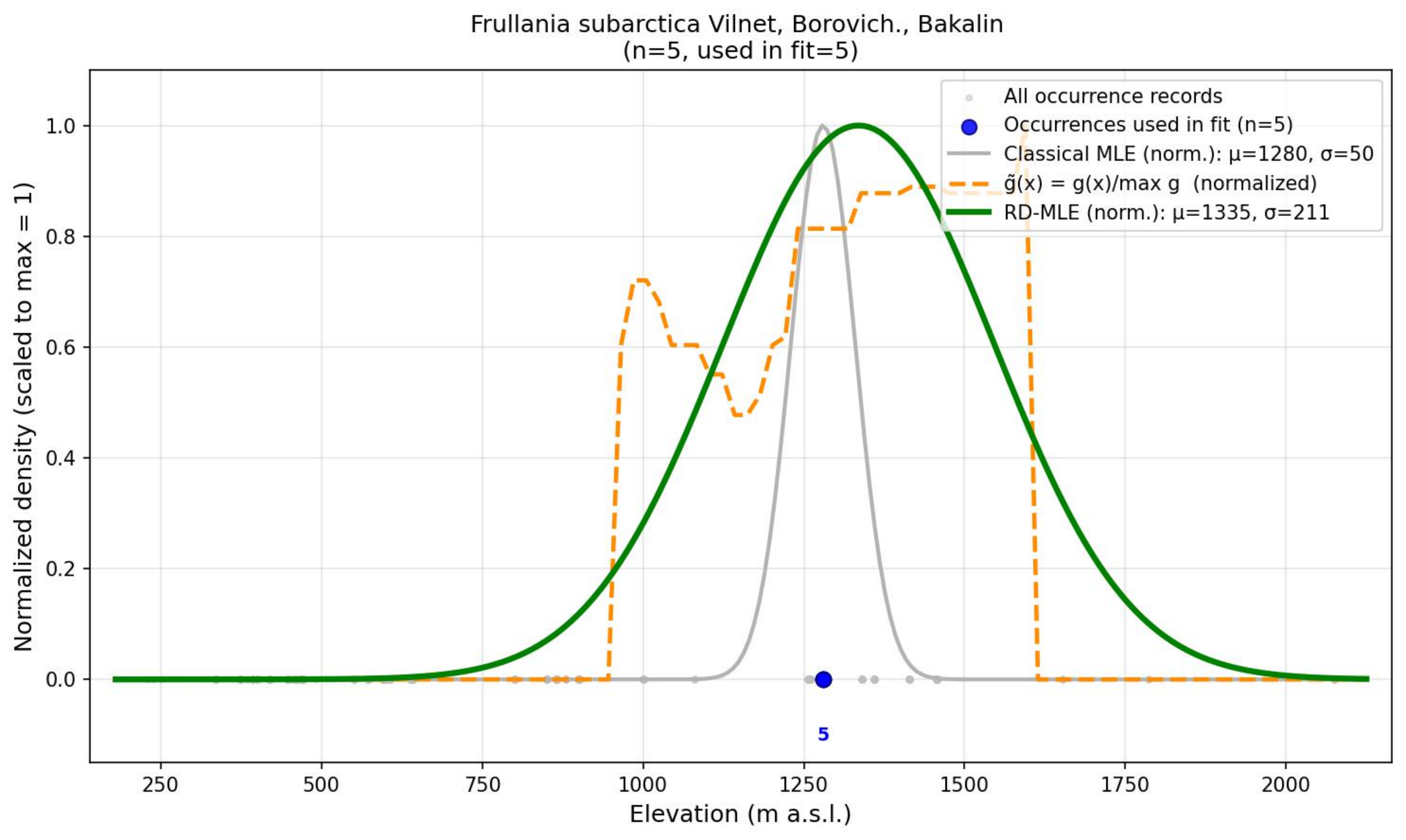
Frullania subarctica Vilnet, Borovich., Bakalin
(n=5, used in fit=5)
All occurrence records
Occurrences used in fit (n=5)
Classical MLE (norm.): μ=1280, σ=50
g̃(x) = g(x)/max g (normalized)
RD-MLE (norm.): μ=1335, σ=211
Normalized density (scaled to max = 1)
Elevation (m a.s.l.)
5
0.0
0.2
0.4
0.6
0.8
1.0
250
500
750
1000
1250
1500
1750
2000

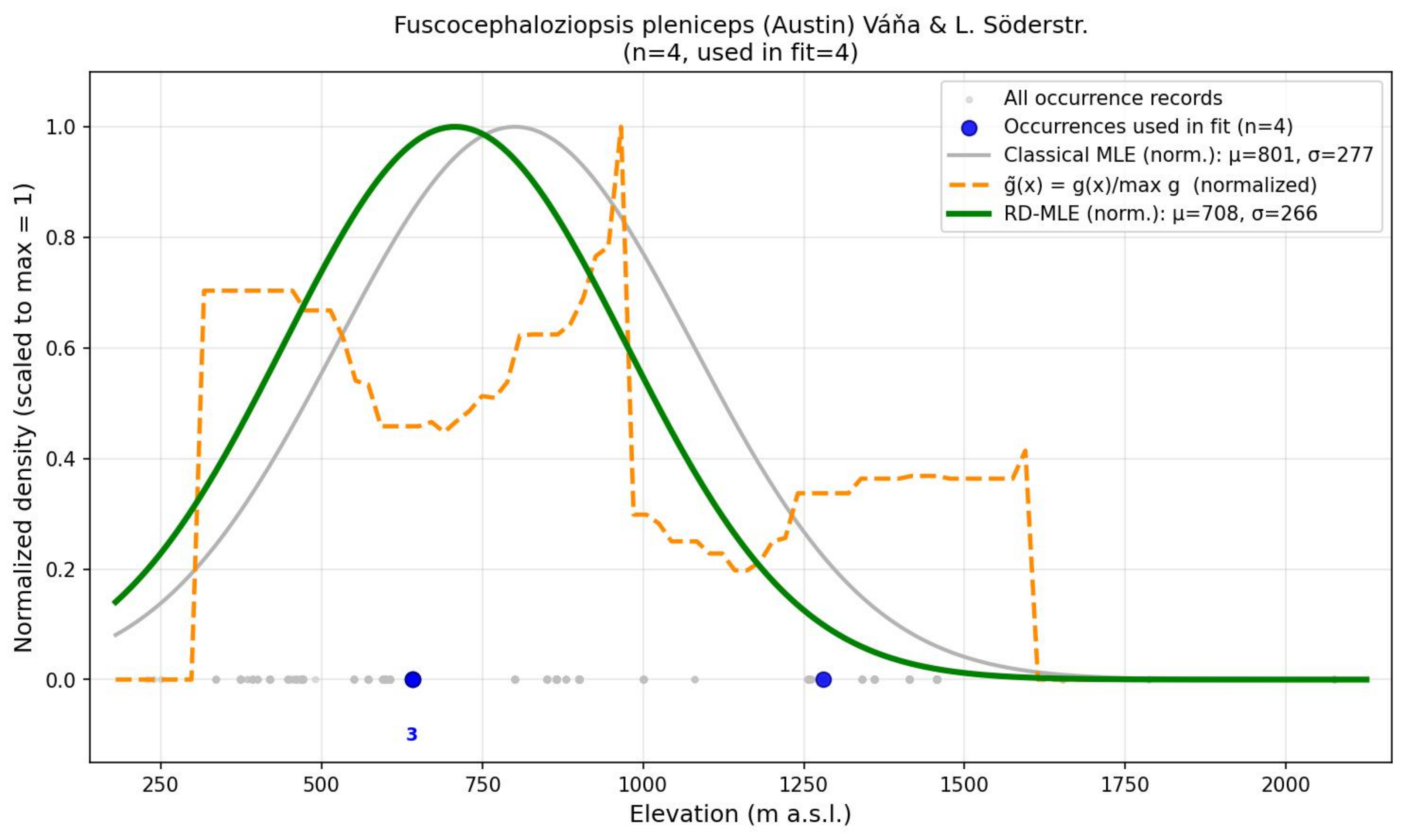

Fuscocephaloziopsis pleniceps (Austin) Váňa & L. Söderstr.
(n=4, used in fit=4)
All occurrence records
Occurrences used in fit (n=4)
Classical MLE (norm.): μ=801, σ=277
g̃(x) = g(x)/max g (normalized)
RD-MLE (norm.): μ=708, σ=266
Normalized density (scaled to max = 1)
0.0
0.2
0.4
0.6
0.8
1.0
3
250
500
750
1000
1250
1500
1750
2000
Elevation (m a.s.l.)

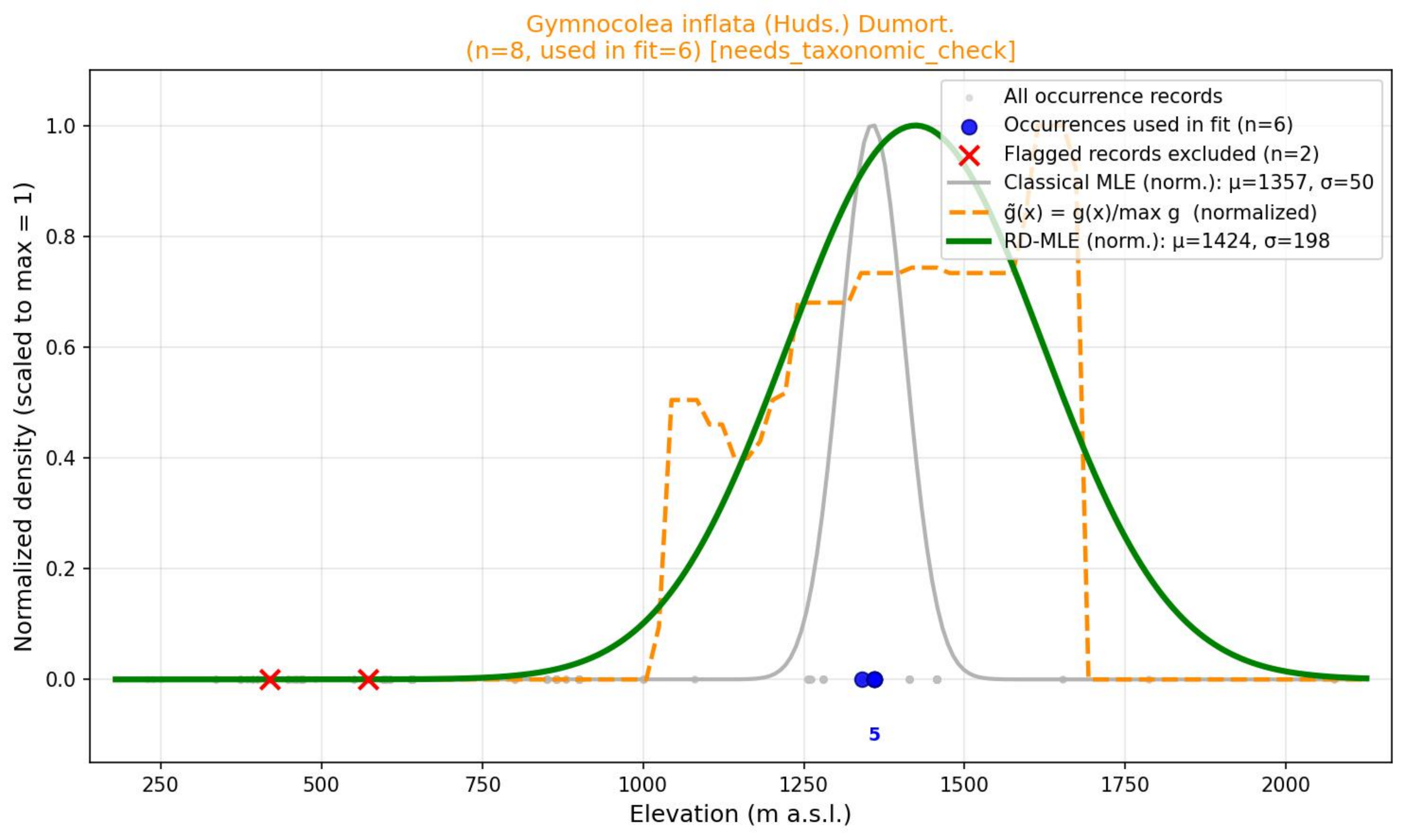

Gymnocolea inflata (Huds.) Dumort.
(n=8, used in fit=6) [needs_taxonomic_check]
All occurrence records
Occurrences used in fit (n=6)
Flagged records excluded (n=2)
Classical MLE (norm.): μ=1357, σ=50
g̃(x) = g(x)/max g (normalized)
RD-MLE (norm.): μ=1424, σ=198
Normalized density (scaled to max = 1)
Elevation (m a.s.l.)
5
1.0
0.8
0.6
0.4
0.2
0.0
250
500
750
1000
1250
1500
1750
2000

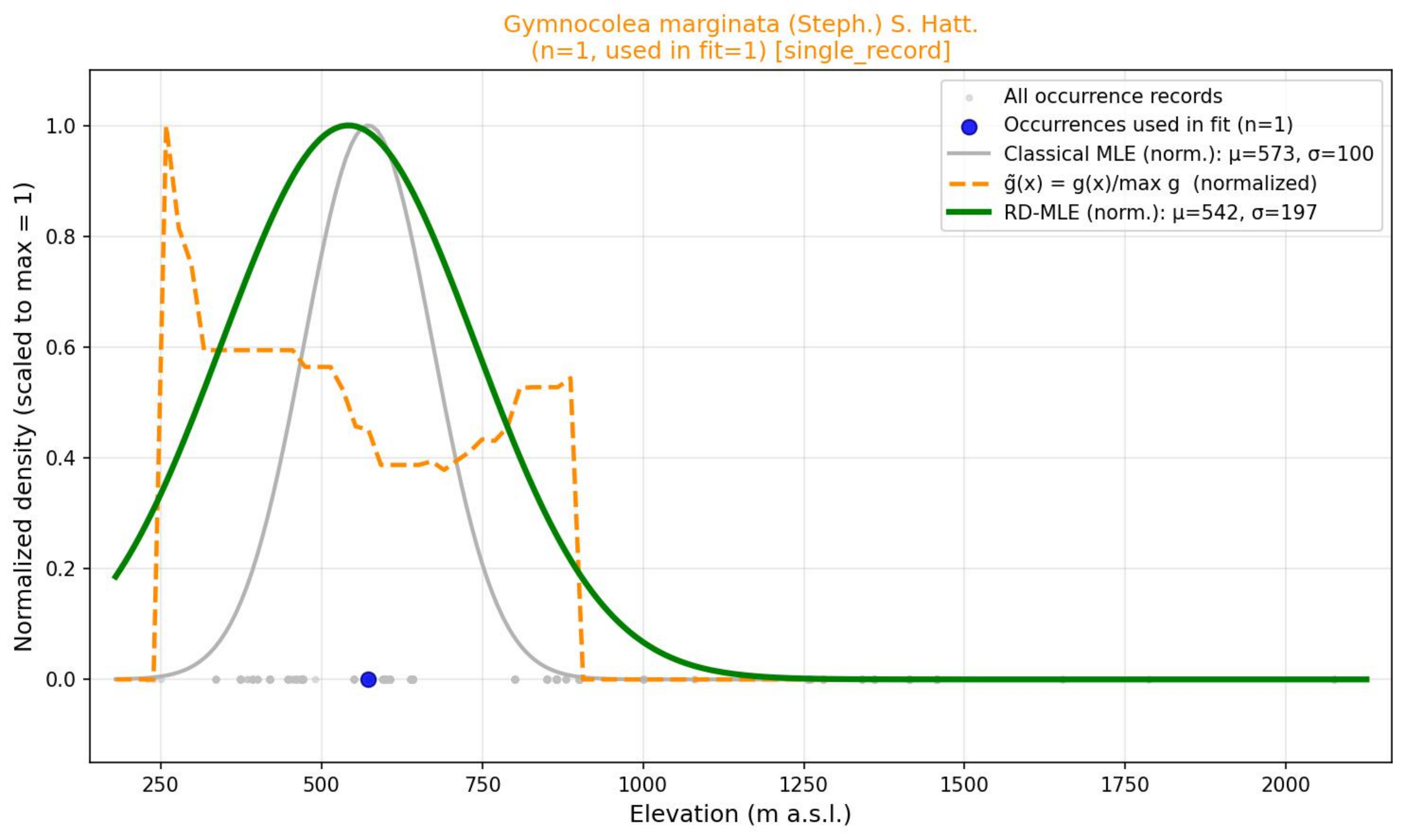

Gymnocolea marginata (Steph.) S. Hatt.
(n=1, used in fit=1) [single_record]
All occurrence records
Occurrences used in fit (n=1)
Classical MLE (norm.): μ=573, σ=100
g̃(x) = g(x)/max g (normalized)
RD-MLE (norm.): μ=542, σ=197
Normalized density (scaled to max = 1)
Elevation (m a.s.l.)
0.0
0.2
0.4
0.6
0.8
1.0
250
500
750
1000
1250
1500
1750
2000

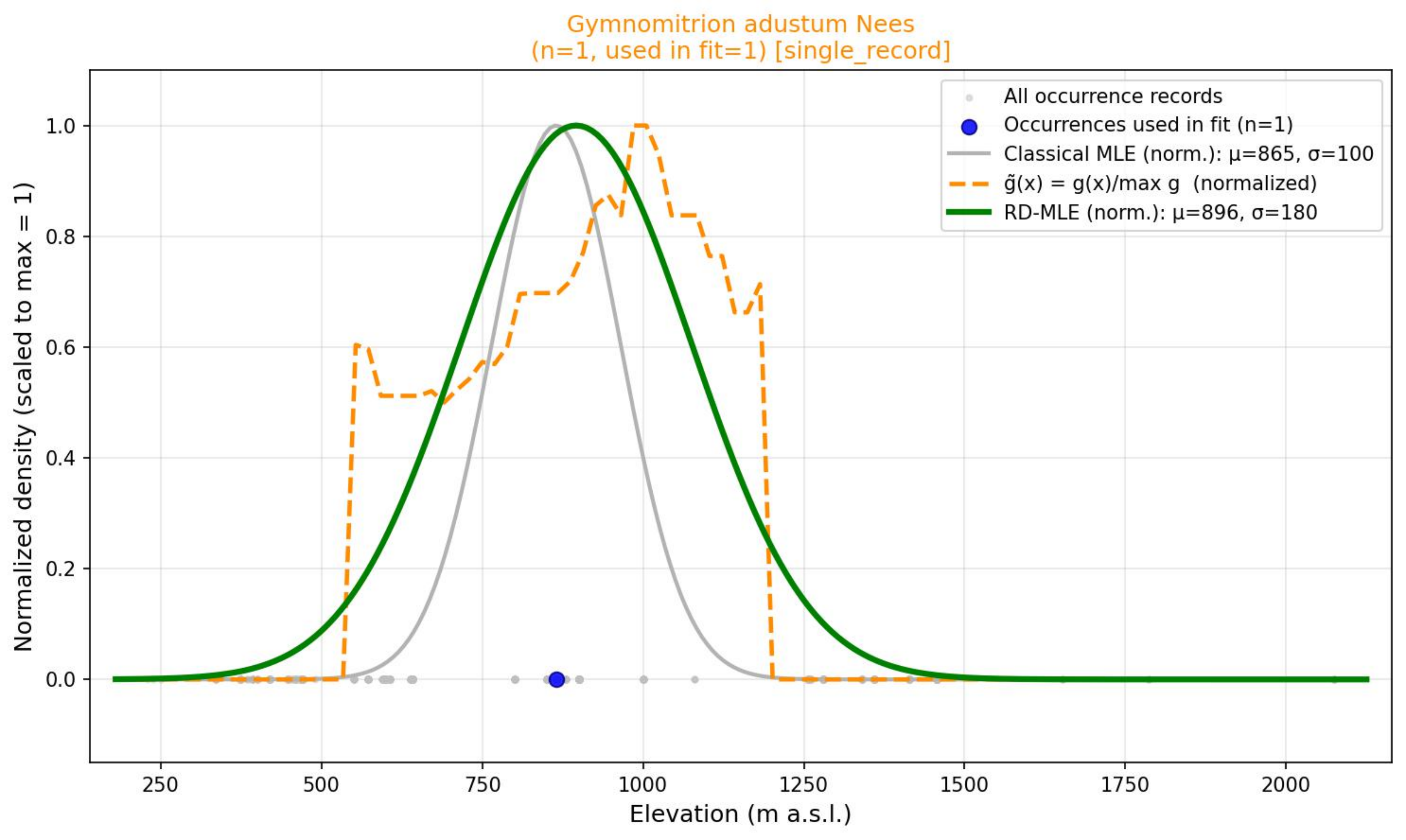
Gymnomitrion adustum Nees
(n=1, used in fit=1) [single_record]
All occurrence records
Occurrences used in fit (n=1)
Classical MLE (norm.): μ=865, σ=100
g̃(x) = g(x)/max g (normalized)
RD-MLE (norm.): μ=896, σ=180
Normalized density (scaled to max = 1)
Elevation (m a.s.l.)
0.0
0.2
0.4
0.6
0.8
1.0
250
500
750
1000
1250
1500
1750
2000

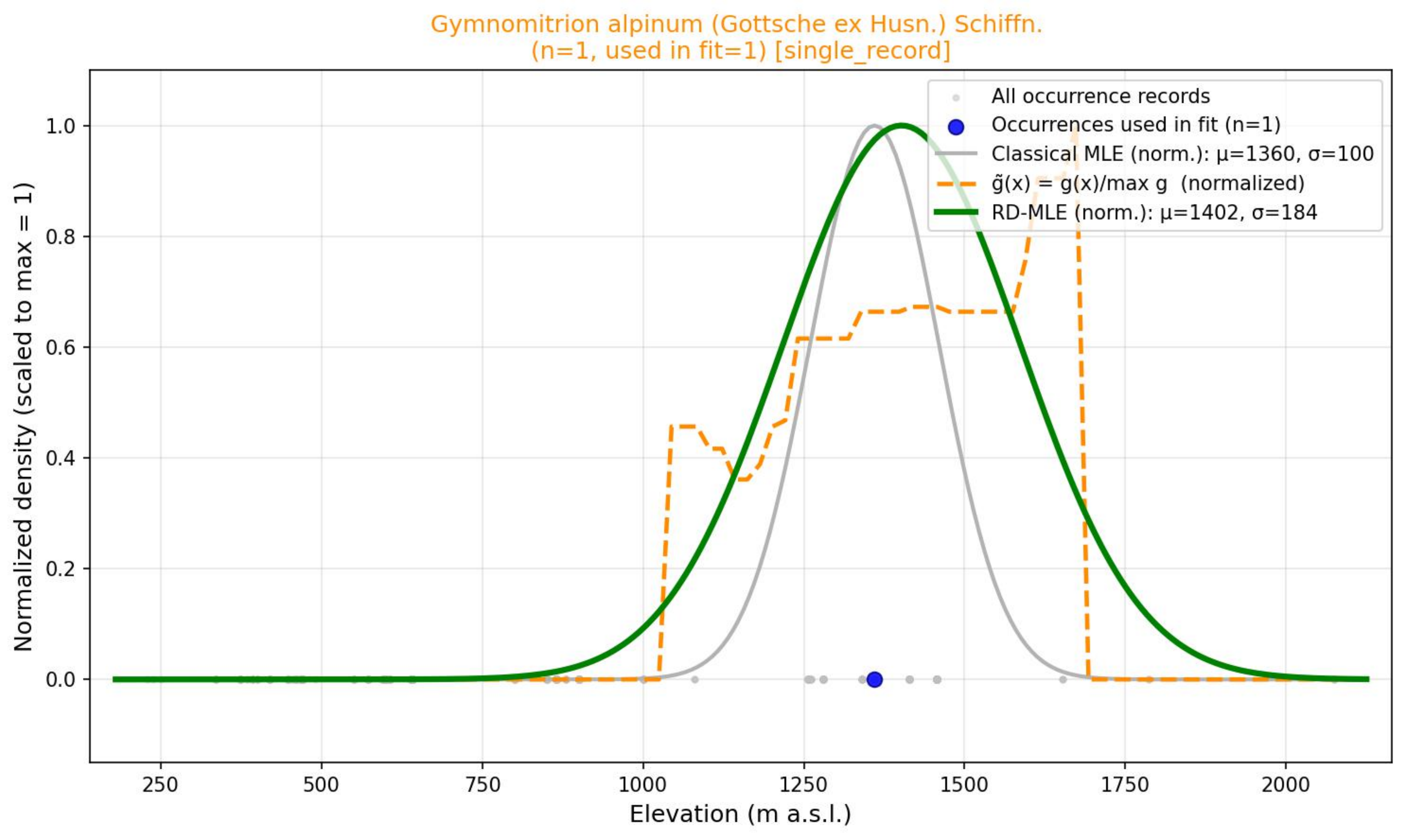
Gymnomitrion alpinum (Gottsche ex Husn.) Schiffn.
(n=1, used in fit=1) [single_record]
All occurrence records
Occurrences used in fit (n=1)
Classical MLE (norm.): μ=1360, σ=100
g̃(x) = g(x)/max g (normalized)
RD-MLE (norm.): μ=1402, σ=184
Normalized density (scaled to max = 1)
Elevation (m a.s.l.)
1.0
0.8
0.6
0.4
0.2
0.0
250
500
750
1000
1250
1500
1750
2000

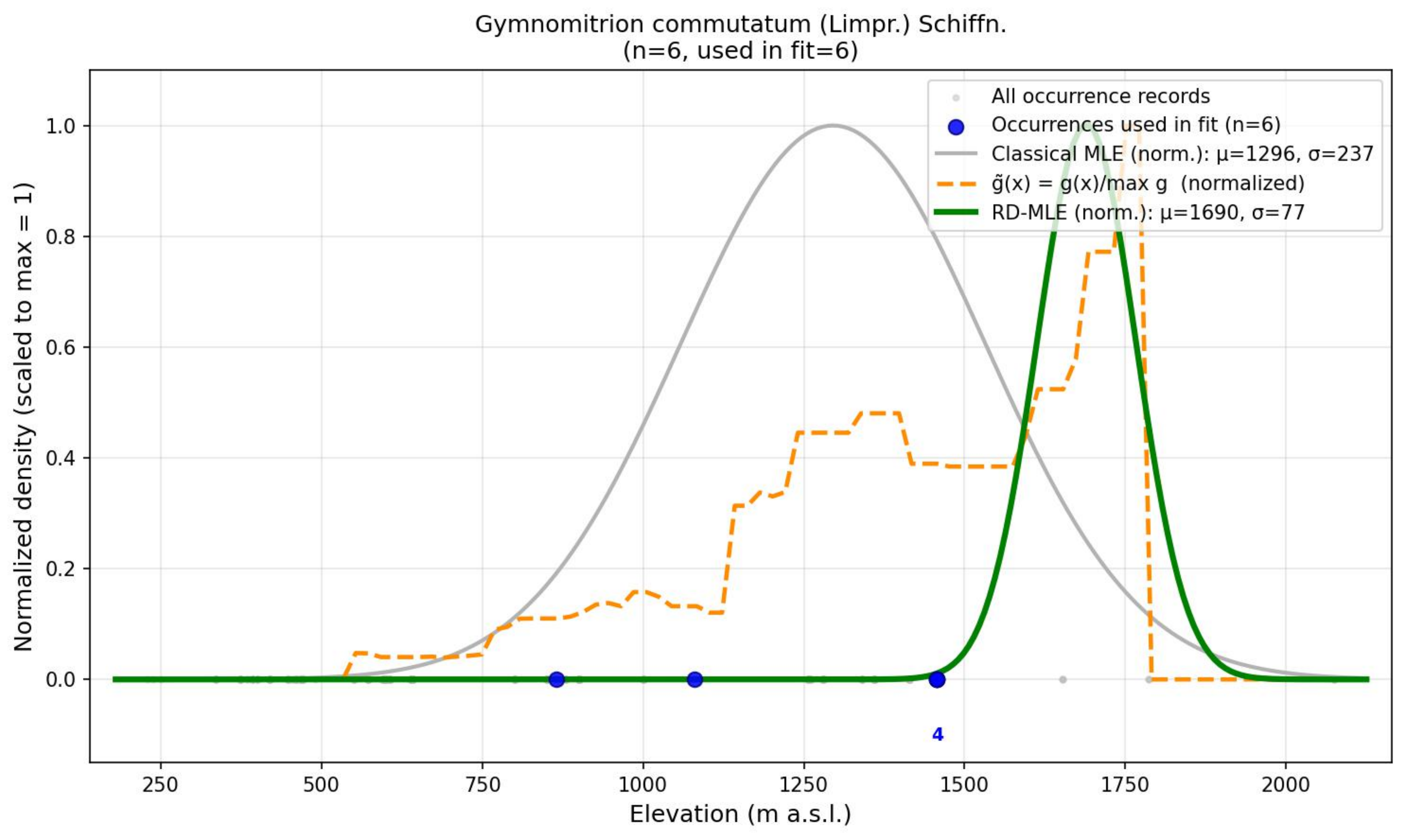

Gymnomitrion commutatum (Limpr.) Schiffn.
(n=6, used in fit=6)
All occurrence records
Occurrences used in fit (n=6)
Classical MLE (norm.): μ=1296, σ=237
g̃(x) = g(x)/max g (normalized)
RD-MLE (norm.): μ=1690, σ=77
Normalized density (scaled to max = 1)
1.0
0.8
0.6
0.4
0.2
0.0
4
250
500
750
1000
1250
1500
1750
2000
Elevation (m a.s.l.)

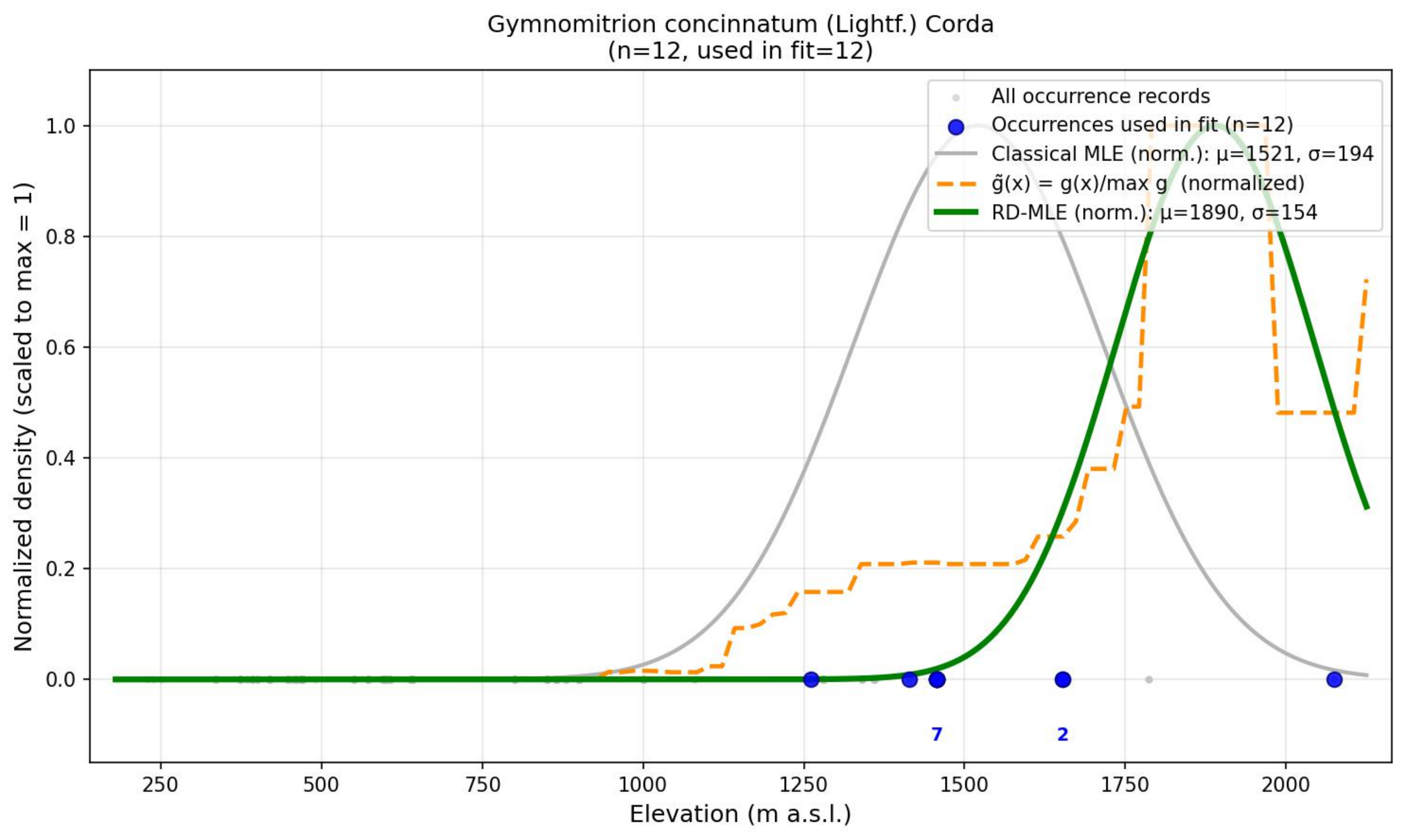

Gymnomitrion concinnatum (Lightf.) Corda
(n=12, used in fit=12)
All occurrence records
Occurrences used in fit (n=12)
Classical MLE (norm.): μ=1521, σ=194
g̃(x) = g(x)/max g (normalized)
RD-MLE (norm.): μ=1890, σ=154
Normalized density (scaled to max = 1)
Elevation (m a.s.l.)
0.0
0.2
0.4
0.6
0.8
1.0
250
500
750
1000
1250
1500
1750
2000
7
2

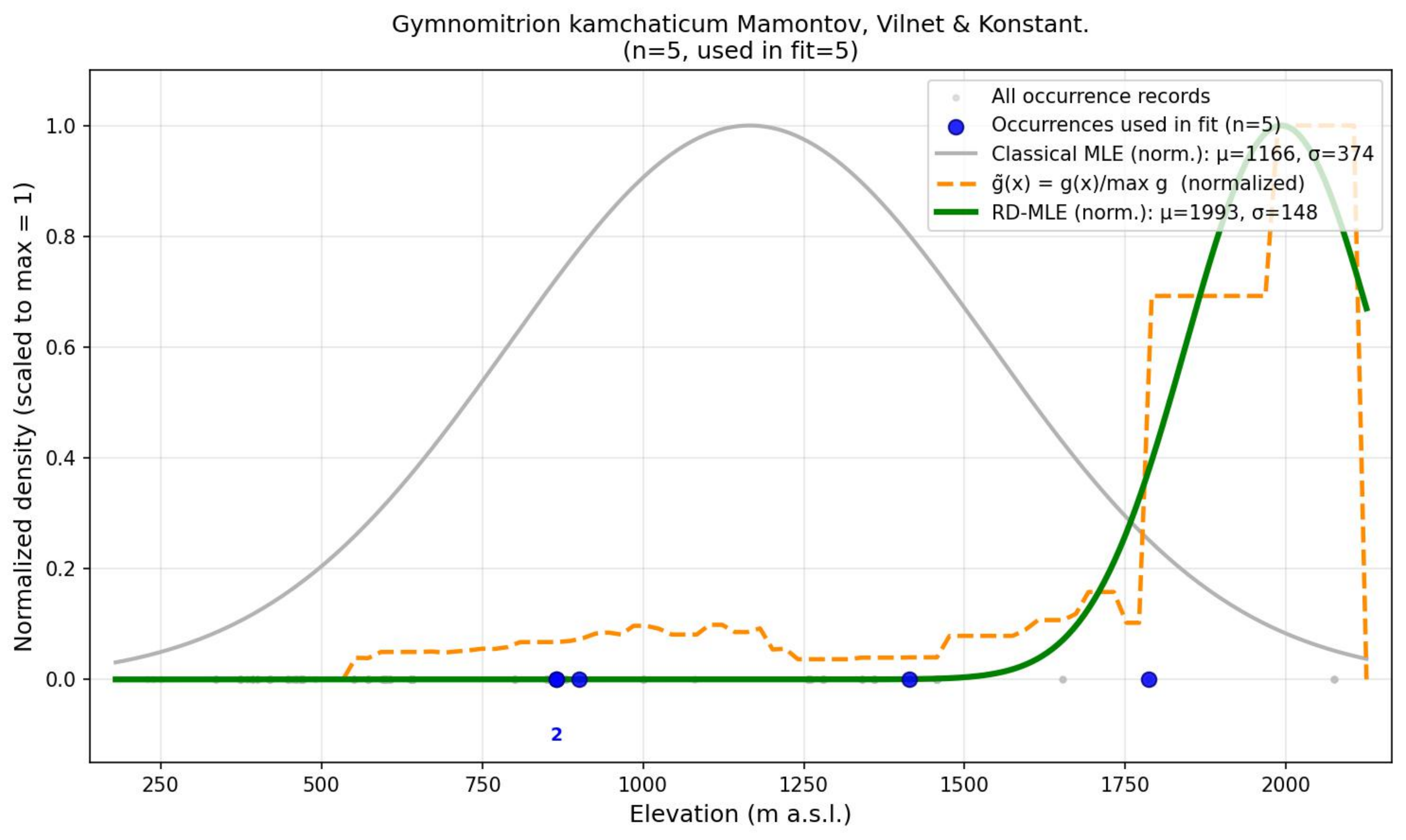
Gymnomitrion kamchaticum Mamontov, Vilnet & Konstant.
(n=5, used in fit=5)
All occurrence records
Occurrences used in fit (n=5)
Classical MLE (norm.): μ=1166, σ=374
g̃(x) = g(x)/max g (normalized)
RD-MLE (norm.): μ=1993, σ=148
Normalized density (scaled to max = 1)
Elevation (m a.s.l.)
2
0.0
0.2
0.4
0.6
0.8
1.0
250
500
750
1000
1250
1500
1750
2000

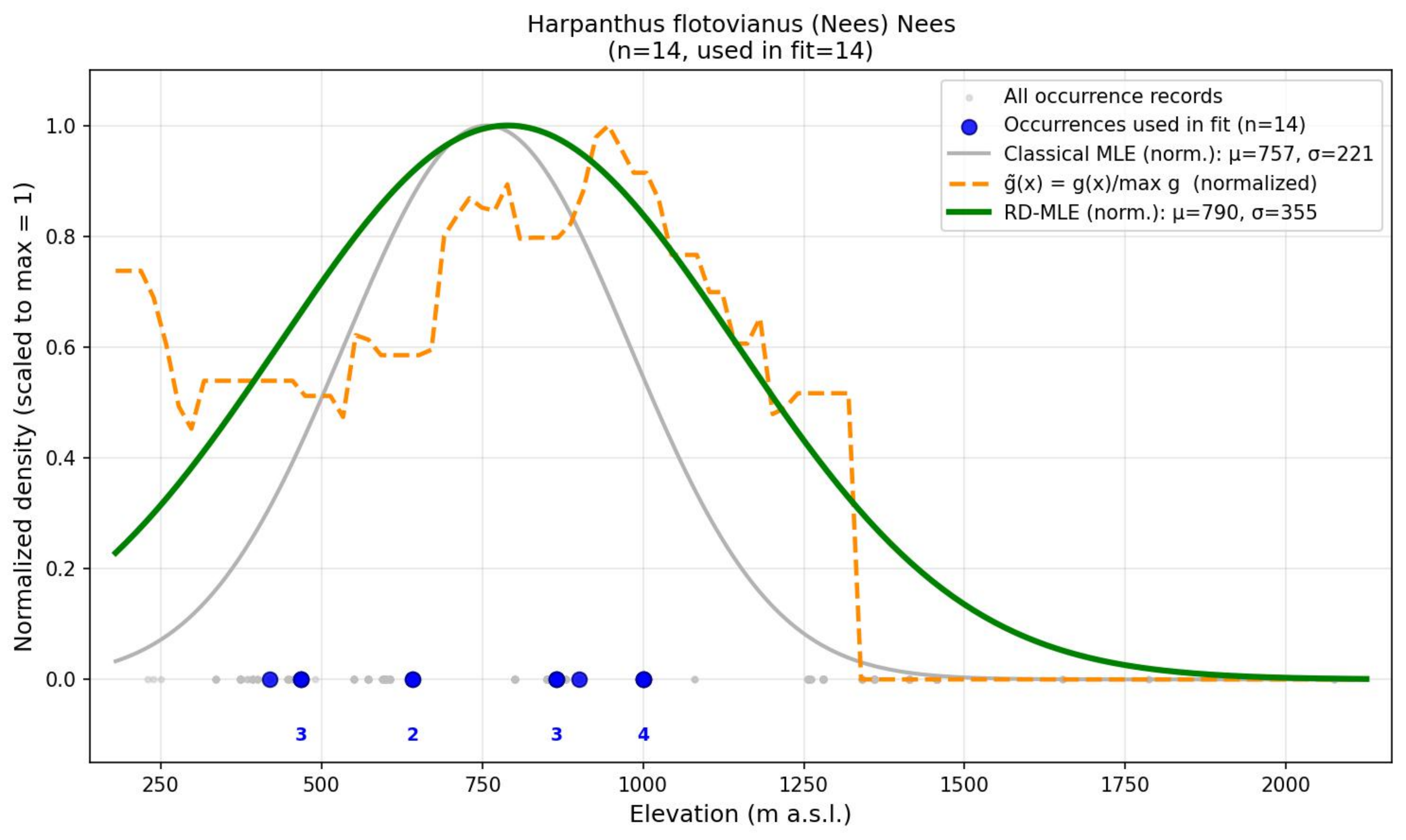
Harpanthus flotovianus (Nees) Nees
(n=14, used in fit=14)
All occurrence records
Occurrences used in fit (n=14)
Classical MLE (norm.): μ=757, σ=221
g̃(x) = g(x)/max g (normalized)
RD-MLE (norm.): μ=790, σ=355
Normalized density (scaled to max = 1)
Elevation (m a.s.l.)
1.0
0.8
0.6
0.4
0.2
0.0
250
500
750
1000
1250
1500
1750
2000
3
2
3
4

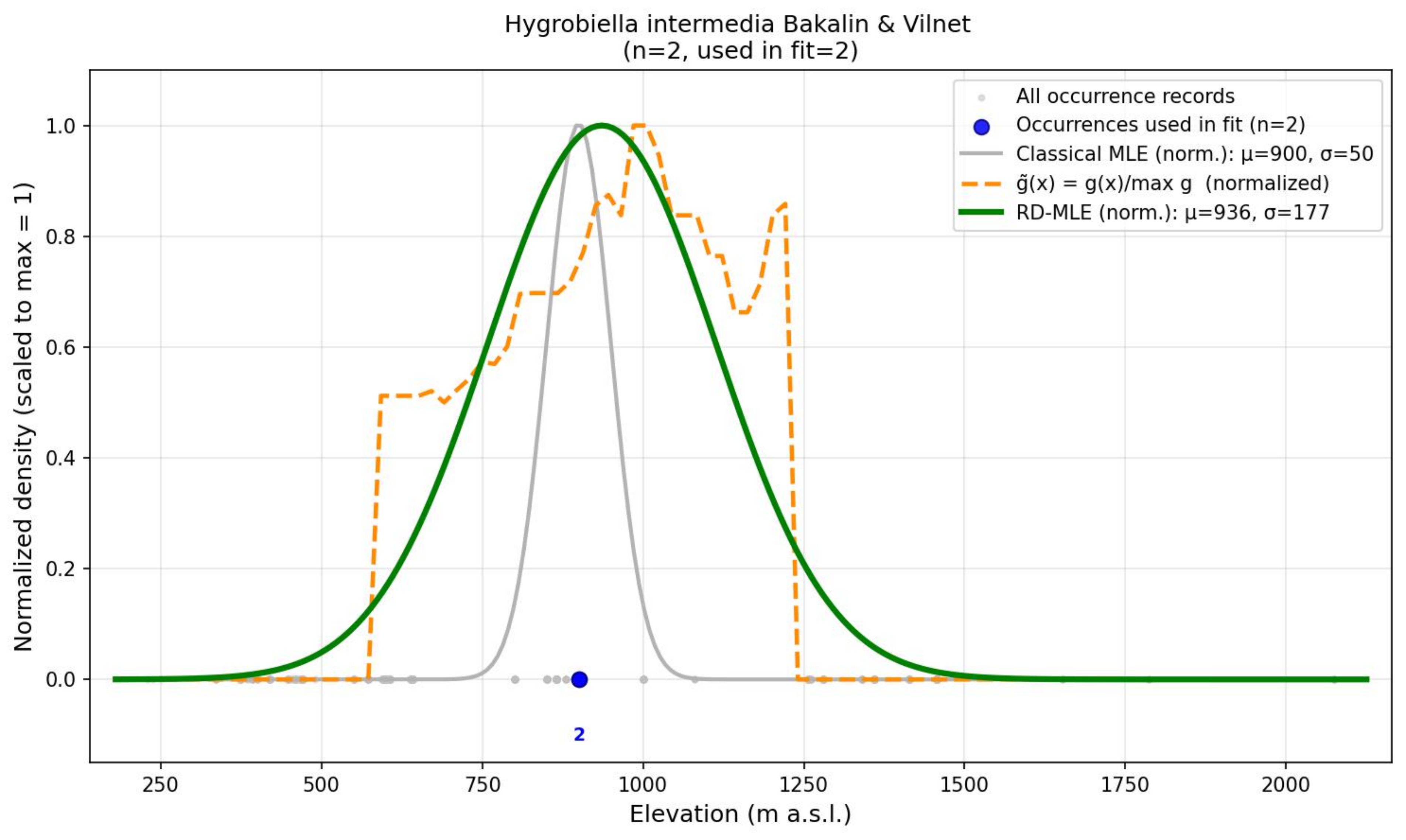
Hygrobiella intermedia Bakalin & Vilnet
(n=2, used in fit=2)
All occurrence records
Occurrences used in fit (n=2)
Classical MLE (norm.): μ=900, σ=50
g̃(x) = g(x)/max g (normalized)
RD-MLE (norm.): μ=936, σ=177
Normalized density (scaled to max = 1)
Elevation (m a.s.l.)
1.0
0.8
0.6
0.4
0.2
0.0
250
500
750
1000
1250
1500
1750
2000
2

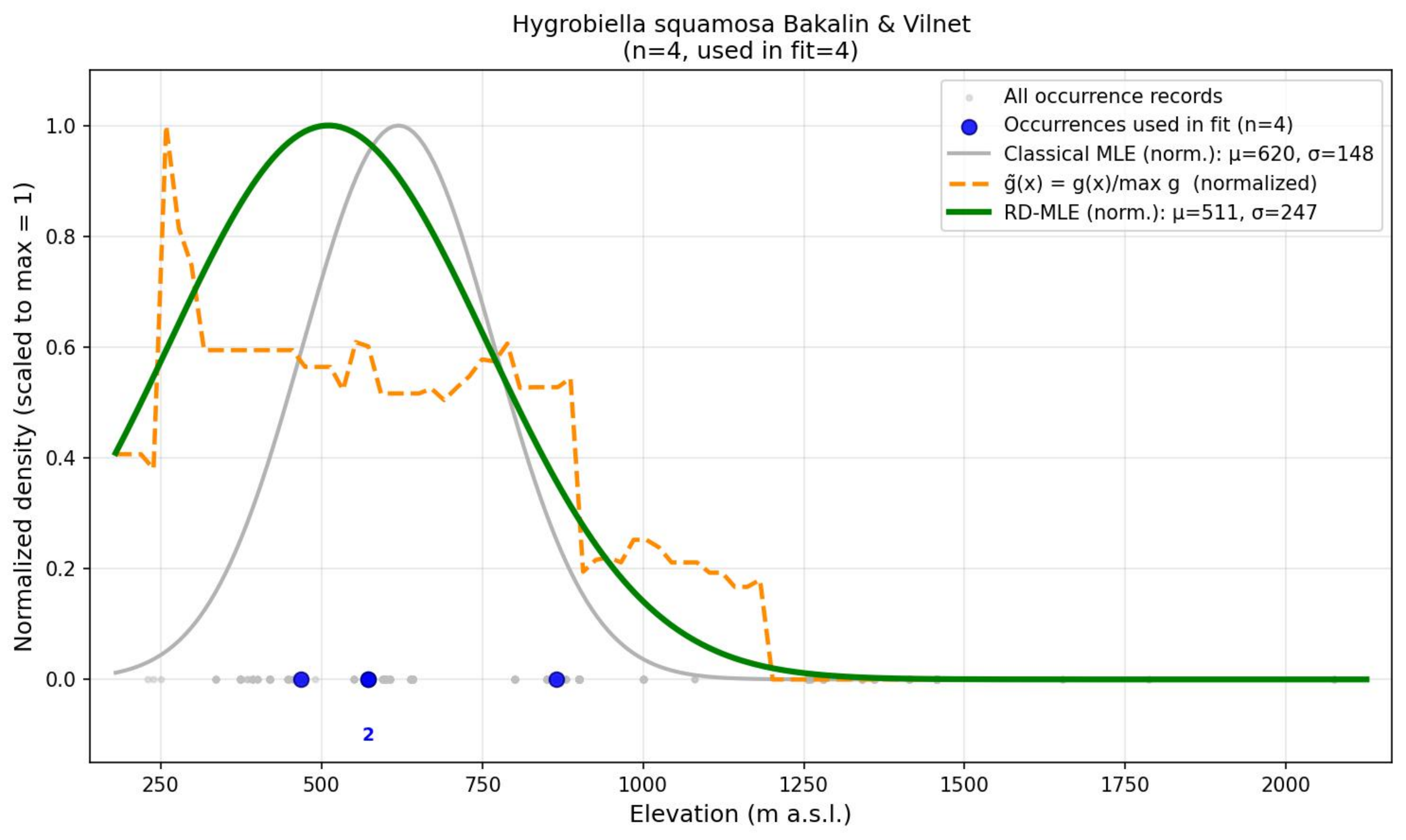

Hygrobiella squamosa Bakalin & Vilnet
(n=4, used in fit=4)
All occurrence records
Occurrences used in fit (n=4)
Classical MLE (norm.): μ=620, σ=148
g̃(x) = g(x)/max g (normalized)
RD-MLE (norm.): μ=511, σ=247
Normalized density (scaled to max = 1)
Elevation (m a.s.l.)
0.0
0.2
0.4
0.6
0.8
1.0
250
500
750
1000
1250
1500
1750
2000
2

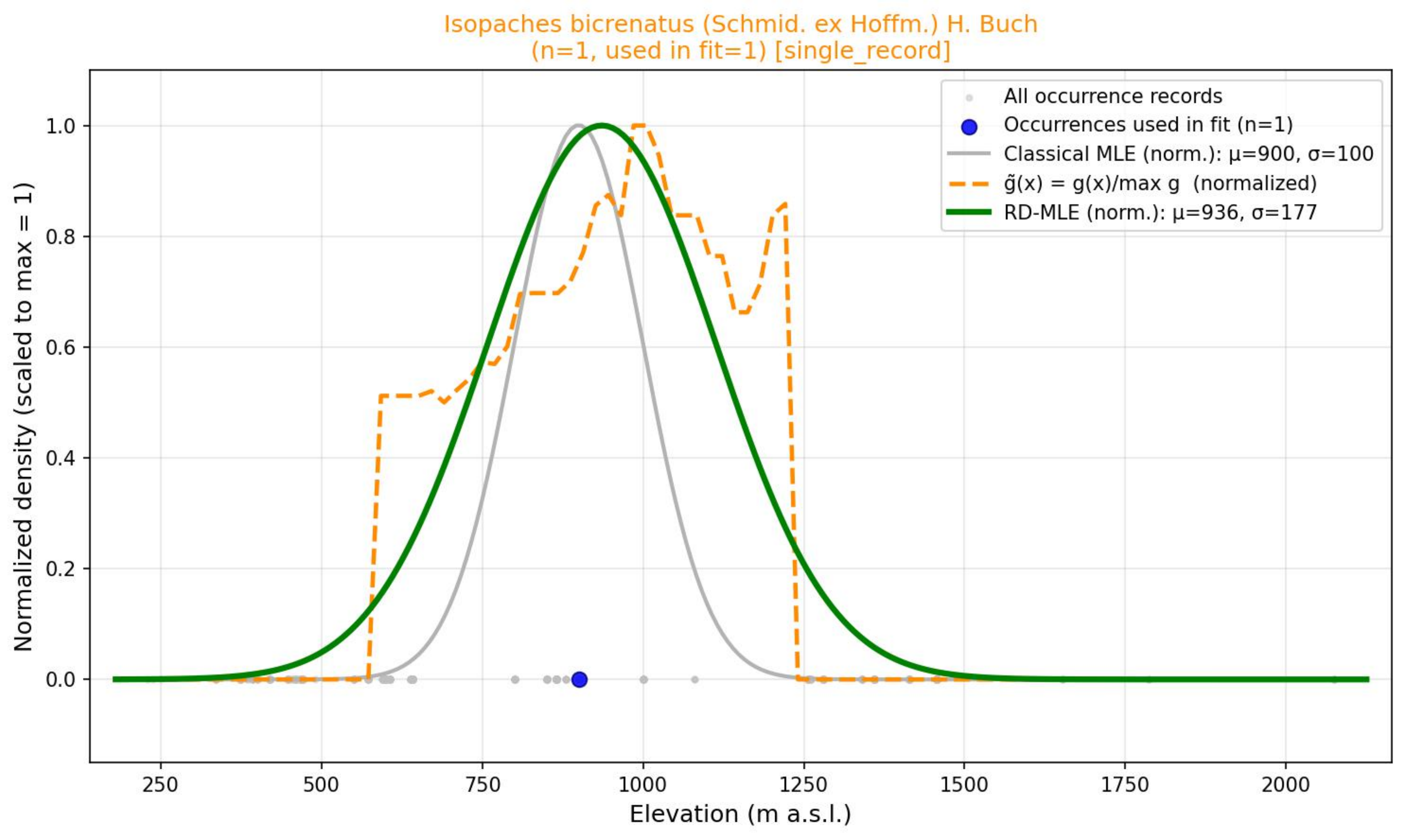
Isopaches bicrenatus (Schmid. ex Hoffm.) H. Buch
(n=1, used in fit=1) [single_record]
All occurrence records
Occurrences used in fit (n=1)
Classical MLE (norm.): μ=900, σ=100
g̃(x) = g(x)/max g (normalized)
RD-MLE (norm.): μ=936, σ=177
Normalized density (scaled to max = 1)
Elevation (m a.s.l.)
0.0
0.2
0.4
0.6
0.8
1.0
250
500
750
1000
1250
1500
1750
2000

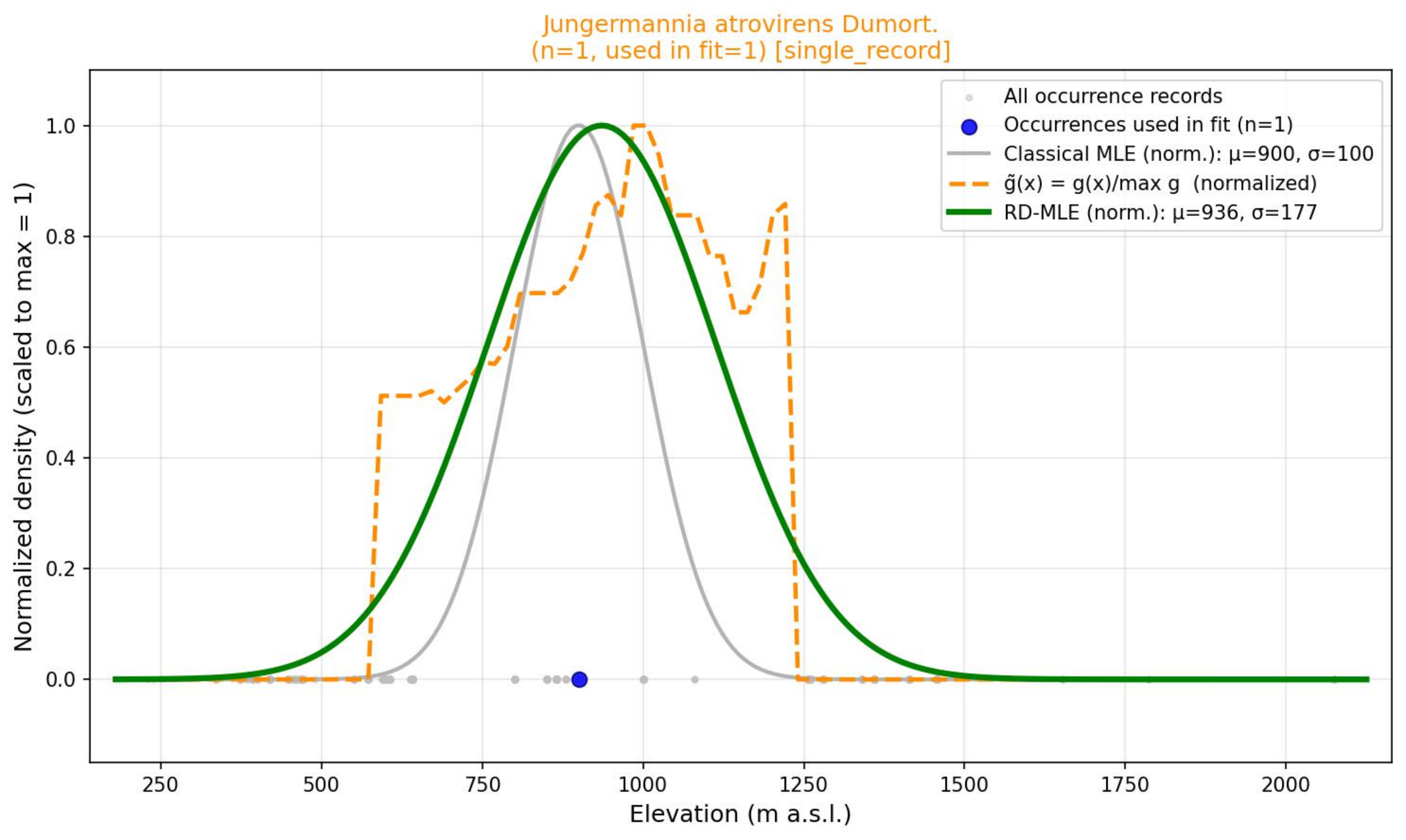
Jungermannia atrovirens Dumort.
(n=1, used in fit=1) [single_record]
All occurrence records
Occurrences used in fit (n=1)
Classical MLE (norm.): μ=900, σ=100
g̃(x) = g(x)/max g (normalized)
RD-MLE (norm.): μ=936, σ=177
Normalized density (scaled to max = 1)
Elevation (m a.s.l.)
0.0
0.2
0.4
0.6
0.8
1.0
250
500
750
1000
1250
1500
1750
2000

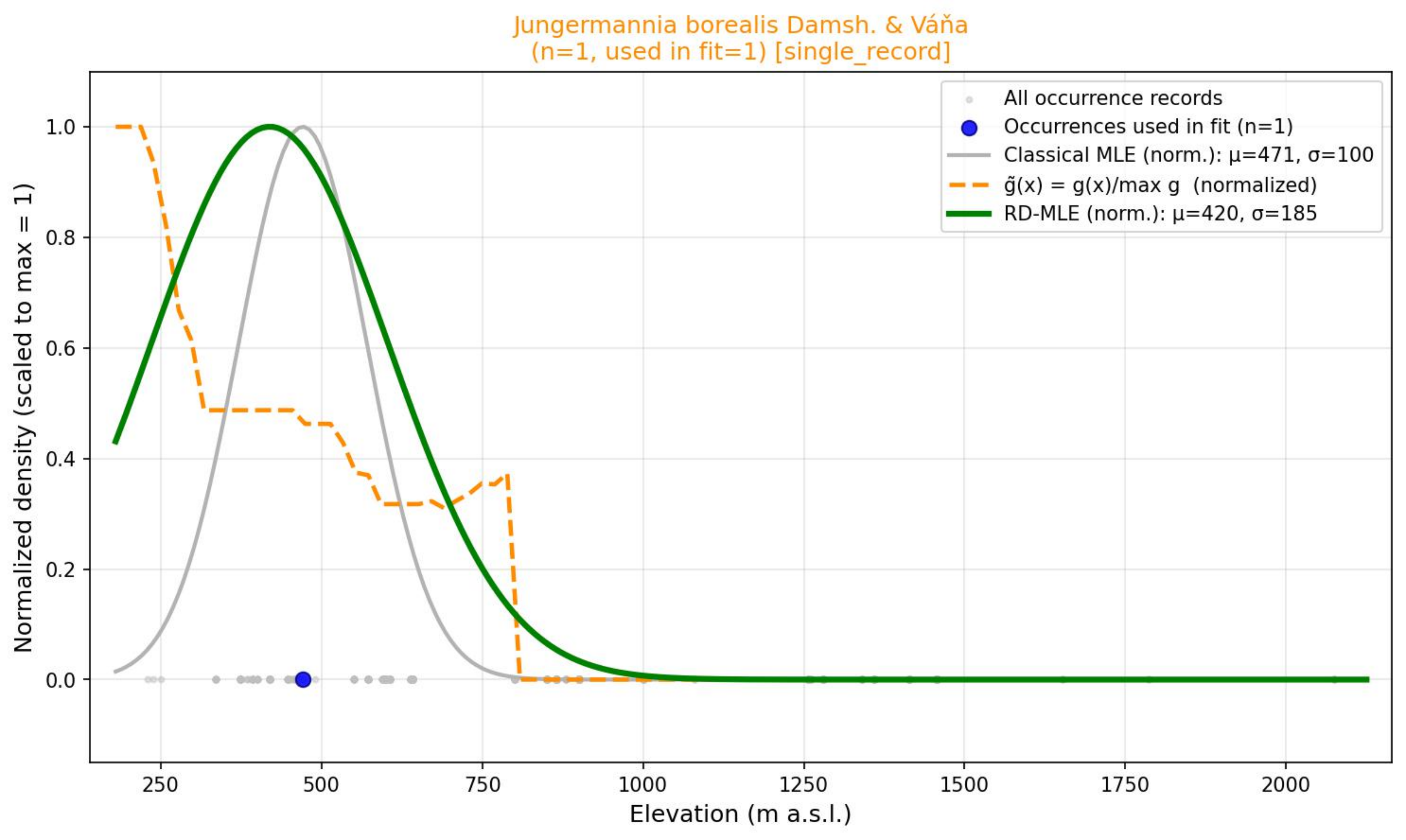
Jungermannia borealis Damsh. & Váňa
(n=1, used in fit=1) [single_record]
All occurrence records
Occurrences used in fit (n=1)
Classical MLE (norm.): μ=471, σ=100
g̃(x) = g(x)/max g (normalized)
RD-MLE (norm.): μ=420, σ=185
Normalized density (scaled to max = 1)
Elevation (m a.s.l.)
0.0
0.2
0.4
0.6
0.8
1.0
250
500
750
1000
1250
1500
1750
2000

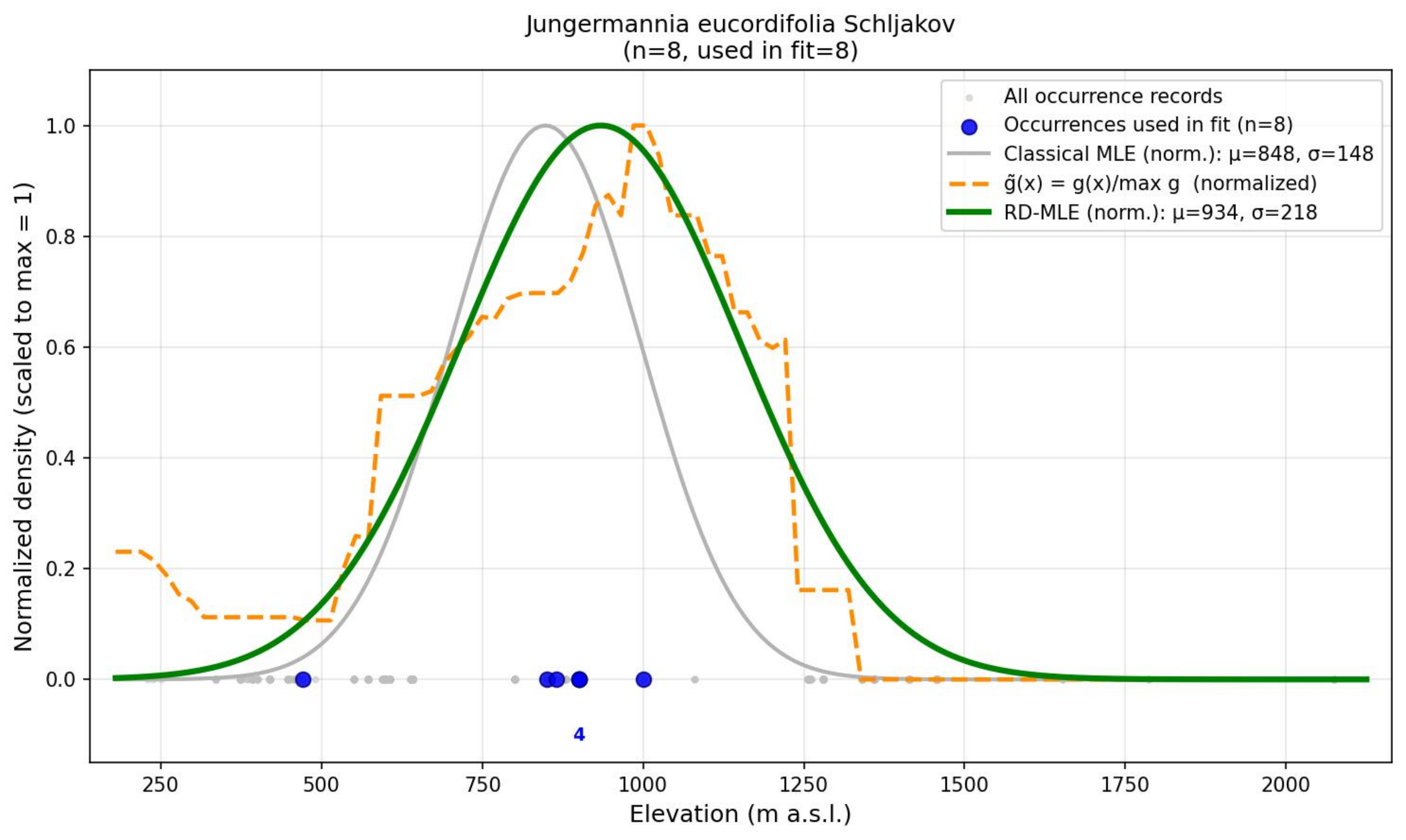

Jungermannia eucordifolia Schljakov
(n=8, used in fit=8)
All occurrence records
Occurrences used in fit (n=8)
Classical MLE (norm.): μ=848, σ=148
g̃(x) = g(x)/max g (normalized)
RD-MLE (norm.): μ=934, σ=218
Normalized density (scaled to max = 1)
Elevation (m a.s.l.)
0.0
0.2
0.4
0.6
0.8
1.0
250
500
750
1000
1250
1500
1750
2000
4

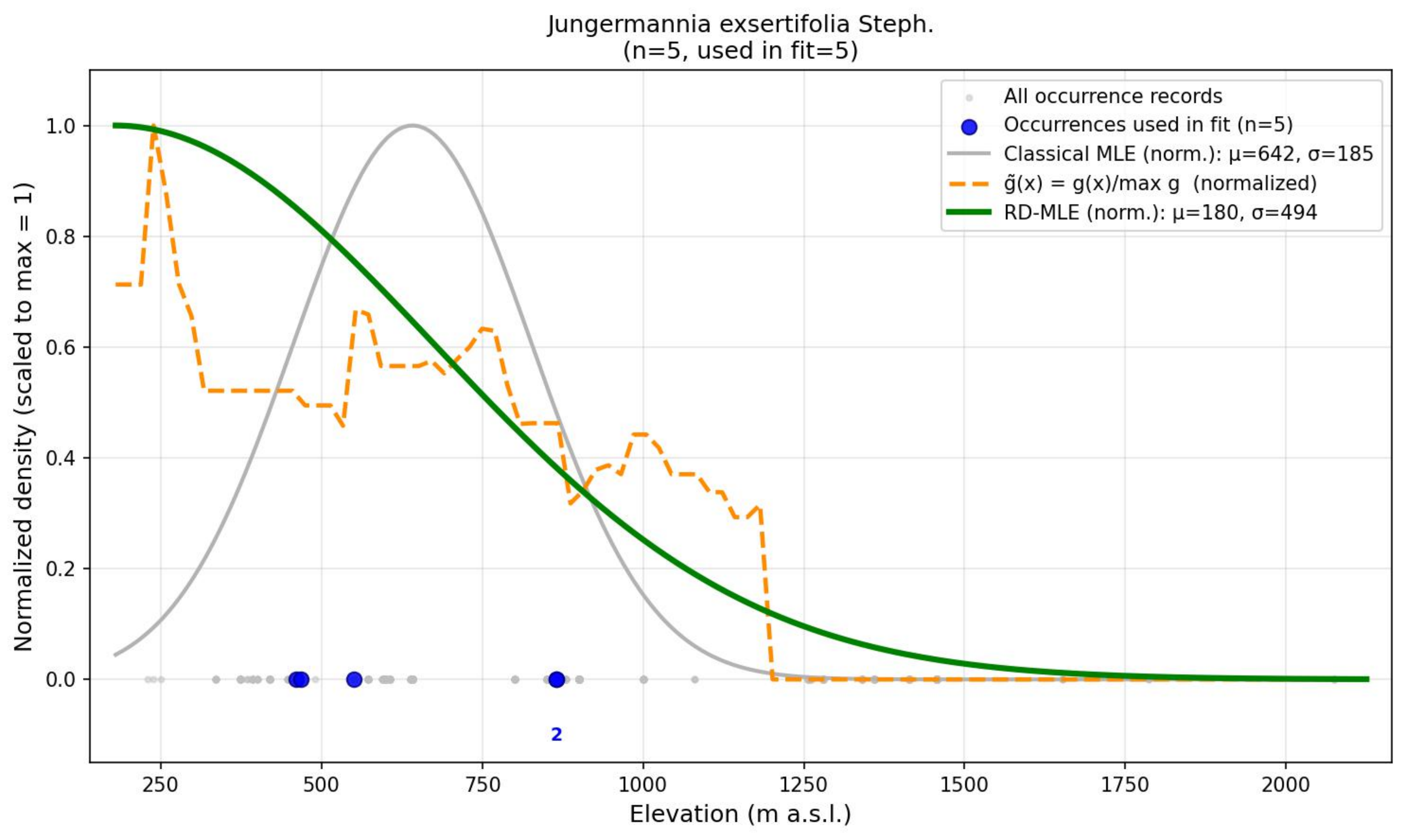
Jungermannia exsertifolia Steph.
(n=5, used in fit=5)
All occurrence records
Occurrences used in fit (n=5)
Classical MLE (norm.): μ=642, σ=185
g̃(x) = g(x)/max g (normalized)
RD-MLE (norm.): μ=180, σ=494
Normalized density (scaled to max = 1)
Elevation (m a.s.l.)
0.0
0.2
0.4
0.6
0.8
1.0
250
500
750
1000
1250
1500
1750
2000
2

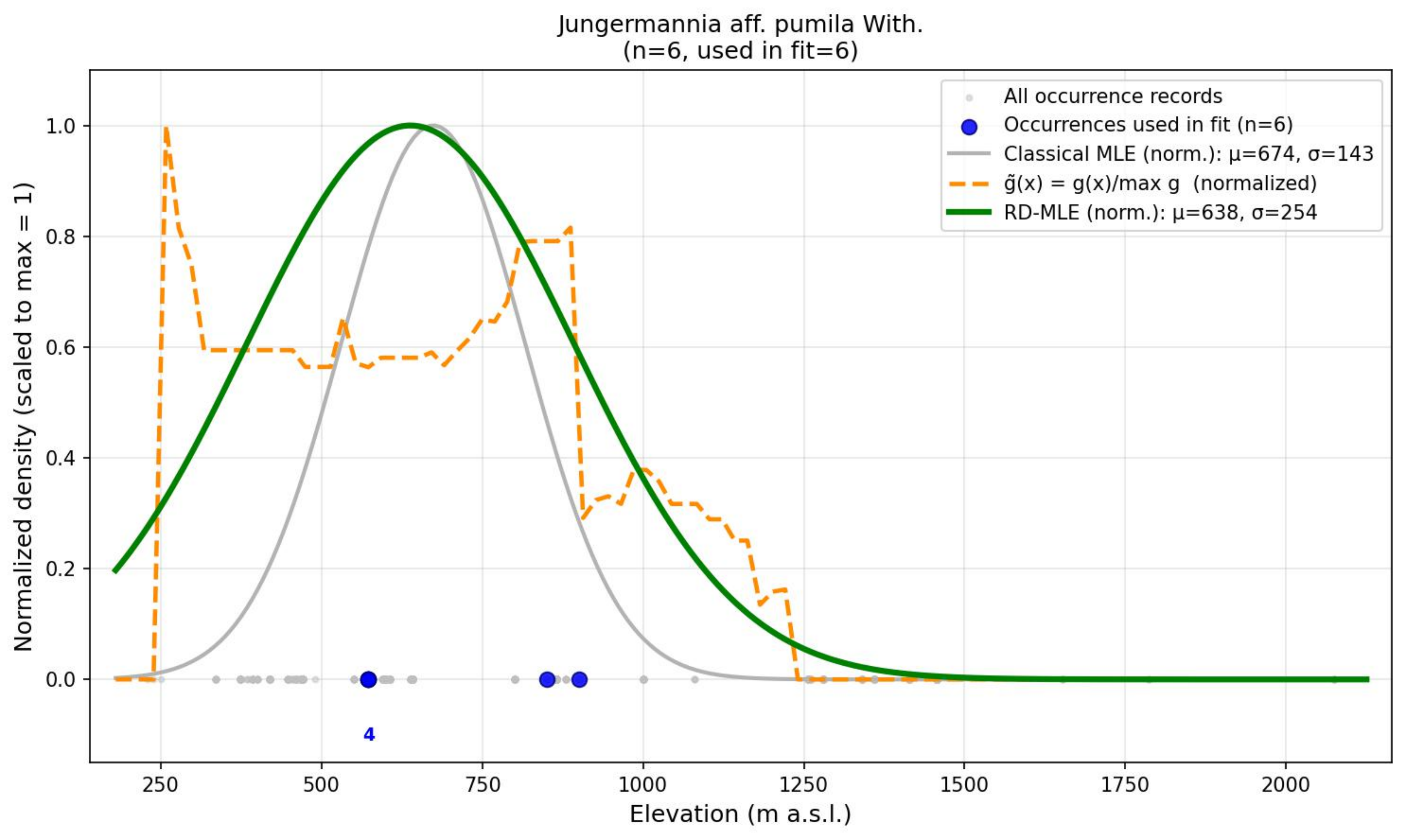

Jungermannia aff. pumila With.
(n=6, used in fit=6)
All occurrence records
Occurrences used in fit (n=6)
Classical MLE (norm.): μ=674, σ=143
g̃(x) = g(x)/max g (normalized)
RD-MLE (norm.): μ=638, σ=254
Normalized density (scaled to max = 1)
Elevation (m a.s.l.)
4
0.0
0.2
0.4
0.6
0.8
1.0
250
500
750
1000
1250
1500
1750
2000

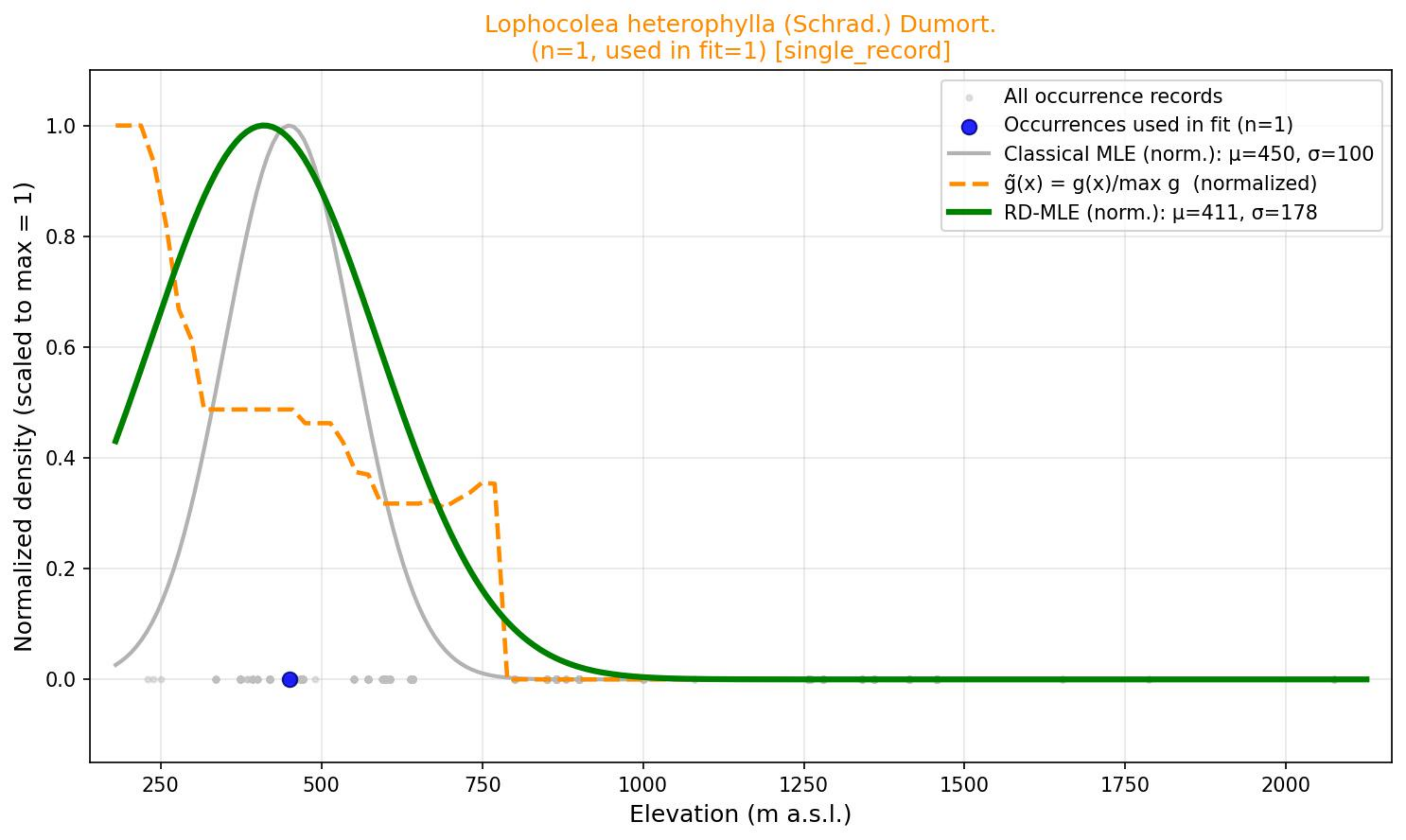

Lophocolea heterophylla (Schrad.) Dumort.
(n=1, used in fit=1) [single_record]
All occurrence records
Occurrences used in fit (n=1)
Classical MLE (norm.): μ=450, σ=100
g̃(x) = g(x)/max g (normalized)
RD-MLE (norm.): μ=411, σ=178
Normalized density (scaled to max = 1)
Elevation (m a.s.l.)
0.0
0.2
0.4
0.6
0.8
1.0
250
500
750
1000
1250
1500
1750
2000

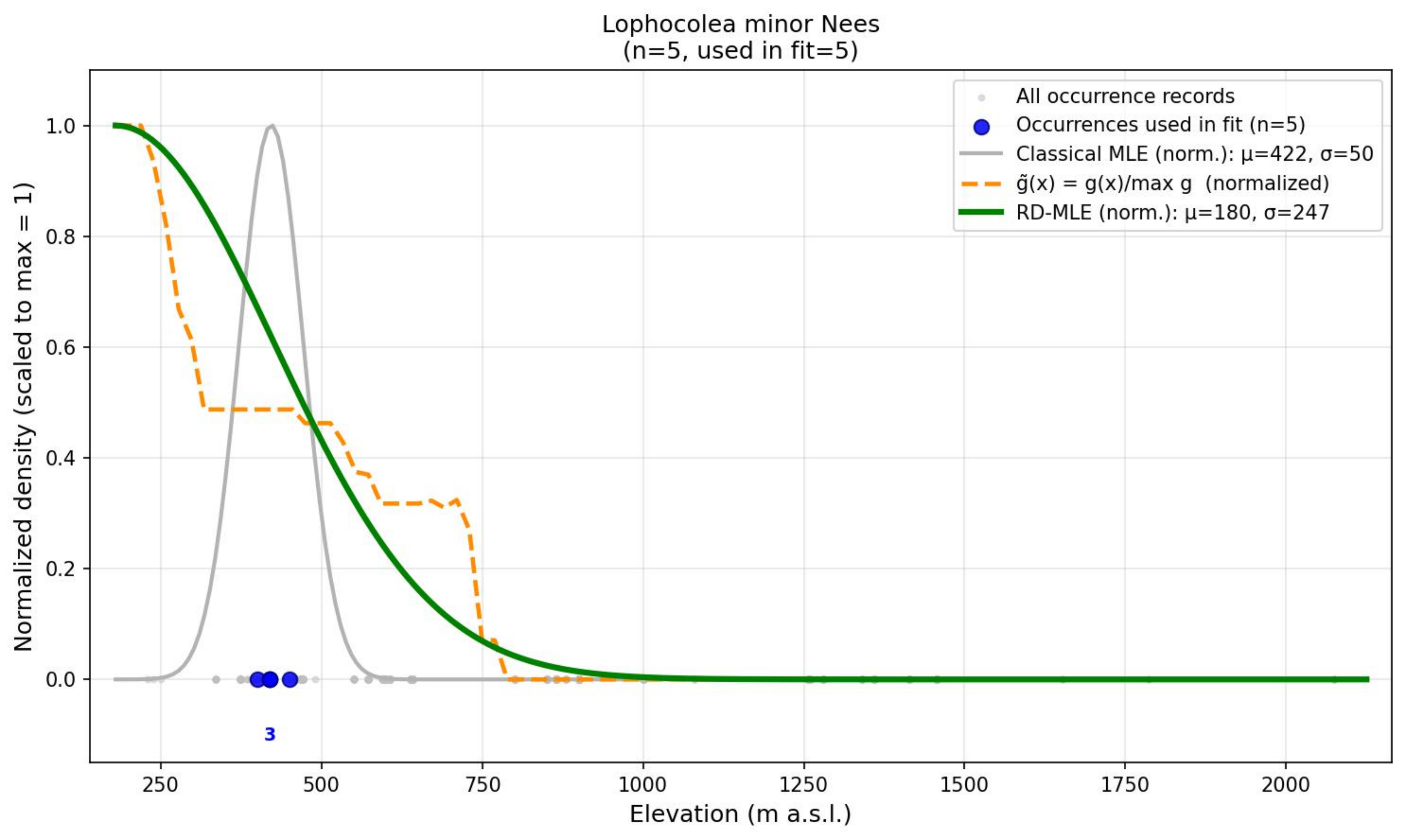

Lophocolea minor Nees
(n=5, used in fit=5)
All occurrence records
Occurrences used in fit (n=5)
Classical MLE (norm.): μ=422, σ=50
g̃(x) = g(x)/max g (normalized)
RD-MLE (norm.): μ=180, σ=247
Normalized density (scaled to max = 1)
Elevation (m a.s.l.)
1.0
0.8
0.6
0.4
0.2
0.0
250
500
750
1000
1250
1500
1750
2000
3

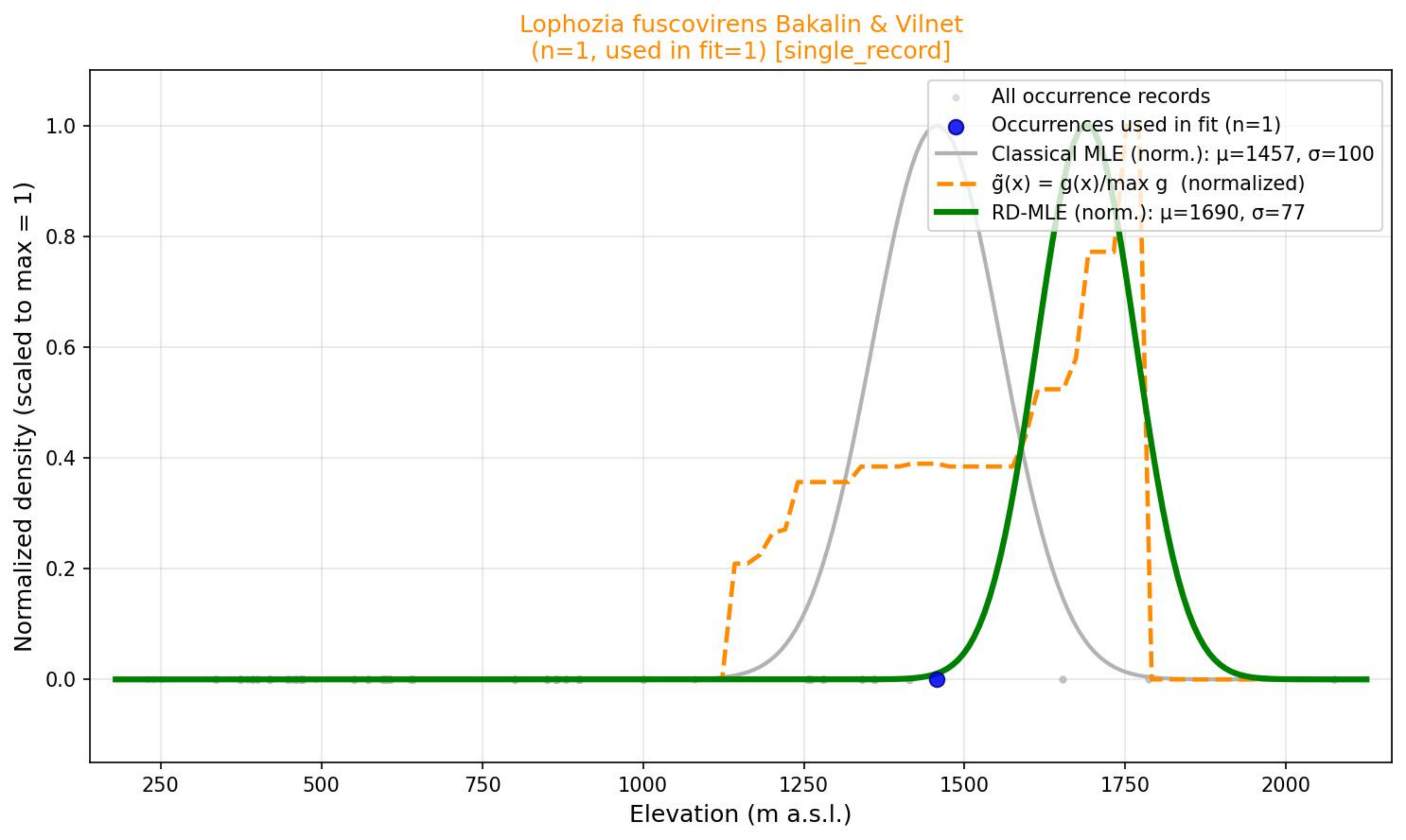

Lophozia fuscovirens Bakalin & Vilnet
(n=1, used in fit=1) [single_record]
All occurrence records
Occurrences used in fit (n=1)
Classical MLE (norm.): μ=1457, σ=100
g̃(x) = g(x)/max g (normalized)
RD-MLE (norm.): μ=1690, σ=77
Normalized density (scaled to max = 1)
Elevation (m a.s.l.)
0.0
0.2
0.4
0.6
0.8
1.0
250
500
750
1000
1250
1500
1750
2000

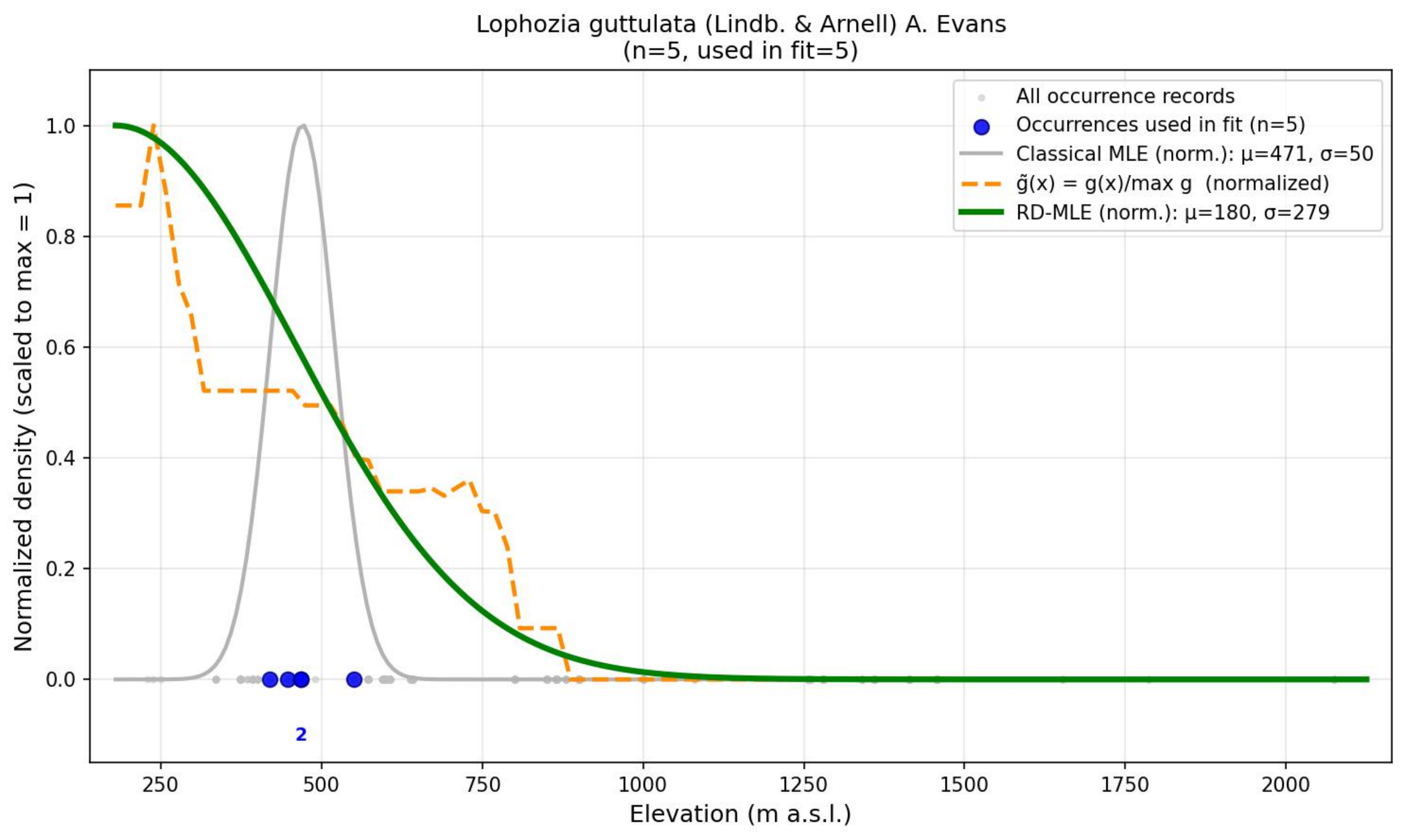

Lophozia guttulata (Lindb. & Arnell) A. Evans
(n=5, used in fit=5)
All occurrence records
Occurrences used in fit (n=5)
Classical MLE (norm.): μ=471, σ=50
g̃(x) = g(x)/max g (normalized)
RD-MLE (norm.): μ=180, σ=279
Normalized density (scaled to max = 1)
Elevation (m a.s.l.)
1.0
0.8
0.6
0.4
0.2
0.0
250
500
750
1000
1250
1500
1750
2000
2

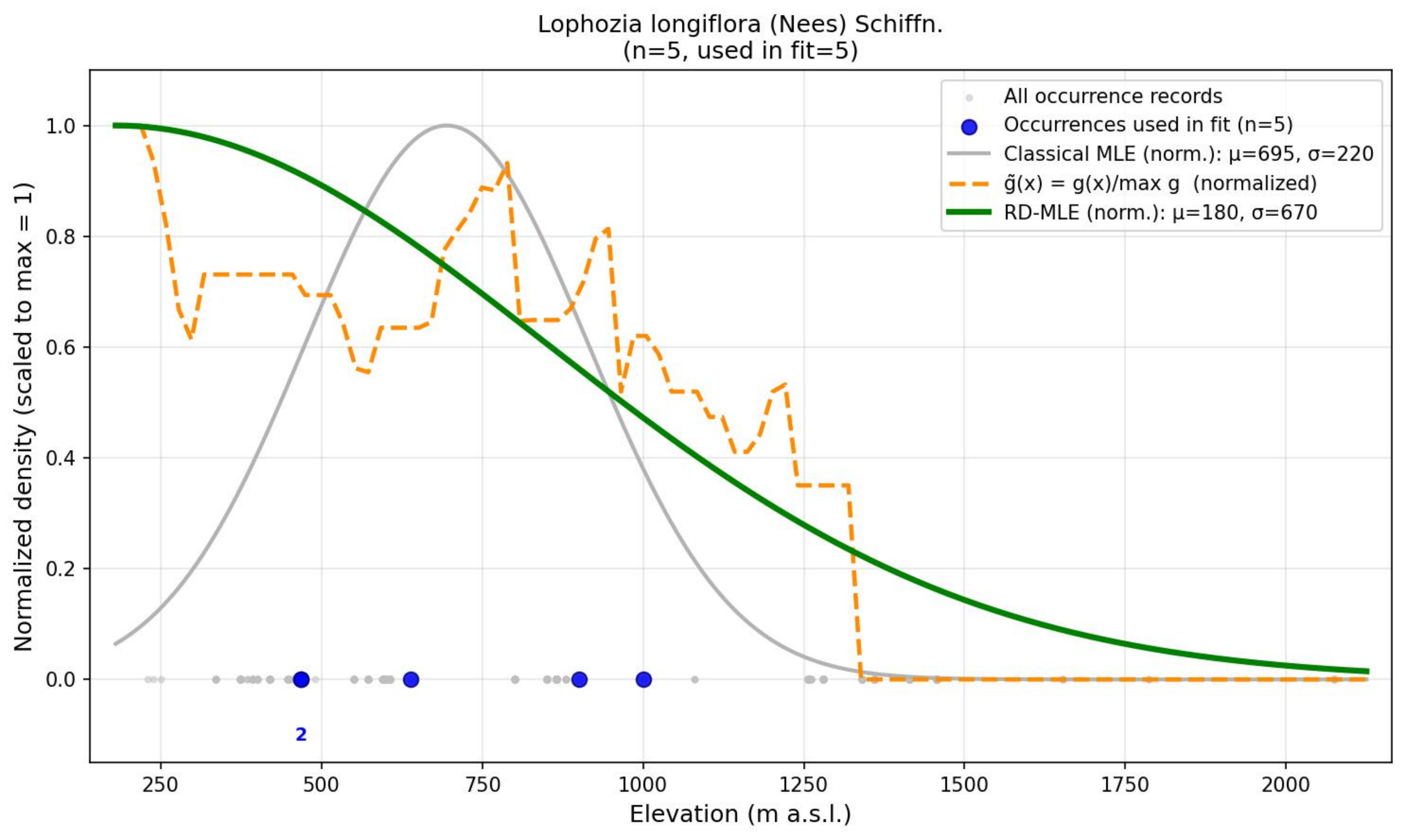

Lophozia longiflora (Nees) Schiffn.
(n=5, used in fit=5)
All occurrence records
Occurrences used in fit (n=5)
Classical MLE (norm.): μ=695, σ=220
g̃(x) = g(x)/max g (normalized)
RD-MLE (norm.): μ=180, σ=670
Normalized density (scaled to max = 1)
Elevation (m a.s.l.)
1.0
0.8
0.6
0.4
0.2
0.0
250
500
750
1000
1250
1500
1750
2000
2

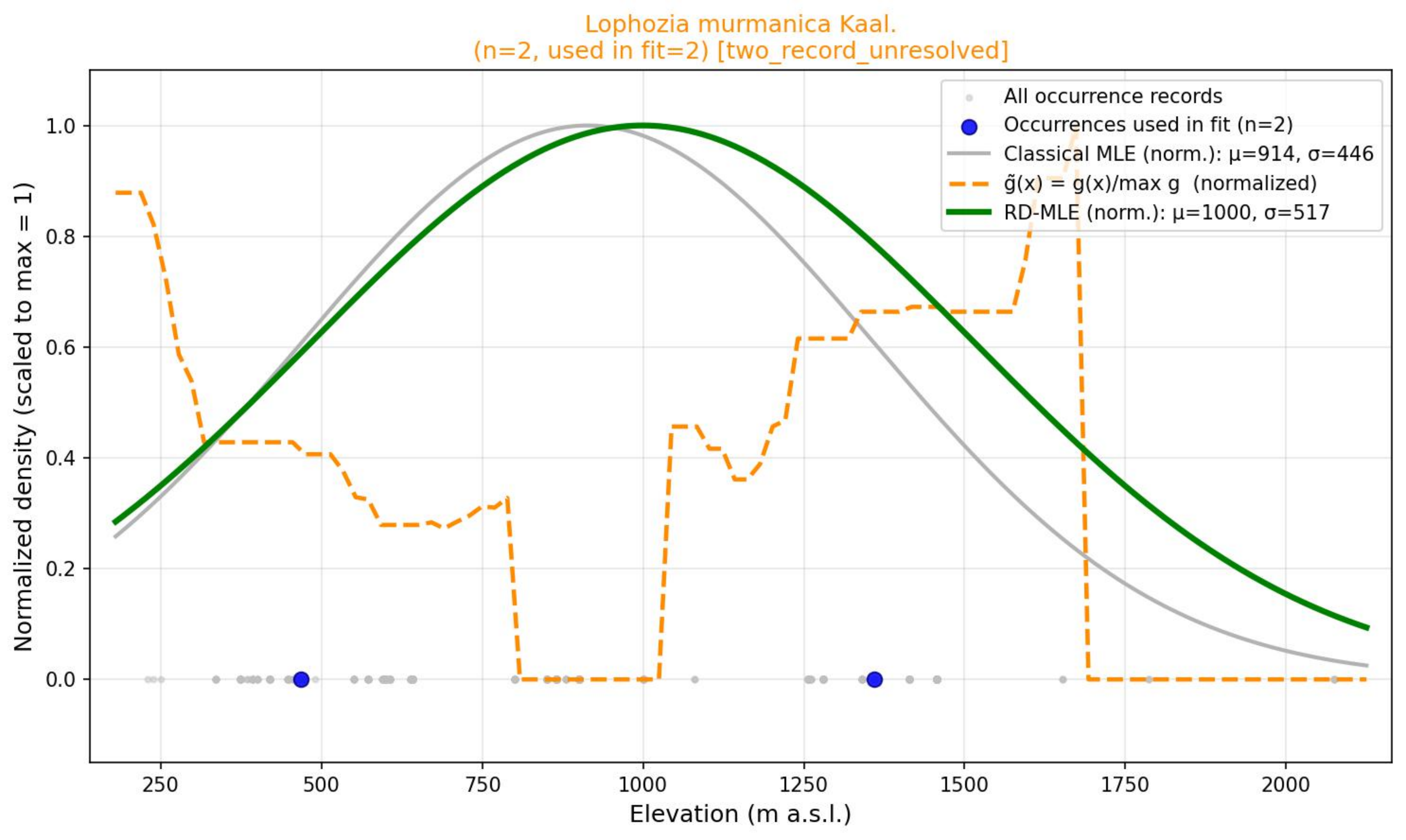

Lophozia murmanica Kaal.
(n=2, used in fit=2) [two_record_unresolved]
All occurrence records
Occurrences used in fit (n=2)
Classical MLE (norm.): μ=914, σ=446
g̃(x) = g(x)/max g (normalized)
RD-MLE (norm.): μ=1000, σ=517
Normalized density (scaled to max = 1)
0.0
0.2
0.4
0.6
0.8
1.0
250
500
750
1000
1250
1500
1750
2000
Elevation (m a.s.l.)

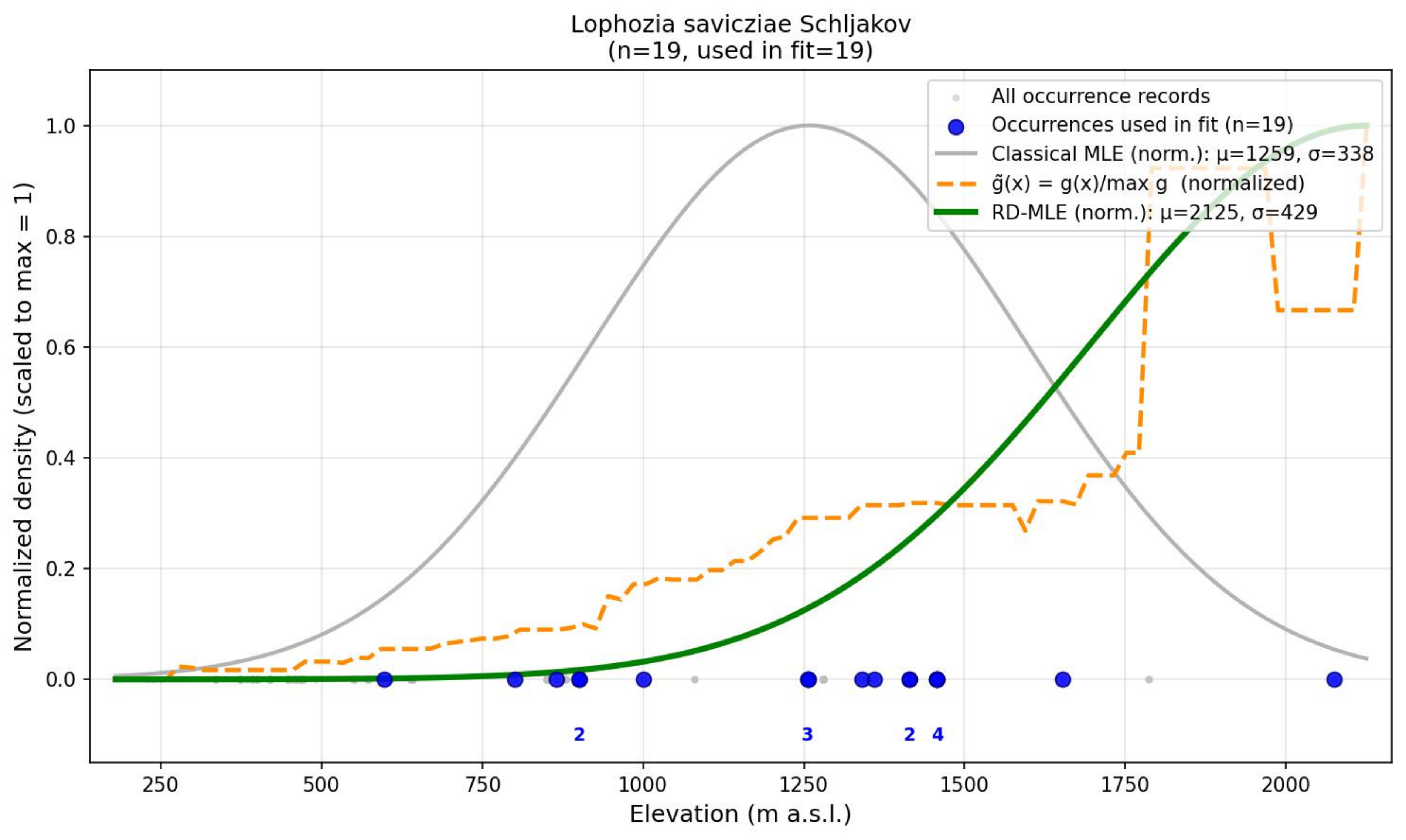

Lophozia saviczíae Schljakov
(n=19, used in fit=19)
All occurrence records
Occurrences used in fit (n=19)
Classical MLE (norm.): μ=1259, σ=338
g̃(x) = g(x)/max g (normalized)
RD-MLE (norm.): μ=2125, σ=429
Normalized density (scaled to max = 1)
Elevation (m a.s.l.)
1.0
0.8
0.6
0.4
0.2
0.0
250
500
750
1000
1250
1500
1750
2000
2
3
2
4

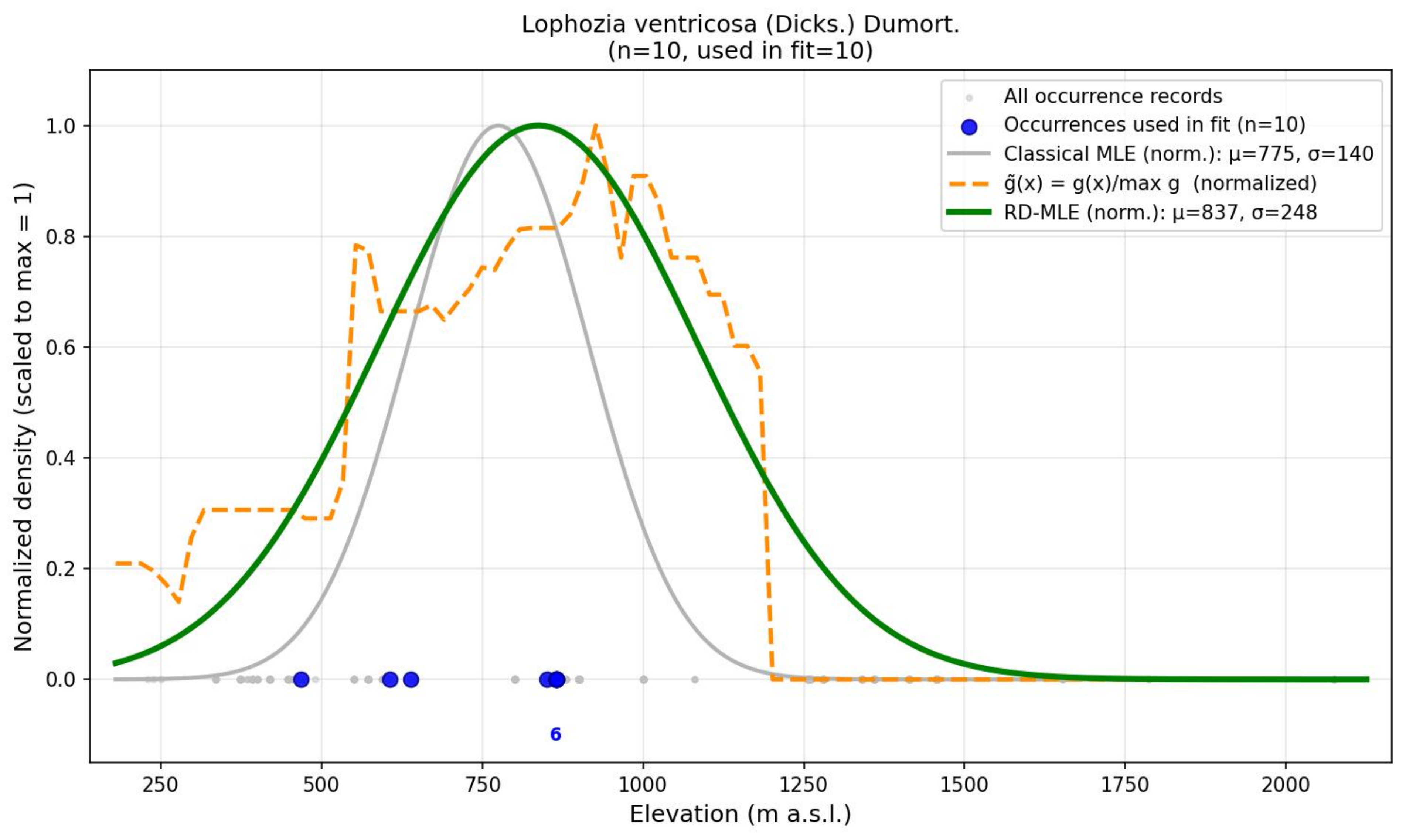
Lophozia ventricosa (Dicks.) Dumort.
(n=10, used in fit=10)
All occurrence records
Occurrences used in fit (n=10)
Classical MLE (norm.): μ=775, σ=140
g̃(x) = g(x)/max g (normalized)
RD-MLE (norm.): μ=837, σ=248
Normalized density (scaled to max = 1)
Elevation (m a.s.l.)
6
0.0
0.2
0.4
0.6
0.8
1.0
250
500
750
1000
1250
1500
1750
2000

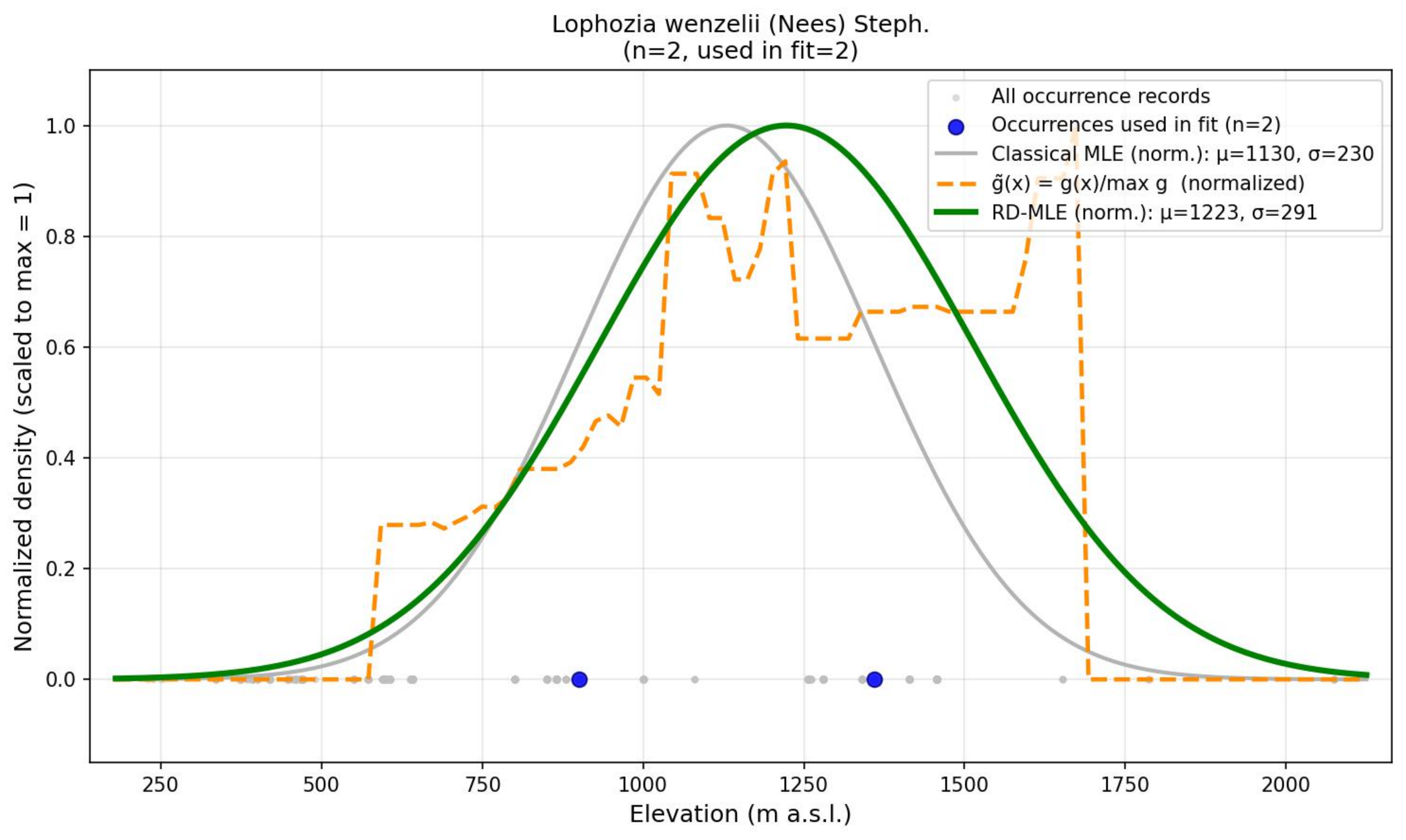
Lophozia wenzelii (Nees) Steph.
(n=2, used in fit=2)
All occurrence records
Occurrences used in fit (n=2)
Classical MLE (norm.): μ=1130, σ=230
g̃(x) = g(x)/max g (normalized)
RD-MLE (norm.): μ=1223, σ=291
Normalized density (scaled to max = 1)
Elevation (m a.s.l.)
1.0
0.8
0.6
0.4
0.2
0.0
250
500
750
1000
1250
1500
1750
2000

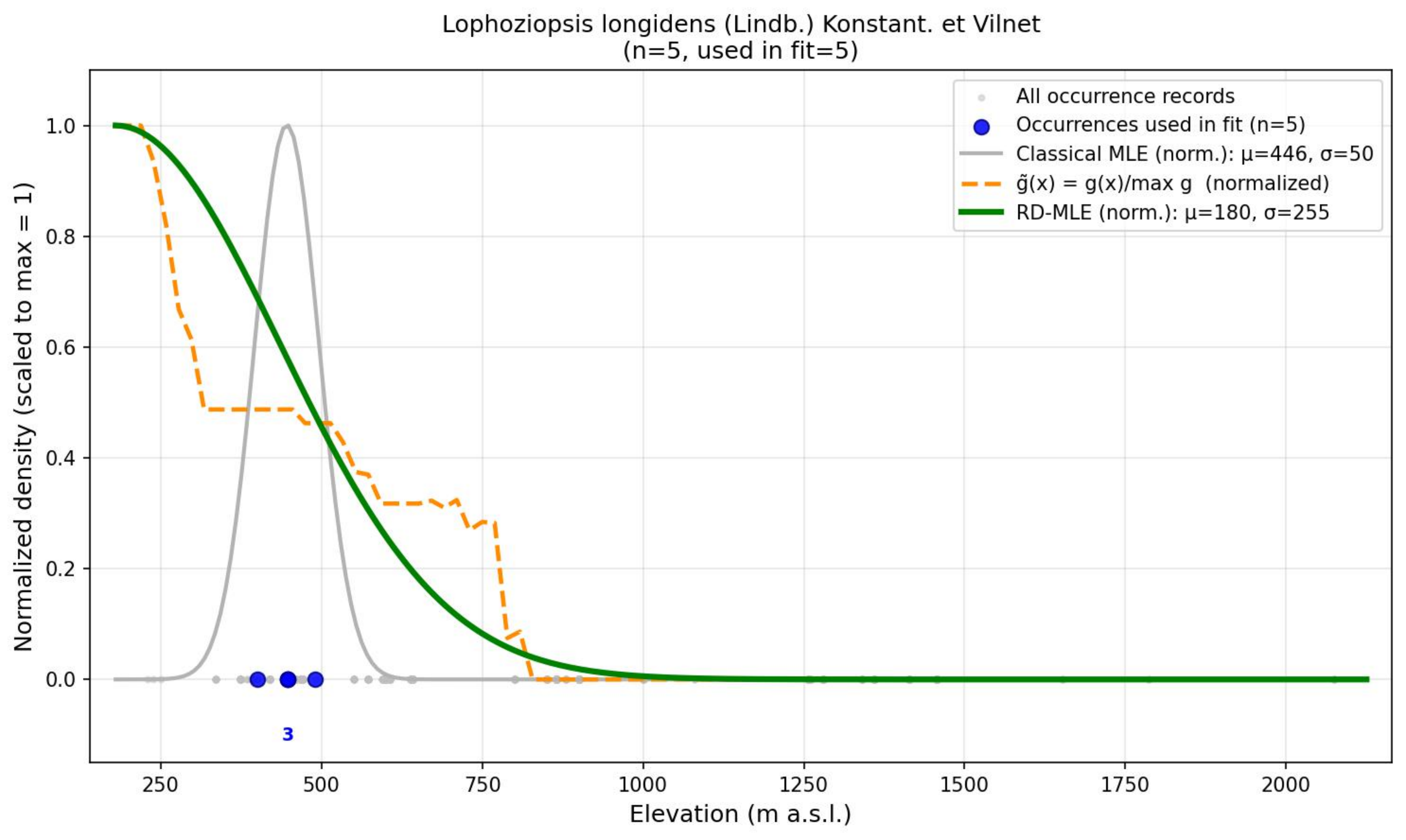

Lophoziopsis longidens (Lindb.) Konstant. et Vilnet
(n=5, used in fit=5)
All occurrence records
Occurrences used in fit (n=5)
Classical MLE (norm.): μ=446, σ=50
g̃(x) = g(x)/max g (normalized)
RD-MLE (norm.): μ=180, σ=255
Normalized density (scaled to max = 1)
Elevation (m a.s.l.)
0.0
0.2
0.4
0.6
0.8
1.0
250
500
750
1000
1250
1500
1750
2000
3

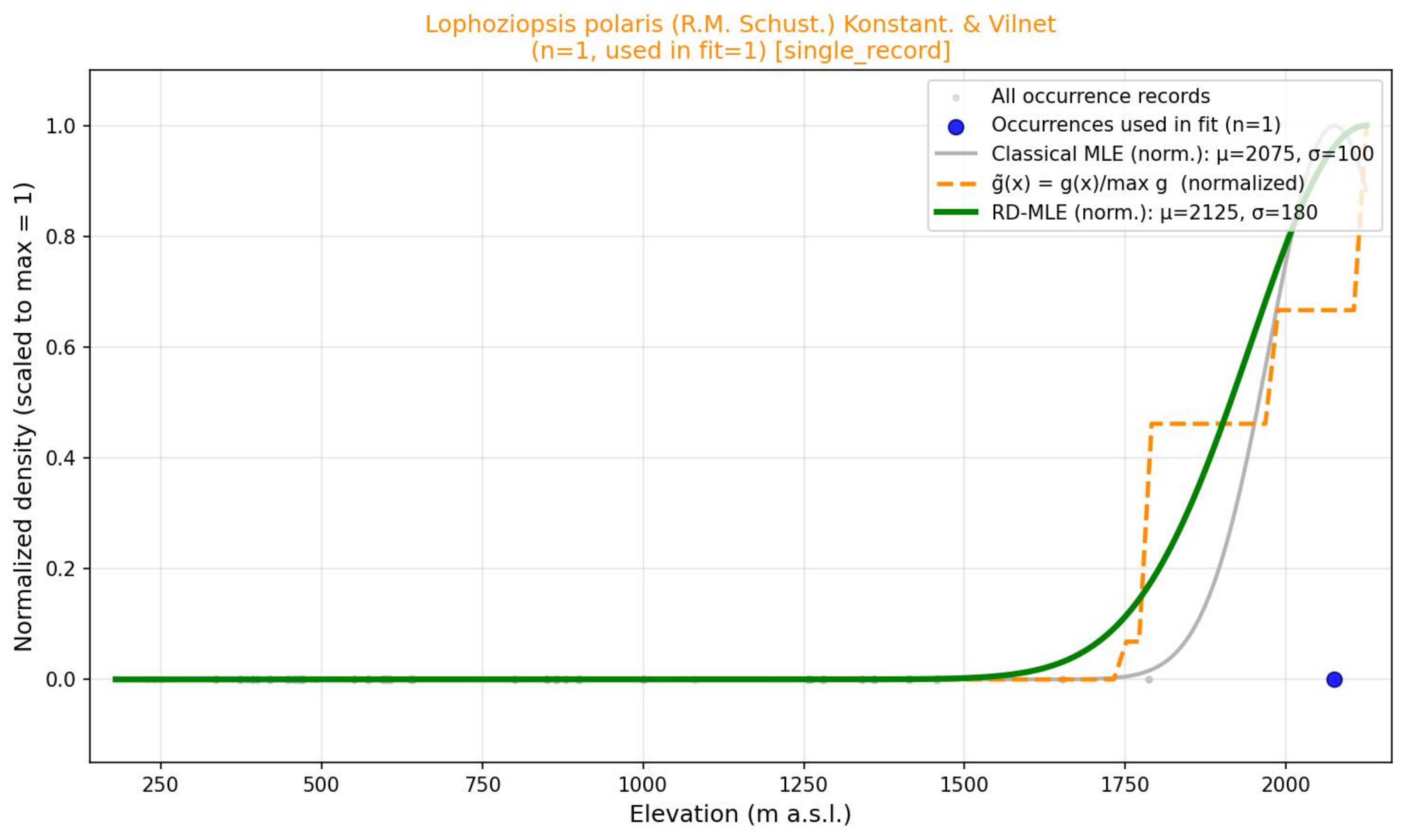

Lophoziopsis polaris (R.M. Schust.) Konstant. & Vilnet
(n=1, used in fit=1) [single_record]
All occurrence records
Occurrences used in fit (n=1)
Classical MLE (norm.): μ=2075, σ=100
g̃(x) = g(x)/max g (normalized)
RD-MLE (norm.): μ=2125, σ=180
Normalized density (scaled to max = 1)
Elevation (m a.s.l.)
1.0
0.8
0.6
0.4
0.2
0.0
250
500
750
1000
1250
1500
1750
2000

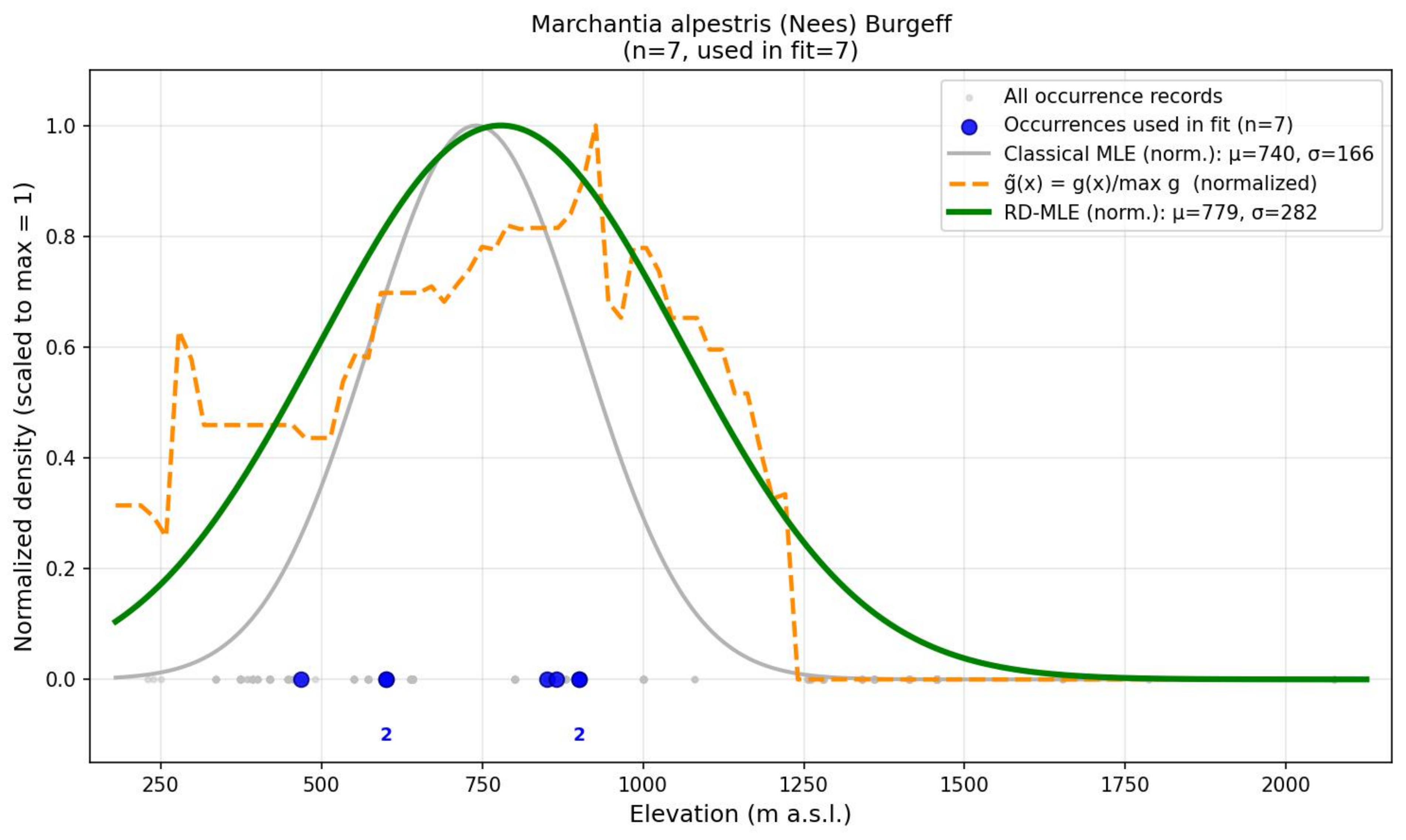

Marchantia alpestris (Nees) Burgeff
(n=7, used in fit=7)
All occurrence records
Occurrences used in fit (n=7)
Classical MLE (norm.): μ=740, σ=166
g̃(x) = g(x)/max g (normalized)
RD-MLE (norm.): μ=779, σ=282
Normalized density (scaled to max = 1)
Elevation (m a.s.l.)
0.0
0.2
0.4
0.6
0.8
1.0
250
500
750
1000
1250
1500
1750
2000
2
2

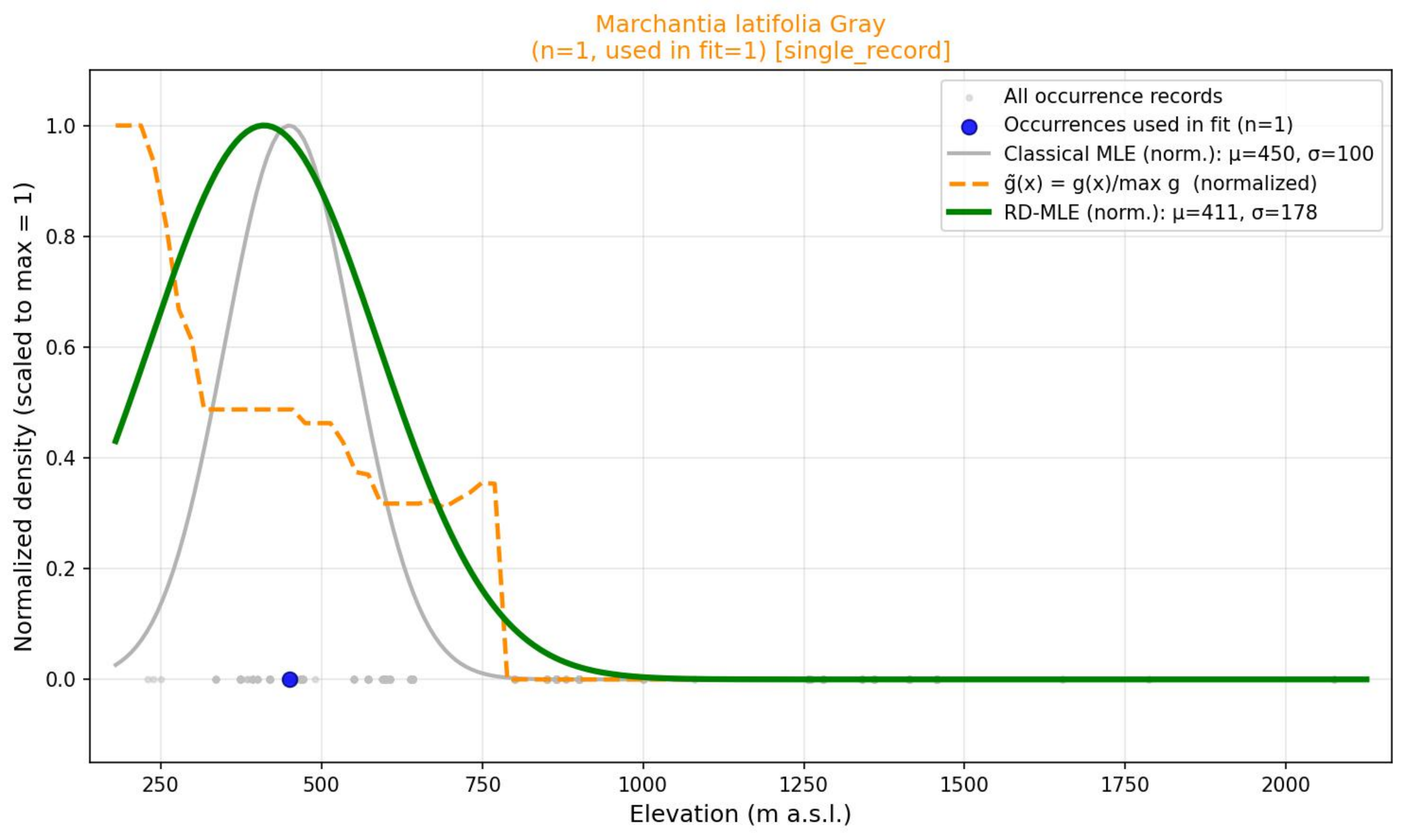

Marchantia latifolia Gray
(n=1, used in fit=1) [single_record]
All occurrence records
Occurrences used in fit (n=1)
Classical MLE (norm.): μ=450, σ=100
g̃(x) = g(x)/max g (normalized)
RD-MLE (norm.): μ=411, σ=178
Normalized density (scaled to max = 1)
Elevation (m a.s.l.)
0.0
0.2
0.4
0.6
0.8
1.0
250
500
750
1000
1250
1500
1750
2000

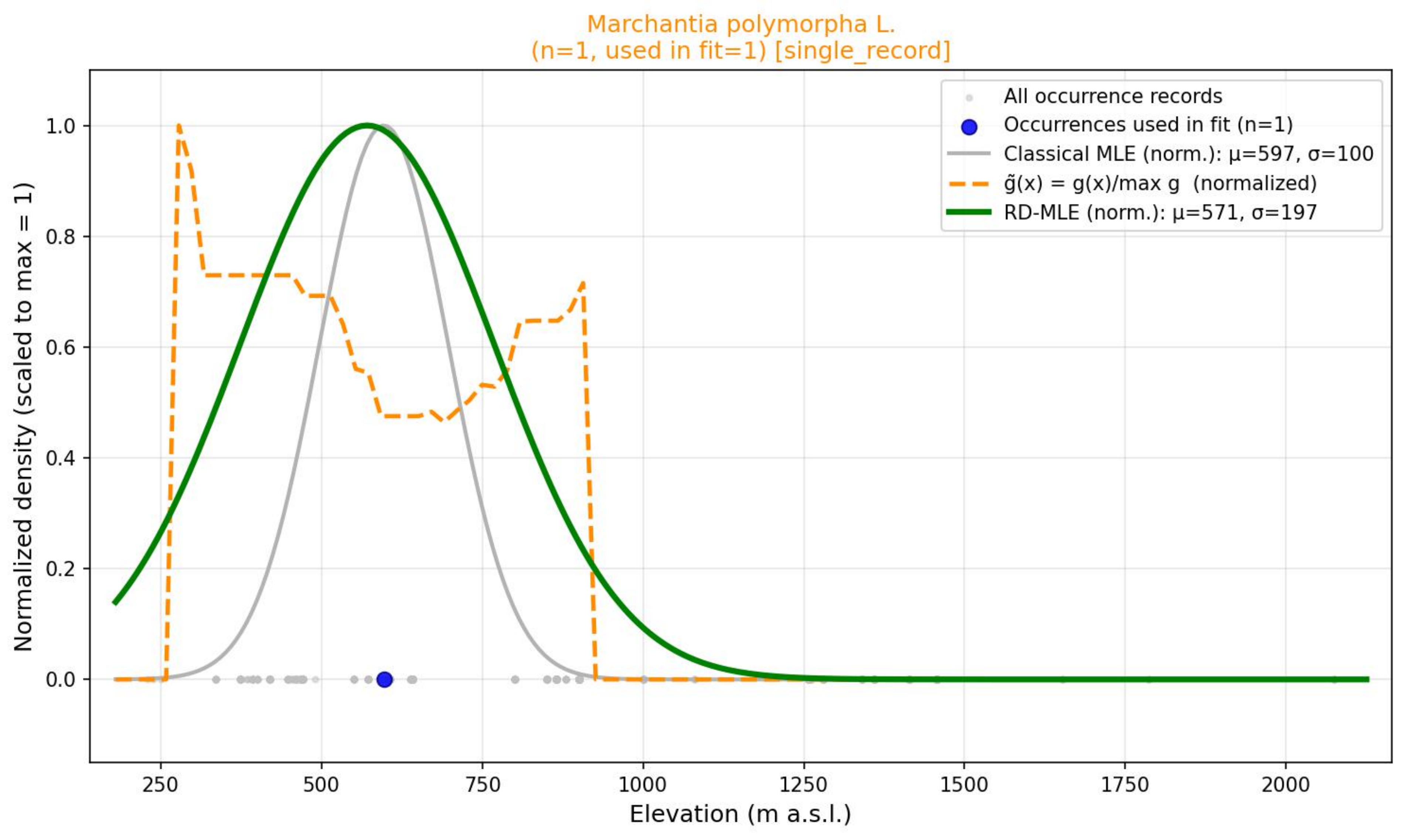

Marchantia polymorpha L.
(n=1, used in fit=1) [single_record]
All occurrence records
Occurrences used in fit (n=1)
Classical MLE (norm.): μ=597, σ=100
g̃(x) = g(x)/max g (normalized)
RD-MLE (norm.): μ=571, σ=197
Normalized density (scaled to max = 1)
1.0
0.8
0.6
0.4
0.2
0.0
250
500
750
1000
1250
1500
1750
2000
Elevation (m a.s.l.)

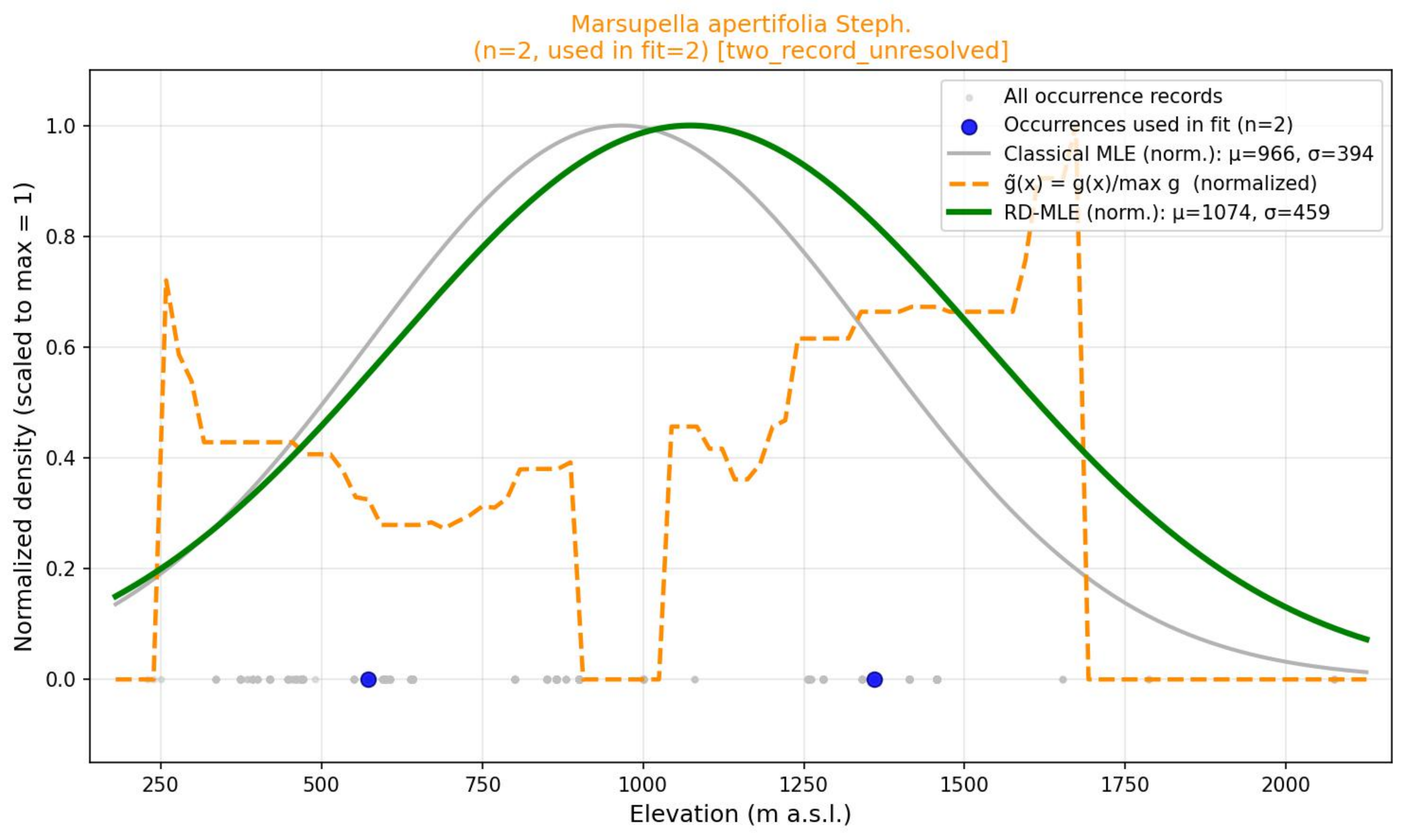
Marsupella apertifolia Steph.
(n=2, used in fit=2) [two_record_unresolved]
All occurrence records
Occurrences used in fit (n=2)
Classical MLE (norm.): μ=966, σ=394
g̃(x) = g(x)/max g (normalized)
RD-MLE (norm.): μ=1074, σ=459
Normalized density (scaled to max = 1)
Elevation (m a.s.l.)
0.0
0.2
0.4
0.6
0.8
1.0
250
500
750
1000
1250
1500
1750
2000

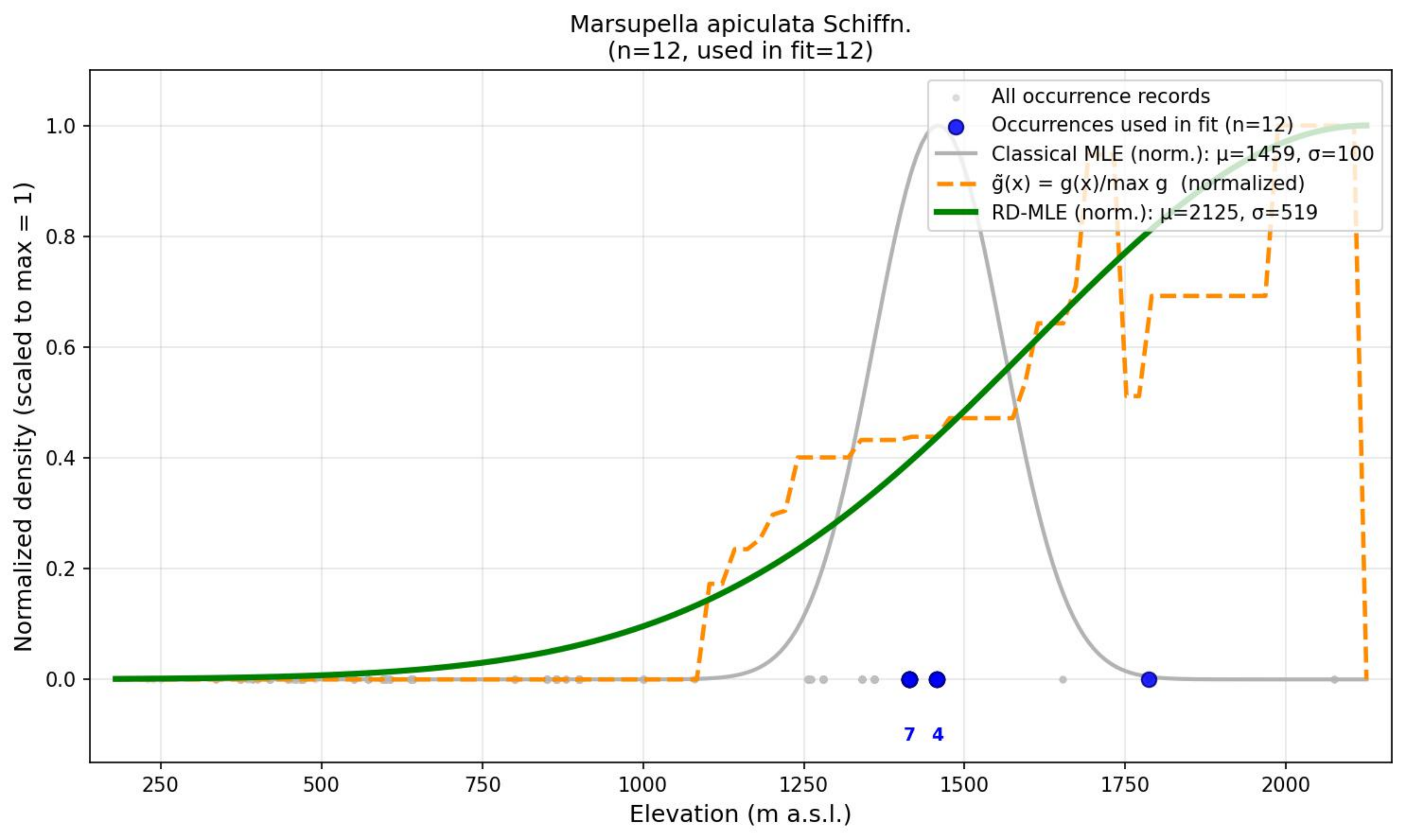

Marsupella apiculata Schiffn.
(n=12, used in fit=12)
All occurrence records
Occurrences used in fit (n=12)
Classical MLE (norm.): μ=1459, σ=100
g̃(x) = g(x)/max g (normalized)
RD-MLE (norm.): μ=2125, σ=519
Normalized density (scaled to max = 1)
1.0
0.8
0.6
0.4
0.2
0.0
7
4
250
500
750
1000
1250
1500
1750
2000
Elevation (m a.s.l.)

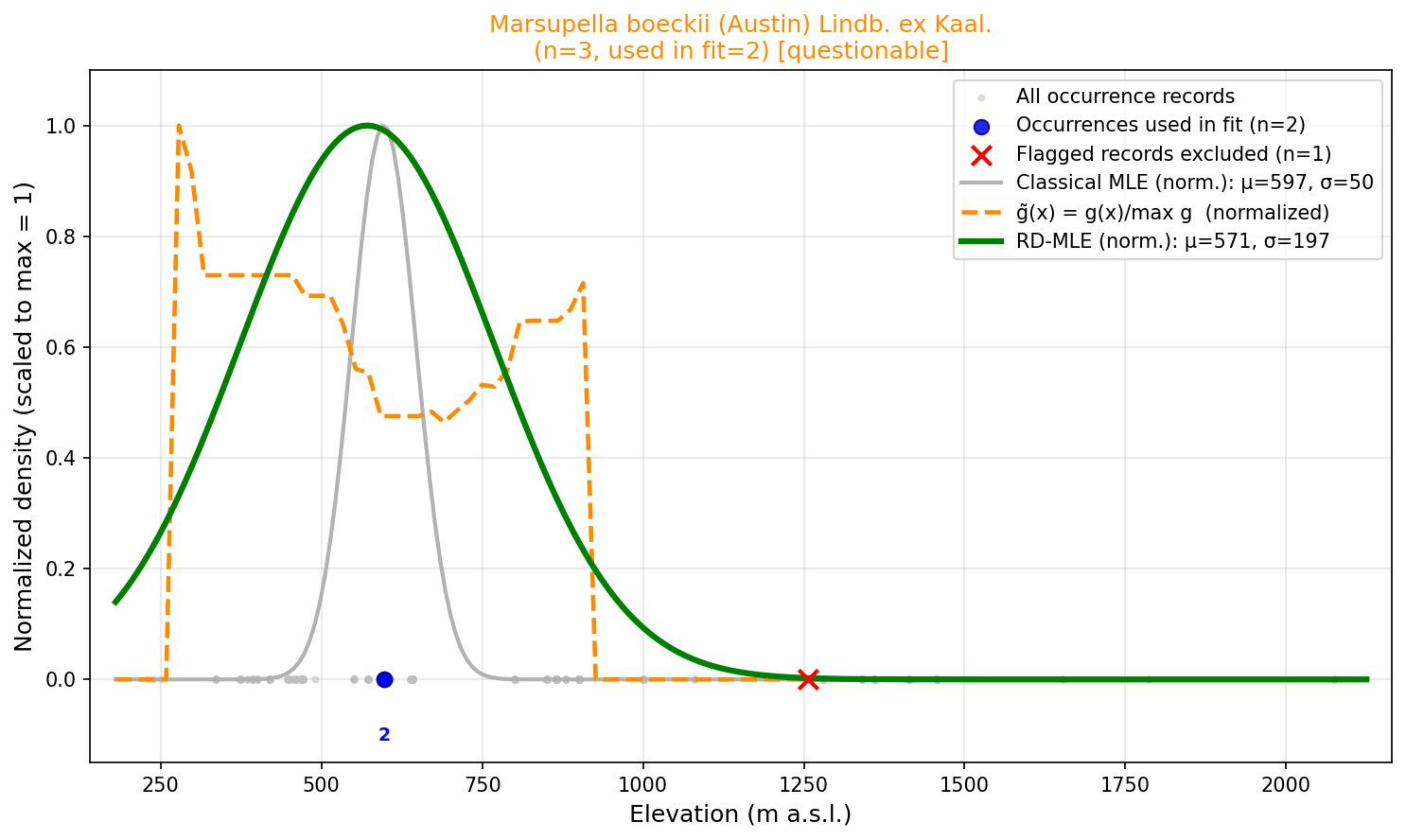

Marsupella boeckii (Austin) Lindb. ex Kaal.
(n=3, used in fit=2) [questionable]
All occurrence records
Occurrences used in fit (n=2)
Flagged records excluded (n=1)
Classical MLE (norm.): μ=597, σ=50
g̃(x) = g(x)/max g (normalized)
RD-MLE (norm.): μ=571, σ=197
Normalized density (scaled to max = 1)
Elevation (m a.s.l.)
2
1.0
0.8
0.6
0.4
0.2
0.0
250
500
750
1000
1250
1500
1750
2000

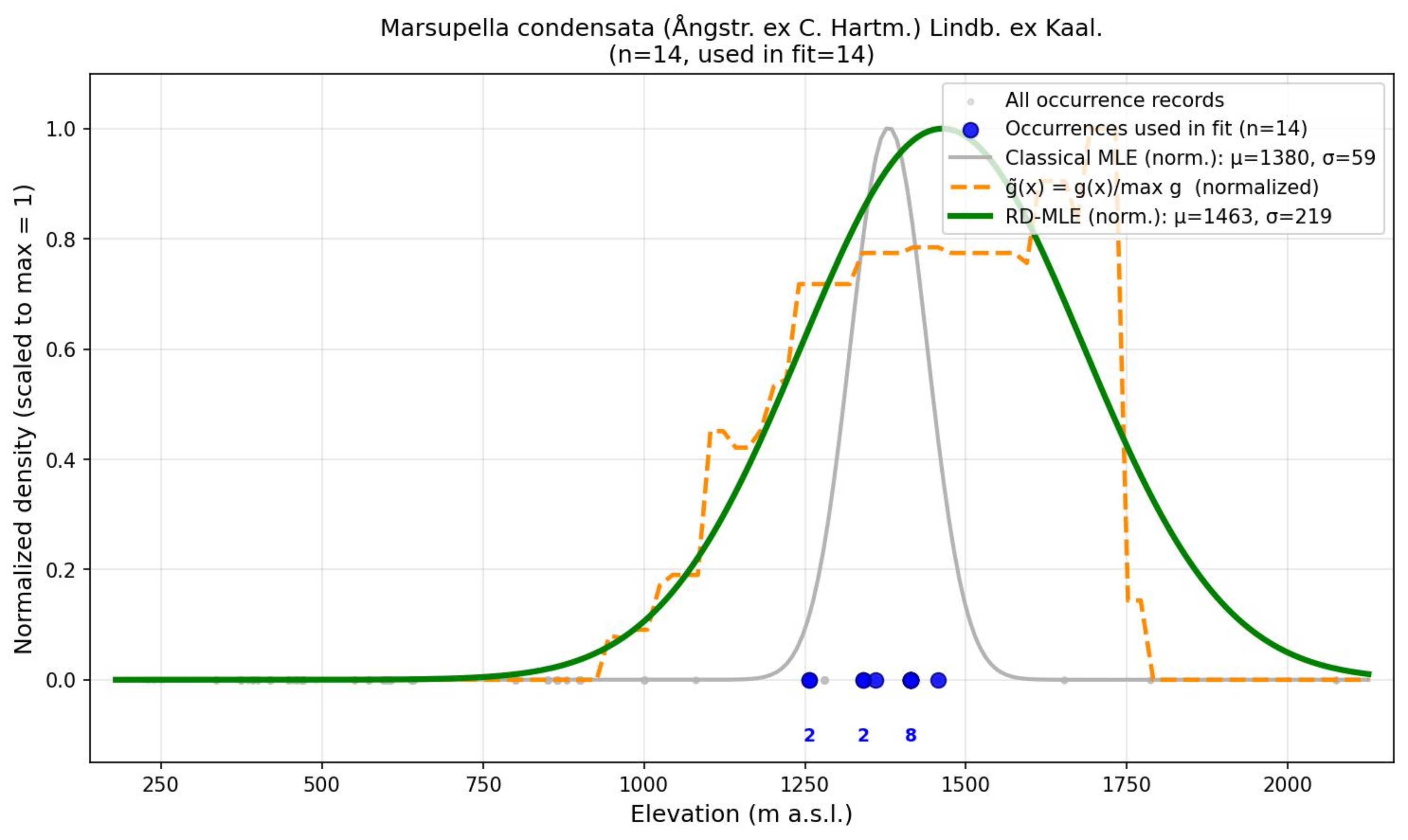

Marsupella condensata (Ångstr. ex C. Hartm.) Lindb. ex Kaal.
(n=14, used in fit=14)
All occurrence records
Occurrences used in fit (n=14)
Classical MLE (norm.): μ=1380, σ=59
g̃(x) = g(x)/max g (normalized)
RD-MLE (norm.): μ=1463, σ=219
Normalized density (scaled to max = 1)
1.0
0.8
0.6
0.4
0.2
0.0
2
2
8
250
500
750
1000
1250
1500
1750
2000
Elevation (m a.s.l.)

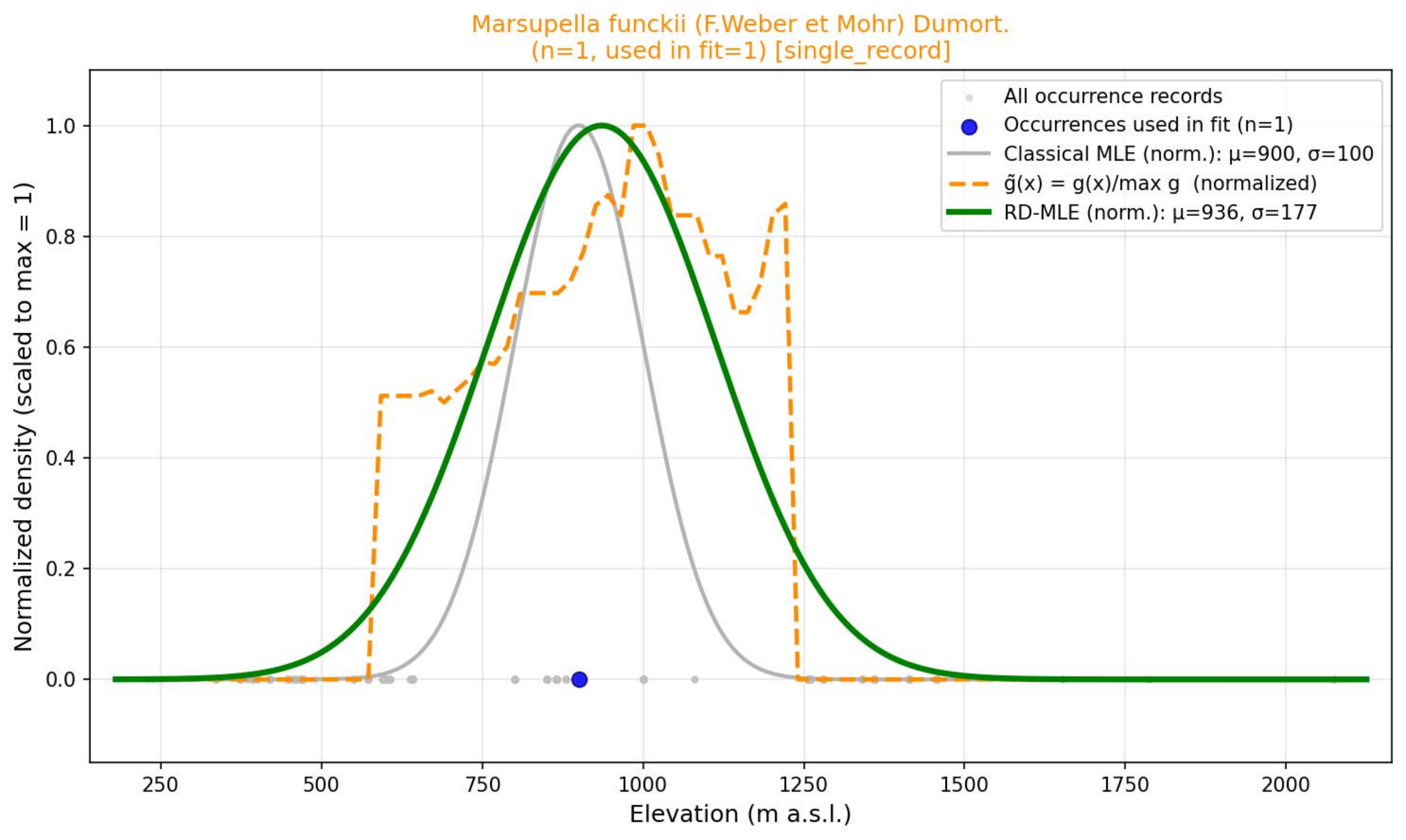
Marsupella funckii (F.Weber et Mohr) Dumort.
(n=1, used in fit=1) [single_record]
All occurrence records
Occurrences used in fit (n=1)
Classical MLE (norm.): μ=900, σ=100
g̃(x) = g(x)/max g (normalized)
RD-MLE (norm.): μ=936, σ=177
Normalized density (scaled to max = 1)
Elevation (m a.s.l.)
0.0
0.2
0.4
0.6
0.8
1.0
250
500
750
1000
1250
1500
1750
2000

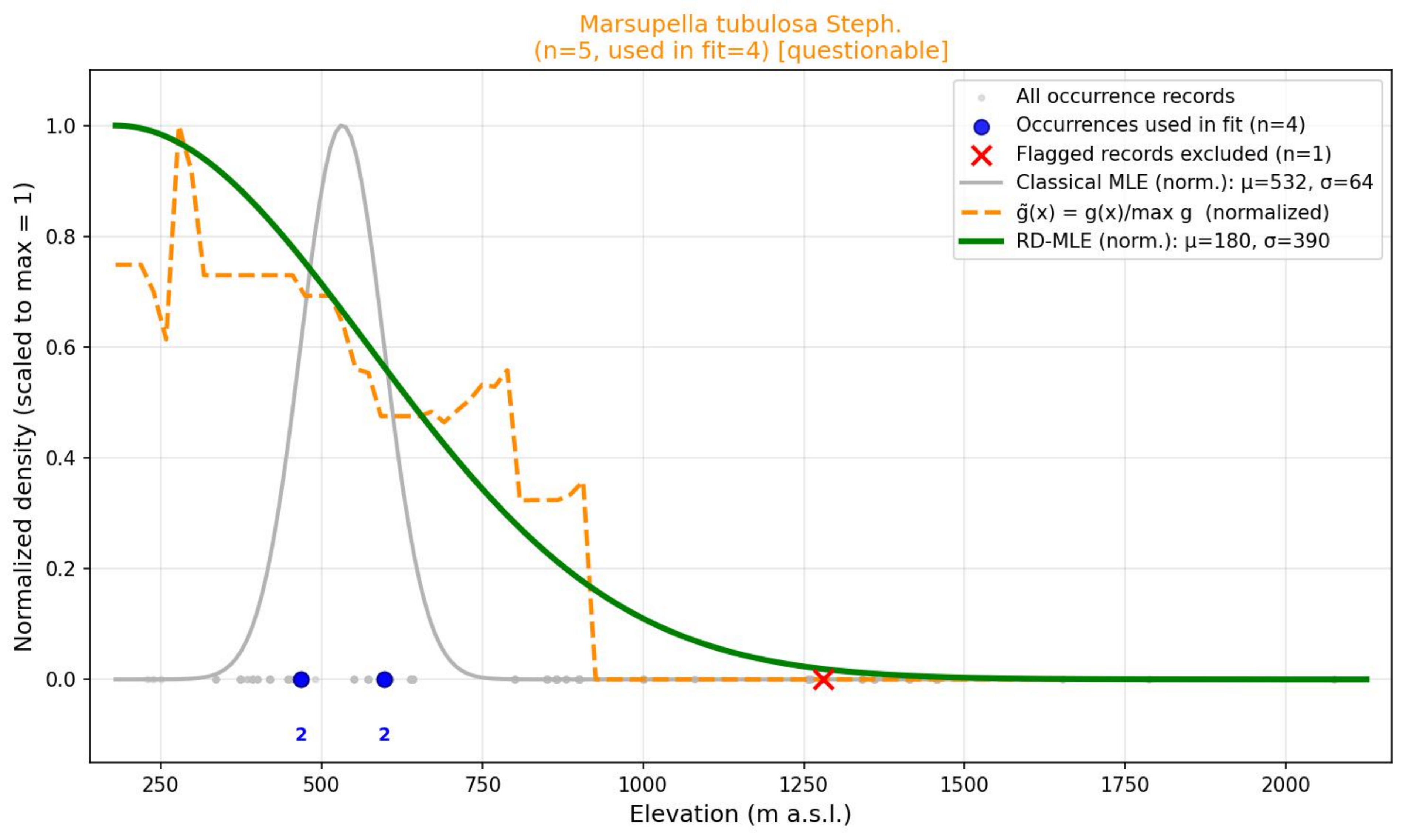

Marsupella tubulosa Steph.
(n=5, used in fit=4) [questionable]
All occurrence records
Occurrences used in fit (n=4)
Flagged records excluded (n=1)
Classical MLE (norm.): μ=532, σ=64
g̃(x) = g(x)/max g (normalized)
RD-MLE (norm.): μ=180, σ=390
Normalized density (scaled to max = 1)
Elevation (m a.s.l.)
1.0
0.8
0.6
0.4
0.2
0.0
250
500
750
1000
1250
1500
1750
2000
2
2

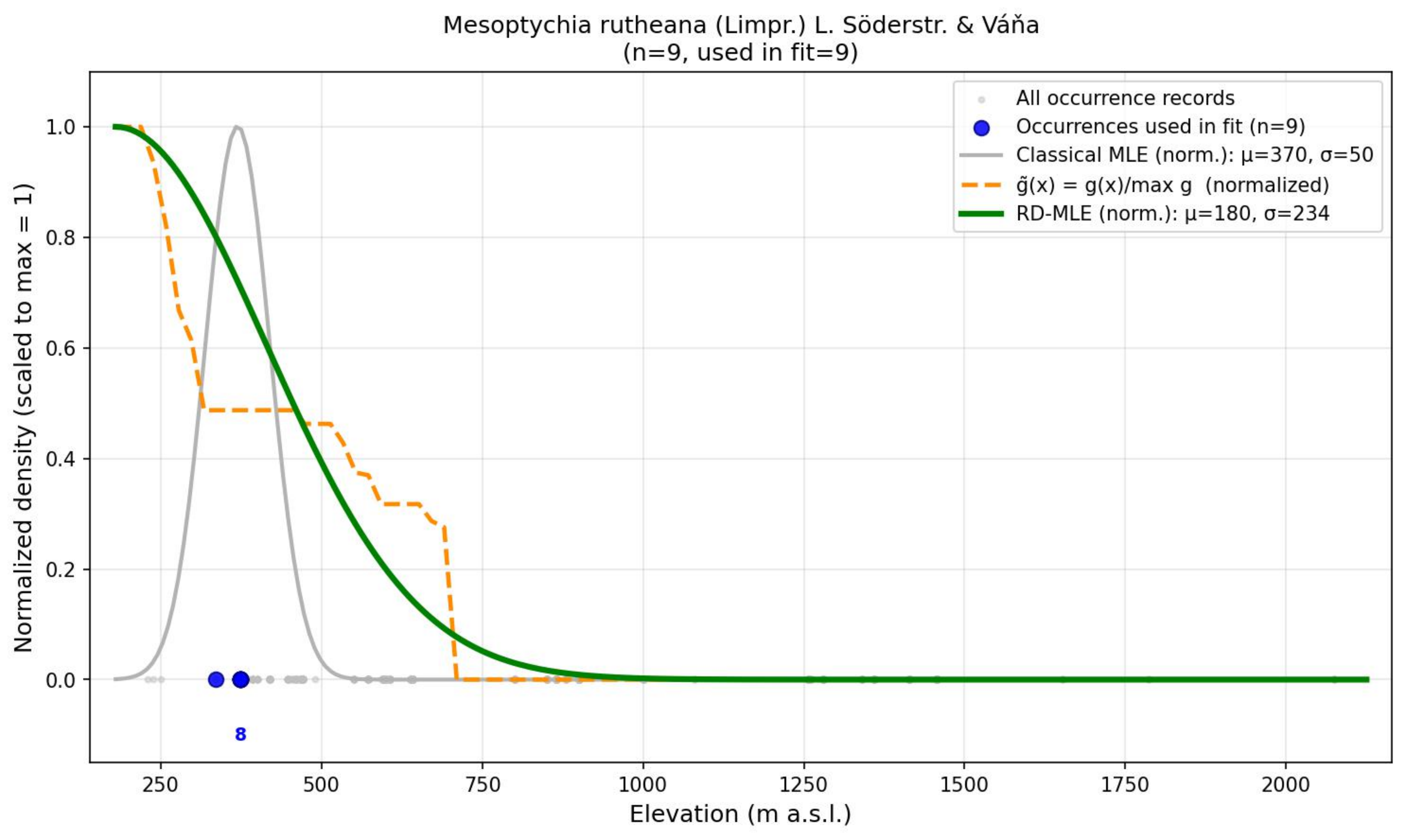

Mesoptychia rutheana (Limpr.) L. Söderstr. & Váňa
(n=9, used in fit=9)
All occurrence records
Occurrences used in fit (n=9)
Classical MLE (norm.): μ=370, σ=50
g̃(x) = g(x)/max g (normalized)
RD-MLE (norm.): μ=180, σ=234
Normalized density (scaled to max = 1)
Elevation (m a.s.l.)
8
0.0
0.2
0.4
0.6
0.8
1.0
250
500
750
1000
1250
1500
1750
2000

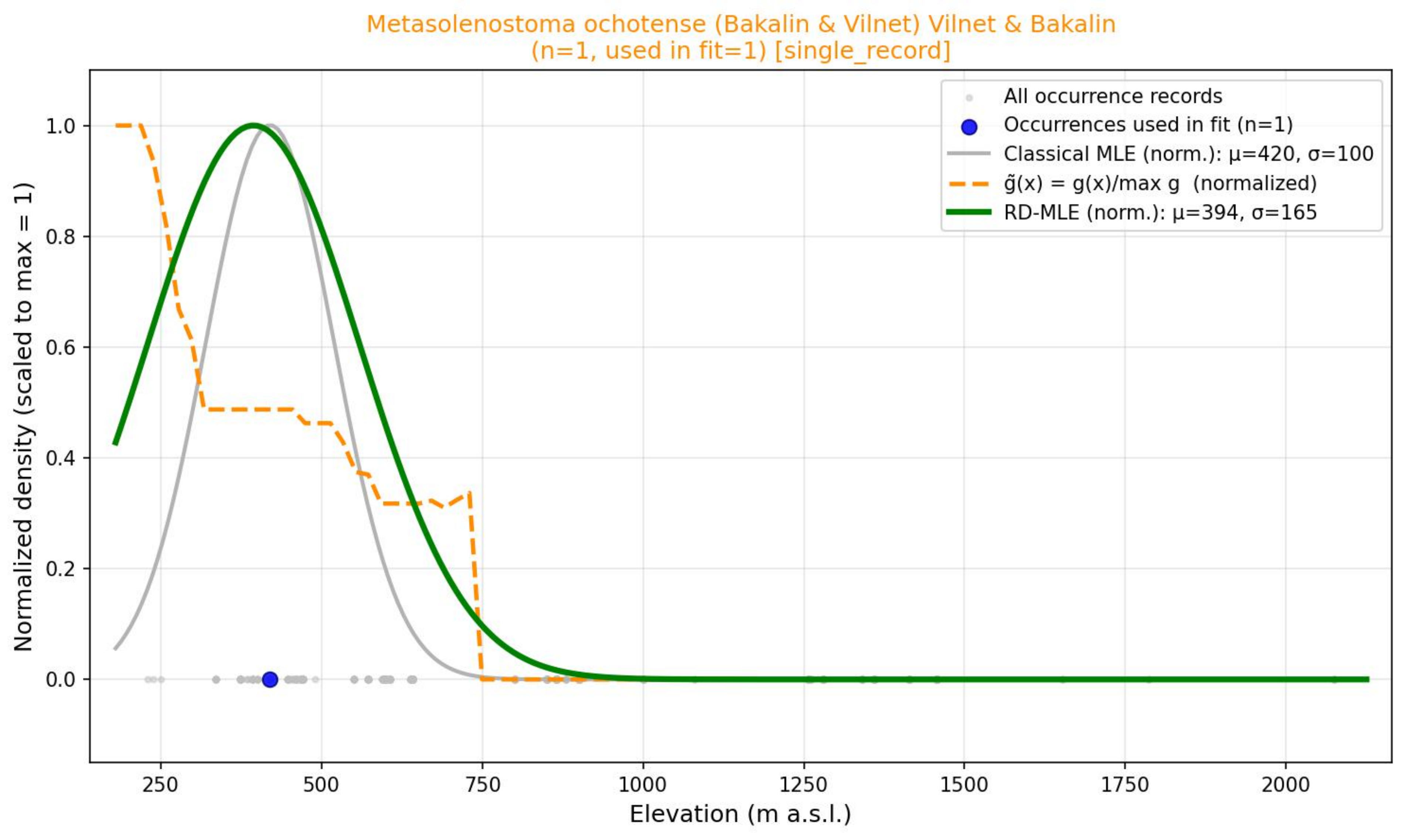
Metasolenostoma ochotense (Bakalin & Vilnet) Vilnet & Bakalin
(n=1, used in fit=1) [single_record]
All occurrence records
Occurrences used in fit (n=1)
Classical MLE (norm.): μ=420, σ=100
g̃(x) = g(x)/max g (normalized)
RD-MLE (norm.): μ=394, σ=165
Normalized density (scaled to max = 1)
Elevation (m a.s.l.)
1.0
0.8
0.6
0.4
0.2
0.0
250
500
750
1000
1250
1500
1750
2000

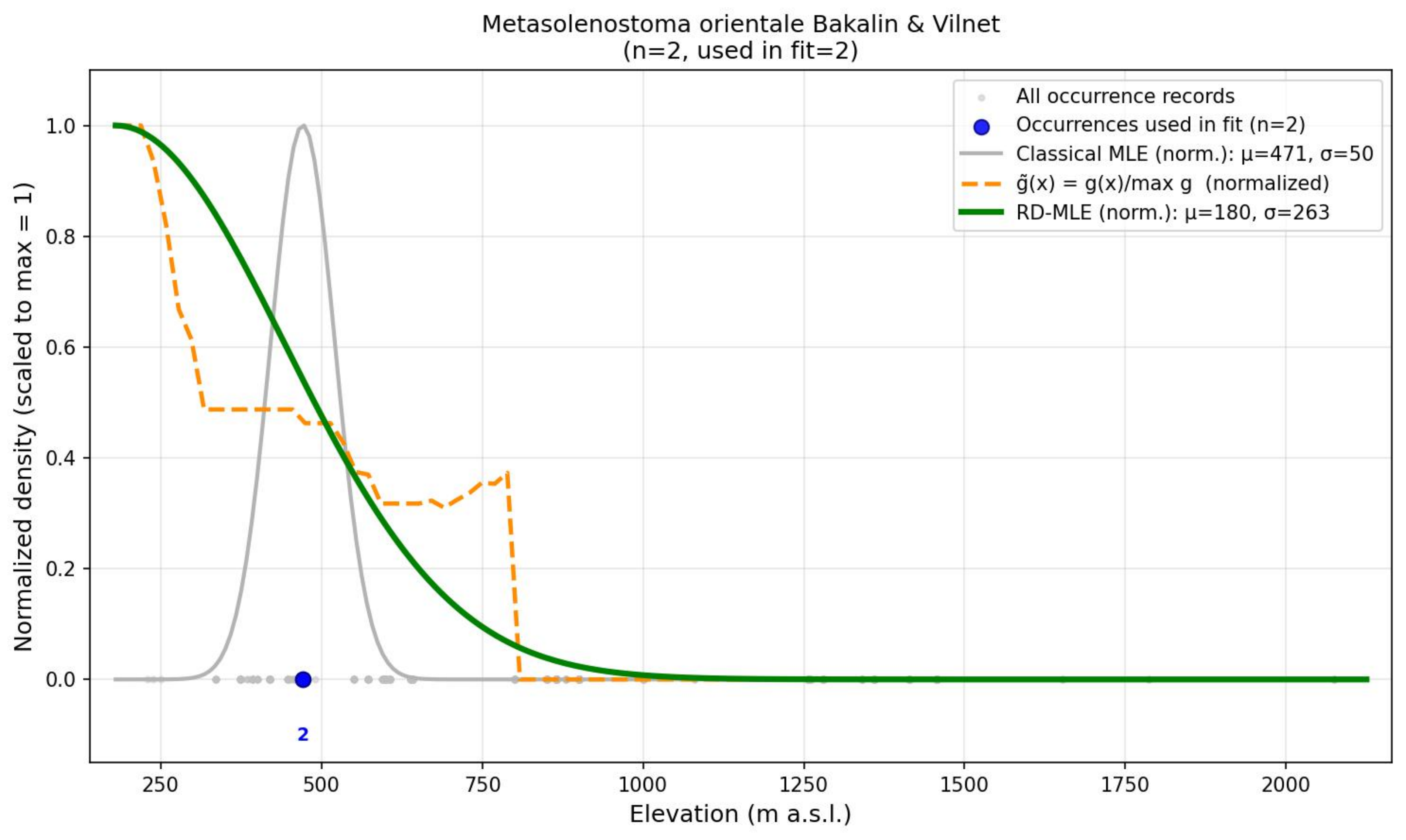

Metasolenostoma orientale Bakalin & Vilnet
(n=2, used in fit=2)
All occurrence records
Occurrences used in fit (n=2)
Classical MLE (norm.): μ=471, σ=50
g̃(x) = g(x)/max g (normalized)
RD-MLE (norm.): μ=180, σ=263
Normalized density (scaled to max = 1)
Elevation (m a.s.l.)
1.0
0.8
0.6
0.4
0.2
0.0
250
500
750
1000
1250
1500
1750
2000
2

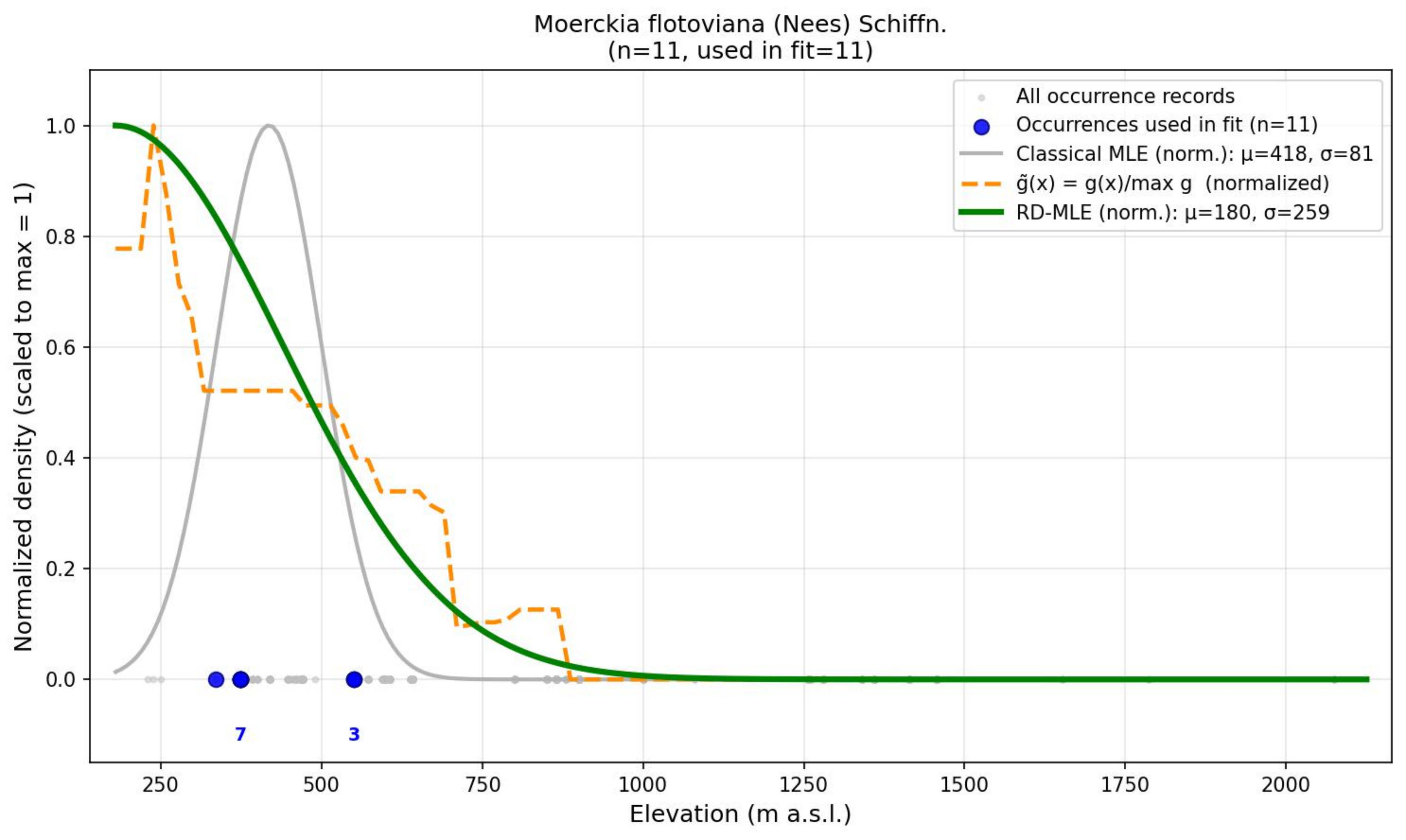

Moerckia flotoviana (Nees) Schiffn.
(n=11, used in fit=11)
All occurrence records
Occurrences used in fit (n=11)
Classical MLE (norm.): μ=418, σ=81
g̃(x) = g(x)/max g (normalized)
RD-MLE (norm.): μ=180, σ=259
Normalized density (scaled to max = 1)
Elevation (m a.s.l.)
7
3
0.0
0.2
0.4
0.6
0.8
1.0
250
500
750
1000
1250
1500
1750
2000

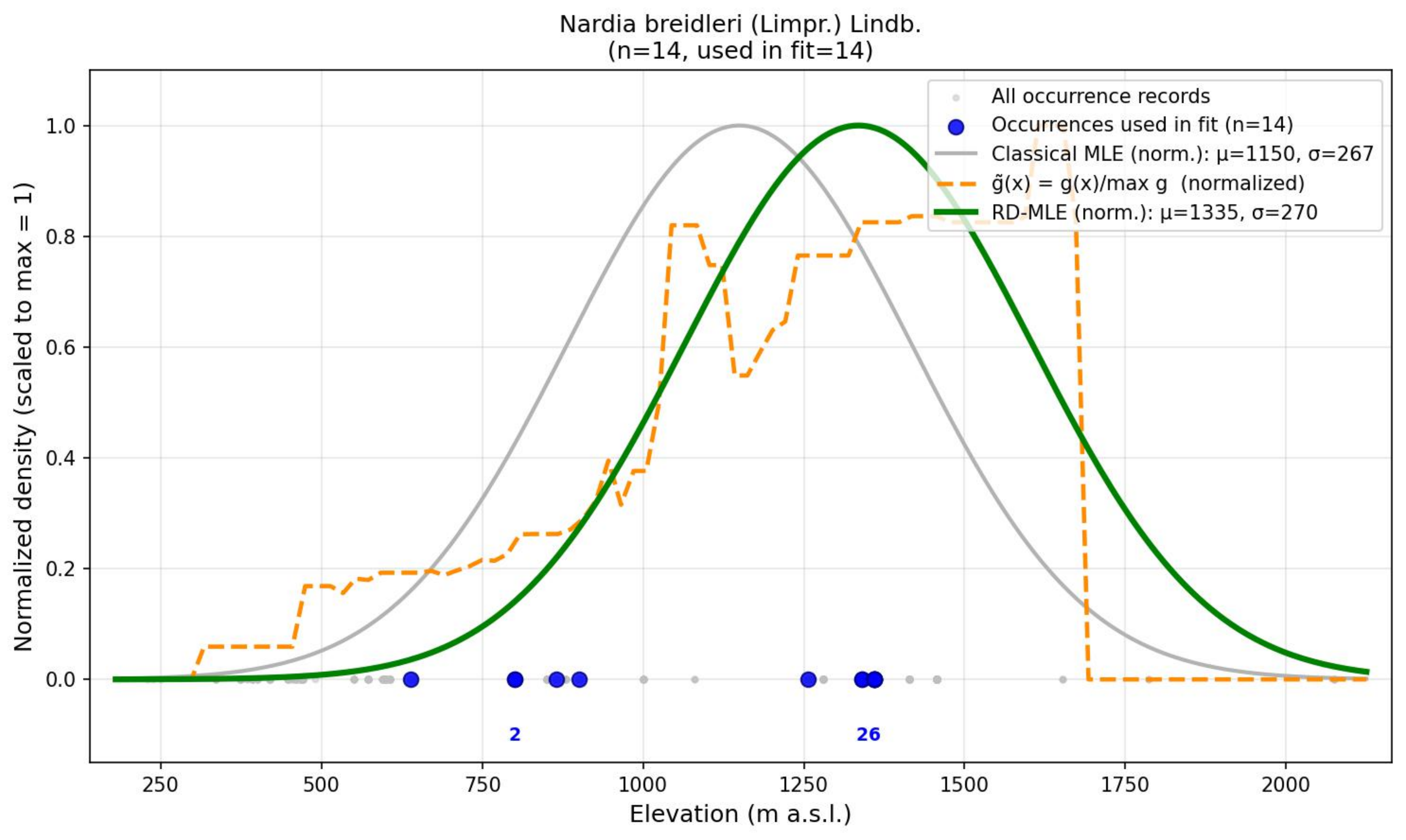

Nardia breidleri (Limpr.) Lindb.
(n=14, used in fit=14)
All occurrence records
Occurrences used in fit (n=14)
Classical MLE (norm.): μ=1150, σ=267
g̃(x) = g(x)/max g (normalized)
RD-MLE (norm.): μ=1335, σ=270
Normalized density (scaled to max = 1)
Elevation (m a.s.l.)
1.0
0.8
0.6
0.4
0.2
0.0
250
500
750
1000
1250
1500
1750
2000
2
26

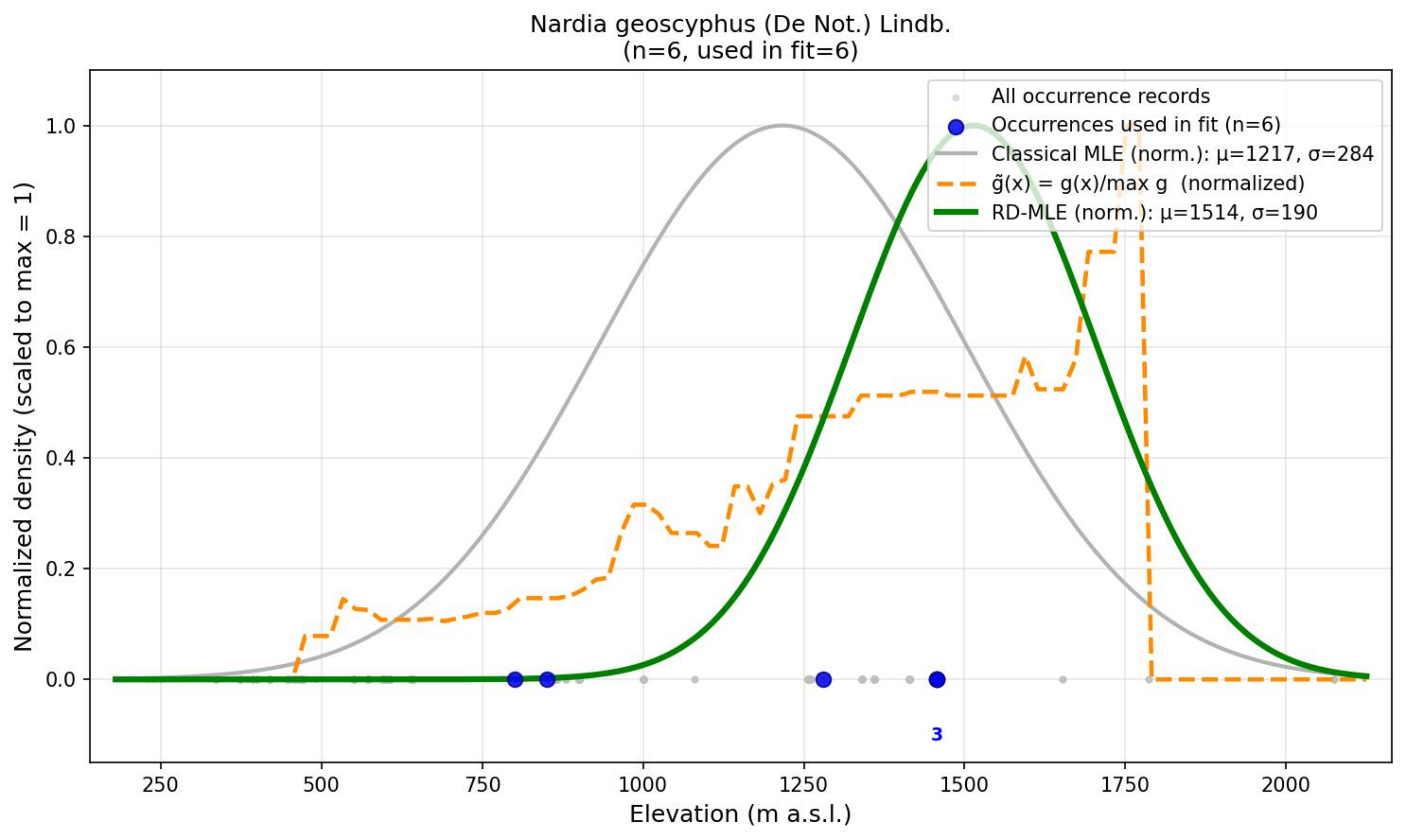

Nardia geoscyphus (De Not.) Lindb.
(n=6, used in fit=6)
All occurrence records
Occurrences used in fit (n=6)
Classical MLE (norm.): μ=1217, σ=284
g̃(x) = g(x)/max g (normalized)
RD-MLE (norm.): μ=1514, σ=190
Normalized density (scaled to max = 1)
Elevation (m a.s.l.)
0.0
0.2
0.4
0.6
0.8
1.0
250
500
750
1000
1250
1500
1750
2000
3

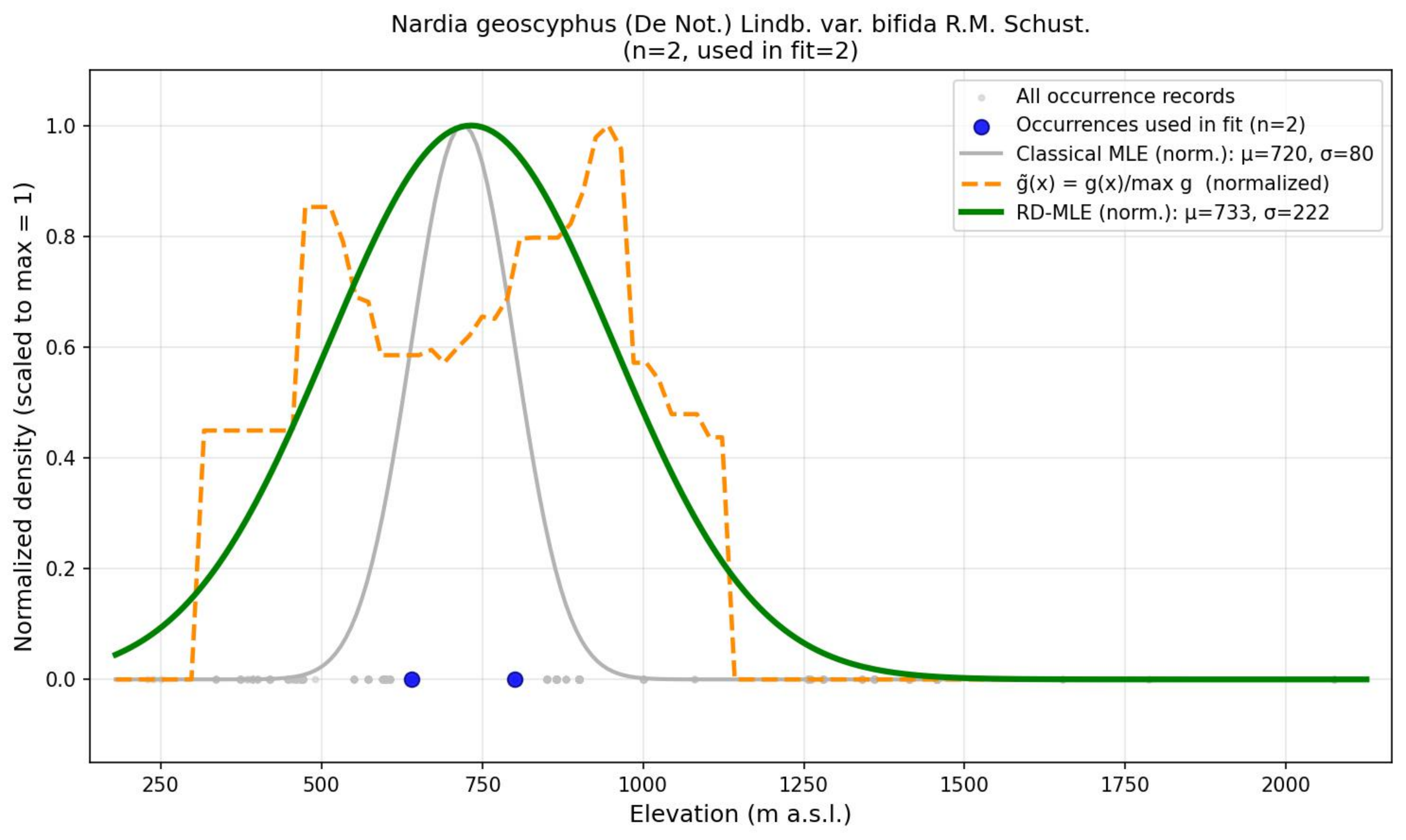
Nardia geoscyphus (De Not.) Lindb. var. bifida R.M. Schust.
(n=2, used in fit=2)
All occurrence records
Occurrences used in fit (n=2)
Classical MLE (norm.): μ=720, σ=80
g̃(x) = g(x)/max g (normalized)
RD-MLE (norm.): μ=733, σ=222
Normalized density (scaled to max = 1)
Elevation (m a.s.l.)
0.0
0.2
0.4
0.6
0.8
1.0
250
500
750
1000
1250
1500
1750
2000

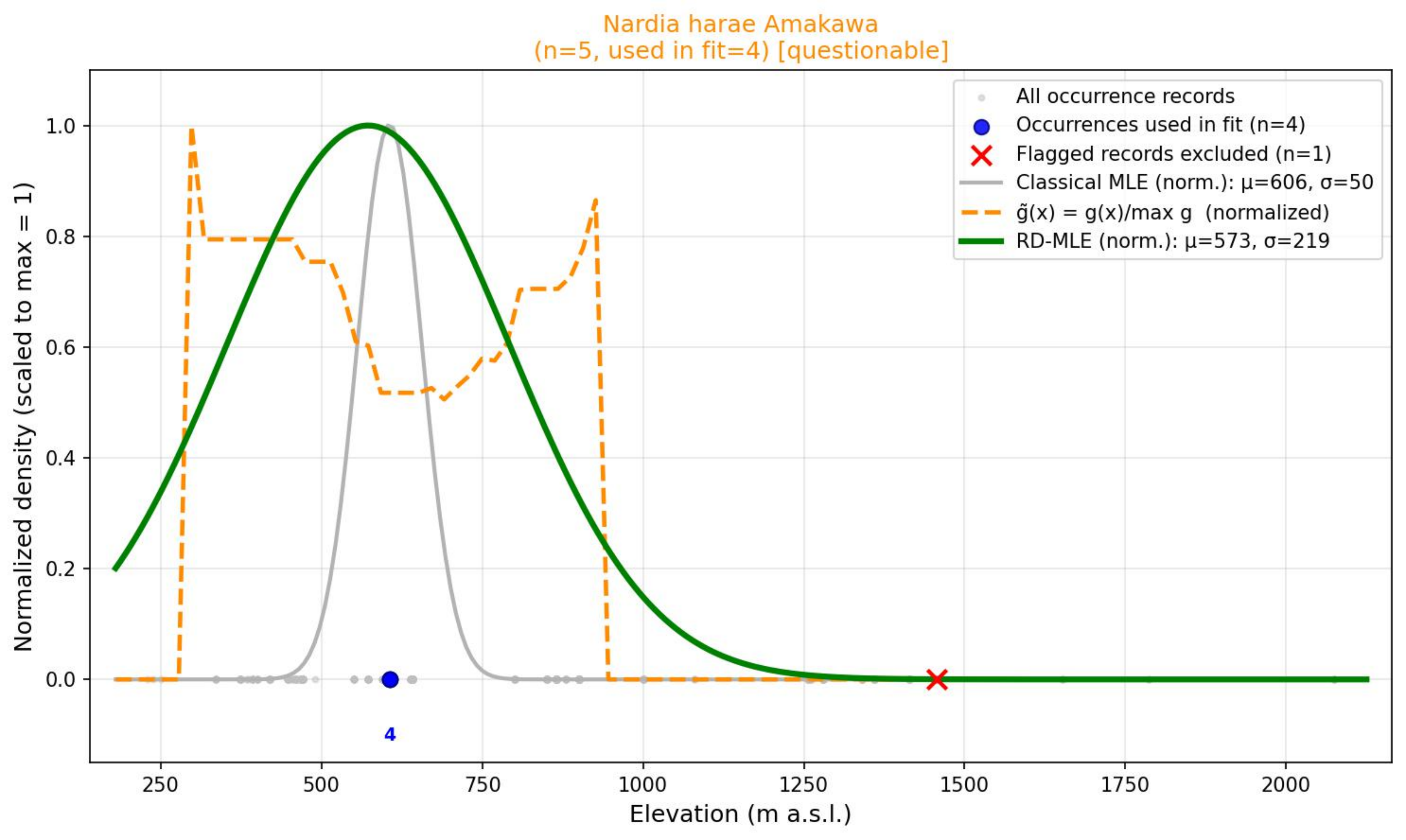

Nardia harae Amakawa
(n=5, used in fit=4) [questionable]
All occurrence records
Occurrences used in fit (n=4)
Flagged records excluded (n=1)
Classical MLE (norm.): μ=606, σ=50
g̃(x) = g(x)/max g (normalized)
RD-MLE (norm.): μ=573, σ=219
Normalized density (scaled to max = 1)
1.0
0.8
0.6
0.4
0.2
0.0
4
250
500
750
1000
1250
1500
1750
2000
Elevation (m a.s.l.)

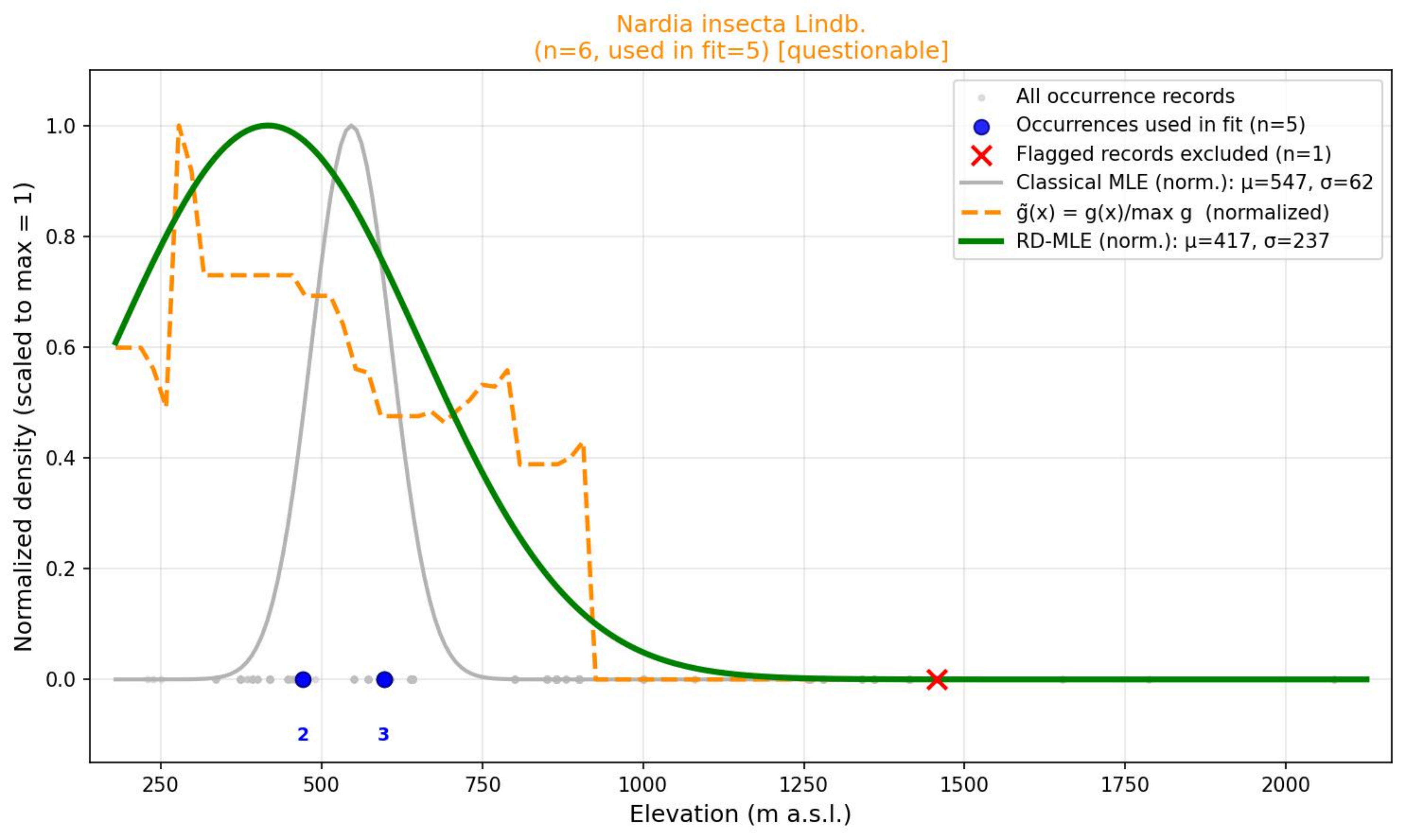

Nardia insecta Lindb.
(n=6, used in fit=5) [questionable]
All occurrence records
Occurrences used in fit (n=5)
Flagged records excluded (n=1)
Classical MLE (norm.): μ=547, σ=62
g̃(x) = g(x)/max g (normalized)
RD-MLE (norm.): μ=417, σ=237
Normalized density (scaled to max = 1)
Elevation (m a.s.l.)
1.0
0.8
0.6
0.4
0.2
0.0
250
500
750
1000
1250
1500
1750
2000
2
3

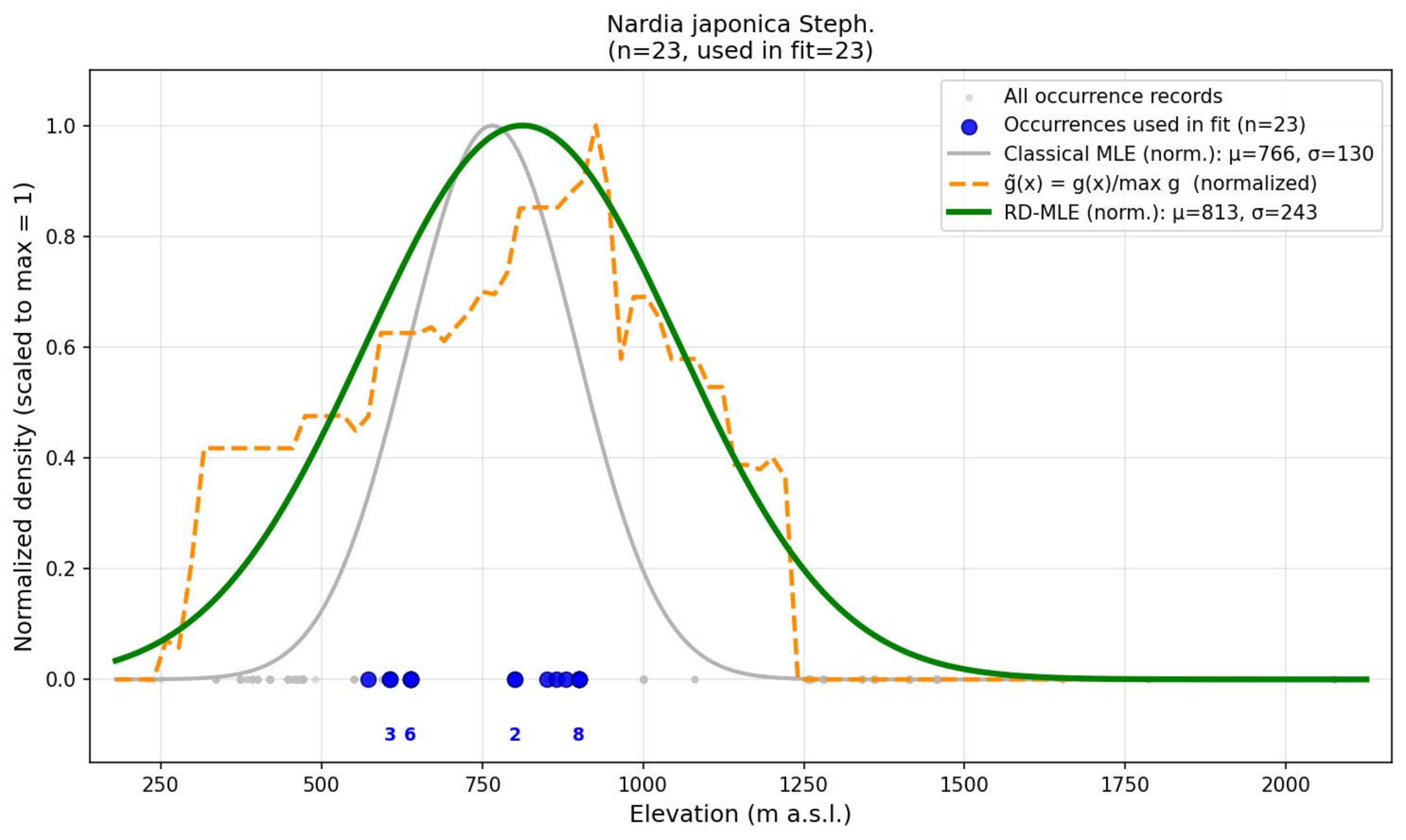

Nardia japonica Steph.
(n=23, used in fit=23)
All occurrence records
Occurrences used in fit (n=23)
Classical MLE (norm.): μ=766, σ=130
g̃(x) = g(x)/max g (normalized)
RD-MLE (norm.): μ=813, σ=243
Normalized density (scaled to max = 1)
Elevation (m a.s.l.)
1.0
0.8
0.6
0.4
0.2
0.0
250
500
750
1000
1250
1500
1750
2000
3
6
2
8

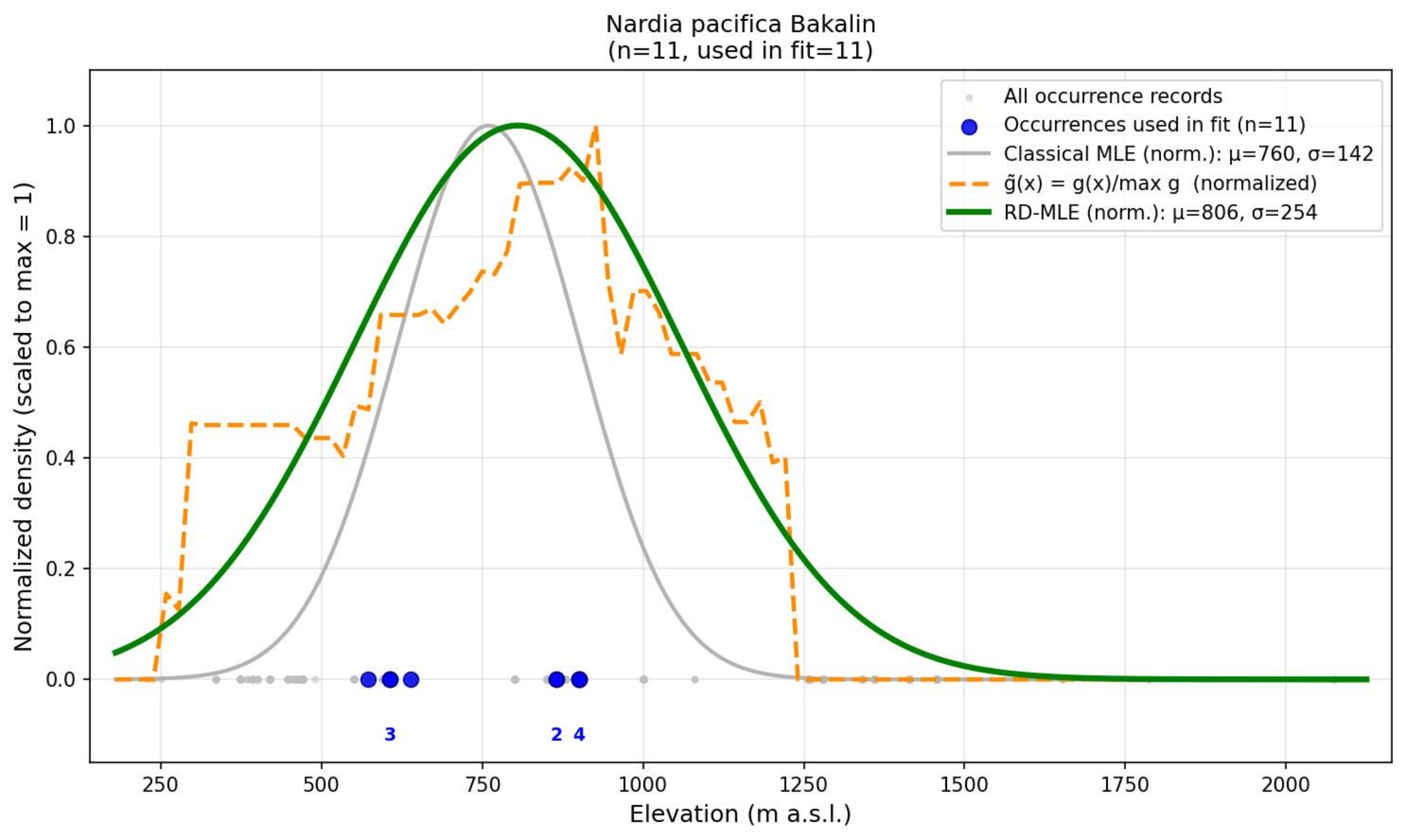

Nardia pacifica Bakalin
(n=11, used in fit=11)
All occurrence records
Occurrences used in fit (n=11)
Classical MLE (norm.): μ=760, σ=142
g̃(x) = g(x)/max g (normalized)
RD-MLE (norm.): μ=806, σ=254
Normalized density (scaled to max = 1)
Elevation (m a.s.l.)
1.0
0.8
0.6
0.4
0.2
0.0
250
500
750
1000
1250
1500
1750
2000
3
2
4

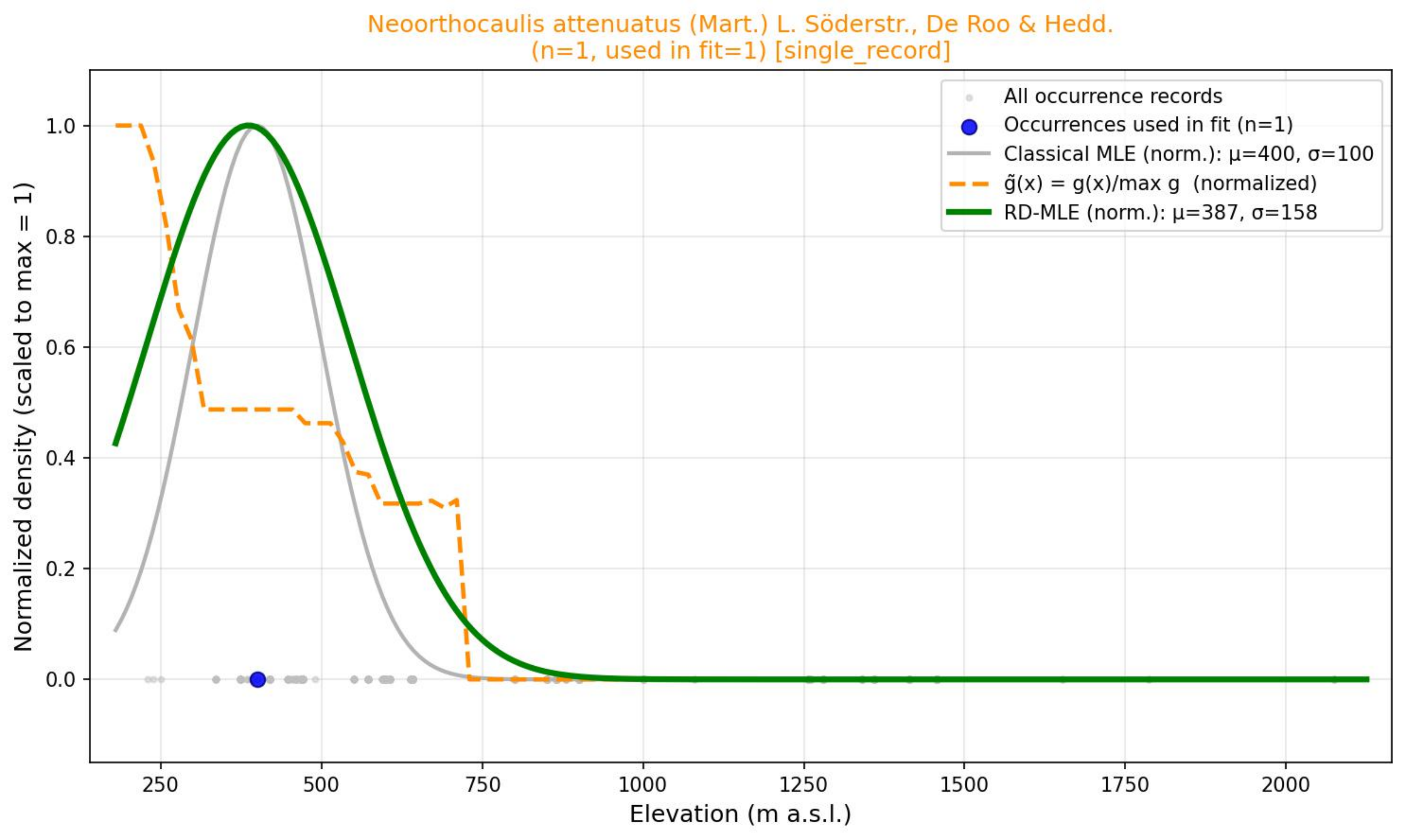
Neoorthocaulis attenuatus (Mart.) L. Söderstr., De Roo & Hedd.
(n=1, used in fit=1) [single_record]
All occurrence records
Occurrences used in fit (n=1)
Classical MLE (norm.): μ=400, σ=100
g̃(x) = g(x)/max g (normalized)
RD-MLE (norm.): μ=387, σ=158
Normalized density (scaled to max = 1)
Elevation (m a.s.l.)
0.0
0.2
0.4
0.6
0.8
1.0
250
500
750
1000
1250
1500
1750
2000

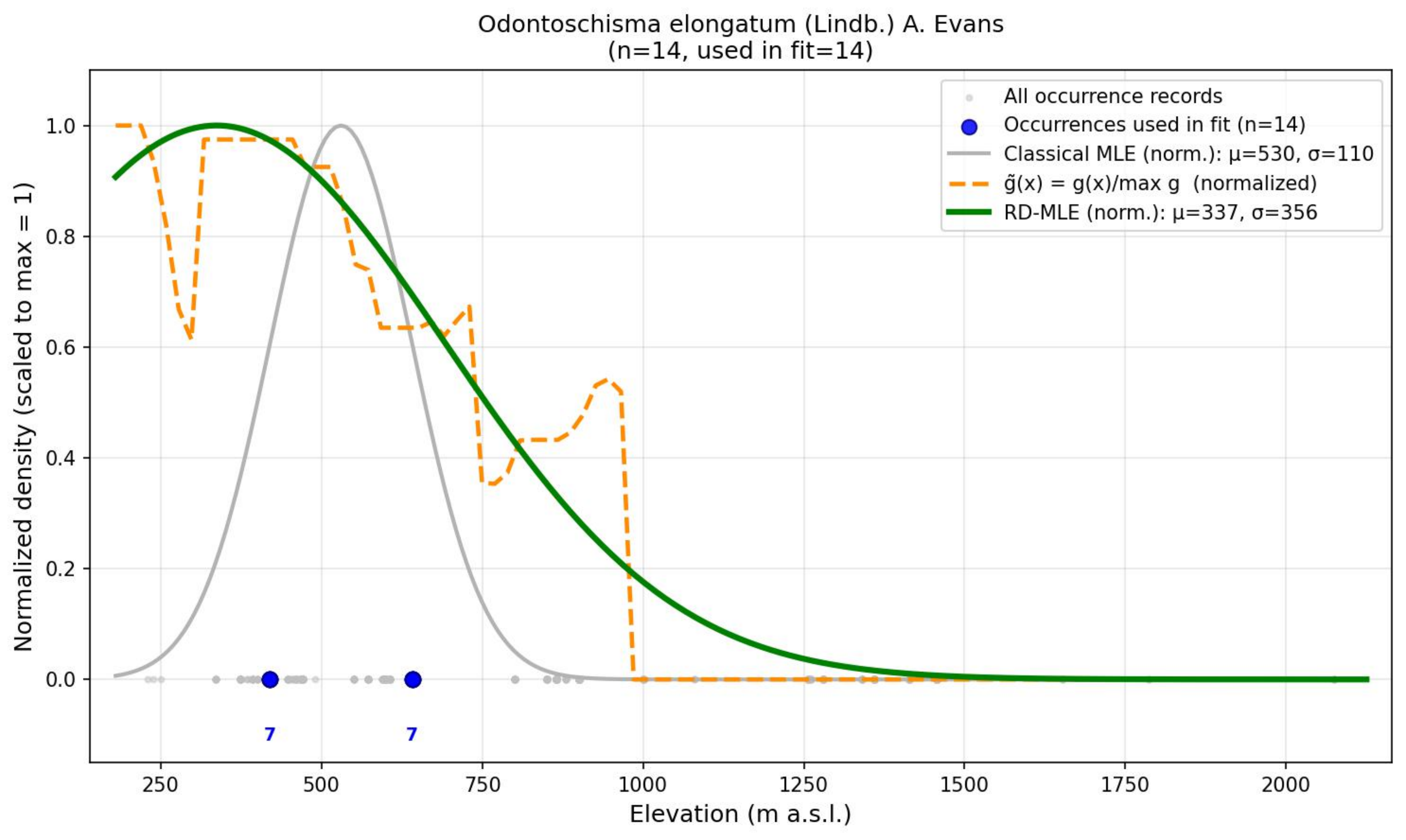
Odontoschisma elongatum (Lindb.) A. Evans
(n=14, used in fit=14)
All occurrence records
Occurrences used in fit (n=14)
Classical MLE (norm.): μ=530, σ=110
g̃(x) = g(x)/max g (normalized)
RD-MLE (norm.): μ=337, σ=356
Normalized density (scaled to max = 1)
Elevation (m a.s.l.)
1.0
0.8
0.6
0.4
0.2
0.0
250
500
750
1000
1250
1500
1750
2000
7
7

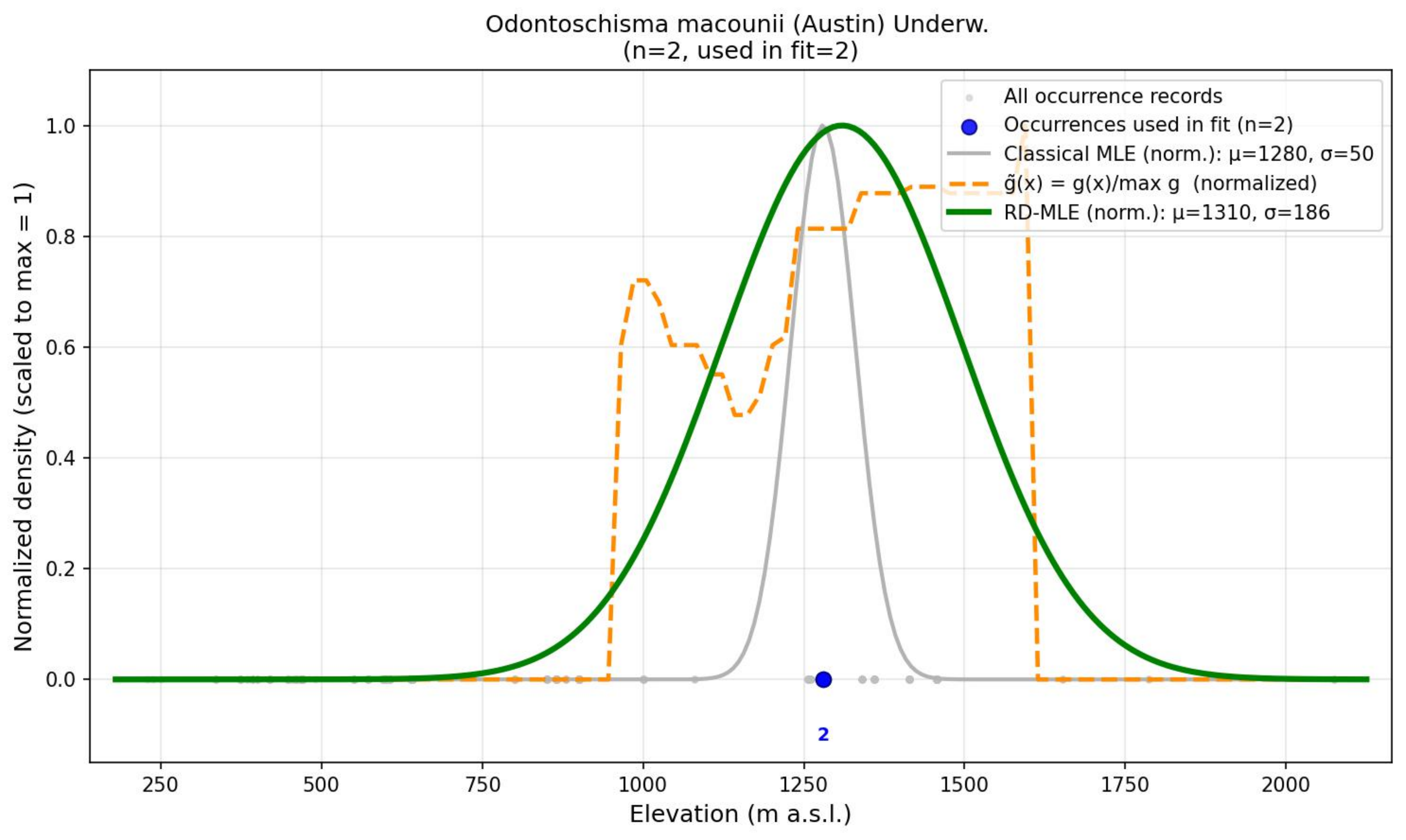
Odontoschisma macounii (Austin) Underw.
(n=2, used in fit=2)
All occurrence records
Occurrences used in fit (n=2)
Classical MLE (norm.): μ=1280, σ=50
g̃(x) = g(x)/max g (normalized)
RD-MLE (norm.): μ=1310, σ=186
Normalized density (scaled to max = 1)
Elevation (m a.s.l.)
2
1.0
0.8
0.6
0.4
0.2
0.0
250
500
750
1000
1250
1500
1750
2000

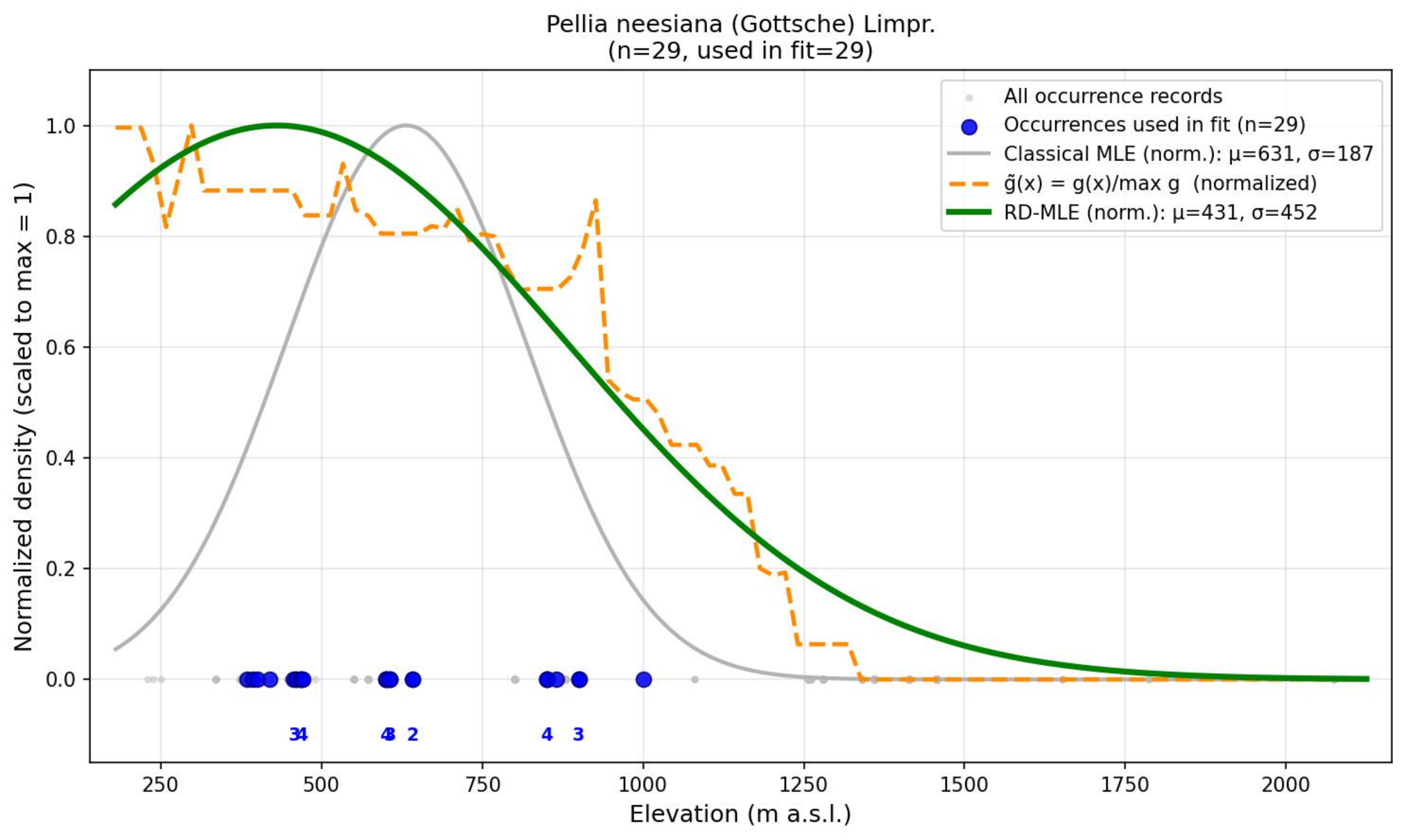

Pellia neesiana (Gottsche) Limpr.
(n=29, used in fit=29)
All occurrence records
Occurrences used in fit (n=29)
Classical MLE (norm.): μ=631, σ=187
g̃(x) = g(x)/max g (normalized)
RD-MLE (norm.): μ=431, σ=452
Normalized density (scaled to max = 1)
Elevation (m a.s.l.)
0.0
0.2
0.4
0.6
0.8
1.0
250
500
750
1000
1250
1500
1750
2000

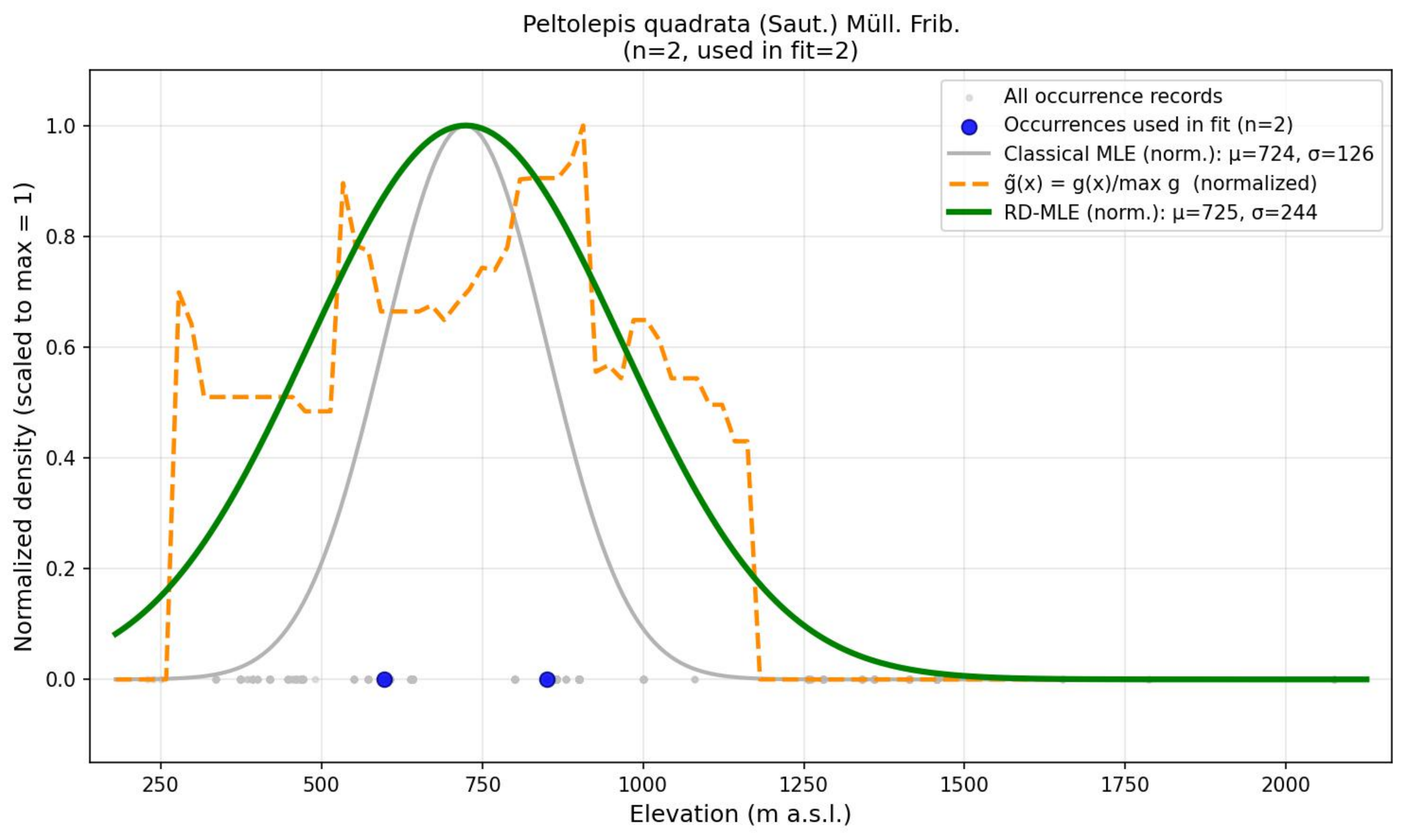
Peltolepis quadrata (Saut.) Müll. Frib.
(n=2, used in fit=2)
All occurrence records
Occurrences used in fit (n=2)
Classical MLE (norm.): μ=724, σ=126
g̃(x) = g(x)/max g (normalized)
RD-MLE (norm.): μ=725, σ=244
Normalized density (scaled to max = 1)
Elevation (m a.s.l.)
0.0
0.2
0.4
0.6
0.8
1.0
250
500
750
1000
1250
1500
1750
2000

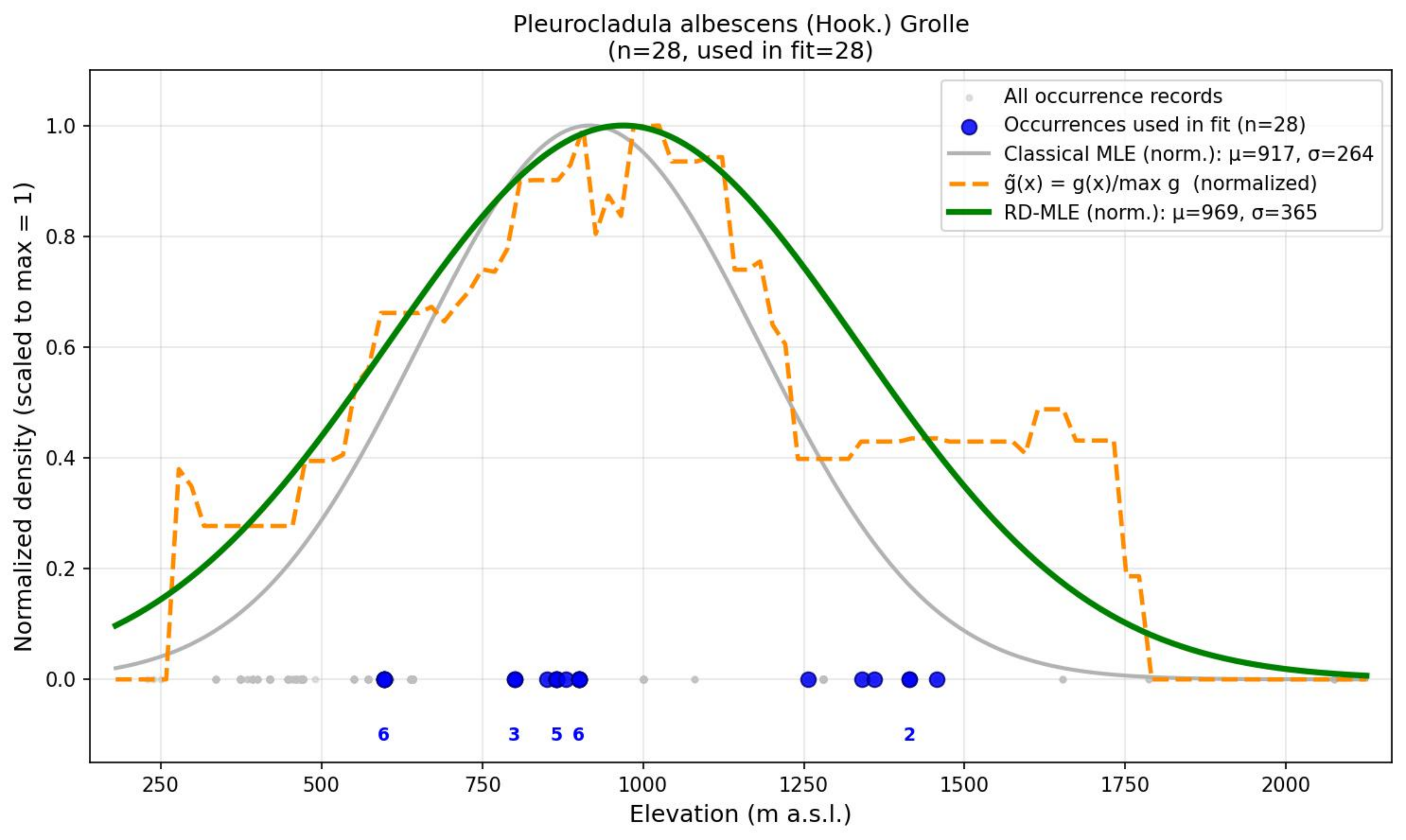

Pleurocladula albescens (Hook.) Grolle
(n=28, used in fit=28)
All occurrence records
Occurrences used in fit (n=28)
Classical MLE (norm.): μ=917, σ=264
g̃(x) = g(x)/max g (normalized)
RD-MLE (norm.): μ=969, σ=365
Normalized density (scaled to max = 1)
Elevation (m a.s.l.)
0.0
0.2
0.4
0.6
0.8
1.0
250
500
750
1000
1250
1500
1750
2000
6
3
5
6
2

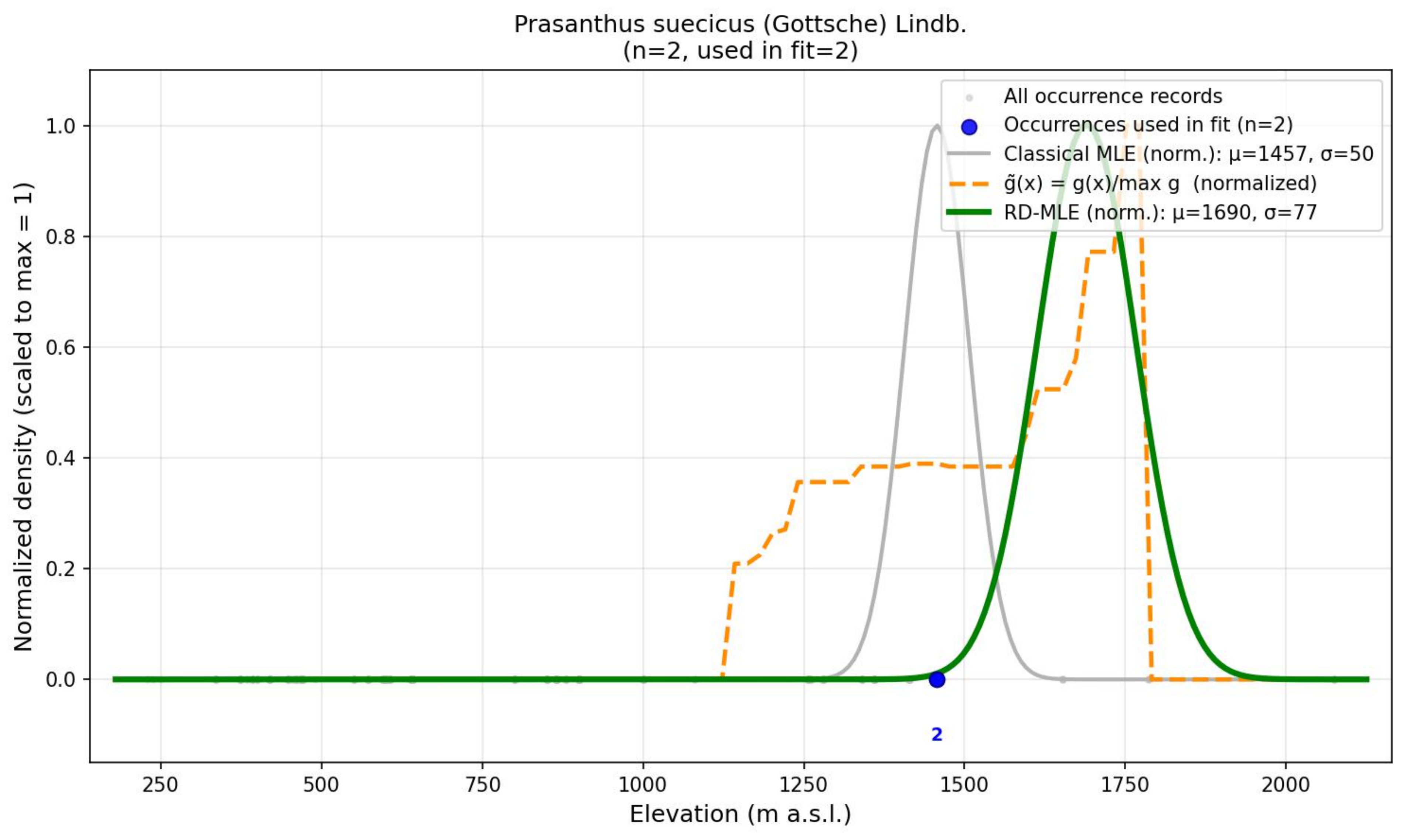

Prasanthus suecicus (Gottsche) Lindb.
(n=2, used in fit=2)
All occurrence records
Occurrences used in fit (n=2)
Classical MLE (norm.): μ=1457, σ=50
g̃(x) = g(x)/max g (normalized)
RD-MLE (norm.): μ=1690, σ=77
Normalized density (scaled to max = 1)
Elevation (m a.s.l.)
0.0
0.2
0.4
0.6
0.8
1.0
250
500
750
1000
1250
1500
1750
2000
2

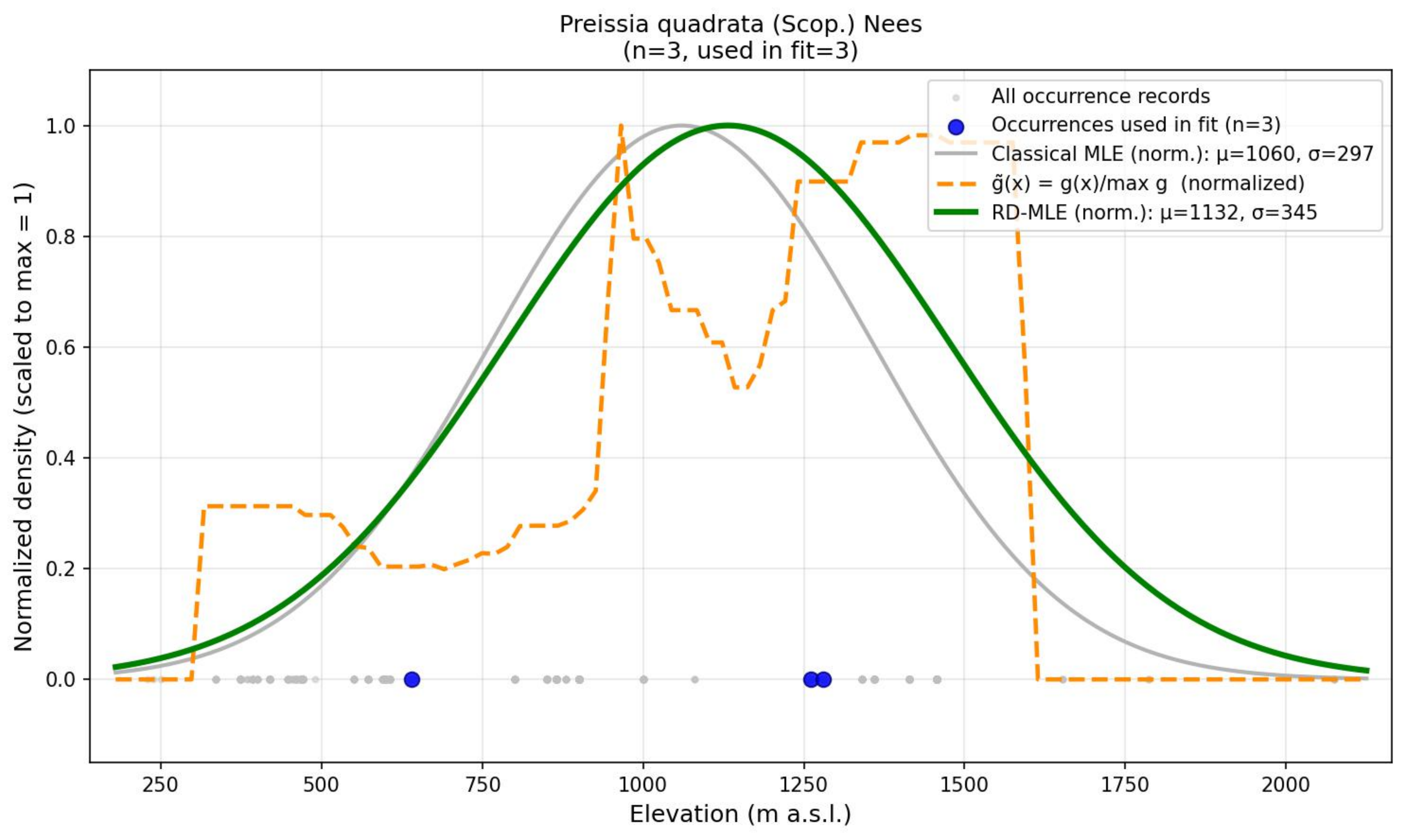

Preissia quadrata (Scop.) Nees
(n=3, used in fit=3)
All occurrence records
Occurrences used in fit (n=3)
Classical MLE (norm.): μ=1060, σ=297
g̃(x) = g(x)/max g (normalized)
RD-MLE (norm.): μ=1132, σ=345
Normalized density (scaled to max = 1)
Elevation (m a.s.l.)
0.0
0.2
0.4
0.6
0.8
1.0
250
500
750
1000
1250
1500
1750
2000

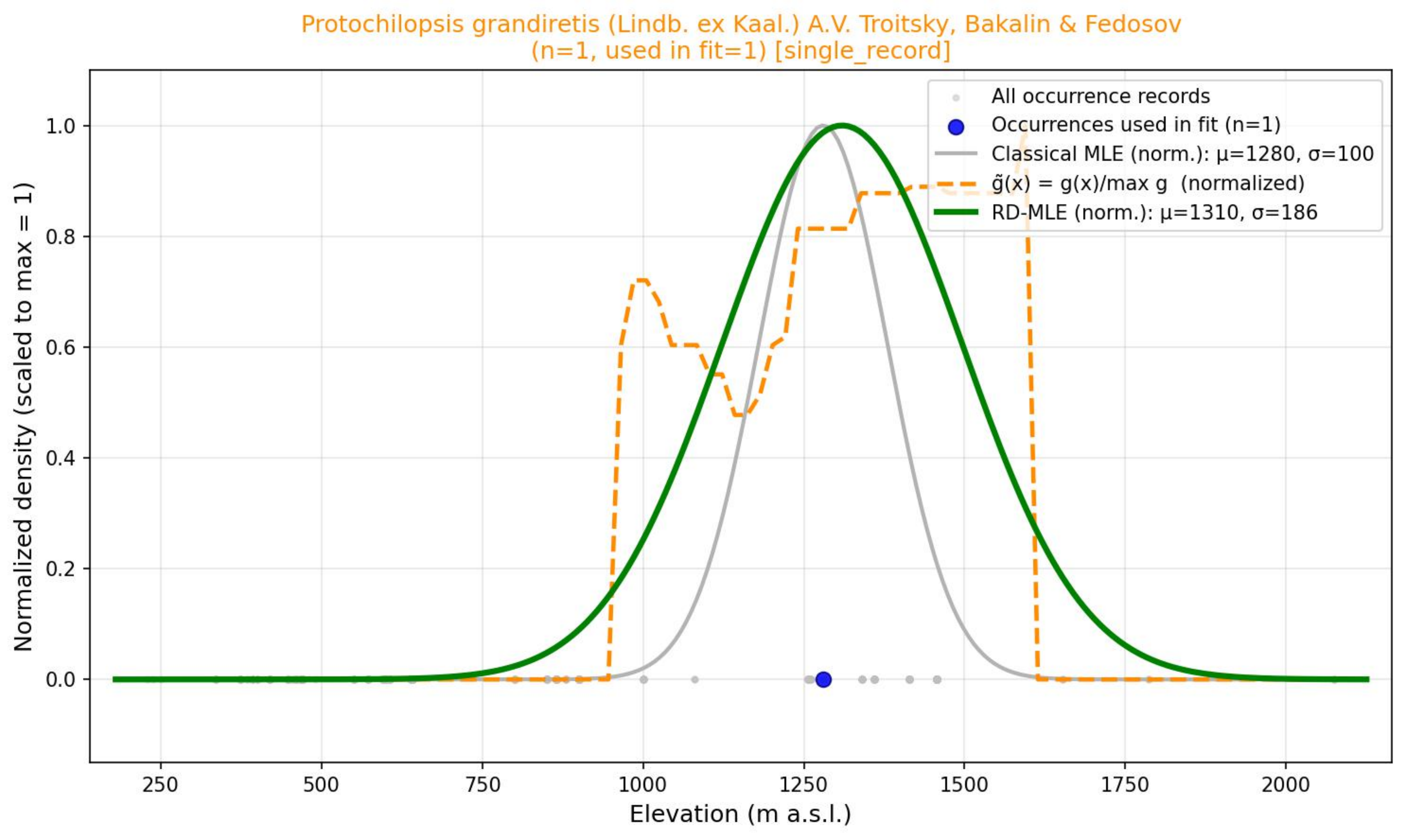

Protochilopsis grandiretis (Lindb. ex Kaal.) A.V. Troitsky, Bakalin & Fedosov
(n=1, used in fit=1) [single_record]
All occurrence records
Occurrences used in fit (n=1)
Classical MLE (norm.): μ=1280, σ=100
g̃(x) = g(x)/max g (normalized)
RD-MLE (norm.): μ=1310, σ=186
Normalized density (scaled to max = 1)
Elevation (m a.s.l.)
0.0
0.2
0.4
0.6
0.8
1.0
250
500
750
1000
1250
1500
1750
2000

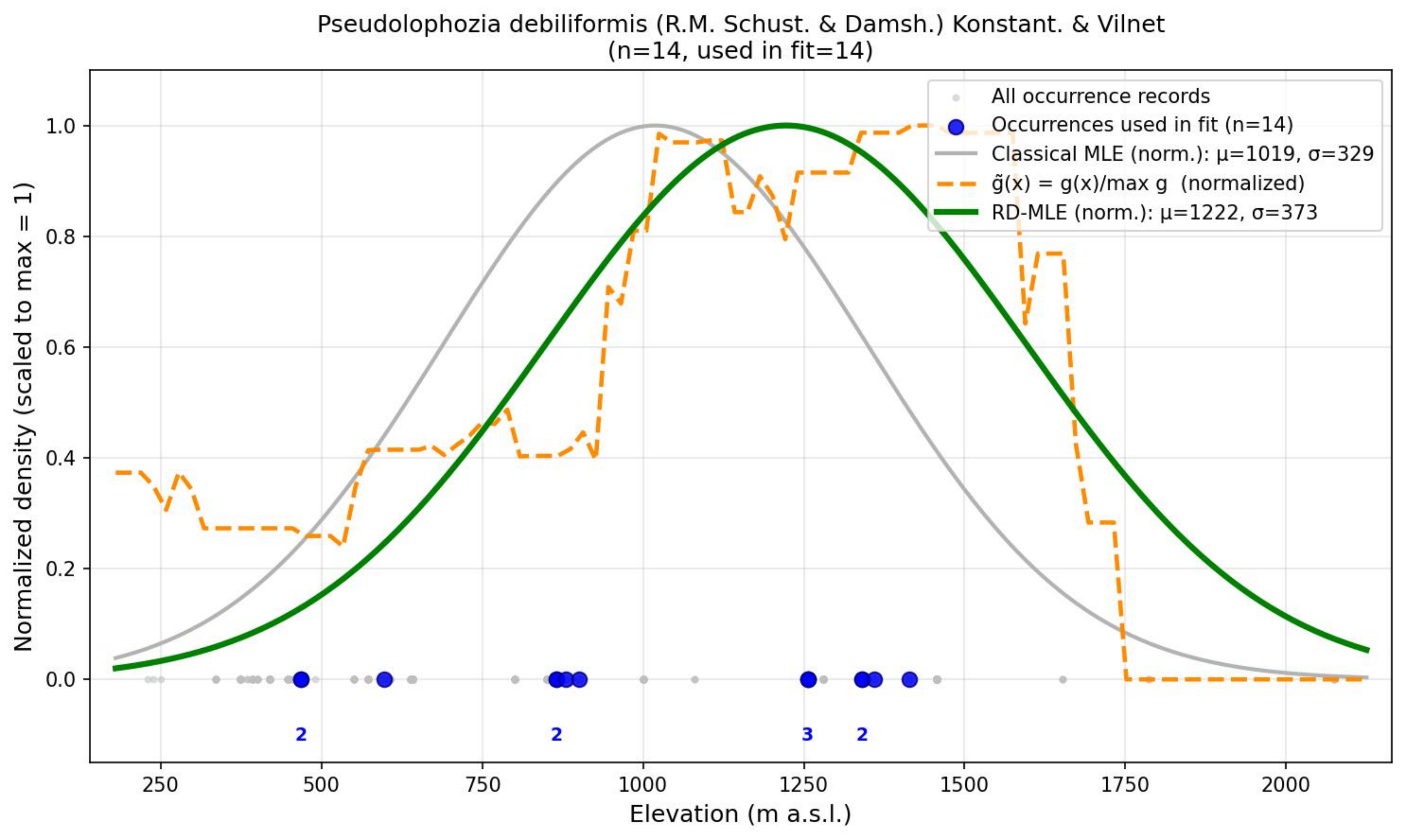

Pseudolophozia debiliformis (R.M. Schust. & Damsh.) Konstant. & Vilnet
(n=14, used in fit=14)
All occurrence records
Occurrences used in fit (n=14)
Classical MLE (norm.): μ=1019, σ=329
g̃(x) = g(x)/max g (normalized)
RD-MLE (norm.): μ=1222, σ=373
Normalized density (scaled to max = 1)
Elevation (m a.s.l.)
0.0
0.2
0.4
0.6
0.8
1.0
250
500
750
1000
1250
1500
1750
2000
2
2
3
2

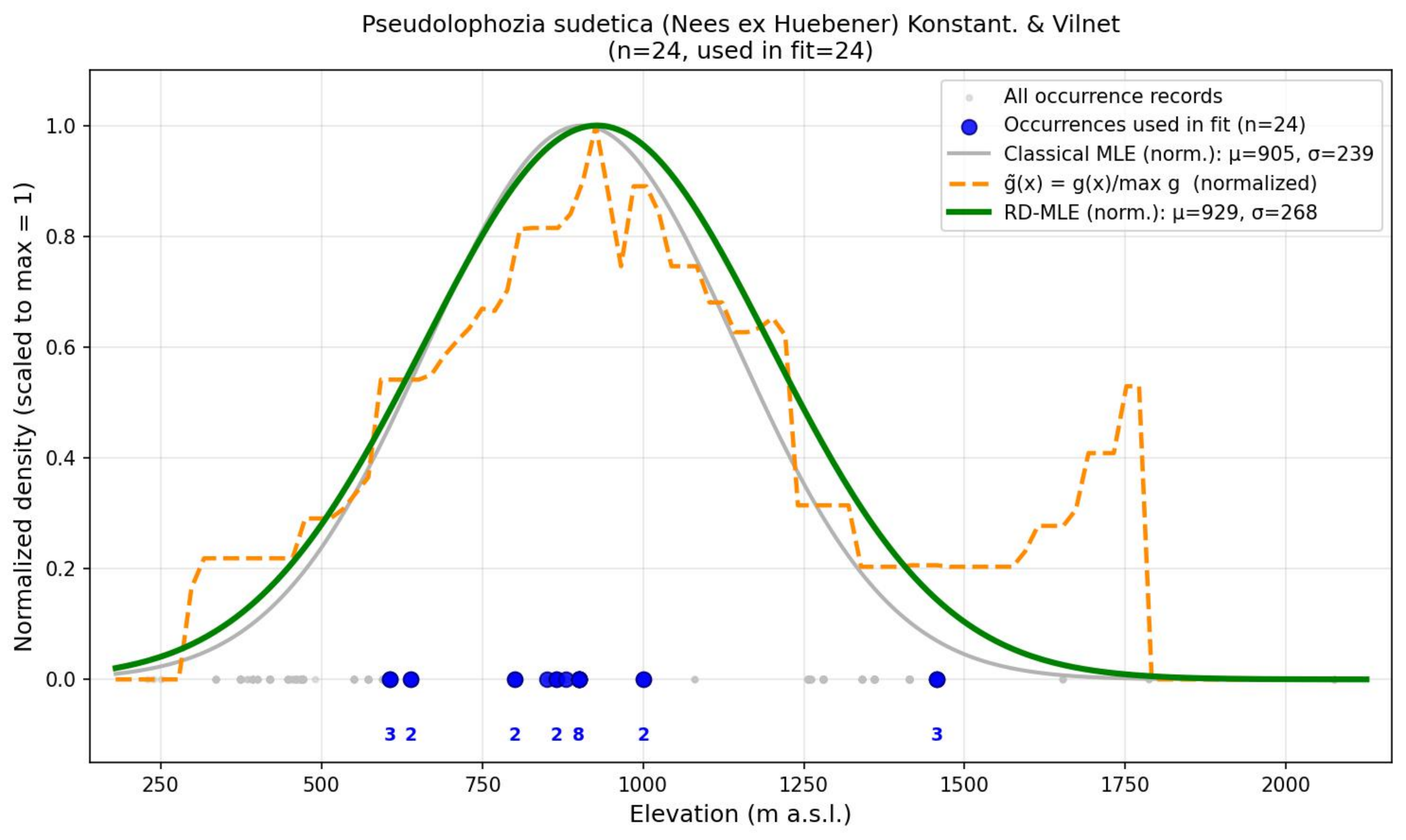

Pseudolophozia sudetica (Nees ex Huebener) Konstant. & Vilnet
(n=24, used in fit=24)
All occurrence records
Occurrences used in fit (n=24)
Classical MLE (norm.): μ=905, σ=239
g̃(x) = g(x)/max g (normalized)
RD-MLE (norm.): μ=929, σ=268
Normalized density (scaled to max = 1)
Elevation (m a.s.l.)
0.0
0.2
0.4
0.6
0.8
1.0
250
500
750
1000
1250
1500
1750
2000
3 2 2 2 8 2 3

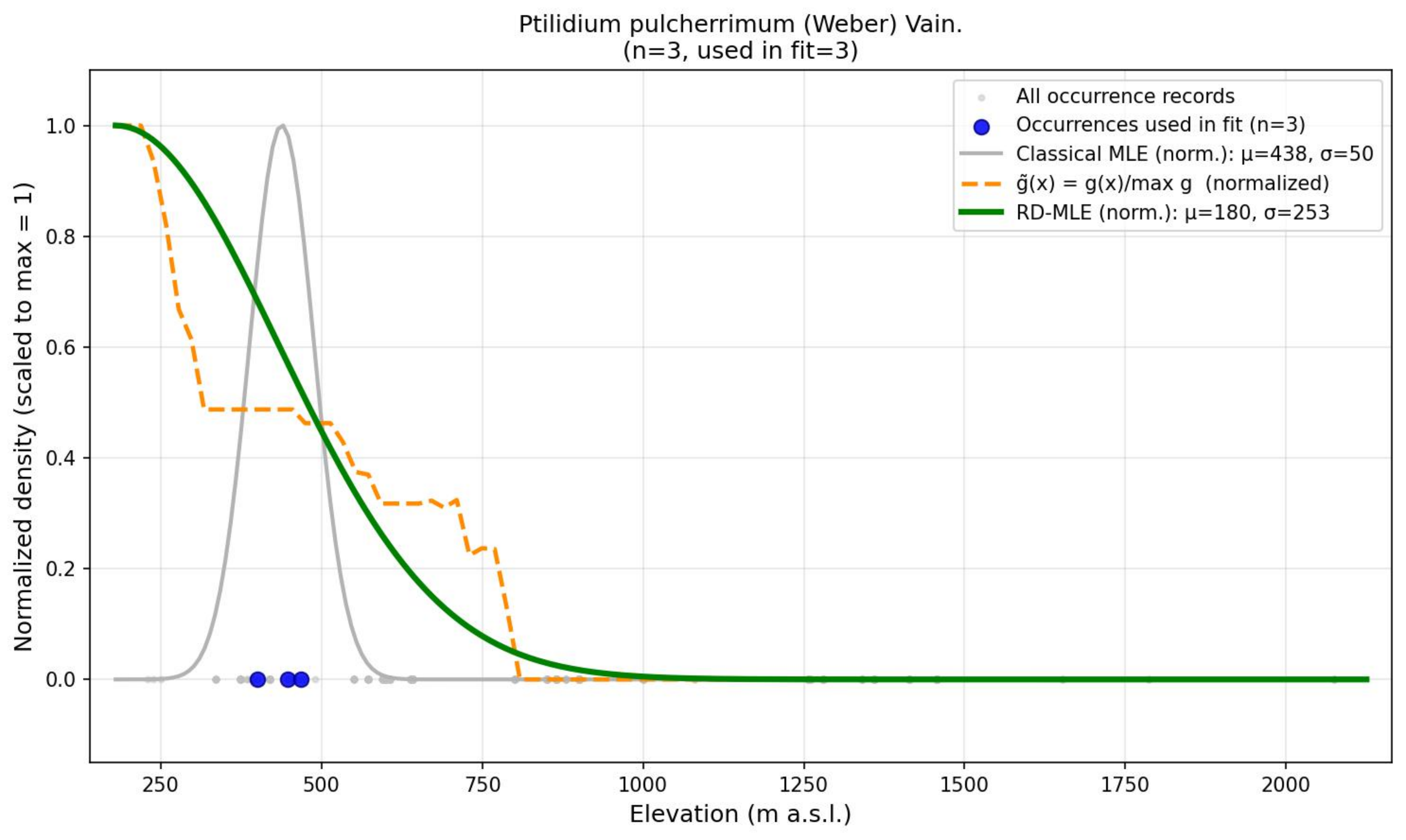

Ptilidium pulcherrimum (Weber) Vain.
(n=3, used in fit=3)
All occurrence records
Occurrences used in fit (n=3)
Classical MLE (norm.): μ=438, σ=50
g̃(x) = g(x)/max g (normalized)
RD-MLE (norm.): μ=180, σ=253
Normalized density (scaled to max = 1)
Elevation (m a.s.l.)
0.0
0.2
0.4
0.6
0.8
1.0
250
500
750
1000
1250
1500
1750
2000

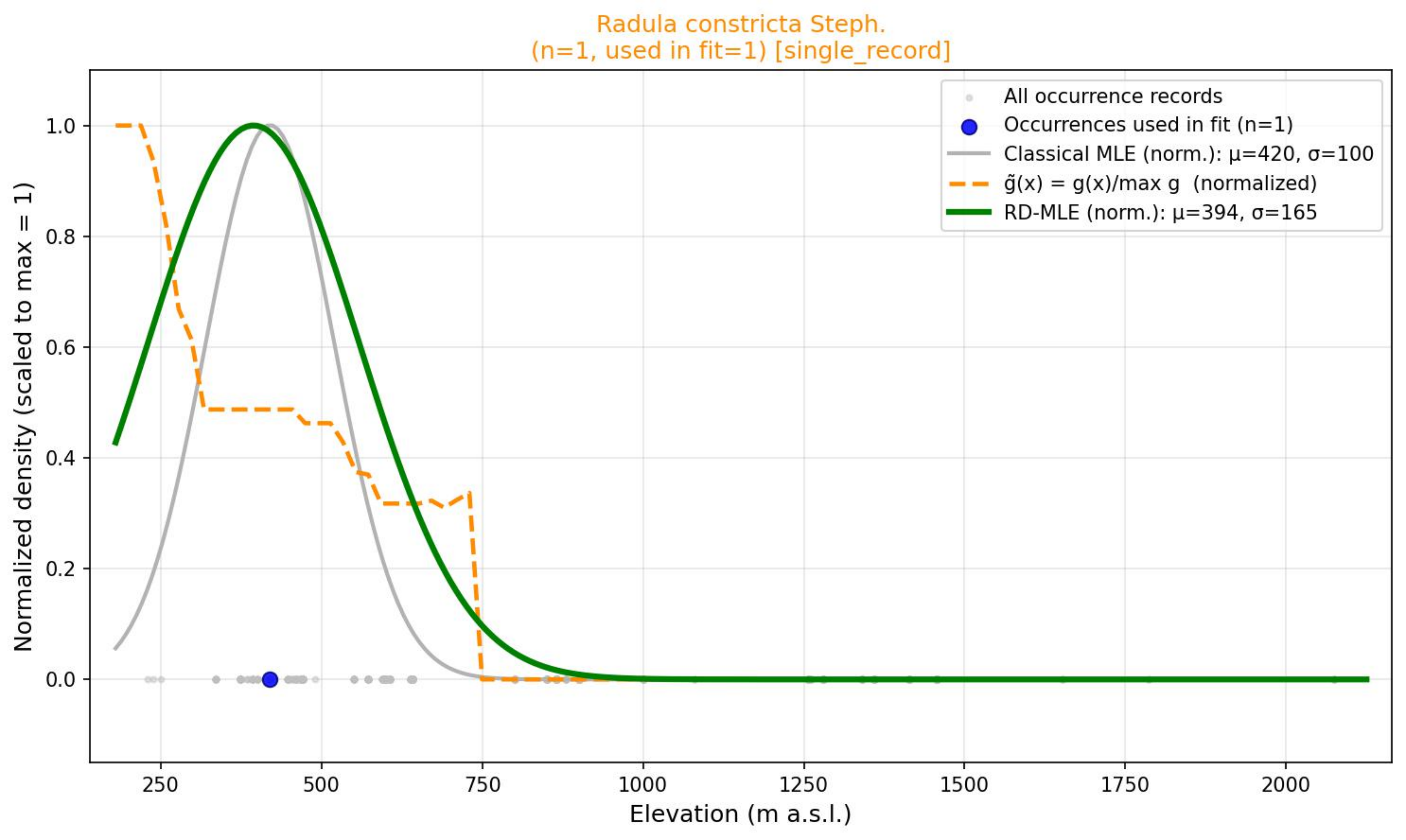

Radula constricta Steph.
(n=1, used in fit=1) [single_record]
All occurrence records
Occurrences used in fit (n=1)
Classical MLE (norm.): μ=420, σ=100
g̃(x) = g(x)/max g (normalized)
RD-MLE (norm.): μ=394, σ=165
Normalized density (scaled to max = 1)
Elevation (m a.s.l.)
0.0
0.2
0.4
0.6
0.8
1.0
250
500
750
1000
1250
1500
1750
2000

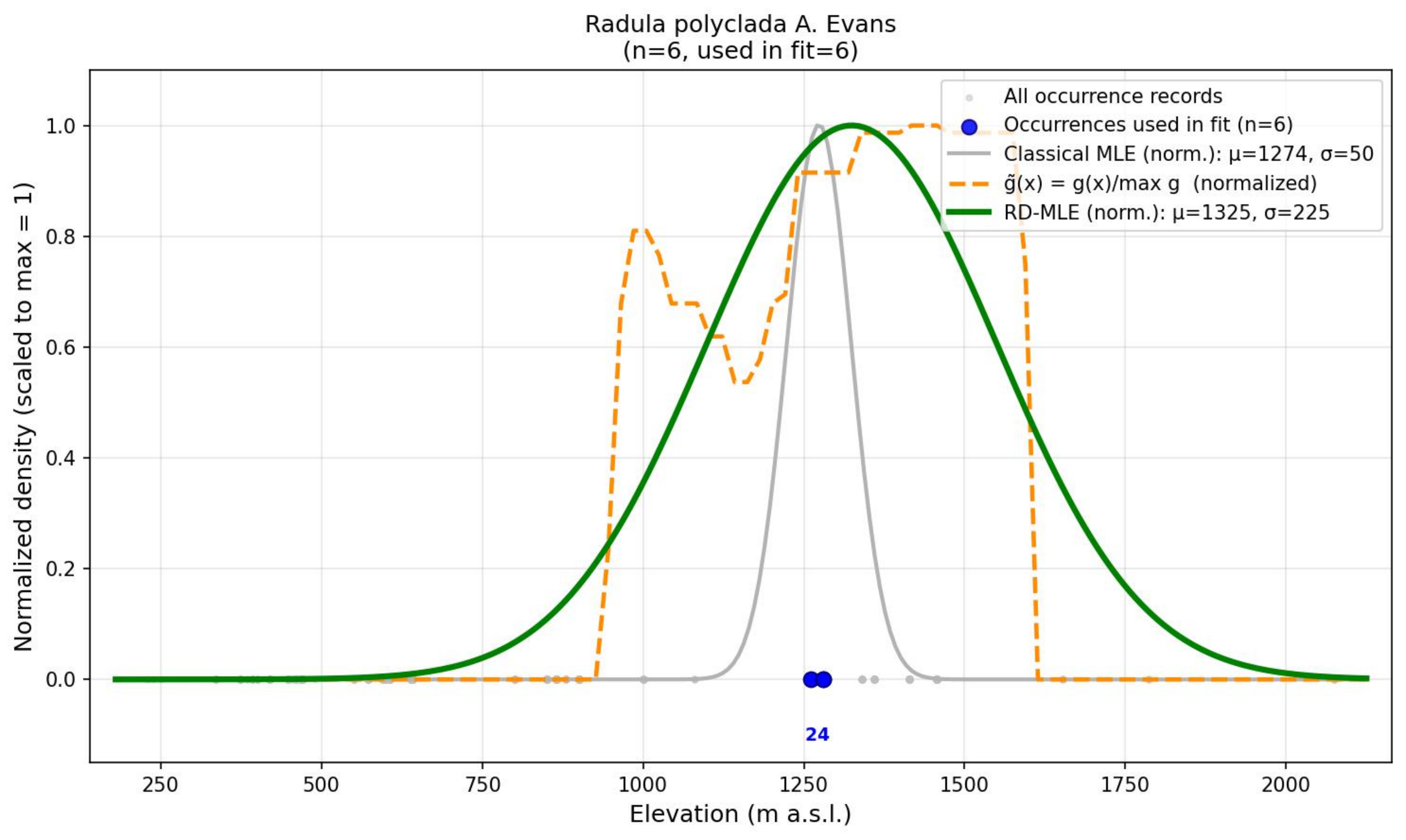

Radula polyclada A. Evans
(n=6, used in fit=6)
All occurrence records
Occurrences used in fit (n=6)
Classical MLE (norm.): μ=1274, σ=50
g̃(x) = g(x)/max g (normalized)
RD-MLE (norm.): μ=1325, σ=225
Normalized density (scaled to max = 1)
1.0
0.8
0.6
0.4
0.2
0.0
24
250
500
750
1000
1250
1500
1750
2000
Elevation (m a.s.l.)

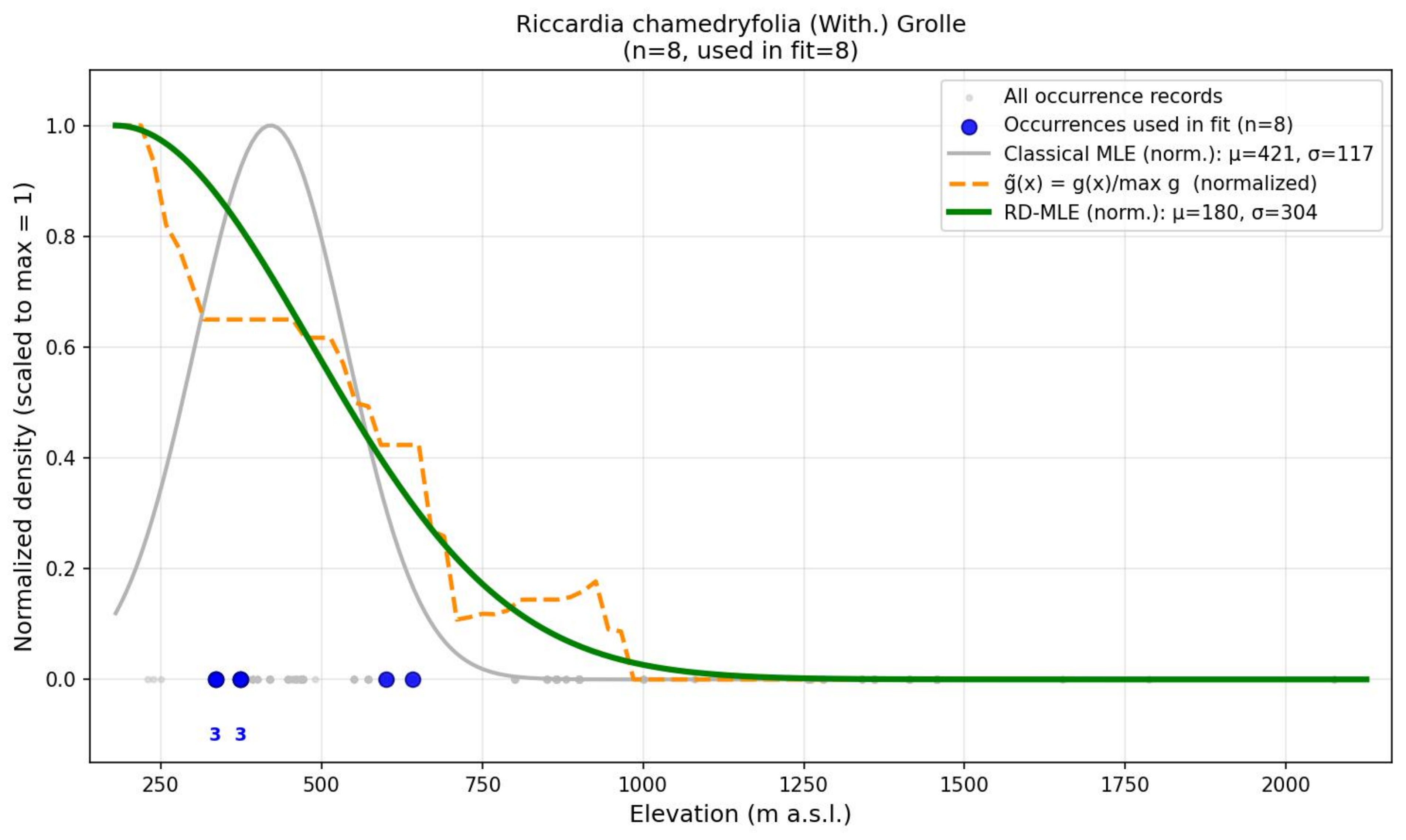
Riccardia chamedryfolia (With.) Grolle
(n=8, used in fit=8)
All occurrence records
Occurrences used in fit (n=8)
Classical MLE (norm.): μ=421, σ=117
g̃(x) = g(x)/max g (normalized)
RD-MLE (norm.): μ=180, σ=304
Normalized density (scaled to max = 1)
Elevation (m a.s.l.)
1.0
0.8
0.6
0.4
0.2
0.0
250
500
750
1000
1250
1500
1750
2000
3
3

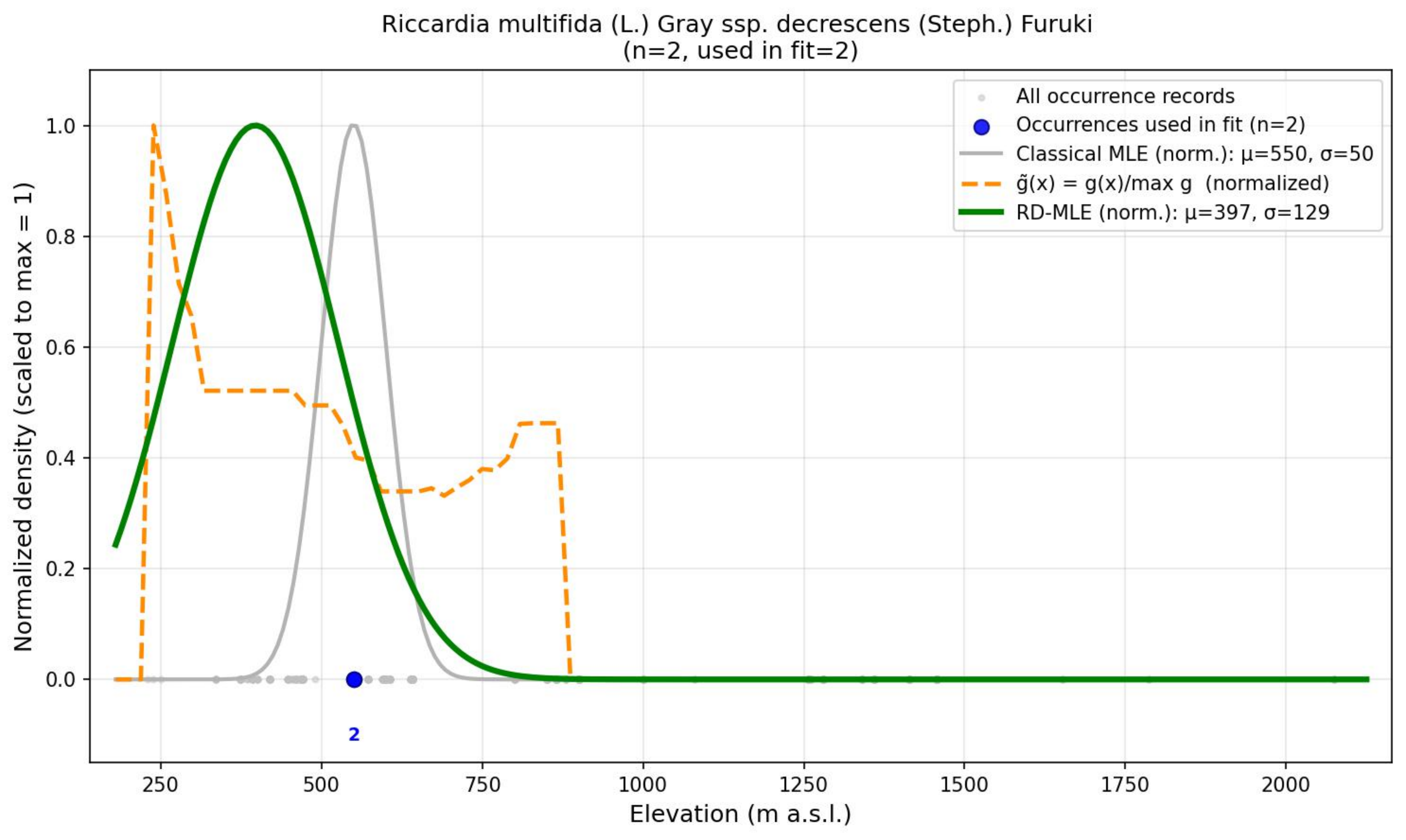

Riccardia multifida (L.) Gray ssp. decrescens (Steph.) Furuki
(n=2, used in fit=2)
All occurrence records
Occurrences used in fit (n=2)
Classical MLE (norm.): μ=550, σ=50
g̃(x) = g(x)/max g (normalized)
RD-MLE (norm.): μ=397, σ=129
Normalized density (scaled to max = 1)
Elevation (m a.s.l.)
0.0
0.2
0.4
0.6
0.8
1.0
250
500
750
1000
1250
1500
1750
2000
2

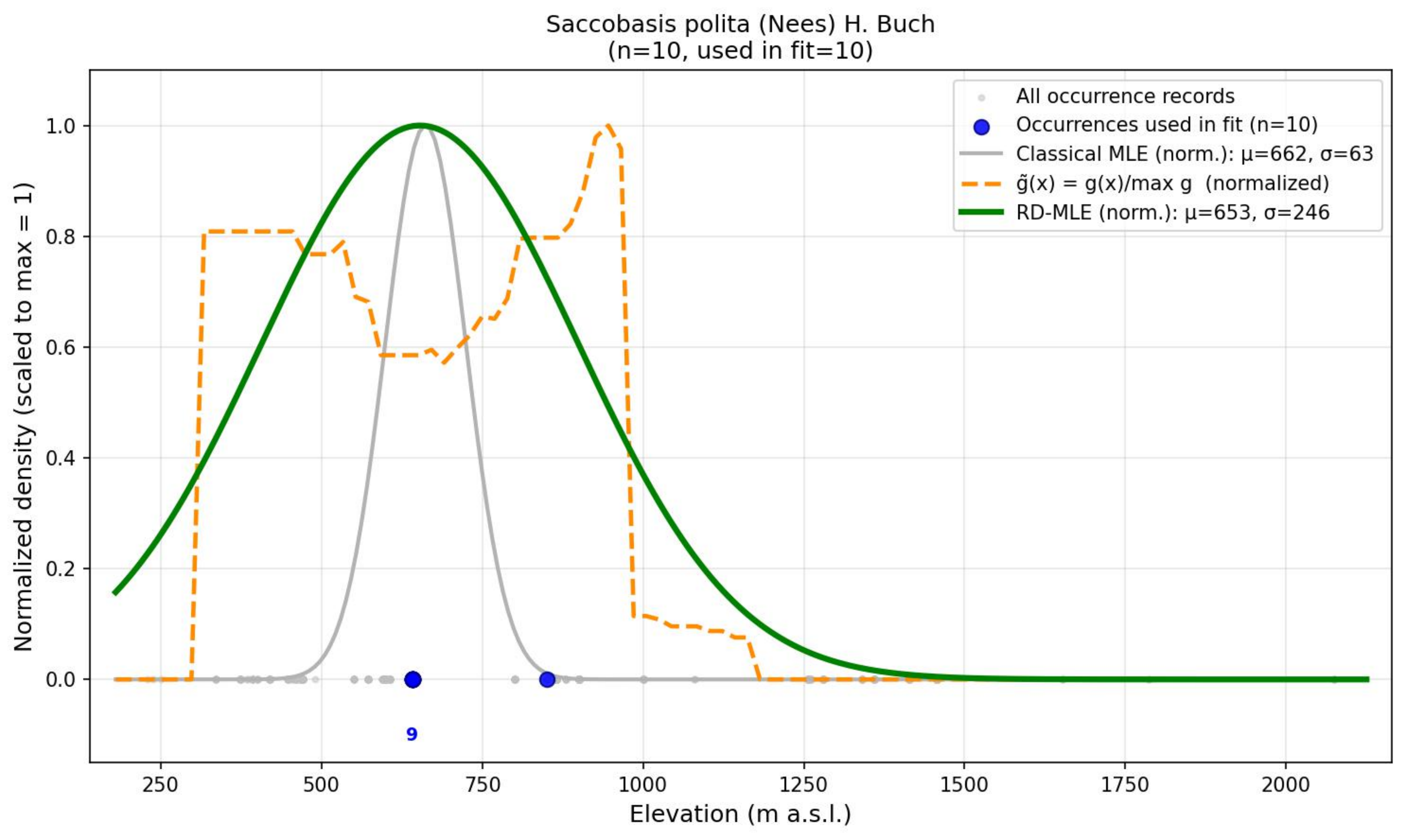

Saccobasis polita (Nees) H. Buch
(n=10, used in fit=10)
All occurrence records
Occurrences used in fit (n=10)
Classical MLE (norm.): μ=662, σ=63
g̃(x) = g(x)/max g (normalized)
RD-MLE (norm.): μ=653, σ=246
Normalized density (scaled to max = 1)
1.0
0.8
0.6
0.4
0.2
0.0
9
250
500
750
1000
1250
1500
1750
2000
Elevation (m a.s.l.)

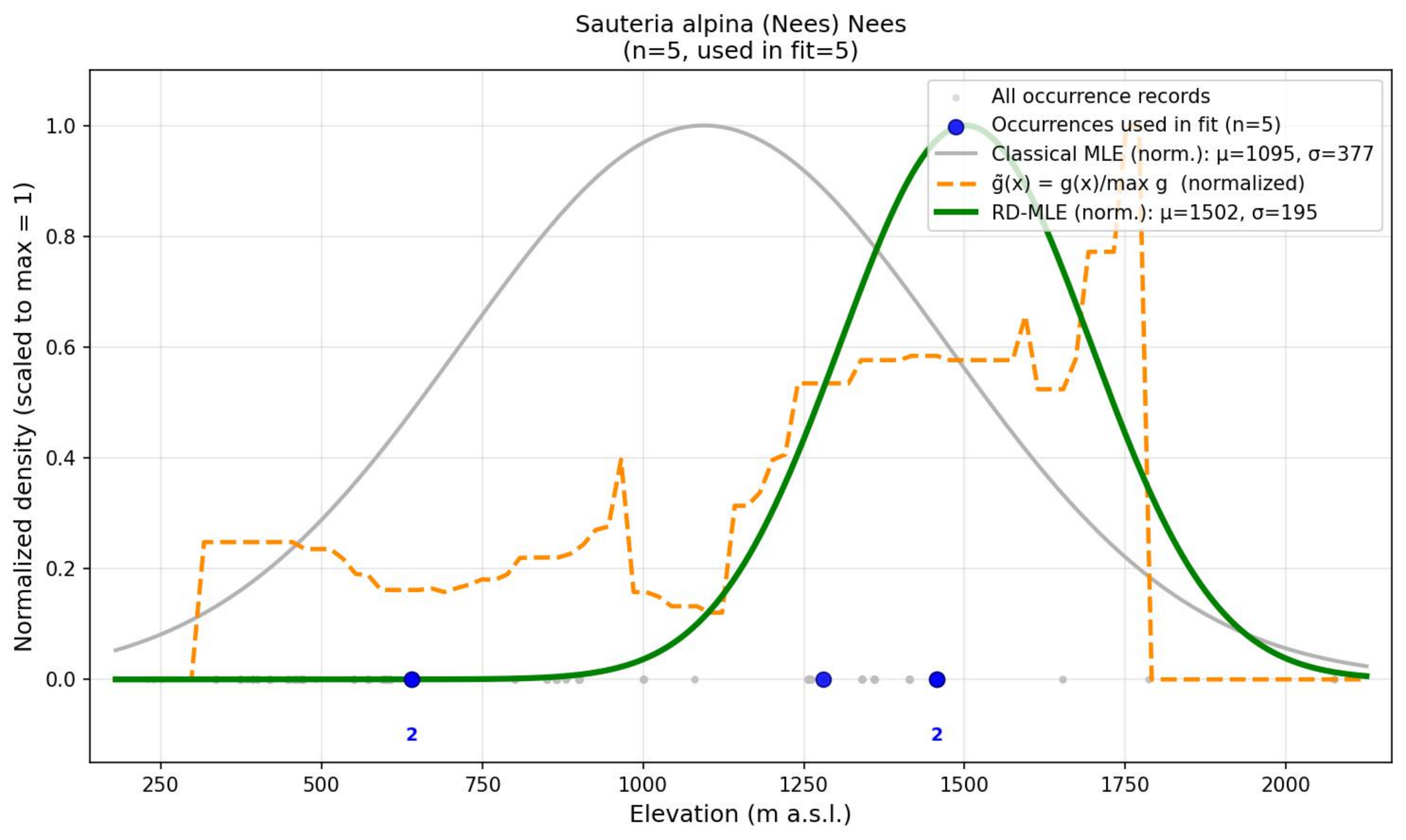
Sauteria alpina (Nees) Nees
(n=5, used in fit=5)
All occurrence records
Occurrences used in fit (n=5)
Classical MLE (norm.): μ=1095, σ=377
g̃(x) = g(x)/max g (normalized)
RD-MLE (norm.): μ=1502, σ=195
Normalized density (scaled to max = 1)
Elevation (m a.s.l.)
0.0
0.2
0.4
0.6
0.8
1.0
250
500
750
1000
1250
1500
1750
2000
2
2

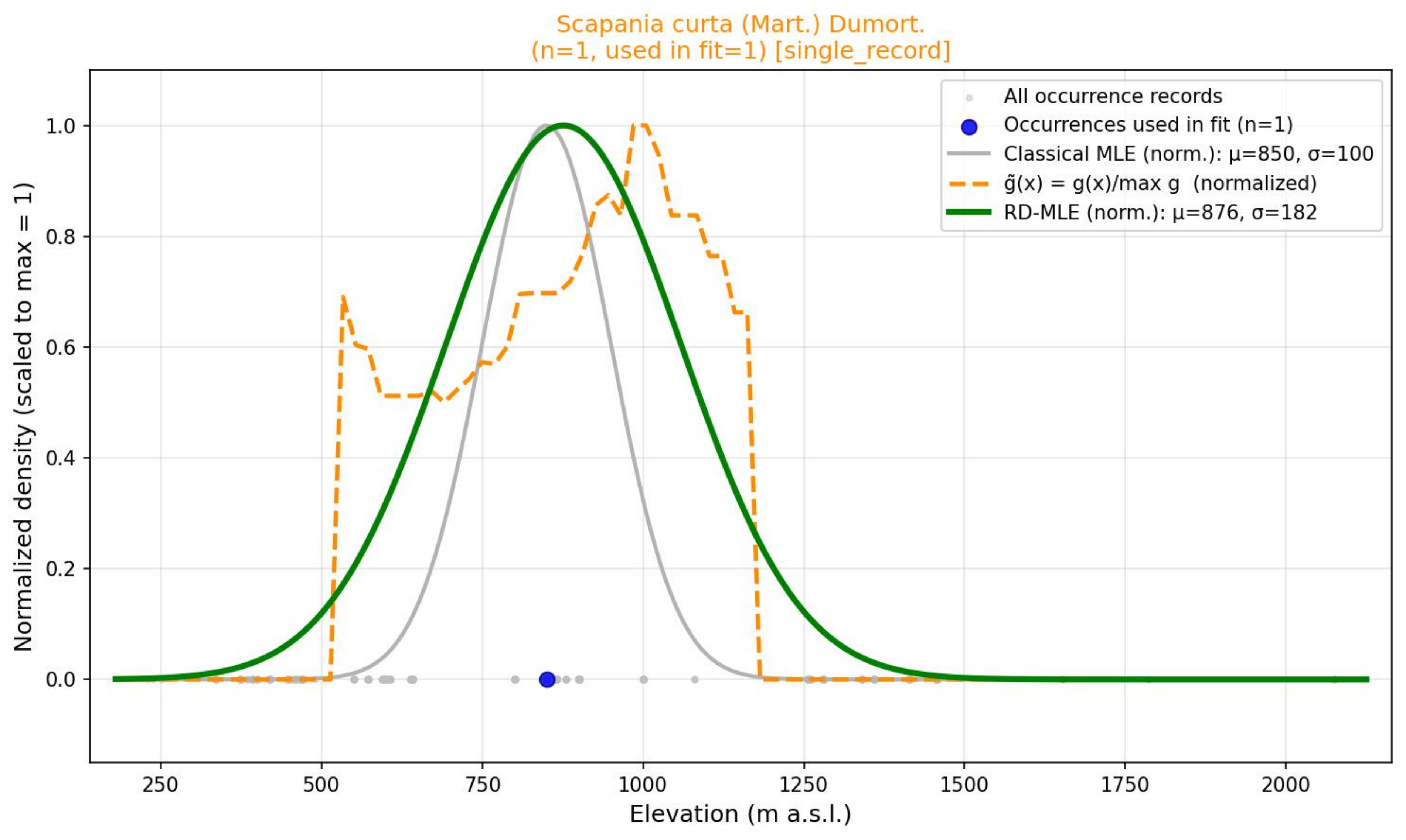
Scapania curta (Mart.) Dumort.
(n=1, used in fit=1) [single_record]
All occurrence records
Occurrences used in fit (n=1)
Classical MLE (norm.): μ=850, σ=100
g̃(x) = g(x)/max g (normalized)
RD-MLE (norm.): μ=876, σ=182
Normalized density (scaled to max = 1)
Elevation (m a.s.l.)
0.0
0.2
0.4
0.6
0.8
1.0
250
500
750
1000
1250
1500
1750
2000

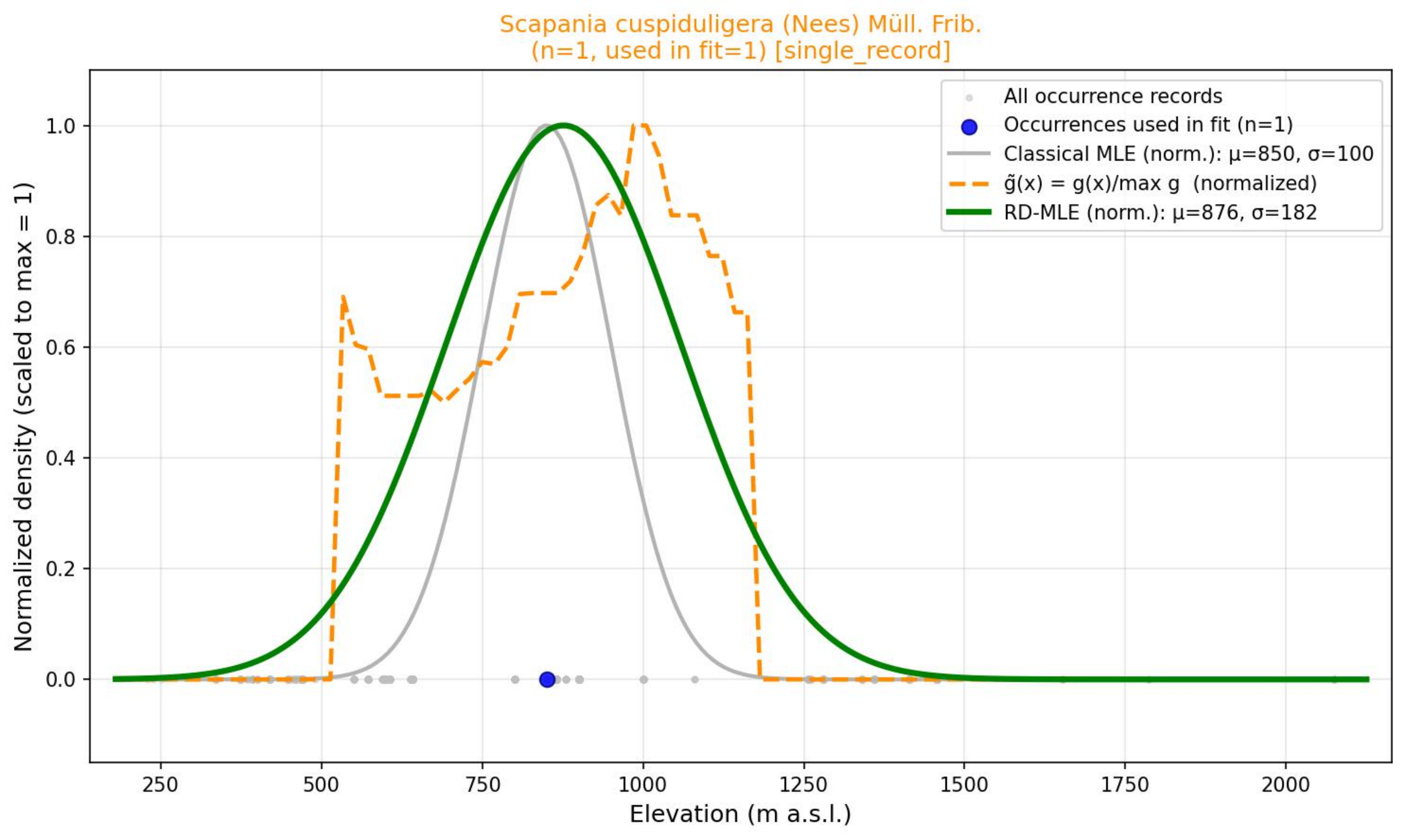
Scapania cuspiduligera (Nees) Müll. Frib.
(n=1, used in fit=1) [single_record]
All occurrence records
Occurrences used in fit (n=1)
Classical MLE (norm.): μ=850, σ=100
g̃(x) = g(x)/max g (normalized)
RD-MLE (norm.): μ=876, σ=182
Normalized density (scaled to max = 1)
Elevation (m a.s.l.)
0.0
0.2
0.4
0.6
0.8
1.0
250
500
750
1000
1250
1500
1750
2000

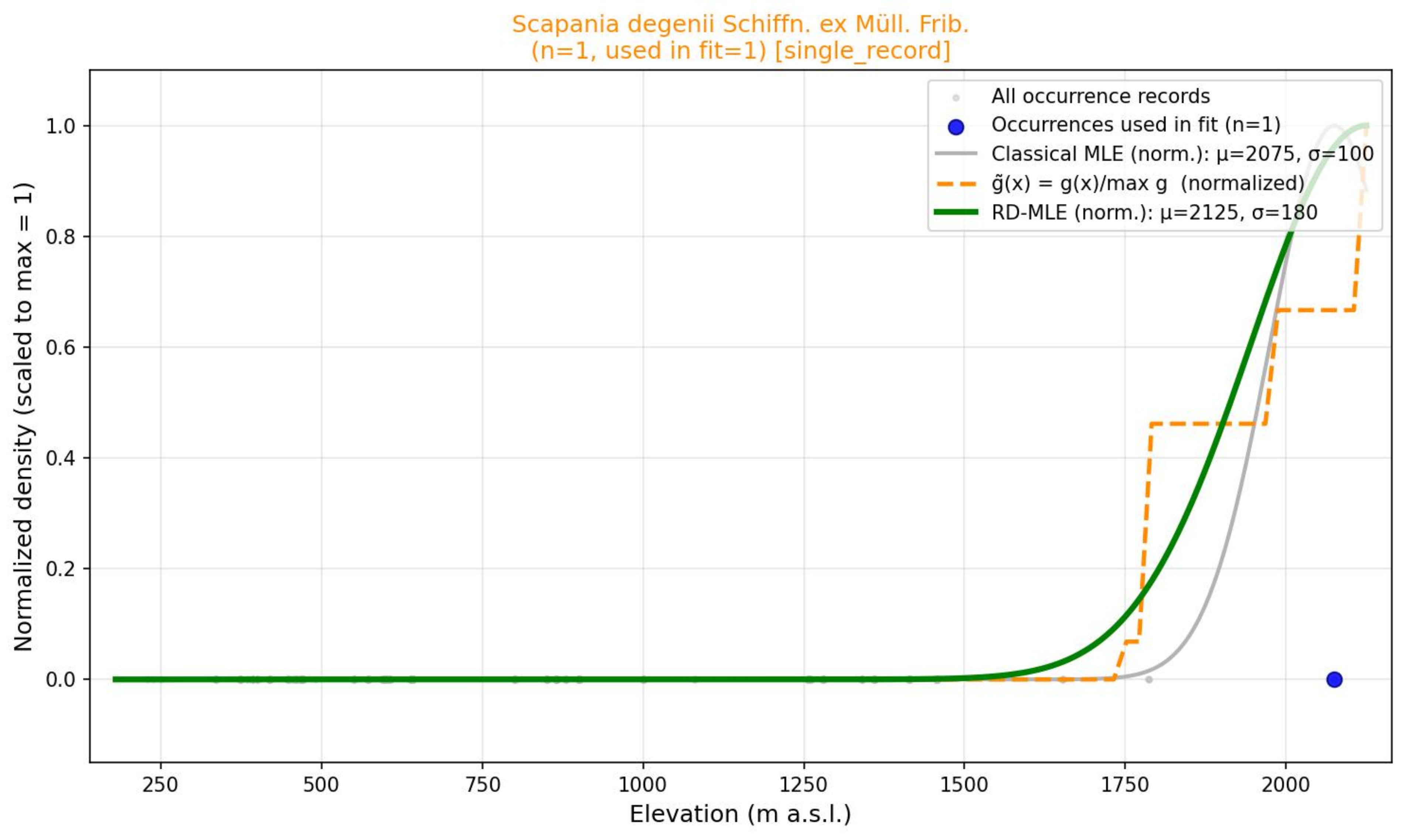
Scapania degenii Schiffn. ex Müll. Frib.
(n=1, used in fit=1) [single_record]
All occurrence records
Occurrences used in fit (n=1)
Classical MLE (norm.): μ=2075, σ=100
g̃(x) = g(x)/max g (normalized)
RD-MLE (norm.): μ=2125, σ=180
Normalized density (scaled to max = 1)
Elevation (m a.s.l.)
0.0
0.2
0.4
0.6
0.8
1.0
250
500
750
1000
1250
1500
1750
2000

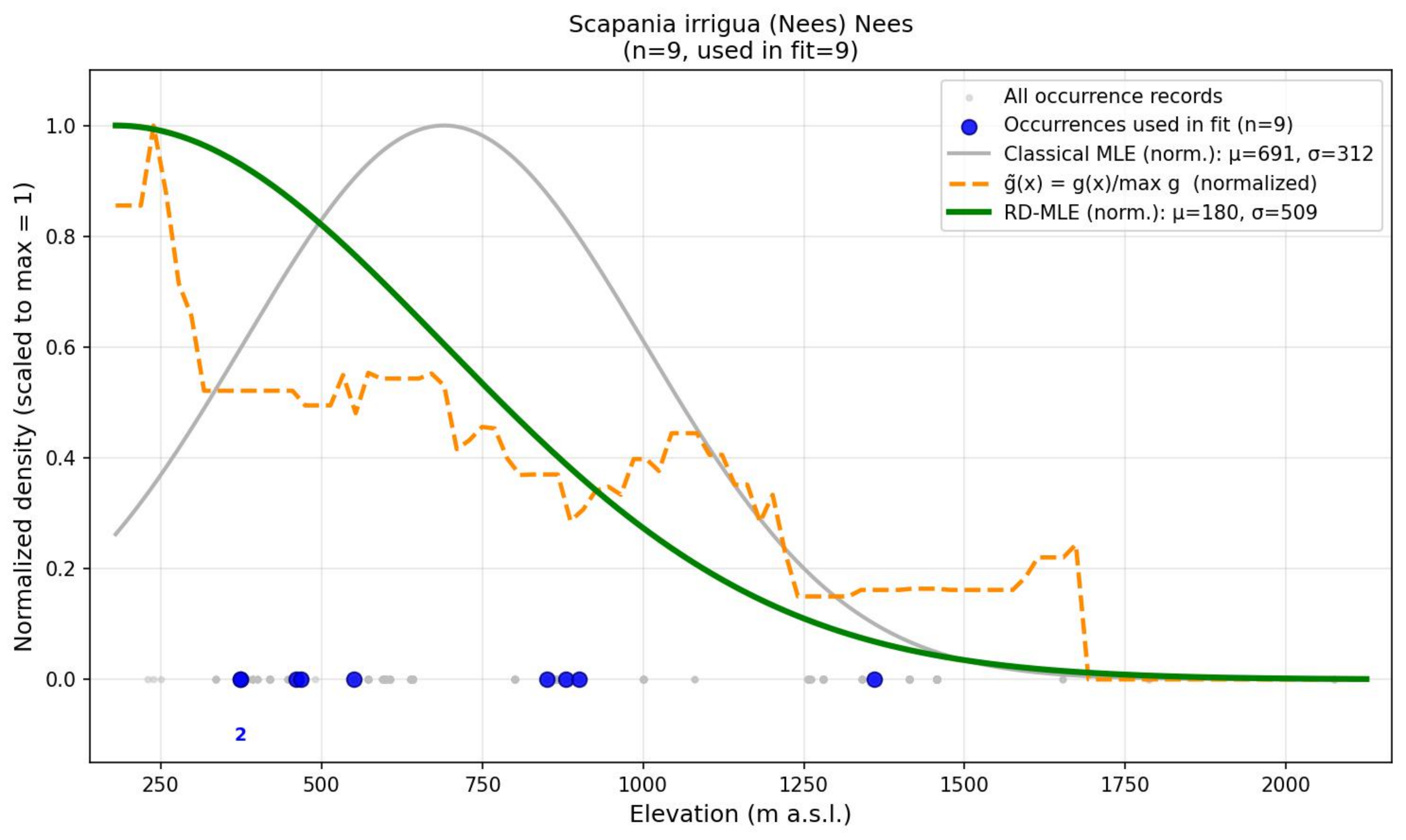

Scapania irrigua (Nees) Nees
(n=9, used in fit=9)
All occurrence records
Occurrences used in fit (n=9)
Classical MLE (norm.): μ=691, σ=312
g̃(x) = g(x)/max g (normalized)
RD-MLE (norm.): μ=180, σ=509
Normalized density (scaled to max = 1)
Elevation (m a.s.l.)
1.0
0.8
0.6
0.4
0.2
0.0
250
500
750
1000
1250
1500
1750
2000
2

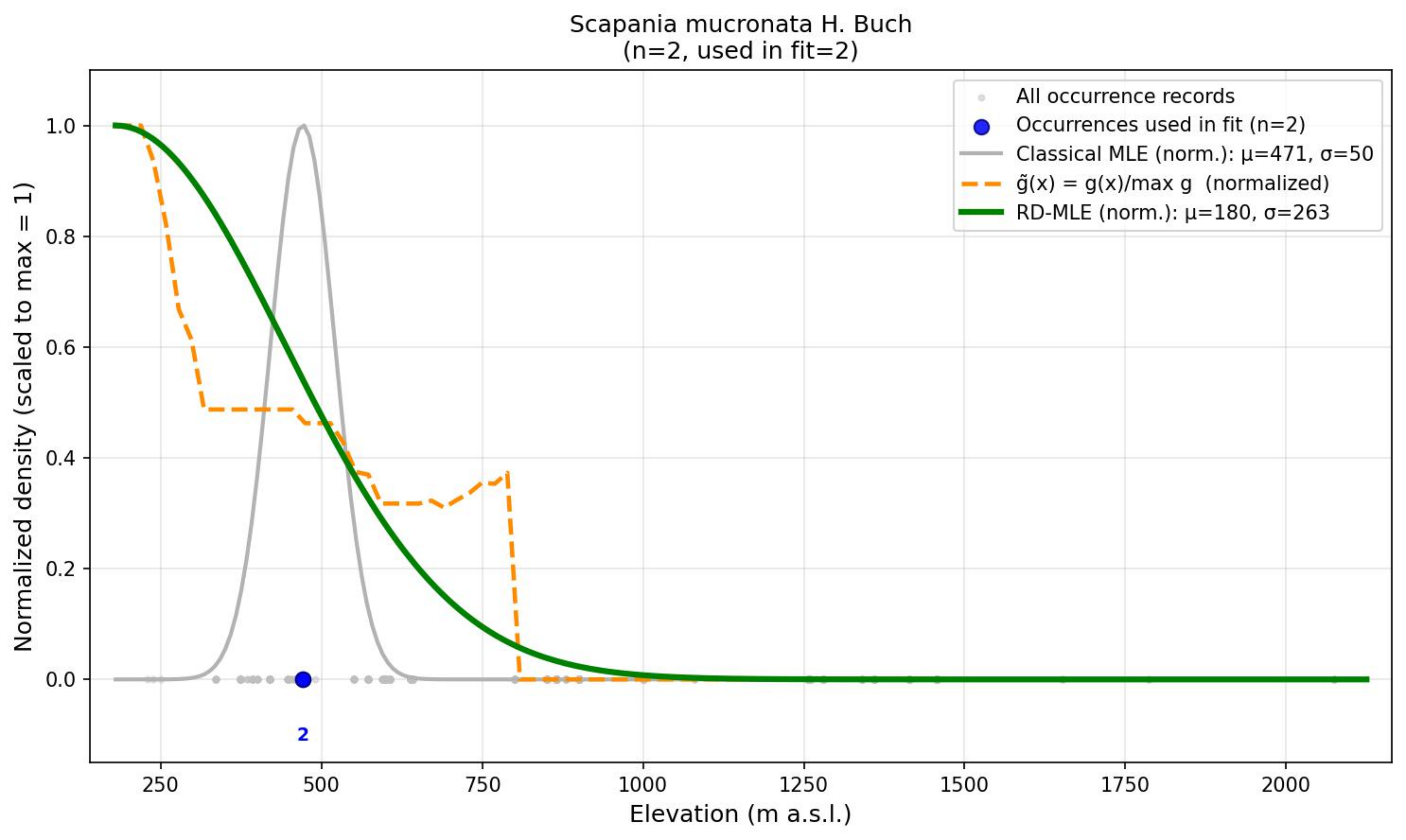

Scapania mucronata H. Buch
(n=2, used in fit=2)
All occurrence records
Occurrences used in fit (n=2)
Classical MLE (norm.): μ=471, σ=50
g̃(x) = g(x)/max g (normalized)
RD-MLE (norm.): μ=180, σ=263
Normalized density (scaled to max = 1)
1.0
0.8
0.6
0.4
0.2
0.0
2
250
500
750
1000
1250
1500
1750
2000
Elevation (m a.s.l.)

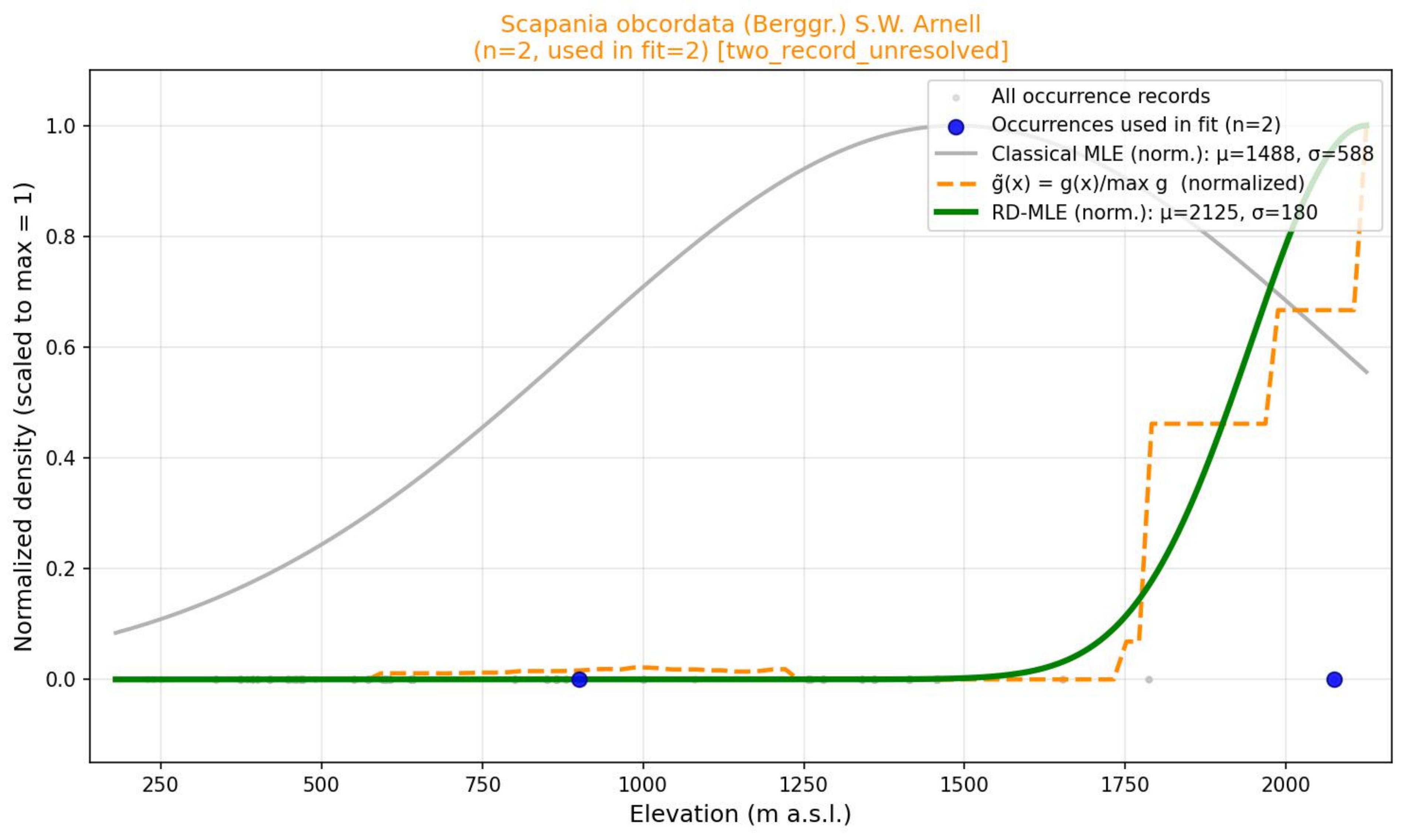

Scapania obcordata (Berggr.) S.W. Arnell
(n=2, used in fit=2) [two_record_unresolved]
All occurrence records
Occurrences used in fit (n=2)
Classical MLE (norm.): μ=1488, σ=588
g̃(x) = g(x)/max g (normalized)
RD-MLE (norm.): μ=2125, σ=180
Normalized density (scaled to max = 1)
Elevation (m a.s.l.)
0.0
0.2
0.4
0.6
0.8
1.0
250
500
750
1000
1250
1500
1750
2000

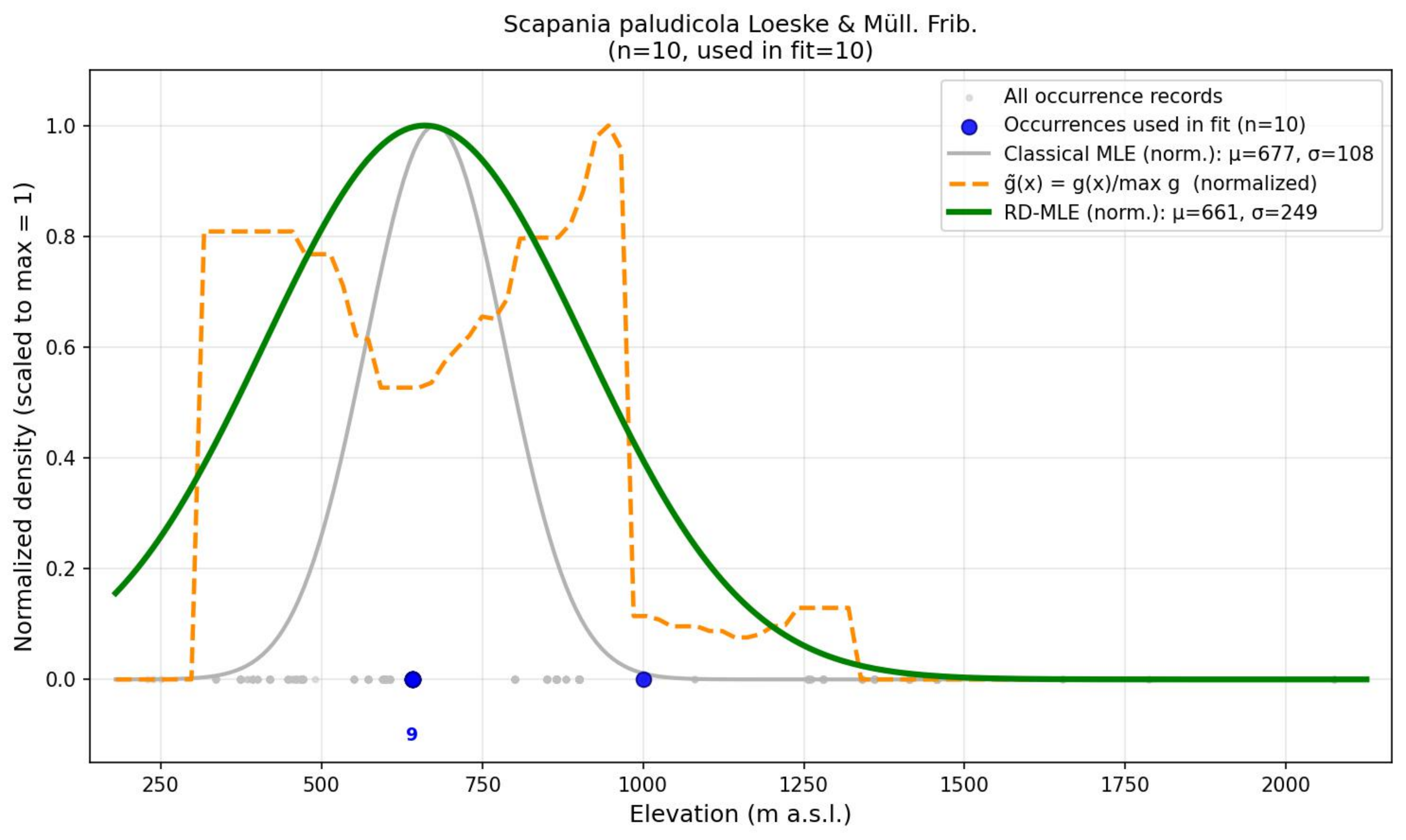

Scapania paludicola Loeske & Müll. Frib.
(n=10, used in fit=10)
All occurrence records
Occurrences used in fit (n=10)
Classical MLE (norm.): μ=677, σ=108
g̃(x) = g(x)/max g (normalized)
RD-MLE (norm.): μ=661, σ=249
Normalized density (scaled to max = 1)
Elevation (m a.s.l.)
1.0
0.8
0.6
0.4
0.2
0.0
250
500
750
1000
1250
1500
1750
2000
9

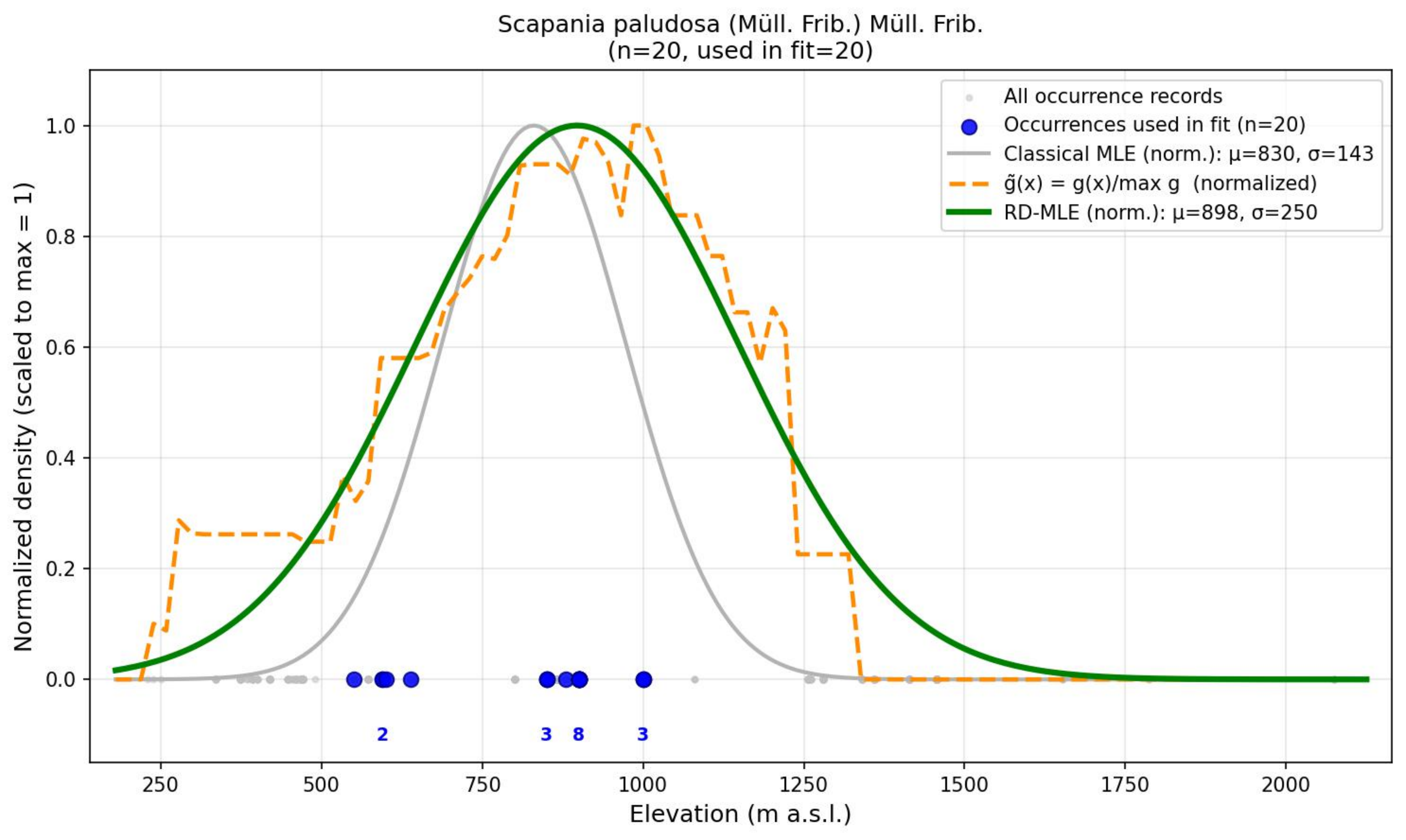

Scapania paludosa (Müll. Frib.) Müll. Frib.
(n=20, used in fit=20)
All occurrence records
Occurrences used in fit (n=20)
Classical MLE (norm.): μ=830, σ=143
g̃(x) = g(x)/max g (normalized)
RD-MLE (norm.): μ=898, σ=250
Normalized density (scaled to max = 1)
Elevation (m a.s.l.)
0.0
0.2
0.4
0.6
0.8
1.0
250
500
750
1000
1250
1500
1750
2000
2
3
8
3

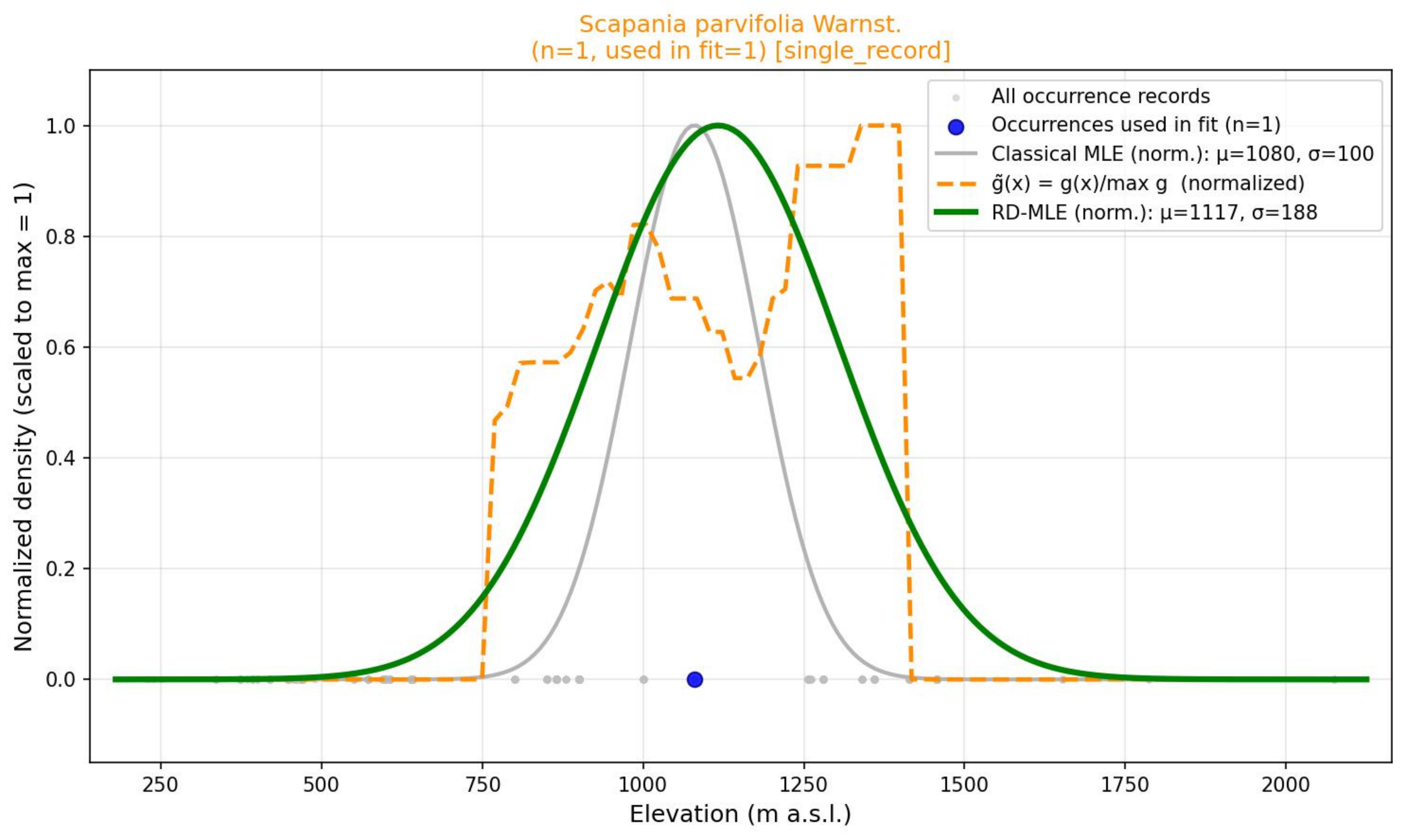
Scapania parvifolia Warnst.
(n=1, used in fit=1) [single_record]
All occurrence records
Occurrences used in fit (n=1)
Classical MLE (norm.): μ=1080, σ=100
g̃(x) = g(x)/max g (normalized)
RD-MLE (norm.): μ=1117, σ=188
Normalized density (scaled to max = 1)
Elevation (m a.s.l.)
0.0
0.2
0.4
0.6
0.8
1.0
250
500
750
1000
1250
1500
1750
2000

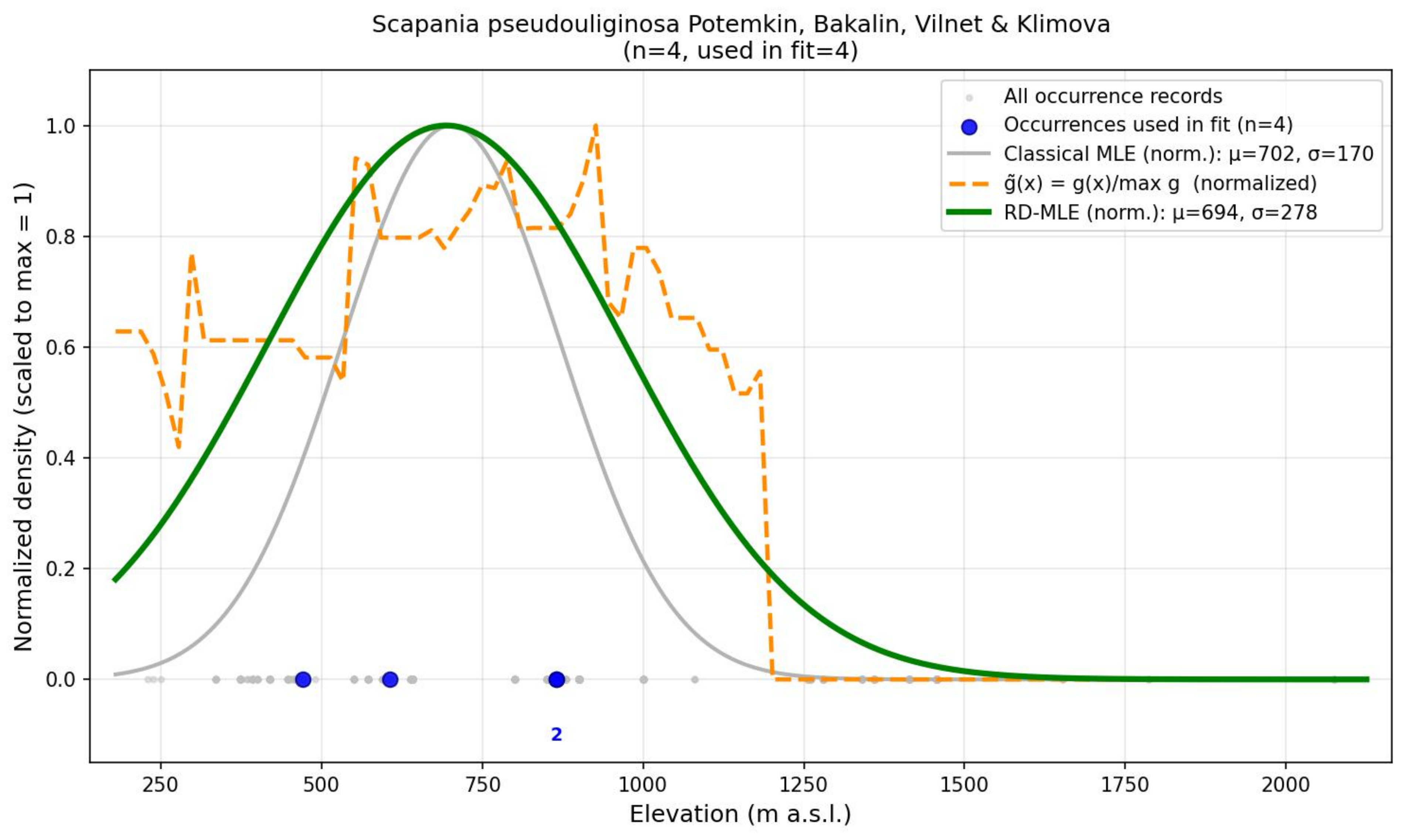
Scapania pseudouliginosa Potemkin, Bakalin, Vilnet & Klimova
(n=4, used in fit=4)
All occurrence records
Occurrences used in fit (n=4)
Classical MLE (norm.): μ=702, σ=170
g̃(x) = g(x)/max g (normalized)
RD-MLE (norm.): μ=694, σ=278
Normalized density (scaled to max = 1)
Elevation (m a.s.l.)
1.0
0.8
0.6
0.4
0.2
0.0
250
500
750
1000
1250
1500
1750
2000
2

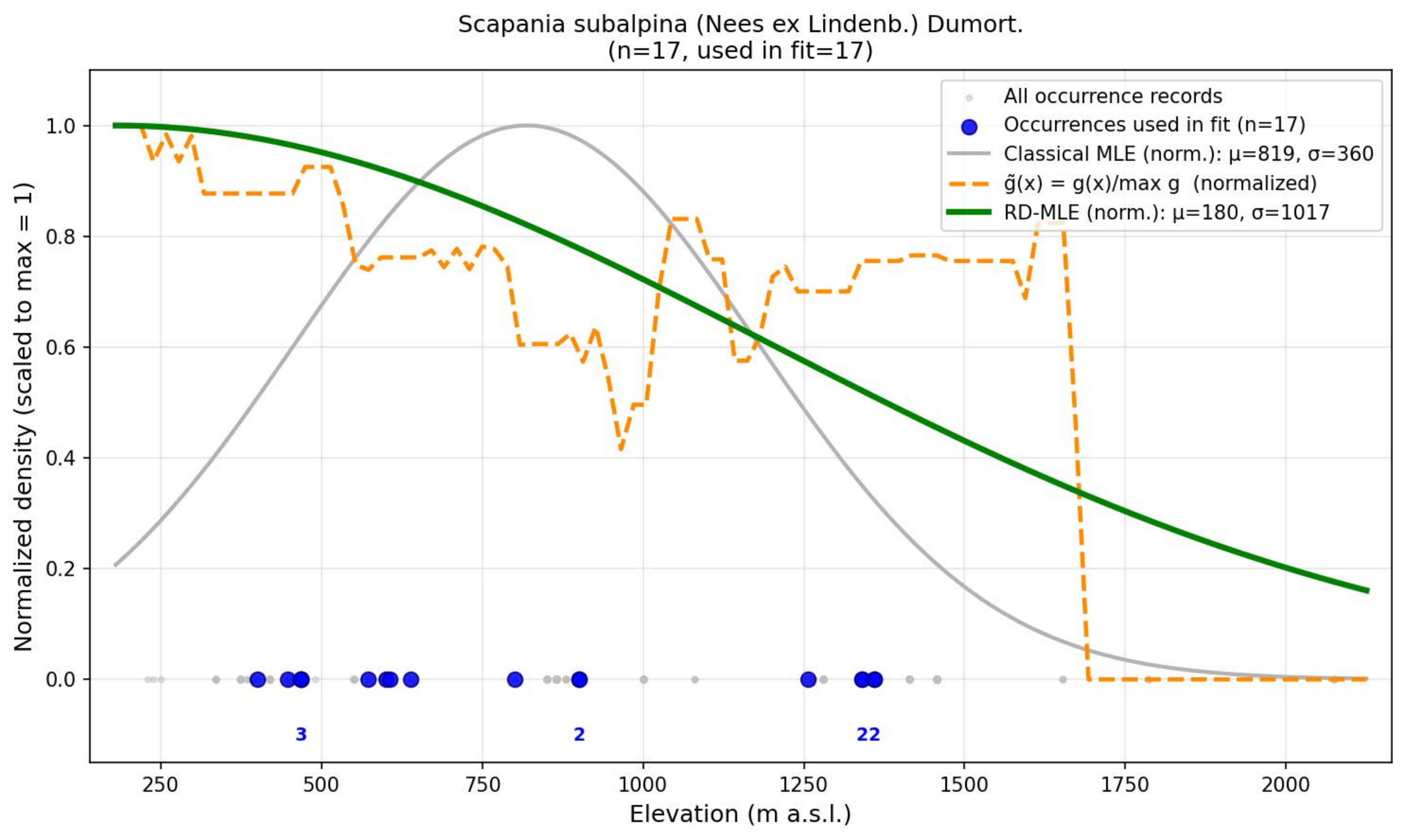

Scapania subalpina (Nees ex Lindenb.) Dumort.
(n=17, used in fit=17)
All occurrence records
Occurrences used in fit (n=17)
Classical MLE (norm.): μ=819, σ=360
g̃(x) = g(x)/max g (normalized)
RD-MLE (norm.): μ=180, σ=1017
Normalized density (scaled to max = 1)
Elevation (m a.s.l.)
1.0
0.8
0.6
0.4
0.2
0.0
250
500
750
1000
1250
1500
1750
2000
3
2
22

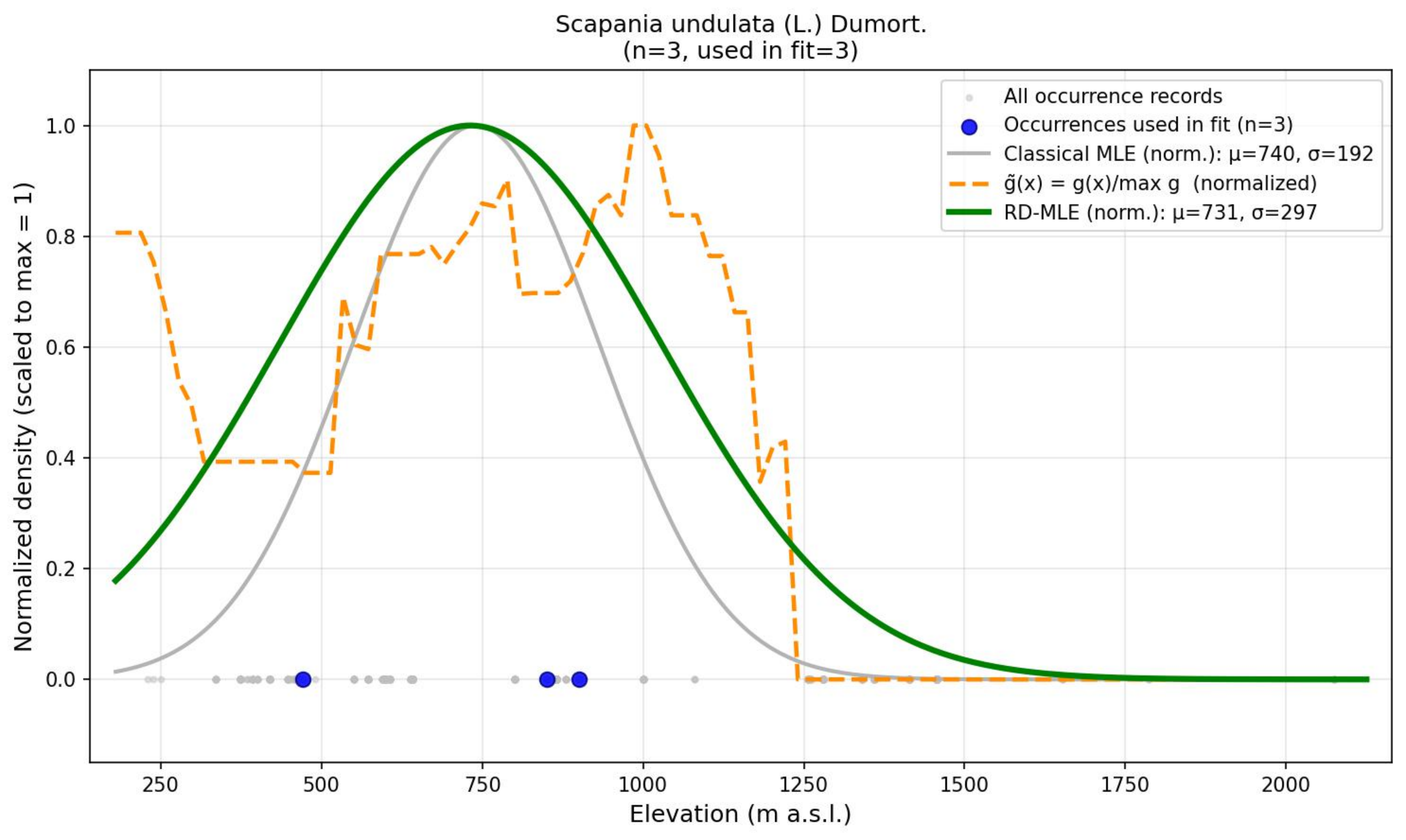

Scapania undulata (L.) Dumort.
(n=3, used in fit=3)
All occurrence records
Occurrences used in fit (n=3)
Classical MLE (norm.): μ=740, σ=192
g̃(x) = g(x)/max g (normalized)
RD-MLE (norm.): μ=731, σ=297
Normalized density (scaled to max = 1)
Elevation (m a.s.l.)
1.0
0.8
0.6
0.4
0.2
0.0
250
500
750
1000
1250
1500
1750
2000

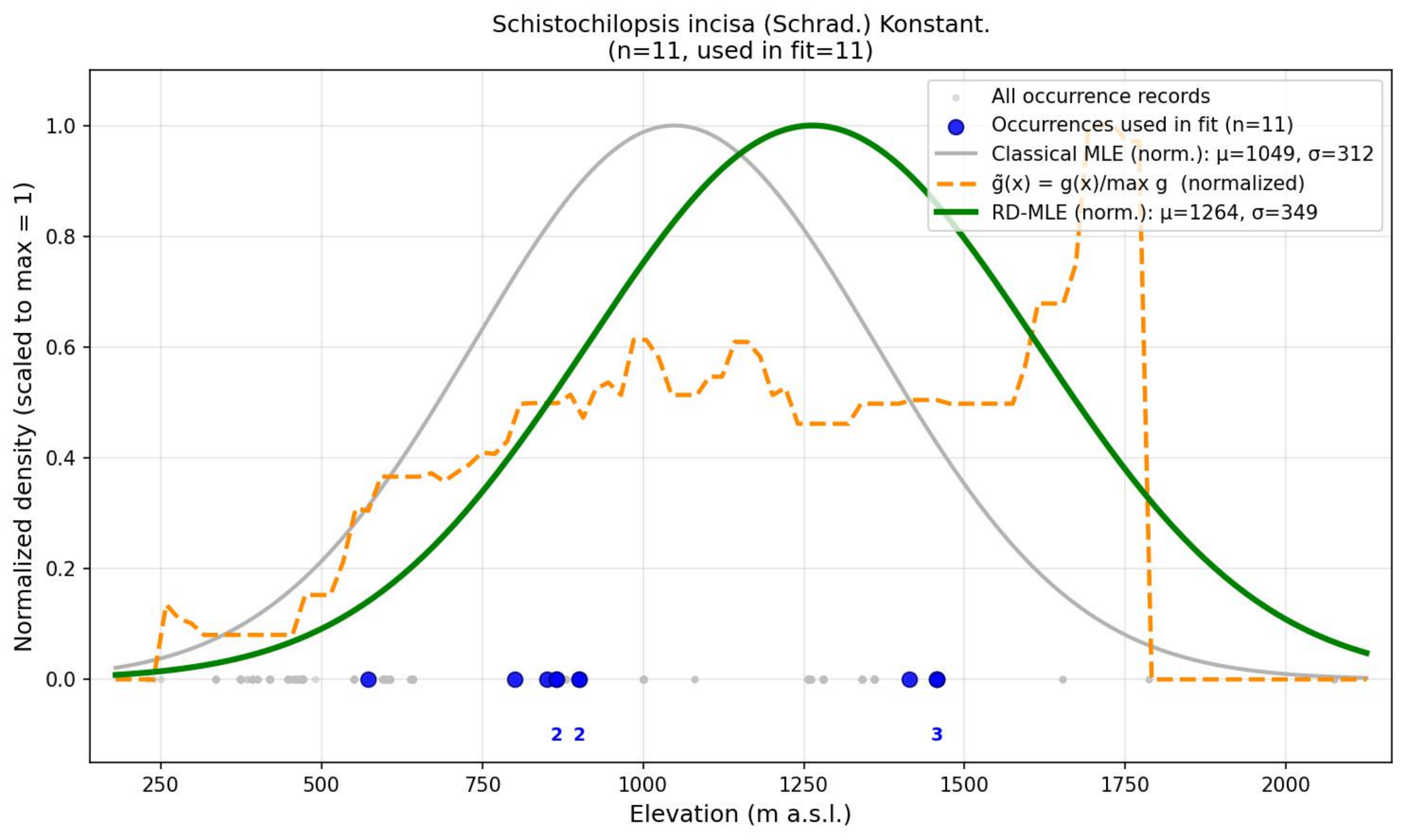

Schistochilopsis incisa (Schrad.) Konstant.
(n=11, used in fit=11)
All occurrence records
Occurrences used in fit (n=11)
Classical MLE (norm.): μ=1049, σ=312
g̃(x) = g(x)/max g (normalized)
RD-MLE (norm.): μ=1264, σ=349
Normalized density (scaled to max = 1)
1.0
0.8
0.6
0.4
0.2
0.0
2
2
3
250
500
750
1000
1250
1500
1750
2000
Elevation (m a.s.l.)

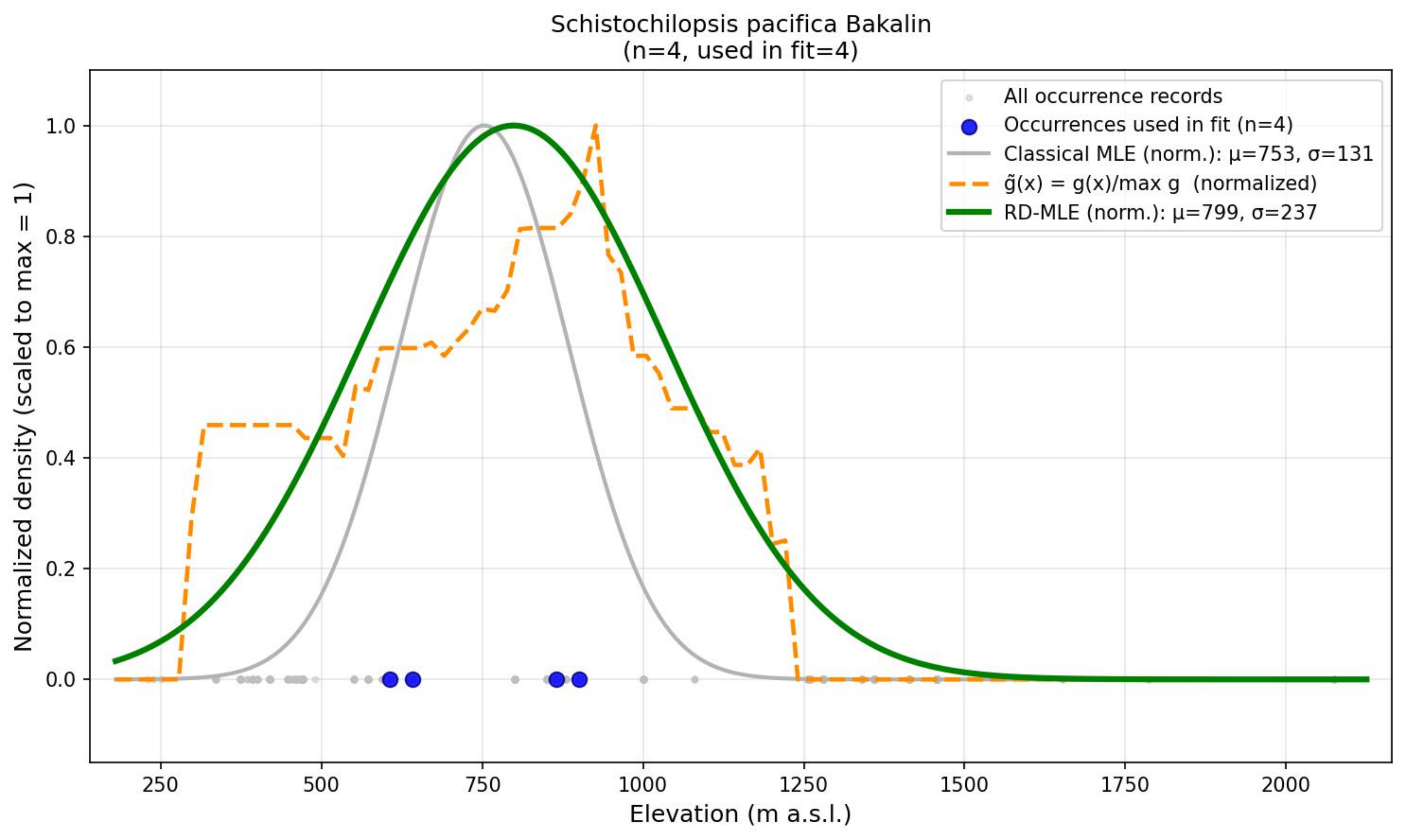

Schistochilopsis pacifica Bakalin
(n=4, used in fit=4)
All occurrence records
Occurrences used in fit (n=4)
Classical MLE (norm.): μ=753, σ=131
g̃(x) = g(x)/max g (normalized)
RD-MLE (norm.): μ=799, σ=237
Normalized density (scaled to max = 1)
Elevation (m a.s.l.)
0.0
0.2
0.4
0.6
0.8
1.0
250
500
750
1000
1250
1500
1750
2000

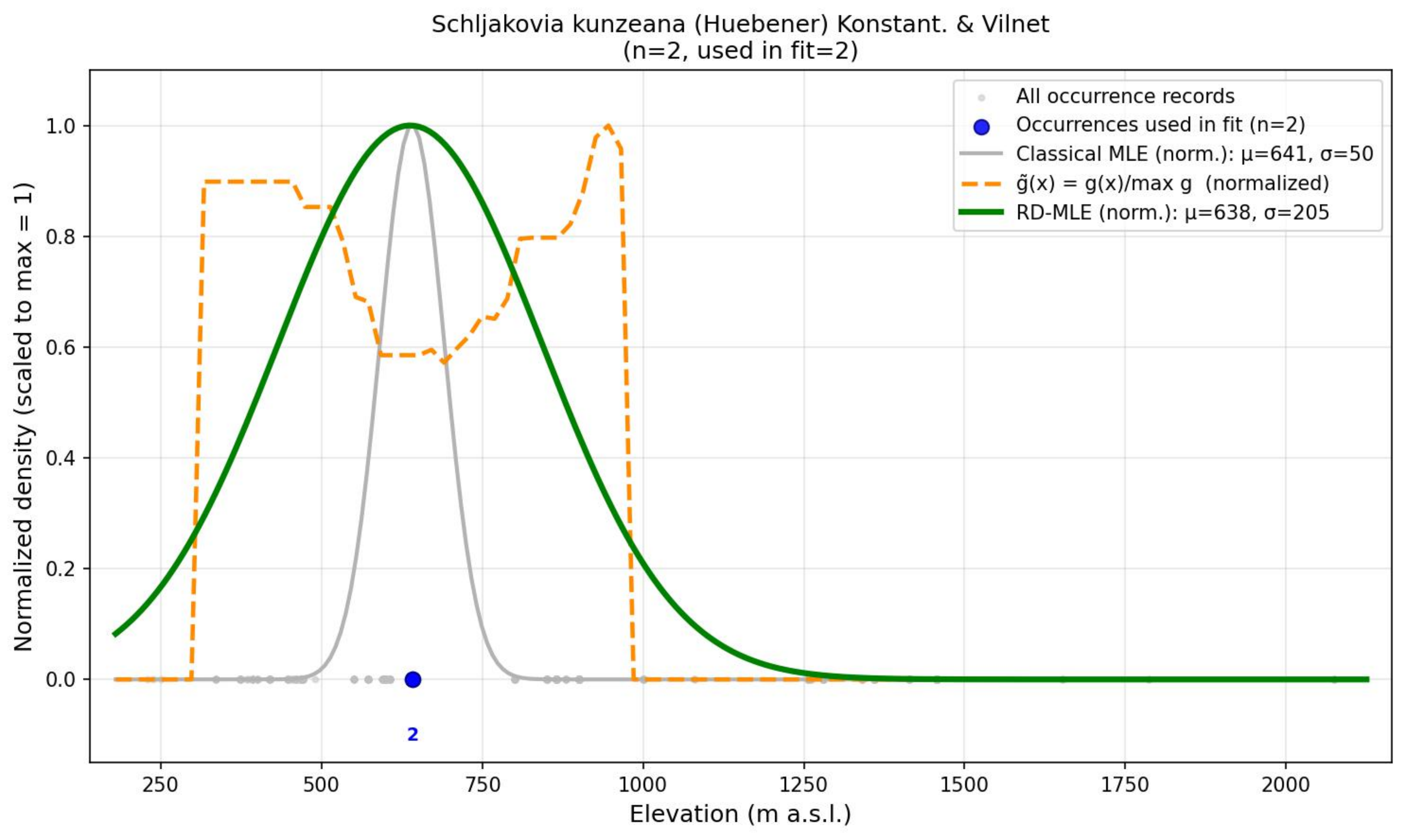
Schljakovia kunzeana (Huebener) Konstant. & Vilnet
(n=2, used in fit=2)
All occurrence records
Occurrences used in fit (n=2)
Classical MLE (norm.): μ=641, σ=50
g̃(x) = g(x)/max g (normalized)
RD-MLE (norm.): μ=638, σ=205
Normalized density (scaled to max = 1)
Elevation (m a.s.l.)
2
0.0
0.2
0.4
0.6
0.8
1.0
250
500
750
1000
1250
1500
1750
2000

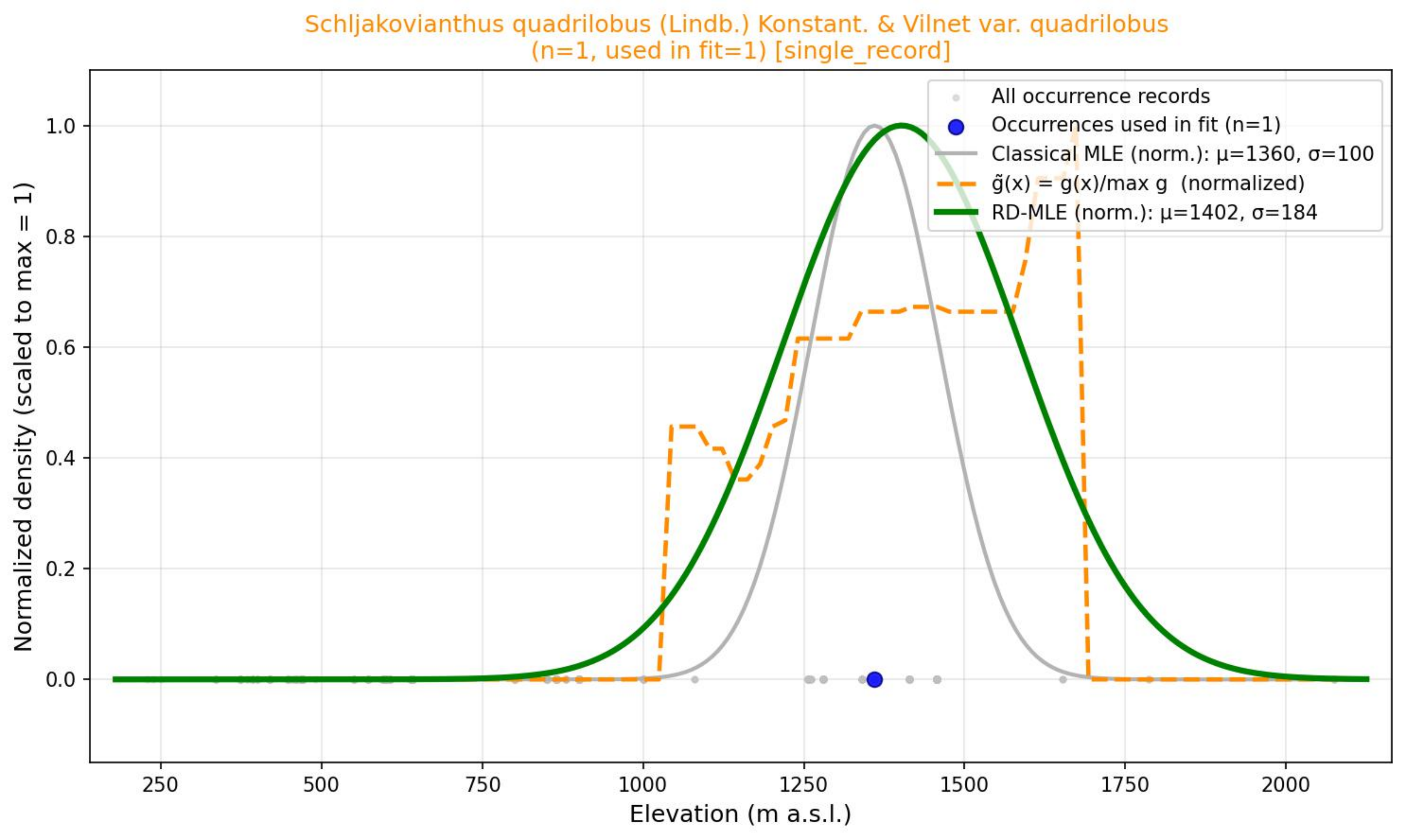
Schljakovianthus quadrilobus (Lindb.) Konstant. & Vilnet var. quadrilobus
(n=1, used in fit=1) [single_record]
All occurrence records
Occurrences used in fit (n=1)
Classical MLE (norm.): μ=1360, σ=100
g̃(x) = g(x)/max g (normalized)
RD-MLE (norm.): μ=1402, σ=184
Normalized density (scaled to max = 1)
Elevation (m a.s.l.)
0.0
0.2
0.4
0.6
0.8
1.0
250
500
750
1000
1250
1500
1750
2000

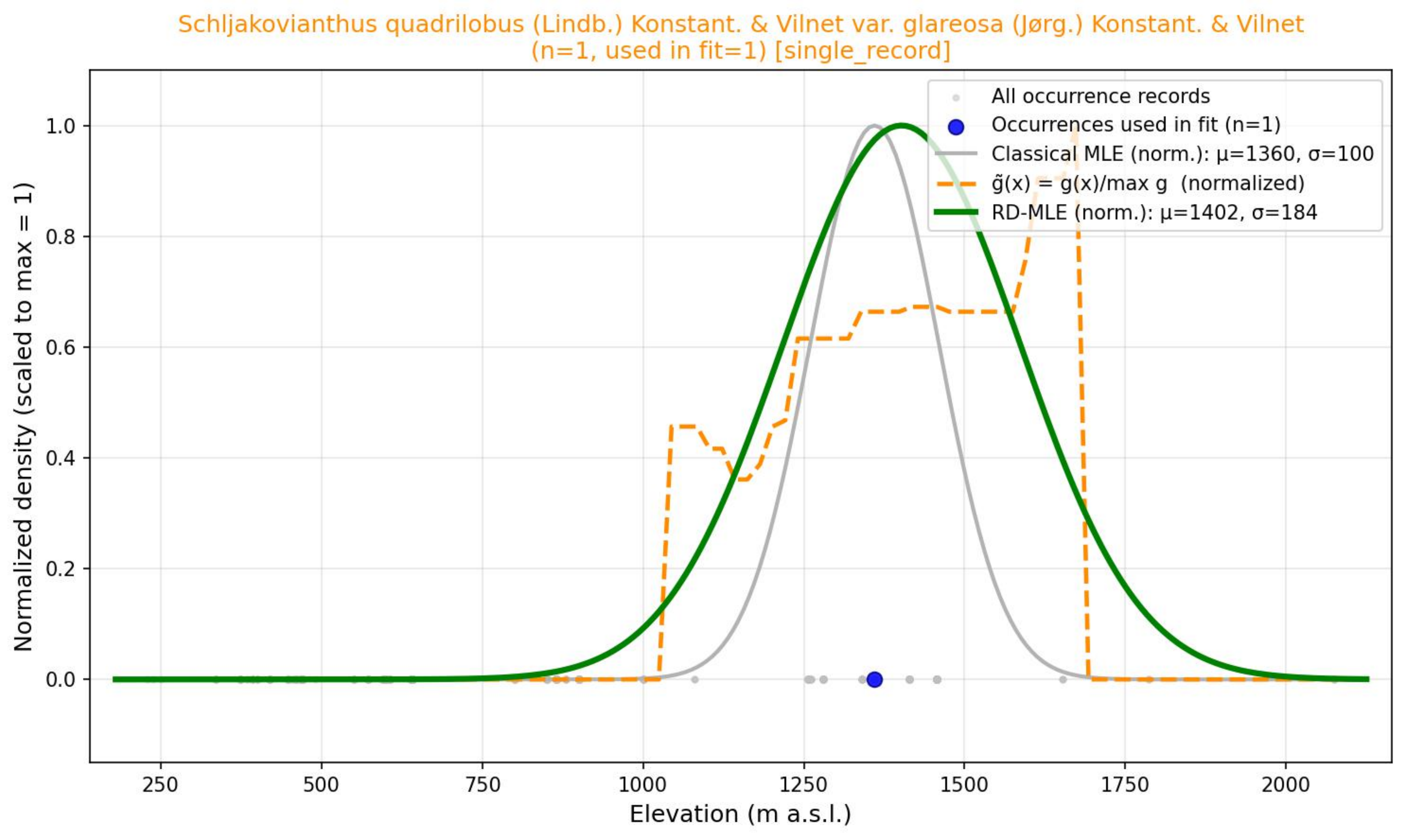

Schljakovianthus quadrilobus (Lindb.) Konstant. & Vilnet var. glareosa (Jørg.) Konstant. & Vilnet
(n=1, used in fit=1) [single_record]
All occurrence records
Occurrences used in fit (n=1)
Classical MLE (norm.): μ=1360, σ=100
g̃(x) = g(x)/max g (normalized)
RD-MLE (norm.): μ=1402, σ=184
Normalized density (scaled to max = 1)
Elevation (m a.s.l.)
0.0
0.2
0.4
0.6
0.8
1.0
250
500
750
1000
1250
1500
1750
2000

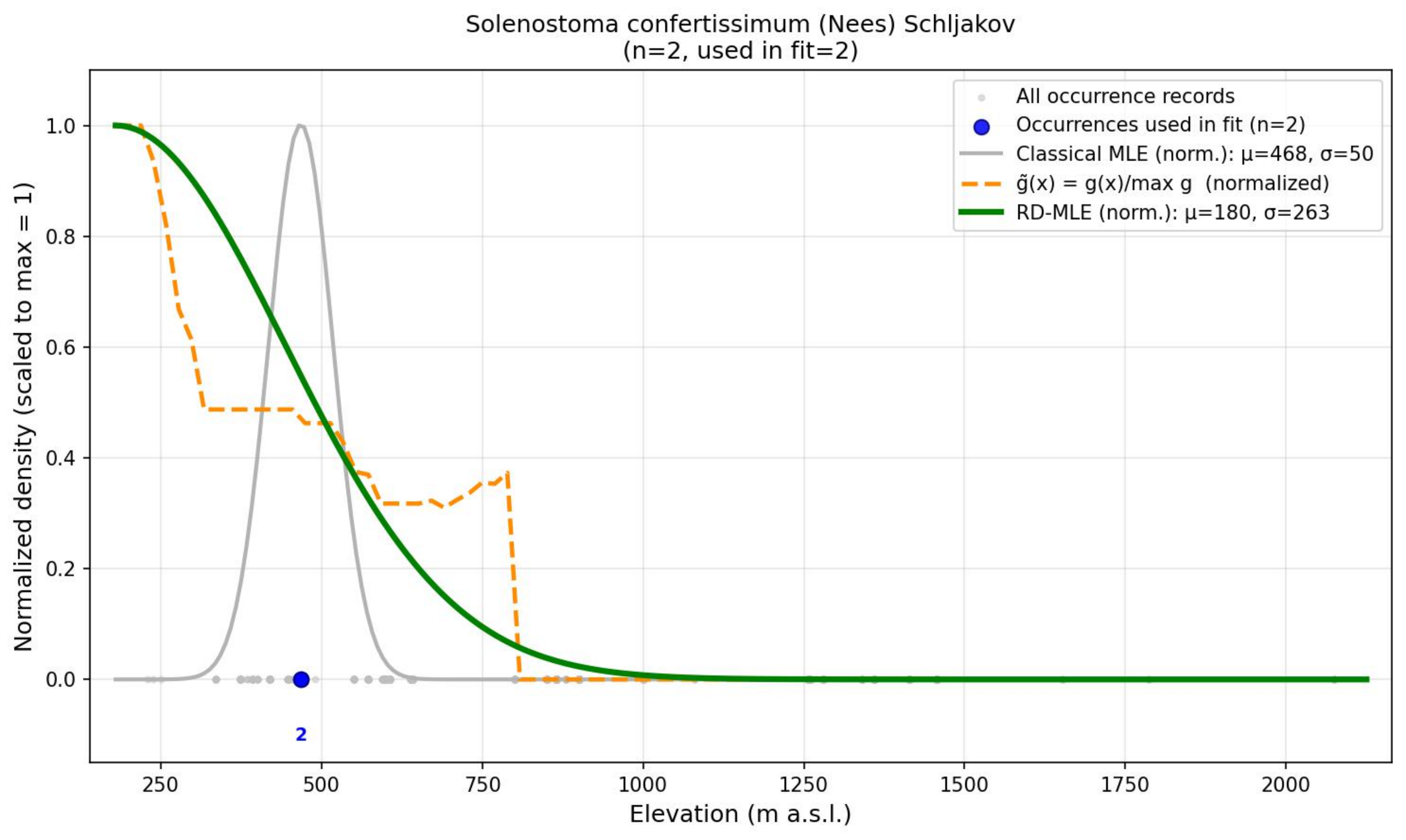

Solenostoma confertissimum (Nees) Schljakov
(n=2, used in fit=2)
All occurrence records
Occurrences used in fit (n=2)
Classical MLE (norm.): μ=468, σ=50
g̃(x) = g(x)/max g (normalized)
RD-MLE (norm.): μ=180, σ=263
Normalized density (scaled to max = 1)
1.0
0.8
0.6
0.4
0.2
0.0
2
250
500
750
1000
1250
1500
1750
2000
Elevation (m a.s.l.)

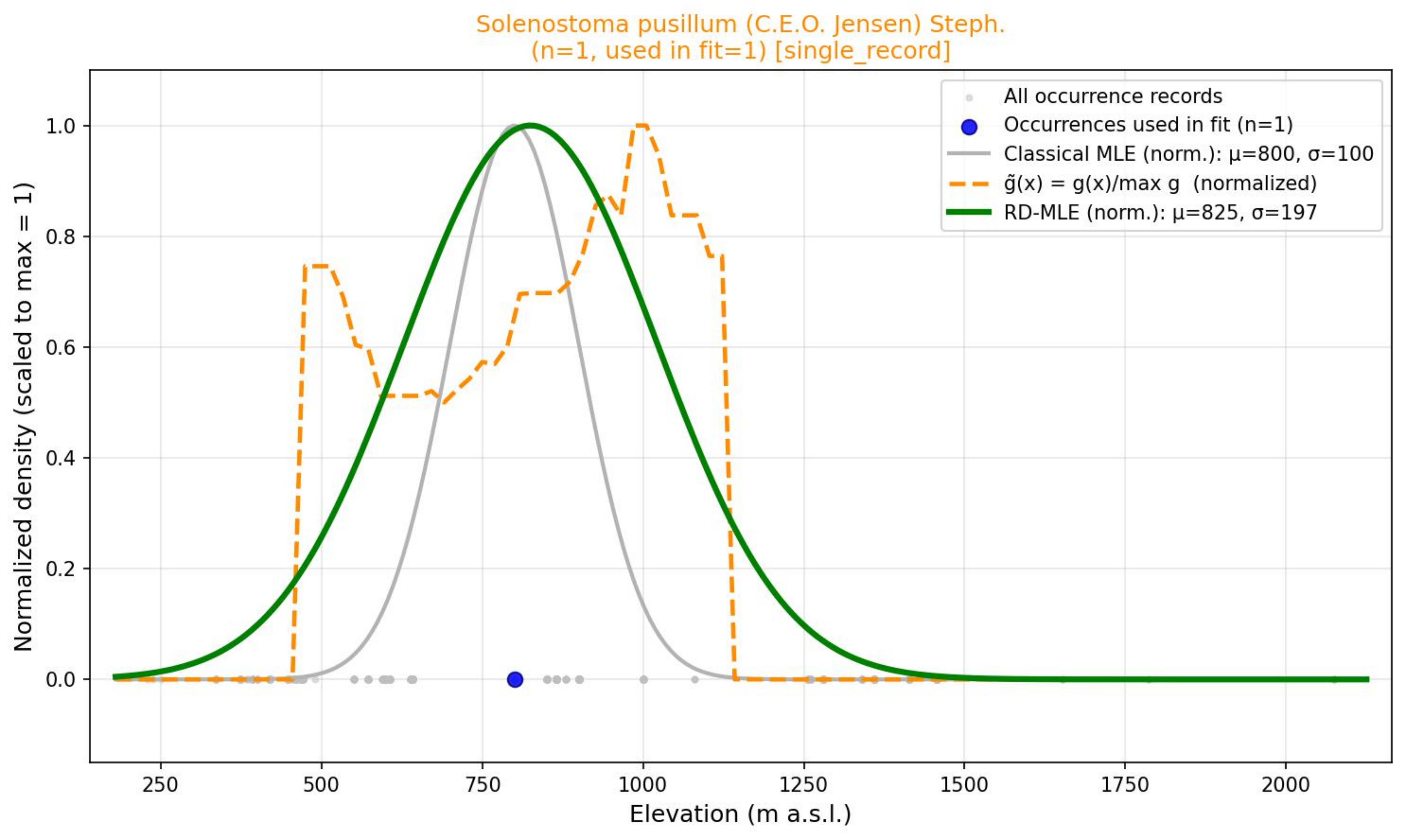
Solenostoma pusillum (C.E.O. Jensen) Steph.
(n=1, used in fit=1) [single_record]
All occurrence records
Occurrences used in fit (n=1)
Classical MLE (norm.): μ=800, σ=100
g̃(x) = g(x)/max g (normalized)
RD-MLE (norm.): μ=825, σ=197
Normalized density (scaled to max = 1)
Elevation (m a.s.l.)
1.0
0.8
0.6
0.4
0.2
0.0
250
500
750
1000
1250
1500
1750
2000

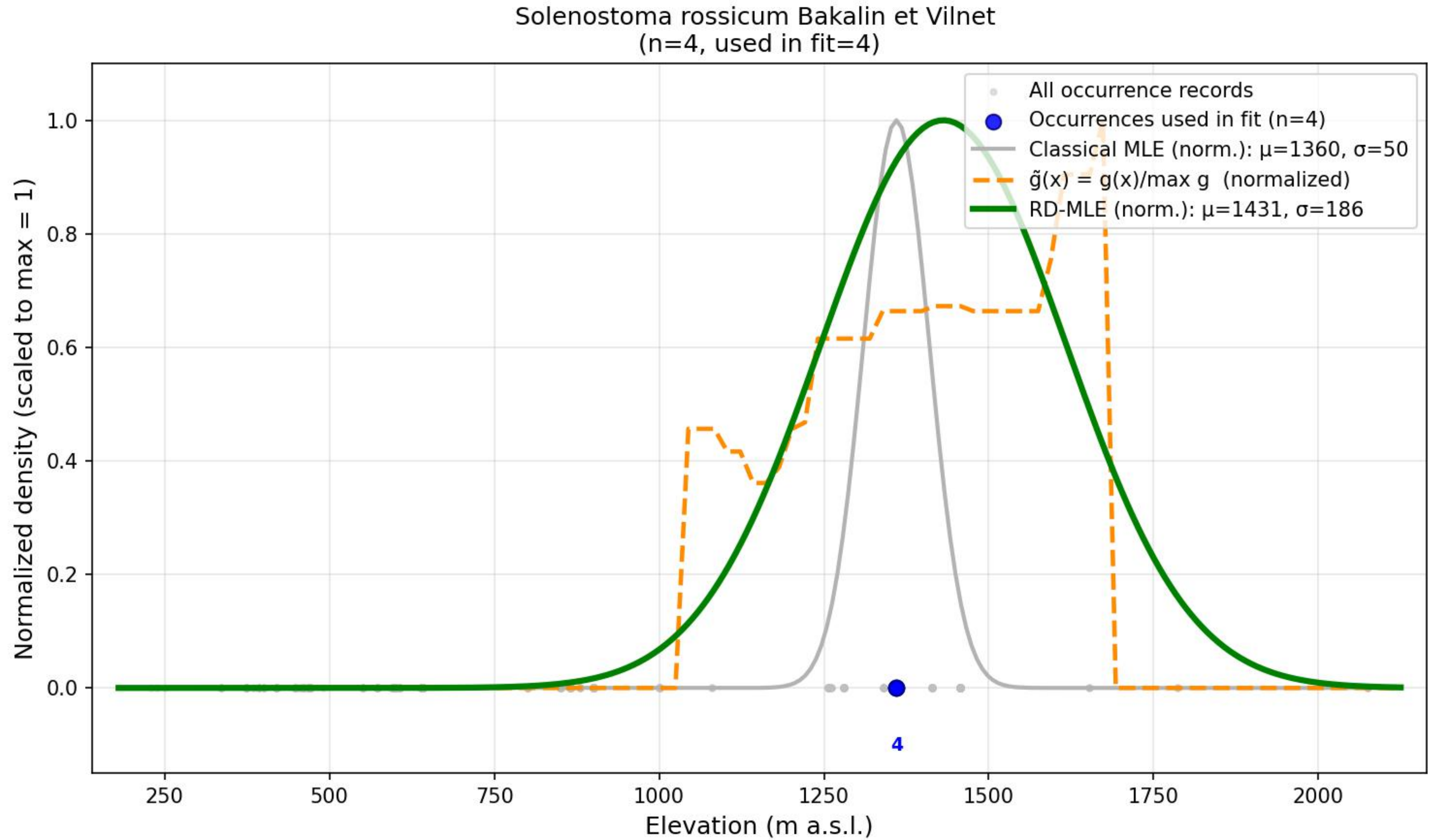
Solenostoma rossicum Bakalin et Vilnet
(n=4, used in fit=4)
All occurrence records
Occurrences used in fit (n=4)
Classical MLE (norm.): μ=1360, σ=50
g̃(x) = g(x)/max g (normalized)
RD-MLE (norm.): μ=1431, σ=186
Normalized density (scaled to max = 1)
Elevation (m a.s.l.)
4
1.0
0.8
0.6
0.4
0.2
0.0
250
500
750
1000
1250
1500
1750
2000

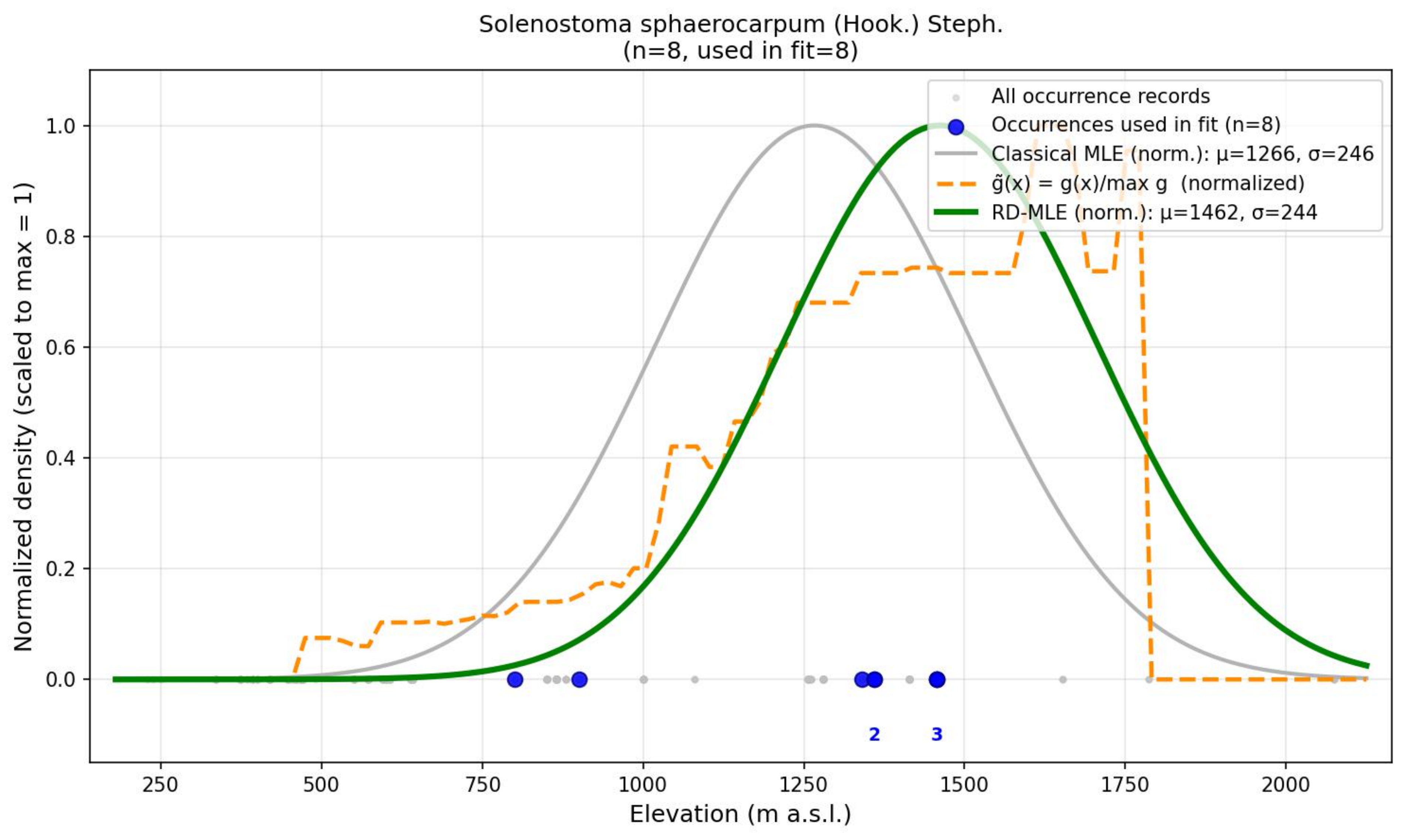

Solenostoma sphaerocarpum (Hook.) Steph.
(n=8, used in fit=8)
All occurrence records
Occurrences used in fit (n=8)
Classical MLE (norm.): μ=1266, σ=246
g̃(x) = g(x)/max g (normalized)
RD-MLE (norm.): μ=1462, σ=244
Normalized density (scaled to max = 1)
Elevation (m a.s.l.)
0.0
0.2
0.4
0.6
0.8
1.0
250
500
750
1000
1250
1500
1750
2000
2
3

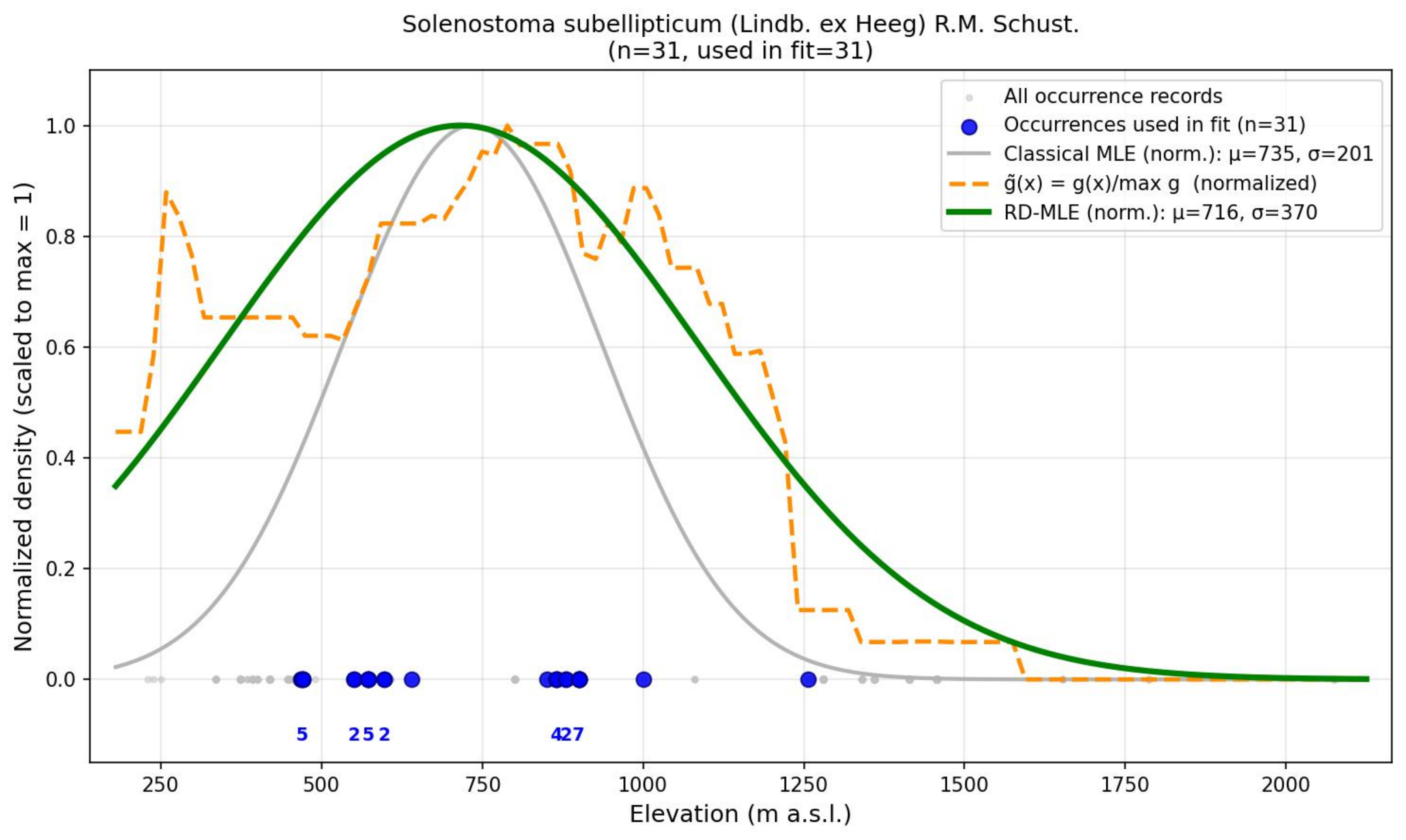

Solenostoma subellipticum (Lindb. ex Heeg) R.M. Schust.
(n=31, used in fit=31)
All occurrence records
Occurrences used in fit (n=31)
Classical MLE (norm.): μ=735, σ=201
g̃(x) = g(x)/max g (normalized)
RD-MLE (norm.): μ=716, σ=370
Normalized density (scaled to max = 1)
Elevation (m a.s.l.)
0.0
0.2
0.4
0.6
0.8
1.0
250
500
750
1000
1250
1500
1750
2000
5
2
5
2
4
2
7

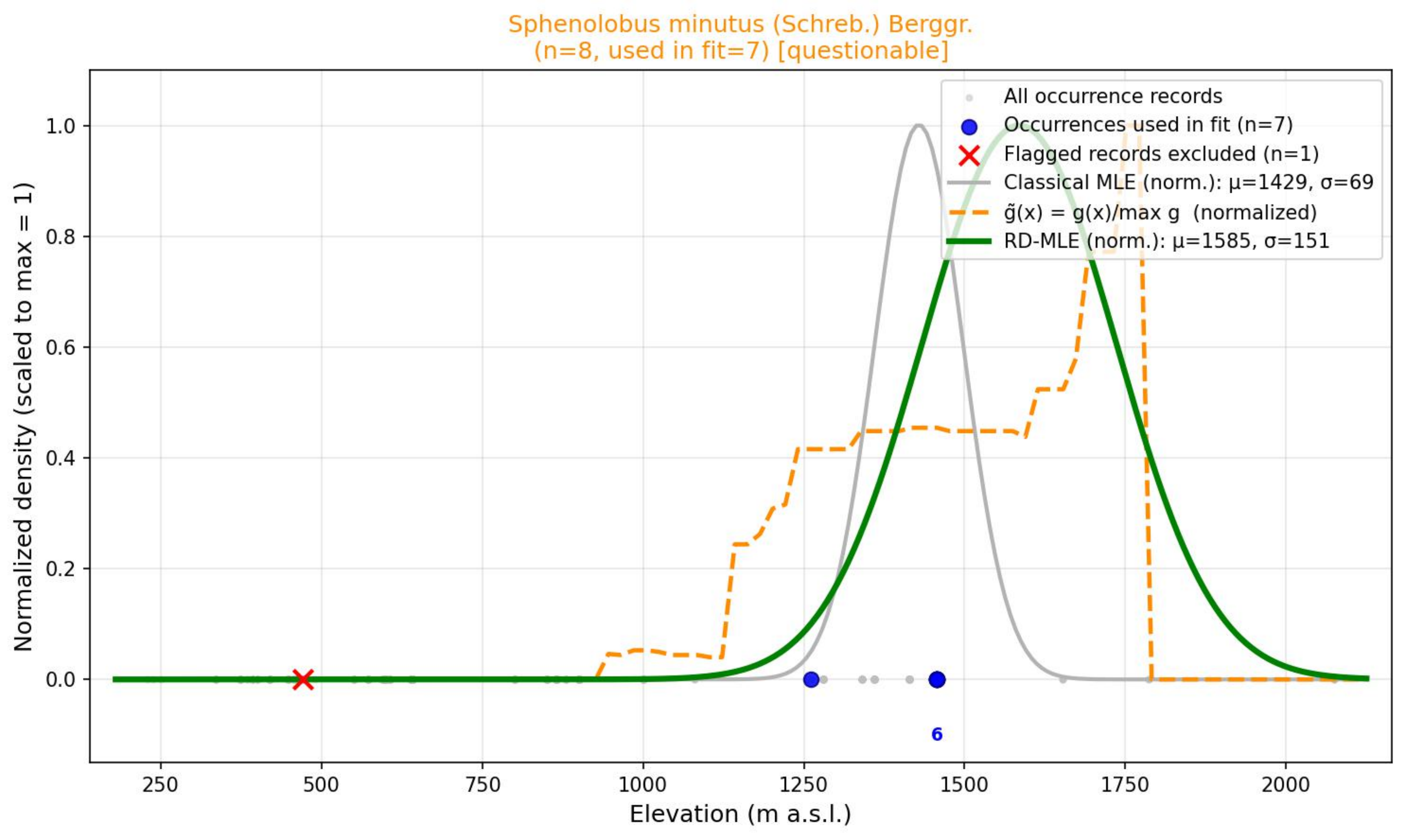
Sphenolobus minutus (Schreb.) Berggr.
(n=8, used in fit=7) [questionable]
All occurrence records
Occurrences used in fit (n=7)
Flagged records excluded (n=1)
Classical MLE (norm.): μ=1429, σ=69
g̃(x) = g(x)/max g (normalized)
RD-MLE (norm.): μ=1585, σ=151
Normalized density (scaled to max = 1)
0.0
0.2
0.4
0.6
0.8
1.0
6
250
500
750
1000
1250
1500
1750
2000
Elevation (m a.s.l.)

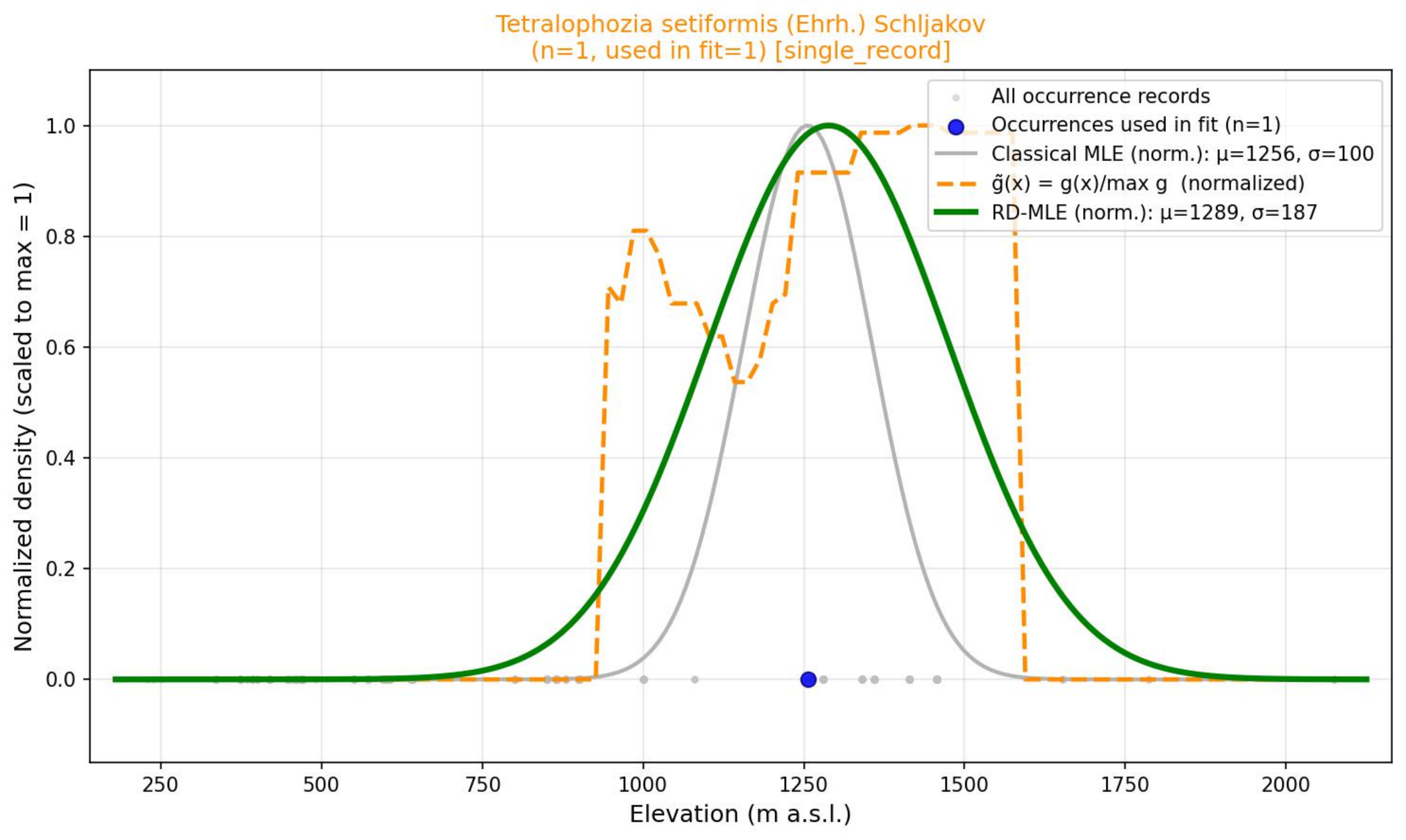

Tetralophozia setiformis (Ehrh.) Schljakov
(n=1, used in fit=1) [single_record]
All occurrence records
Occurrences used in fit (n=1)
Classical MLE (norm.): μ=1256, σ=100
g̃(x) = g(x)/max g (normalized)
RD-MLE (norm.): μ=1289, σ=187
Normalized density (scaled to max = 1)
1.0
0.8
0.6
0.4
0.2
0.0
250
500
750
1000
1250
1500
1750
2000
Elevation (m a.s.l.)

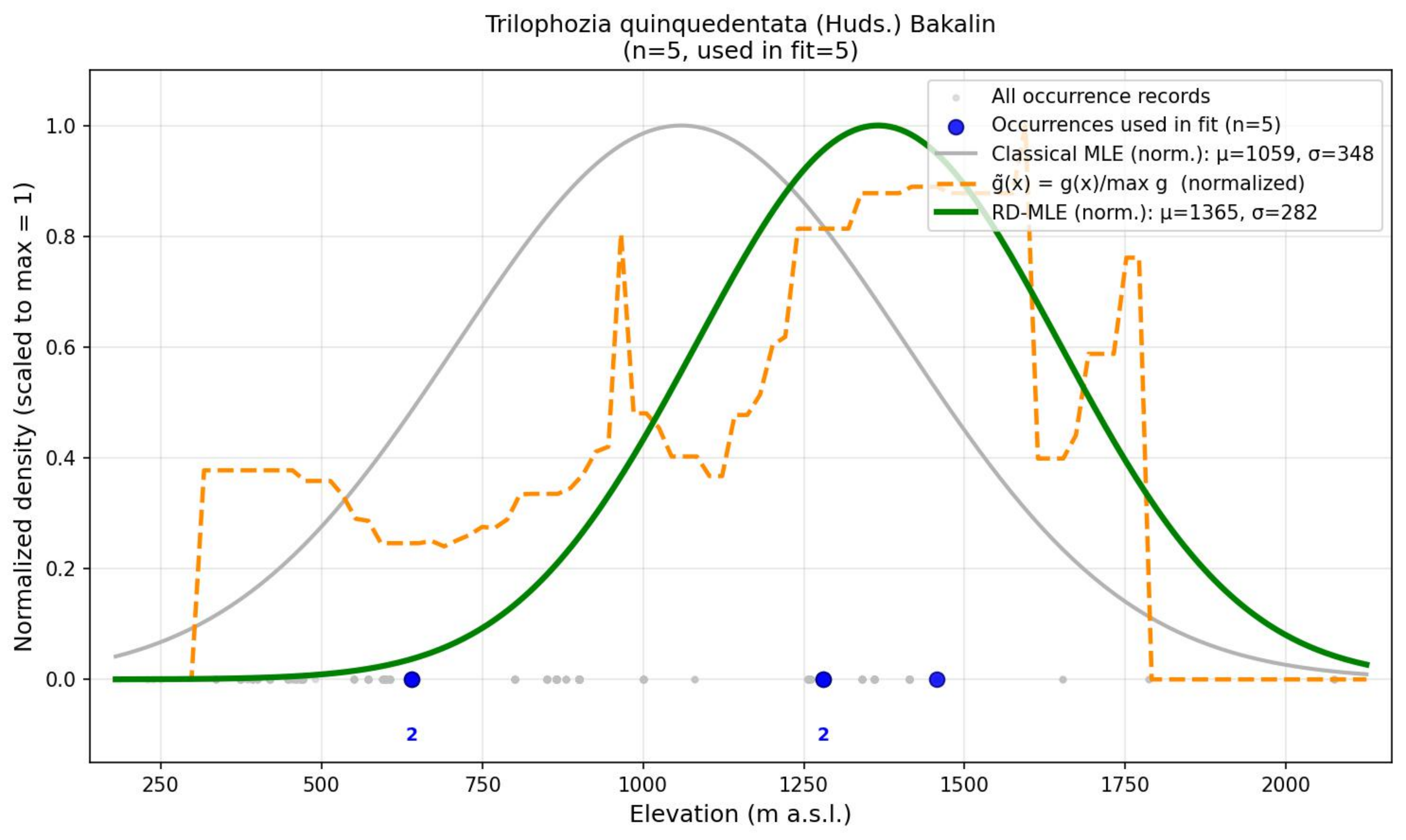

Trilophozia quinquedentata (Huds.) Bakalin
(n=5, used in fit=5)
All occurrence records
Occurrences used in fit (n=5)
Classical MLE (norm.): μ=1059, σ=348
g̃(x) = g(x)/max g (normalized)
RD-MLE (norm.): μ=1365, σ=282
Normalized density (scaled to max = 1)
Elevation (m a.s.l.)
0.0
0.2
0.4
0.6
0.8
1.0
250
500
750
1000
1250
1500
1750
2000
2
2
