# Supplementary material for: Liverwort altitudinal patterns in East Kamchatka: dividing the indivisible
Source: Front Plant Sci. 2026 Sep 11;17:1904286. doi: 10.3389/fpls.2026.1904286 (PMC13612434; doi:10.3389/fpls.2026.1904286)
Supplement: Supplementary file 2 [file DataSheet2.pdf]

number	date of collection	N	E	m a.s.l.	geographic position	vegetation features
1	17 July 2022	53°30'33.58"	158°46'41.66"	335	Upper course of Levaya Nalycheva River	Mesotrophic mire over travertine strata.
2	17 July 2022	53°30'49.09"	158°45'09.38"	384	Upper course of Levaya Nalycheva River	Old travertine shield with remnant thermal activity.
3	18 July 2022	53°34'30.91"	158°50'17.34"	456	Upper course of Levaya Nalycheva River, Talovskie Hot Springs area	The edge of thermal mire.
4	20 July 2022	53°37'02.72"	158°55'36.08"	1414	Southeast spur of Dzendzur Volcano, narrow SE-facing ridge	Dwarf shrub-lichen tundra with rocky outcrops.
5	20 July 2022	53°37'18.77"	158°55'16.05"	1653	Southeast spur of Dzendzur Volcano, narrow SE-facing ridge	Dwarf shrub-lichen tundra with rocky outcrops.
6	20 July 2022	53°37'30.23"	158°55'07.56"	1786	Southeast spur of Dzendzur Volcano, narrow SE-facing ridge	SE-facing gravelly barrens field.
7	20 July 2022	53°38'08.31"	158°55'23.27"	2075	Southeast spur of Dzendzur Volcano, narrow SE-facing ridge	Vertical NW-facing cliffs near mountain top.
8	21 July 2022	53°36'58.13"	158°56'01.43"	1265	Upper course of Shaibnaya River	Pyroclastic field with many snowbeds.
9	21 July 2022	53°37'16.57"	158°56'23.26"	1340	Upper course of Shaibnaya River, area near Dzendzur volcano fumarole field	Pyroclastic field with many snowbeds.
10	21 July 2022	53°37'35.52"	158°57'32.64"	1457	Dzendzur Pass in upper reaches of Shaibnaya River	Mountain tundra with many rocky outcrops on N-facing steep slope.
11	21 July 2022	53°37'25.28"	158°56'47.97"	1414	Dzendzur Pass area in upper reaches of Shaibnaya River	Old clinker flow near the pass.
12	23 July 2022	53°34'57"	158°54'24"	597	Upper course of the right tributary of Shaibnaya River	Snowbed hollow surrounded by *Alnus fruticosa* thickets.
13	24 July 2022	53°34'18.30"	158°50'23.14"	447	Lower course of Talovaya River	*Betula ermanii* forest with shrub-grass cover (*Calamagrostis langsdorffii* dominating).
14	24 July 2022	53°34'37.43"	158°50'06.54"	471	Lower course of Talovaya River	*Betula ermanii* forest with tall grass (mostly *Filipendula camtschatica*) cover.
15	27 July 2022	53°25'17.43"	158°44'23.72"	573	Area near the waterfall in middle course of Shumnaya River	*Alnus fruticosa* belt.
16	27 July 2022	53°25'59.71"	158°43'42.15"	638	Upper course of left tributary of Shumnaya River	Small stream across grass tundra community surrounded by *Alnus fruticosa* thickets.
17	28 July 2022	53°26'08.97"	158°43'45.98"	606	Upper course of the left tributary of Shumnaya River	Area near mineral *Fe* -rich springs (Aag Springs) in *Alnus fruticosa* belt.

18	28 July 2022	53°26'11.99"	158°43'41.73"	594	Upper course of the left tributary of Shumnaya River, area near sulfur springs	Area near sulphur springs surrounded by *Carex-Juncus* mesotrophic swamp and scattered cold streams (without sulfur) in *Alnus fruticosa* belt.
19	29 July 2022	53°29'13.84"	158°44'21.11"	468	Middle course of Levaya Nalycheva River	Small stream with big boulders across *Filipendula camtschatica* thickets in *Betula ermanii* forest.
20	30 July 2022	53°30'30.58"	158°46'59.26"	420	Middle course of Levaya Nalycheva River	*Betula ermanii* forest with small, sometimes drying up, lake (large pond).
21	31 July 2022	53°26'49.80"	158°39'20.08"	865	Upper reaches of Levaya Nalycheva River	*Alnus fruticosa* belt with several streams, snowbeds and alpine grass-dwarf shrub tundra.
22	01 August 2022	53°26'38.35"	158°38'11.53"	1080	Pinachevsky Pass, E-facing slope just below the pass	*Alnus fruticosa* thickets with rocky outcrops.
23	01 August 2022	53°26'56.66"	158°38'15.56"	1261	Pinachevsky Pass, massive cliffs northward of the pass	Cliffs with scattered dwarf shrubs around.
24	01 August 2022	53°27'32.86"	158°35'37.56"	641	Upper course of Pinacheva River	Mesotrophic ridge-hollow swampy complex surrounded by *Alnus fruticosa* thickets.
25	02 August 2022	53°23'27.43"	158°26'04.10"	239	Middle course of Pinacheva River	*Alnus hirsuta* floodplain forest.
