# Supplementary material for: Liverwort altitudinal patterns in East Kamchatka: dividing the indivisible
Source: Front Plant Sci. 2026 Sep 11;17:1904286. doi: 10.3389/fpls.2026.1904286 (PMC13612434; doi:10.3389/fpls.2026.1904286)
Supplement: Supplementary file 3 [file DataSheet3.pdf]

Species	Altitudes	CoordIDs (	verified habitats	BF	CF	TB	PF	AH	repetitive altitude	literature data repetitive a	FieldIDs	Additionals
Aneura maxima (Schiffn.) Steph.	335	14	Betula ermanii forest belt; over the peat on hammock side in mesotrophic mire over travertive strata.	1					*335*, *335*, *335*		K-43-1-22, K-43-3-22, K-43-7-22 (arch.)	Calypogeia kamchatica Bakalin, A.V. Troitsky et Maltseva
Aneura pinguis (L.) Dumort.	420, 1261	15, 18	Betula ermanii forest belt; temporarily flooded humus on the slope to the sometimes flooded and drying up large pond edge. Mountain tundra belt; open moist cliff crevice.	1		1			*420*, *1261*	*374*, *374*, *374*, *388*, *460*, *460*, *1280*, *1280*, *450*	K-62-9-22, K-65-2-22	Odontoschisma elongatum (Lindb.) A. Evans, Radula polyclada A. Evans
Anthelia juratzkana (Limpr.) Trevis.	865, 1265, 1340, 1414, 1457, 1786	2, 3, 4, 6, 8, 19	Alnus fruticosa belt; open moist boulders near the streams and moist clayish soi in snowbed habitat. Mountain tundra belt; open moist clayish soil in cliff crevices and steep slopes, also over cliffs and rocks covered with thin layer of soil. Pyroclastic fields; moist fine soil in snowbed habitats and along streams, clinker crevices.		1	1	1		*1786*, *1256*, *1256*, *1256*, *1256*, *1256*, *1340*, *1457*, *1457*, *1457*, *1457*, *1457*, *1457*, *1457*, *1457*, *1457*, *1414*, *1414*, *1414*, *865*, *865*, *865*, *865*, *865*	*1360*, *1360*, *1360*, *800*, *900*, *900*	K-48-2-22, K-50-3-22, K-50-4-22, K-50-5-22, K-50-6-22, K-50-7-22, K-51-3-22, K-52-10-22, K-52-14-22, K-52-2-22, K-52-23-22, K-52-24-22, K-52-4-22, K-52-5-22, K-52-6-22, K-52-7-22, K-53-10-22, K-53-12-22, K-53-9-22, K-63-11-22, K-63-20-22, K-63-22-22, K-63-25-22, K-63-26-22	Cephalozia bicuspidata (L.) Dumort., Cryptocolea imbricata R.M. Schust., Cryptocoleopsis imbricata Amak., Diplophyllum taxifolium (Wahlenb.) Dumort., Gymnomitrion commutatum (Limpr.) Schiffn., Gymnomitrion concinnatum (Lightf.) Corda, Lophozia savicziae Schljakov, Lophozia ventricosa (Dicks.) Dumort., Marsupella apiculata Schiffn., Marsupella condensata (Ångstr. ex C. Hartm.) Lindb. ex Kaal., Nardia breidleri (Limpr.) Lindb., Nardia geoscyphus (De Not.) Lindb., Nardia insecta Lindb., Nardia pacifica Bakalin, Pleurocladula albescens (Hook.) Grolle, Prasanthus suecicus (Gottsche) Lindb., Pseudolophozia debiliformis (R.M. Schust. & Damsh.) Konstant. & Vilnet, Pseudolophozia sudetica (Nees ex Huebener) Konstant. & Vilnet, Schistochilopsis pacifica Bakalin, Solenostoma sphaerocarpum (Hook.) Steph., Solenostoma subellipticum (Lindb. ex Heeg) R.M. Schust., Sphenolobus minutus (Schreb.) Berggr.
Barbilophozia hatcheri (A. Evans) Loeske	447, 865, 1080	12, 19, 20	Betula ermanii forest belt; mesic Betula trunk base. Alnus fruticosa belt; open moist clayish soil in snowbed and moist cliff crevices.	1	1	1			*447*, *865*, *1080*	*1280*	K-55-7-22 (gemm.), K-63-10-22 (a form with bilobed leaves and no gemmae), K-64-2-22 (gemm.)	Diplophyllum taxifolium (Wahlenb.) Dumort., Pleurocladula albescens (Hook.) Grolle
Barbilophozia lycopodioides (Wallr.) Loeske	447, 468	12, 16	Betula ermanii forest with shrub-grass cover; partly shaded clayish soil on trail cut and moist boulders near streams.	1					*447*, *468*, *468*		K-55-3-22, K-61-25-22, K-61-26-22	Harpanthus flotovianus (Nees) Nees, Marchantia alpestris (Nees) Burgeff, Pellia neesiana (Gottsche) Limpr.
Blepharostoma brevirete (Bryhn & Kaal.) Vilnet & Bakalin						1				*1280*, *1280*, *1280*		
Blepharostoma primum Vilnet et Bakalin	468	16	Betula ermanii forest with Filipendula camtschatica tall grass around; open moist boulder near the stream.	1					*468*		K-61-13-22 (gemm.)	
Calycularia laxa Lindb. & Arnell	597	9	Alnus fruticosa belt; moist tufa cliff in part shade near the snowbed.		1				*597*, *597*	*640*	K-54-7-22 (ant.), K-54-9-22	Diplophyllum taxifolium (Wahlenb.) Dumort., Nardia insecta Lindb., Pleurocladula albescens (Hook.) Grolle
Calypogeia integristipula Steph.	638	23	Alnus fruticosa belt; partly shaded humus on a slope to stream in grass tundra covered valley.		1				*638*		K-58-10-22	Lophozia longiflora (Nees) Schiffn.
Calypogeia kamchatica Bakalin, A.V. Troitsky et Maltseva	335, 456, 471, 641, 865	10, 11, 14, 17, 19	Betula ermanii belt; open moist boulders near streams, including Sulfur rich outcomings and open thermal swamps on travertine streata on percolated Sulfur rich waters.	1	1				*335*, *456*, *456*, *456*, *471*, *471*, *471*, *471*, *471*, *865*, *641*, *641*, *641*	*393*, *393*, *460*, *460*, *460*, *550*	K-43-1-22, K-45-1-22, K-45-2-22, K-45-3-22, K-56-10-22, K-56-11-22, K-56-13-22, K-56-16-22, K-56-3-22 (gemm.), K-63-19-22 (mod. <i>parviretis</i>), K-66-1-22, K-66-3-22, K-66-4-22	Aneura maxima (Schiffn.) Steph., Cephalozia bicuspidata (L.) Dumort., Harpanthus flotovianus (Nees) Nees, Pellia neesiana (Gottsche) Limpr., Scapania pseudouliginosa Potemkin, Bakalin, Vilnet & Klimova, Schistochilopsis pacifica Bakalin, Solenostoma subellipticum (Lindb. ex Heeg) R.M. Schust.
Calypogeia muelleriana (Schiffn.) Müll. Frib.						1				*850*, *850*		
Cephalozia bicuspidata (L.) Dumort.	420, 573, 594, 597, 638, 641, 865, 1340, 1457	2, 6, 9, 15, 17, 19, 21, 23, 24	Betula ermanii belt; open moist boulders near streams, including Sulfur rich outcomings, open thermal swamps on travertine strata on percolated Sulfur rich waters, open moist degraded travertine strata. Alnus fruticosa belt; open to partly shaded moist fine soil, peaty soil and boulders alond streams (including those with Sulfur rich waters). Mountain tundra belt; open moist cliffs, fine soil near the streams and in snowbed habitats. Pyroclastic fields; open moist clayish soil and scoria near streams going from glaciers.	1	1	1	1		*1340*, *1340*, *1340*, *1457*, *1457*, *597*, *573*, *573*, *573*, *573*, *573*, *638*, *638*, *638*, *594*, *594*, *594*, *594*, *594*, *420*, *420*, *865*, *865*, *641*, *641*, *641*, *641*	*393*, *1360*, *1360*, *850*, *850*, *850*, *850*, *1000*, *1000*, *1000*, *1000*, *1000*, *1000*, *900*, *900*, *900*	K-51-1-22 (black colored suppressed plants), K-51-5-22, K-51-6-22, K-52-25-22, K-52-4-22, K-54-13-22, K-57-10-22, K-57-11-22, K-57-12-22, K-57-3-22, K-57-4-22, K-58-14-22, K-58-3-22 (per., ant., spor.), K-58-9-22, K-60-1-22, K-60-2-22, K-60-3-22 (a form with underleaves), K-60-4-22, K-60-5-22, K-62-11-22, K-62-16-22, K-63-21-22, K-63-22-22, K-66-1-22, K-66-14-22, K-66-6-22, K-66-8-22	Anthelia juratzkana (Limpr.) Trevis., Calypogeia kamchatica Bakalin, A.V. Troitsky et Maltseva, Cryptocoleopsis imbricata Amak., Diplophyllum taxifolium (Wahlenb.) Dumort., Fuscocephaloziopsis pleniceps (Austin) Váňa & L. Söderstr., Gymnomitrion concinnatum (Lightf.) Corda, Harpanthus flotovianus (Nees) Nees, Lophozia savicziae Schljakov, Marsupella boeckii (Austin) Lindb. ex Kaal., Marsupella tubulosa Steph., Nardia insecta Lindb., Nardia japonica Steph., Odontoschisma elongatum (Lindb.) A. Evans, Pellia neesiana (Gottsche) Limpr., Pleurocladula albescens (Hook.) Grolle, Prasanthus suecicus (Gottsche) Lindb., Pseudolophozia cf. debiliformis (R.M. Schust. & Damsh.) Konstant. & Vilnet, Pseudolophozia debiliformis (R.M. Schust. & Damsh.) Konstant. & Vilnet, Pseudolophozia sudetica (Nees ex Huebener) Konstant. & Vilnet, Scapania cf. subalpina (Nees ex Lindenb.) Dumort., Scapania paludicola Loeske & Müll. Frib., Scapania subalpina (Nees ex Lindenb.) Dumort., Schistochilopsis pacifica Bakalin, Solenostoma sphaerocarpum (Hook.) Steph., Solenostoma subellipticum (Lindb. ex Heeg) R.M. Schust., Sphenolobus minutus (Schreb.) Berggr.
Cephalozia otaruensis Steph.	865	19	Alnus fruticosa belt; open wet boulder near stream.		1				*865*		K-63-3-22 (ant.)	
Cephaloziella arctogena (R.M. Schust.) Konstant.	447	12	Betula ermanii forest belt; open moist fallen decaying decorticated Betula trunk.	1					*447*		K-55-5-22 (ant., per., arch., spor.; paroicous)	
Cephaloziella divaricata (Sm.) Schiffn.	384, 420, 641, 2075	1, 13, 15, 17	Betula ermanii forest belt; open moist clayishg roadside, moist degrading old travertive sheet. Alnus fruticosa belt; over peat in open wet hollow in mesotrophic swamp. Alpine heathlands; moist cliff crevice in part shade.	1	1			1	*384*, *2075*, *420*, *420*, *641*, *641*	*393*, *393*, *393*, *393*, *393*, *393*	K-44-1-22 (arch.), K-49-2-22, K-62-3-22, K-62-3a-22, K-66-10-22 (per., ant., spor.), K-66-12-22	Fossombronia alaskana Steere & Inoue, Odontoschisma elongatum (Lindb.) A. Evans, Saccobasis polita (Nees) H. Buch, Scapania obcordata (Berggr.) S.W. Arnell, Scapania paludicola Loeske & Müll. Frib.
Cephaloziella rubella (Nees) Warnst.				1						*400*		
Cephaloziella varians (Gottsche) Steph.							1			*1360*		
Chiloscyphus polyanthos (L.) Corda	468, 471	10, 16	Betula ermanii forest; open to partly shaded moist humus and wet boulders along streams including those with Sulfur rich water.	1					*471*, *468*, *468*, *468*, *468*, *468*	*600*, *600*	K-56-12-22 (ant., arch.), K-61-4-22, K-61-5-22, K-61-6-22, K-61-8-22, K-61-9-22 (ant., arch.)	Conocephalum salebrosum Szweyk., Buczk. & Odrzyk., Jungermannia exsertifolia Steph., Pellia neesiana (Gottsche) Limpr., Scapania subalpina (Nees ex Lindenb.) Dumort.
Conocephalum salebrosum Szweyk., Buczk. & Odrzyk.	468, 471, 573	10, 16, 24	Betula ermanii forest; open moiost clayish soil and humus covering rocks along streams, including those with Sulfur rich waters. Alnus fruticosa belt; open moist clayish soil covering rocks.	1	1	1			*471*, *471*, *573*, *468*, *468*	*600*, *1000*, *374*	K-56-12-22, K-56-14-22, K-57-2-22, K-61-22-22, K-61-4-22	Chiloscyphus polyanthos (L.) Corda, Jungermannia aff. pumila With., Pellia neesiana (Gottsche) Limpr., Scapania subalpina (Nees ex Lindenb.) Dumort., Solenostoma confertissimum (Nees) Schljakov, Solenostoma subellipticum (Lindb. ex Heeg) R.M. Schust.
Cryptocolea imbricata R.M. Schust.	1457	2	Mountain tundra belt; open moist clayish soil covering cliff on N-facing steep slope.			1			*1457*		K-52-2-22 (ant., arch.)	Anthelia juratzkana (Limpr.) Trevis., Gymnomitrion commutatum (Limpr.) Schiffn., Lophozia savicziae Schljakov, Nardia insecta Lindb., Solenostoma sphaerocarpum (Hook.) Steph.

Cryptocoleopsis imbricata Amak.	597, 1414, 1457	2, 7, 9	Alnus fruticosa belt; moist tufa cliffs on steep slope to snowbed habitat. Mountain tundra belt; open to partly shaded cliff crevices includign those filled with thin layer of fine soils.		1	1			*1414*, *1457*, *1457*, *1457*, *1457*, *1457*, *1457*, *1457*, *1457*, *597*	*1280*, *1280*, *1280*, *1280*, *1280*, *1280*	K-46-4-22 (arch.), K-52-12-22, K-52-13-22, K-52-19-22 (ant., arch.), K-52-24-22, K-52-3-22, K-52-4-22, K-52-5-22, K-52-7-22 (ant., spor. sol.), K-54-11-22 (ant.)	Anthelia juratzkana (Limpr.) Trevis., Cephalozia bicuspidata (L.) Dumort., Diplophyllum albicans (L.) Dumort., Diplophyllum taxifolium (Wahlenb.) Dumort., Gymnomitrion concinnatum (Lightf.) Corda, Marsupella apiculata Schiffn., Marsupella condensata (Ångstr. ex C. Hartm.) Lindb. ex Kaal., Pseudolophozia sudetica (Nees ex Huebener) Konstant. & Vilnet, Schistochilopsis incisa (Schrad.) Konstant., Sphenolobus minutus (Schreb.) Berggr.
Diplophyllum albicans (L.) Dumort.	573, 1414, 1457, 1653	2, 4, 5, 7, 24	Alnus fruticosa belt; moist boulder on steep slope to valley. Mountain tundra belt; partly shaded mesic to moist cliff crevices including those filled with thin layer of sine soils. Alpine heathlands; crevices in old clinker flow near the pass.		1	1		1	*1414*-1-22, *1653*-2-22, *1457*-16-22, *1457*-3-22, *1414*-8-22, *573*-1-22	*1280*	K-46-1-22, K-47-2-22, K-52-16-22, K-52-3-22, K-53-8-22, K-57-1-22	Cryptocoleopsis imbricata Amak., Diplophyllum taxifolium (Wahlenb.) Dumort., Gymnomitrion concinnatum (Lightf.) Corda, Lophozia savicziae Schljakov, Marsupella apiculata Schiffn., Marsupella condensata (Ångstr. ex C. Hartm.) Lindb. ex Kaal., Schistochilopsis incisa (Schrad.) Konstant.
Diplophyllum taxifolium (Wahlenb.) Dumort.	420, 468, 471, 573, 597, 606, 638, 865, 1261, 1265, 1414, 1457	2, 4, 7, 8, 9, 10, 15, 16, 18, 19, 22, 23, 24	Betula ermanii forest belt; open mesic tufa conglomerates near the stream, temporarity flooded peaty bank of ladge pond. Alnus fruticosa belt; open to partly shaded moist to mesic soil near streams (inclusing those with Sulfur rich waters), partly shaded tufa cliffs near snowbed. Mountain tundra belt; open to partly shaded cliff crevices, fine soil in snowbed, soils and bounders along streams. Pyroclastic fields; open moist boulders near the stream.	1	1	1	1		*1414*, *1256*, *1256*, *1457*, *1457*, *1457*, *1457*, *1457*, *1414*, *597*, *597*, *597*, *597*, *597*, *597*, *471*, *573*, *638*, *606*, *606*, *606*, *468*, *420*, *420*, *420*, *865*, *865*, *865*, *865*, *865*, *865*, *1261*	*1360*, *800*, *800*, *640*, *850*	K-46-3-22, K-50-11-22, K-50-6-22, K-52-13-22, K-52-16-22, K-52-16a-22, K-52-17-22, K-52-20-22, K-53-1-22, K-54-12-22, K-54-14-22, K-54-6-22, K-54-7-22, K-54-8-22, K-54-9-22, K-56-6-22, K-57-13-22, K-58-11-22, K-59-14-22, K-59-5-22, K-59-6-22, K-61-19-22 (ant.), K-62-14-22, K-62-15-22, K-62-16-22, K-63-10-22, K-63-13-22, K-63-6-22 (per.), K-63-7-22, K-63-8-22, K-63-9-22, K-65-1-22	Anthelia juratzkana (Limpr.) Trevis., Barbilophozia hatcheri (A. Evans) Loeske, Calycularia laxa Lindb. & Arnell, Cephalozia bicuspidata (L.) Dumort., Cryptocoleopsis imbricata Amak., Diplophyllum albicans (L.) Dumort., Gymnomitrion commutatum (Limpr.) Schiffn., Gymnomitrion concinnatum (Lightf.) Corda, Lophocolea minor Nees, Lophozia longiflora (Nees) Schiffn., Lophozia savicziae Schljakov, Lophozia ventricosa (Dicks.) Dumort., Marsupella apiculata Schiffn., Nardia insecta Lindb., Nardia japonica Steph., Odontoschisma elongatum (Lindb.) A. Evans, Pellia neesiana (Gottsche) Limpr., Pleurocladula albescens (Hook.) Grolle, Pseudolophozia debiliformis (R.M. Schust. & Damsh.) Konstant. & Vilnet, Pseudolophozia sudetica (Nees ex Huebener) Konstant. & Vilnet, Radula polyclada A. Evans, Schistochilopsis incisa (Schrad.) Konstant., Schistochilopsis pacifica Bakalin, Solenostoma subellipticum (Lindb. ex Heeg) R.M. Schust., Sphenolobus minutus (Schreb.) Berggr., Trilophozia quinquedentata (Huds.) Bakalin
Douinia plicata (Lindb.) Konstant. et Vilnet	1414, 1457	2, 7	Mountain tundra belt; open to prtly shaded mesic to moist cliff crevices.			1			*1414*, *1457*	*1280*	K-46-2-22, K-52-1-22	
Endogemma caespiticia (Lindenb.) Konstant., Vilnet & A.V. Troitsky							1			*1360*		
Fossombronia alaskana Steere & Inoue	420	15	Betula ermanii forest belt; open moist clayish roadside.	1					*420*, *420*, *420*		K-62-3-22 (ant., arch.; the identity to <i>F. alaskana</i> checked by cultivated plants from this specimen), K-62-3a-22 (spor.; these plants were cultivated for two months in Petri dish to obtain mature sporophytes), K-62-4-22 (ant., arch.; the identity to <i>F. alaskana</i> checked by cultivated plants from the same site (K-62-3a-22))	Cephaloziella divaricata (Sm.) Schiffn., Odontoschisma elongatum (Lindb.) A. Evans
Frullania austinii J.J. Atwood, Vilnet, Mamontov & Konstant.	239	25	Betula ermanii forest belt; Alnus hirsuta floodplain forest, bark of Alnus in part shade at the height 1.5 m above the ground.	1	1				*239*	*230*, *250*	K-67-1-22	
Frullania subarctica Vilnet, Borovich., Bakalin						1				*1280*, *1280*, *1280*, *1280*, *1280*		
Fuscocephaloziopsis pleniceps (Austin) Váňa & L. Söderstr.	641	17	Alnus fruticosa belt; over peat in open wet hollow in mesotrophic ridge-hollow swampy complex.		1	1			*641*, *641*, *641*	*1280*	K-66-11-22, K-66-6-22 (per., ant.), K-66-8-22	Cephalozia bicuspidata (L.) Dumort., Scapania paludicola Loeske & Müll. Frib.
Gymnocolea inflata (Huds.) Dumort.	420, 573, 1340	6, 15, 24	Betula ermanii forest belt; open moist clayish roadside. Alnus fruticosa belt; open moist clayish soil near stream. Pyroclastic fields; open moist clayish soil near the stream flowing from snowbed.	1	1		1		*1340*, *573*, *420*	*1360*, *1360*, *1360*, *1360*, *1360*	K-51-4-22 (per.), K-57-15-22 (per., ant.), K-62-2-22	Odontoschisma elongatum (Lindb.) A. Evans
Gymnocolea marginata (Steph.) S. Hatt.	573	24	Alnus fruticosa belt; open moist clayish soil near stream.		1				*573*		K-57-17-22 (ant.)	
Gymnomitrion adustum Nees	865	19	Alnus fruticosa belt; open moist boulder near stream.		1				*865*		K-63-24-22 (ant., arch., spor.; paroicous)	
Gymnomitrion alpinum (Gottsche ex Husn.) Schiffn.							1			*1360*		
Gymnomitrion commutatum (Limpr.) Schiffn.	865, 1080, 1457	2, 19, 20	Alnus fruticosa belt; open moist clayish soil in snowbed habitat and moist cliffs and crevices in them including those covered with thin layer of fine soil. Mountian tundra belt; open to partly shaded cliff moist crevices, including those filled with thin layer of fine soil.		1	1			*1457*, *1457*, *1457*, *1457*, *865*, *1080*		K-52-14-22, K-52-2-22, K-52-23-22, K-52-6-22, K-63-8-22, K-64-3-22 (ant.)	Anthelia juratzkana (Limpr.) Trevis., Cryptocolea imbricata R.M. Schust., Diplophyllum taxifolium (Wahlenb.) Dumort., Gymnomitrion concinnatum (Lightf.) Corda, Lophozia savicziae Schljakov, Marsupella apiculata Schiffn., Nardia insecta Lindb., Prasanthus suecicus (Gottsche) Lindb., Pseudolophozia sudetica (Nees ex Huebener) Konstant. & Vilnet, Solenostoma sphaerocarpum (Hook.) Steph.
Gymnomitrion concinnatum (Lightf.) Corda	1261, 1414, 1457, 1653, 2075	1, 2, 4, 5, 18	Mountain tundra belt; open moist cliffs and their crevices including those filled with fine soil. Alpine heathlands; partly shaded mesic crevices in the cliffs, including old clinker flows.			1		1	*1653*, *1653*, *2075*, *1457*, *1457*, *1457*, *1457*, *1457*, *1457*, *1457*, *1414*, *1261*		K-47-1-22 (arch.), K-47-2-22, K-49-3-22, K-52-10-22, K-52-13-22, K-52-14-22, K-52-17-22, K-52-23-22, K-52-25-22, K-52-6-22, K-53-8-22, K-65-5-22 (arch.)	Anthelia juratzkana (Limpr.) Trevis., Cephalozia bicuspidata (L.) Dumort., Cryptocoleopsis imbricata Amak., Diplophyllum albicans (L.) Dumort., Diplophyllum taxifolium (Wahlenb.) Dumort., Gymnomitrion commutatum (Limpr.) Schiffn., Lophozia savicziae Schljakov, Marsupella apiculata Schiffn., Nardia geoscyphus (De Not.) Lindb., Prasanthus suecicus (Gottsche) Lindb., Pseudolophozia sudetica (Nees ex Huebener) Konstant. & Vilnet, Scapania degenii Schiffn. ex Müll. Frib., Schistochilopsis incisa (Schrad.) Konstant., Solenostoma sphaerocarpum (Hook.) Steph., Sphenolobus minutus (Schreb.) Berggr.
Gymnomitrion kamchaticum Mamontov, Vilnet & Konstant.	865, 1414, 1786	3, 4, 19	Alnus fruticosa belt; open moist clayish soil in solifluction spot. Alpine heathlands; open dry crevices in old clinker flows and mesic clayish soil near temporary streams.		1	1		1	*1414*, *865*, *865*, *1786*	*900*	K-53-3-22 (ant., arch., spor. sol.), K-63-4-22 (ant., spor.), K-63-5-22 (ant., spor.)	Marsupella apiculata Schiffn.
Harpanthus flotovianus (Nees) Nees	420, 468, 641, 865	15, 16, 17, 19	Betula ermanii forest belt; moist boulders near the streams and temporarily flooded humus at the edge of laerge pond. Alnus fruticosa belt; open wet hollow in mesotrophic ridge-hollow swampy complex, moist bouders near streams.	1	1	1			*468*, *468*, *468*, *420*, *865*, *865*, *865*, *641*, *641*	*1000*, *1000*, *1000*, *1000*, *900*	K-61-25-22, K-61-26-22, K-61-27-22 (ant.), K-62-10-22, K-63-19-22, K-63-27-22, K-63-28-22, K-66-1-22, K-66-2-22	Barbilophozia lycopodioides (Wallr.) Loeske, Calypogeia kamchatica Bakalin, A.V. Troitsky et Maltseva, Cephalozia bicuspidata (L.) Dumort., Lophozia ventricosa (Dicks.) Dumort., Marchantia alpestris (Nees) Burgeff, Pellia neesiana (Gottsche) Limpr., Saccobasis polita (Nees) H. Buch, Scapania pseudouliginosa Potemkin, Bakalin, Vilnet & Klimova, Schistochilopsis pacifica Bakalin
Hygrobiella intermedia Bakalin & Vilnet						1				*900*, *900*		
Hygrobiella squamosa Bakalin & Vilnet	468, 573, 865	16, 19, 24	Betula ermanii forest belt; open moist boulder near the stream. Alnus fruticosa belt; open moist boulders near the streams.	1	1				*573*, *573*, *468*, *865*		K-57-7-22 (ant.), K-57-9-22, K-61-12-22 (per., ant.), K-63-29-22 (per., ant.)	Jungermannia aff. pumila With., Solenostoma subellipticum (Lindb. ex Heeg) R.M. Schust.

Isopaches bicrenatus (Schmid. ex Hoffm.) H. Buch						1				*900*		
Jungermannia atrovirens Dumort.						1				*900*		
Jungermannia borealis Damsh. & Váňa	471	10	Betula ermanii forest belt; open wet boulders near Sulfur rich stream.	1					*471*		K-56-18-22 (ant., per. unfert.)	
Jungermannia eucordifolia Schljakov	471, 865	10, 19	Betula ermanii forest belt; partly shaded wet boulder near stream with Sulfur rich waters. Alnus fruticosa belt; open moist boulder near stream.	1		1			*471*, *865*	*850*, *1000*, *900*, *900*, *900*, *900*	K-56-8-22, K-63-23-22	Solenostoma subellipticum (Lindb. ex Heeg) R.M. Schust.
Jungermannia exsertifolia Steph.	468, 865	16, 19	Betula wermanii forest belt; open wet boulder near the stream. Alnus fruticosa belt; open wet boulders near the stream.	1	1				*468*, *865*, *865*	*460*, *550*	K-61-8-22, K-63-1-22, K-63-2-22	Chiloscyphus polyanthos (L.) Corda
Jungermannia pumila With. subsp. orientalis Konstant., Vilnet & Mamontov	573	24	Alnus fruticosa belt; open moist to wet boulders andmoist soil covering rocks near the streams.		1	1			*573*, *573*, *573*, *573*	*850*, *900*	K-57-2-22 (ant. per.; oil bodies biconcentric), K-57-5-22 (per., ant.), K-57-8-22 (per., ant.; oil bodies biconcentric), K-57-9-22 (per., ant.; oil bodies biconcentric)	Conocephalum salebrosum Szweyk., Buczk. & Odrzyk., Hygrobiella squamosa Bakalin & Vilnet, Nardia japonica Steph., Solenostoma subellipticum (Lindb. ex Heeg) R.M. Schust.
Lophocolea heterophylla (Schrad.) Dumort.				1						*450*		
Lophocolea minor Nees	420	15	Betula ermanii forest belt; temporarily flooded humus on the slope to a large pond edge.	1					*420*, *420*, *420*	*450*, *400*	K-62-13-22 (gemm.), K-62-14-22, K-62-7-22 (gemm.)	Diplophyllum taxifolium (Wahlenb.) Dumort., Odontoschisma elongatum (Lindb.) A. Evans
Lophozia fuscovirens Bakalin & Vilnet	1457	2	Mountain tundra belt; open moist clayish soil on a steep slope.			1			*1457*		K-52-9-22 (gemm.)	Nardia geoscyphus (De Not.) Lindb.
Lophozia guttulata (Lindb. & Arnell) A. Evans	420, 447, 468	12, 15, 16	Betula ermanii forest belt; open moist fallen decayinf decorticated Betula trunks and decaying barked Betula roots.	1					*447*, *468*, *468*, *420*	*550*	K-55-6-22 (gemm.), K-61-2-22 (oil bodies granulate, spherical, 4-6 per cell), K-61-3-22 (per., ant., spor.), K-62-5-22 (gemm.)	
Lophozia longiflora (Nees) Schiffn.	468, 638	16, 23	Betula ermanii forest belt; open moist boulders nbear the streams. Alnus fruticosa belt; partly shaded moist humus on slope.	1	1	1			*638*, *468*, *468*	*1000*, *900*	K-58-10-22, K-61-15-22 (per., ant., spor.), K-61-19-22	Calypogeia integristipula Steph., Diplophyllum taxifolium (Wahlenb.) Dumort.
Lophozia murmanica Kaal.	468	16	Betula ermanii forest belt; open moist boulder near the stream. Alnus fruticosa belt; open moist boulders near the streams.	1	1		1		*468*	*1360*	K-61-11-22	
Lophozia savicziae Schljakov	597, 865, 1265, 1340, 1414, 1457, 1653, 2075	1, 2, 4, 5, 6, 8, 9, 19	Alnus fruticosa belt; open moist clayish soil in snowbed. Mountain tundra belt; open to partly shaded clayish soil filling cliff crevices and on steep slopes to watercourses. Pyroclastic fields; moist clayish soil near nival streams, over Sphagnum mats in percolate water moistened slope. Alpine heathlands; moist cliff crevice in part shade.		1	1	1	1	*1653*, *2075*, *1256*, *1256*, *1256*, *1340*, *1457*, *1457*, *1457*, *1457*, *1414*, *1414*, *597*, *865*	*1360*, *800*, *1000*, *900*, *900*	K-47-2-22, K-49-1-22, K-50-1-22 (gemm.), K-50-4-22, K-50-7-22 (mod. <i>densiretis</i>), K-51-2-22 (some plants with brownish gemmae, but with similarly numerous biconcentric oil bodies), K-52-11-22, K-52-2-22, K-52-25-22, K-52-8-22, K-53-12-22, K-53-9-22, K-54-12-22 (gemm.), K-63-11-22	Anthelia juratzkana (Limpr.) Trevis., Cephalozia bicuspidata (L.) Dumort., Cryptocolea imbricata R.M. Schust., Diplophyllum albicans (L.) Dumort., Diplophyllum taxifolium (Wahlenb.) Dumort., Gymnomitrion commutatum (Limpr.) Schiffn., Gymnomitrion concinnatum (Lightf.) Corda, Lophoziopsis polaris (R.M. Schust.) Konstant. & Vilnet, Marsupella apiculata Schiffn., Marsupella condensata (Ångstr. ex C. Hartm.) Lindb. ex Kaal., Nardia breidleri (Limpr.) Lindb., Nardia geoscyphus (De Not.) Lindb., Nardia harae Amakawa, Nardia insecta Lindb., Pleurocladula albescens (Hook.) Grolle, Prasanthus suecicus (Gottsche) Lindb., Solenostoma sphaerocarpum (Hook.) Steph., Sphenolobus minutus (Schreb.) Berggr.
Lophozia ventricosa (Dicks.) Dumort.	468, 606, 638, 865	16, 19, 22, 23	Betula ermanii forest belt; open moisyt boulder near the stream. Alnus fruticosa belt; open moist boulders and humus near streams, clayish soil in snowbed habitat.	1	1	1			*638*, *606*, *468*, *865*, *865*, *865*, *865*, *865*, *865*	*850*	K-58-4-22, K-59-6-22 (gemm.), K-61-20-22 (gemm.), K-63-26-22, K-63-28-22, K-63-31-22 (per., ant.), K-63-32-22 (gemm.), K-63-32a-22, K-63-9-22	Anthelia juratzkana (Limpr.) Trevis., Diplophyllum taxifolium (Wahlenb.) Dumort., Harpanthus flotovianus (Nees) Nees, Pellia neesiana (Gottsche) Limpr., Pleurocladula albescens (Hook.) Grolle, Pseudolophozia debiliformis (R.M. Schust. & Damsh.) Konstant. & Vilnet, Schistochilopsis incisa (Schrad.) Konstant.
Lophozia wenzelii (Nees) Steph.						1	1			*1360*, *900*		
Lophoziopsis longidens (Lindb.) Konstant. et Vilnet	447	12	Betula ermanii forest belt; partly shaded mesic Betula ermanii trunk bases and Pinus pumila branches in part shade.	1					*447*, *447*, *447*	*490*, *400*	K-55-1-22, K-55-2-22 (gemm.), K-55-8-22 (gemm.)	Ptilidium pulcherrimum (Weber) Vain.
Lophoziopsis polaris (R.M. Schust.) Konstant. & Vilnet	2075	1	Alpine heathlands; partly shaded moist cliff crevice.					1	*2075*		K-49-1-22 (gemm.)	Lophozia savicziae Schljakov
Marchantia alpestris (Nees) Burgeff	468, 865	16, 19	Betula ermanii forest belt; partly shaded moist boulder near the stream. Alnus fruticosa belt; open moist clayish soil near the stream.	1	1	1			*468*, *865*	*600*, *600*, *850*, *900*, *900*	K-61-26-22, K-63-15-22	Barbilophozia lycopodioides (Wallr.) Loeske, Harpanthus flotovianus (Nees) Nees, Pellia neesiana (Gottsche) Limpr.
Marchantia latifolia Gray				1						*450*		
Marchantia polymorpha L.	597	9	Alnus fruticosa belt; clayish soil near the stream flowing from snowbed habitat, in part shade.		1				*597*		K-54-5-22 (arch.)	
Marsupella apertifolia Steph.	573	24	Alnus fruticosa belt; open moist boulder near stream.		1	1			*573*	*1360*	K-57-6-22	
Marsupella apiculata Schiffn.	1414, 1457, 1786	2, 3, 4	Mountain tundra belt; oopen moist cliff crevices including those filled with thin layer of fine soil. Alpine heathlands; dry to mesic crevices in old clinker flows and mesic clayish soil near temporary streams in rock fields.			1		1	*1786*, *1457*, *1457*, *1457*, *1457*, *1414*, *1414*, *1414*, *1414*, *1414*, *1414*, *1414*		K-48-1a-22, K-52-13-22, K-52-14-22, K-52-3-22, K-52-5-22, K-53-11-22, K-53-12-22, K-53-2-22, K-53-3-22, K-53-4-22, K-53-6-22, K-53-9-22	Anthelia juratzkana (Limpr.) Trevis., Cryptocoleopsis imbricata Amak., Diplophyllum albicans (L.) Dumort., Diplophyllum taxifolium (Wahlenb.) Dumort., Gymnomitrion commutatum (Limpr.) Schiffn., Gymnomitrion concinnatum (Lightf.) Corda, Gymnomitrion kamchaticum Mamontov, Vilnet & Konstant., Lophozia savicziae Schljakov, Marsupella condensata (Ångstr. ex C. Hartm.) Lindb. ex Kaal., Pleurocladula albescens (Hook.) Grolle, Schistochilopsis incisa (Schrad.) Konstant., Sphenolobus minutus (Schreb.) Berggr.
Marsupella boeckii (Austin) Lindb. ex Kaal.	597, 1265	8, 9	Alnus fruticosa belt; moist tufa cliff in part shade. Pyroclastic field; open miost boulder near nival stream.		1		1		*1256*, *597*, *597*		K-50-9-22 (ant., per.), K-54-10-22 (per., spor.), K-54-13-22	Cephalozia bicuspidata (L.) Dumort., Marsupella tubulosa Steph., Nardia insecta Lindb., Pleurocladula albescens (Hook.) Grolle
Marsupella condensata (Ångstr. ex C. Hartm.) Lindb. ex Kaal.	1265, 1340, 1414, 1457	2, 4, 6, 8	Mountain tundra belt; open moist N-facing cliff. Pyroclastic fields; open dry crevices in old clinker, moist clayish soil near stream from snowbed habitat.			1	1		*1256*, *1256*, *1340*, *1340*, *1457*, *1414*, *1414*, *1414*, *1414*, *1414*, *1414*, *1414*, *1414*	*1360*	K-50-3-22 (spor. sparse), K-50-4-22, K-51-2-22, K-51-3-22, K-52-3-22 (ant., arch., spor.), K-53-10-22, K-53-11-22, K-53-12-22 (ant., arch., spor.), K-53-2-22, K-53-4-22, K-53-5-22, K-53-6-22 (ant., per.), K-53-9-22 (ant., arch.)	Anthelia juratzkana (Limpr.) Trevis., Cryptocoleopsis imbricata Amak., Diplophyllum albicans (L.) Dumort., Lophozia savicziae Schljakov, Marsupella apiculata Schiffn., Nardia breidleri (Limpr.) Lindb., Pleurocladula albescens (Hook.) Grolle
Marsupella funckii (F.Weber et Mohr) Dumort.						1				*900*		
Marsupella tubulosa Steph.	468, 597	9, 16	Betula ermanii forest belt; open moist boulder near the stream. Alnus fruticosa belt; open to partly shaded moist tufa cliffs.	1	1	1			*597*, *597*, *468*, *468*	*1280*	K-54-1-22 (per.; oil bodies biconcentric), K-54-13-22, K-61-18-22, K-61-10-22 (oil bodies biconcentric, but no red or purple pigmentation present)	Cephalozia bicuspidata (L.) Dumort., Marsupella boeckii (Austin) Lindb. ex Kaal., Nardia insecta Lindb., Pseudolophozia debiliformis (R.M. Schust. & Damsh.) Konstant. & Vilnet
Mesoptychia rutheana (Limpr.) L. Söderstr. & Váňa	335	14	Betula ermanii forest belt; peat on hummock side in mesotrophic mire developed over travertine strata.	1					*335*	*374*, *374*, *374*, *374*, *374*, *374*, *374*, *374*	K-43-5-22	Riccardia chamedryfolia (With.) Grolle
Metasolenostoma ochotense (Bakalin & Vilnet) Vilnet & Bakalin	420	15	Betula ermanii forest belt; open moist clayish roadside.	1					*420*		K-62-1-22 (per. juv.)	
Metasolenostoma orientale Bakalin & Vilnet	471	10	Betula ermanii forest belt; open wet and submerged boulders in the stream with Sulfur rich waters.	1					*471*, *471*		K-56-1-22 (mod. <i>leptoderma-viridis</i>), K-56-17-22 (ant.)	

Moerckia flotoviana (Nees) Schiffn.	335	14	Betula ermanii forest belt; over the peat on hummock side in mesotrophic mire developed over travertine strata;.	1	1				*335*	*374*, *374*, *374*, *374*, *374*, *374*, *374*, *550*, *550*, *550*	K-43-2-22 (arch.)	
Nardia breidleri (Limpr.) Lindb.	638, 865, 1265, 1340	6, 8, 19, 23	Alnus fruticosa belt; open moist clayish soil in snowbed and moist scoria on steep slope to stream. Pyroclastic fields; open moist to wet sleyish sloil near streams and in snowbeds.		1	1	1		*1256*, *1340*, *1340*, *638*, *865*	*1360*, *1360*, *1360*, *1360*, *1360*, *1360*, *800*, *800*, *900*	K-50-8-22 (ant., per., spor.), K-51-3-22, K-51-7-22 (hydromorphous phase), K-58-7-22 (oil bodies by 1 per cell), K-63-11-22	Anthelia juratzkana (Limpr.) Trevis., Lophozia savicziae Schljakov, Marsupella condensata (Ångstr. ex C. Hartm.) Lindb. ex Kaal., Pleurocladula albescens (Hook.) Grolle
Nardia geoscyphus (De Not.) Lindb.	1457	2	Mountain tundra belt; open moist clayish soil on a steep slope.		1	1			*1457*, *1457, *1457*	*800*, *1280*, *850*	K-52-10-22 (ant., arch.), K-52-8-22, K-52-9-22	Anthelia juratzkana (Limpr.) Trevis., Gymnomitrion concinnatum (Lightf.) Corda, Lophozia fuscovirens Bakalin & Vilnet, Lophozia savicziae Schljakov, Pseudolophozia sudetica (Nees ex Huebener) Konstant. & Vilnet
Nardia geoscyphus (De Not.) Lindb. var. bifida R.M. Schust.					1					*800*, *640*		
Nardia harae Amakawa	606, 1457	2, 22	Alnus fruticosa belt; open wet clayish soil near the streams with Fe-rich waters. Mountain tundra belt; open moist clayish soil on steep slope.		1	1			*1457*, *606*, *606*, *606*, *606*		K-52-11-22, K-59-10-22, K-59-12-22, K-59-3-22 (arch.), K-59-4-22 (arch.)	Lophozia savicziae Schljakov, Nardia pacifica Bakalin, Pellia neesiana (Gottsche) Limpr., Solenostoma sphaerocarpum (Hook.) Steph.
Nardia insecta Lindb.	471, 597, 1457	2, 9, 10	Betula ermanii forest belt; open mesic to moist tufa conglomerate near the stream. Alnus fruticosa belt; moist tufa cliff near nival hollow. Mountain tundra belt; open moist clayish soil covering rocks.	1	1	1			*1457*, *597*, *597*, *597*, *471*, *471*		K-52-2-22, K-54-12-22, K-54-13-22, K-54-9-22 (ant., arch.), K-56-6-22, K-56-7-22 (per., ant. sol.)	Anthelia juratzkana (Limpr.) Trevis., Calycularia laxa Lindb. & Arnell, Cephalozia bicuspidata (L.) Dumort., Cryptocolea imbricata R.M. Schust., Diplophyllum taxifolium (Wahlenb.) Dumort., Gymnomitrion commutatum (Limpr.) Schiffn., Lophozia savicziae Schljakov, Marsupella boeckii (Austin) Lindb. ex Kaal., Marsupella tubulosa Steph., Pleurocladula albescens (Hook.) Grolle, Solenostoma sphaerocarpum (Hook.) Steph., Solenostoma subellipticum (Lindb. ex Heeg) R.M. Schust.
Nardia japonica Steph.	573, 606, 638, 865	19, 22, 23, 24	Alnus fruticosa belt; open moist clayish soil and boulders along streams, including those with Fe-rich waters, rarerly humus on steep slopes.		1	1			*573*, *638*, *638*, *638*, *638*, *638*, *638*, *606*, *606*, *606*, *865*	*850*, *900*, *900*, *900*, *900*, *900*, *900*, *900*, *900*, *800*, *800*, *880*	K-57-8-22, K-58-1-22, K-58-11-22, K-58-2-22, K-58-5-22, K-58-6-22, K-58-9-22 (oil bodies coarsely granulate), K-59-13-22, K-59-5-22, K-59-7-22, K-63-14-22	Cephalozia bicuspidata (L.) Dumort., Diplophyllum taxifolium (Wahlenb.) Dumort., Jungermannia aff. pumila With., Nardia pacifica Bakalin, Pellia neesiana (Gottsche) Limpr., Pseudolophozia sudetica (Nees ex Huebener) Konstant. & Vilnet
Nardia pacifica Bakalin	573, 606, 638, 865	19, 22, 23, 24	Alnus fruticosa belt; open moist clayish soil and boulders along streams, including those with Fe-rich waters, rarerly humus on steep slopes.		1	1			*573*, *638*, *606*, *606*, *606*, *865*, *865*	*900*, *900*, *900*, *900*	K-57-14-22, K-58-8-22, K-59-10-22, K-59-7-22, K-59-8-22 (oil bodies homogeneous), K-63-17-22 (ant.), K-63-20-22	Anthelia juratzkana (Limpr.) Trevis., Nardia harae Amakawa, Nardia japonica Steph., Schistochilopsis pacifica Bakalin
Neoorthocaulis attenuatus (Mart.) L. Söderstr., De Roo & Hedd.				1						*400*		
Odontoschisma elongatum (Lindb.) A. Evans	420, 641	15, 17	Betula ermanii forest belt; open moisyt clayish and humificated soil in roadside and in temporarily flooded large pond edge. Alnus fruticosa belt; open wet peat in hollow in mesotrophic ridge-hollow swampy community.	1	1				*420*, *420*, *420*, *420*, *420*, *420*, *420*, *641*, *641*, *641*, *641*, *641*, *641*, *641*		K-62-14-22, K-62-16-22 (gemm.), K-62-2-22, K-62-3-22, K-62-4-22, K-62-8-22, K-62-9-22, K-66-13-22, K-66-14-22, K-66-15b-22, K-66-15c-22, K-66-15d-22, K-66-15e-22, K-66-15f-22	Aneura pinguis (L.) Dumort., Cephalozia bicuspidata (L.) Dumort., Cephaloziella divaricata (Sm.) Schiffn., Diplophyllum taxifolium (Wahlenb.) Dumort., Fossombronia alaskana Steere & Inoue, Gymnocolea inflata (Huds.) Dumort., Lophocolea minor Nees, Pellia neesiana (Gottsche) Limpr., Riccardia chamedryfolia (With.) Grolle, Saccobasis polita (Nees) H. Buch, Scapania cf. paludicola Loeske & Müll. Frib., Scapania paludicola Loeske & Müll. Frib.
Odontoschisma macounii (Austin) Underw.						1				*1280*, *1280*		
Pellia neesiana (Gottsche) Limpr.	384, 420, 456, 468, 471, 606, 641, 865	10, 11, 13, 15, 16, 17, 19, 22	Betula ermanii forest belt; open to partly shaded moist to wet boulders including those covered with fine soil and humificated soil manks along streams, including those with high Sulfur content, moist degrading travertine sheets. Alnus fruticosa belt; open wet peat in hollows of mesotrophic swampy communities, open moist boulders along streams, partly shaded wet humus along thermal outlets.	1	1	1			*384*, *456*, *471*, *606*, *606*, *606*, *468*, *468*, *468*, *420*, *865*, *641*, *641*	*393*, *460*, *460*, *600*, *600*, *600*, *600*, *850*, *850*, *850*, *850*, *1000*, *400*, *900*, *900*, *900*	K-44-3-22 (ant.), K-45-1-22 (ant.), K-56-11-22, K-59-13-22, K-59-4-22, K-59-6-22, K-61-26-22, K-61-4-22, K-61-6-22, K-62-10-22, K-63-27-22, K-66-14-22, K-66-4-22	Barbilophozia lycopodioides (Wallr.) Loeske, Calypogeia kamchatica Bakalin, A.V. Troitsky et Maltseva, Cephalozia bicuspidata (L.) Dumort., Chiloscyphus polyanthos (L.) Corda, Conocephalum salebrosum Szweyk., Buczk. & Odrzyk., Diplophyllum taxifolium (Wahlenb.) Dumort., Harpanthus flotovianus (Nees) Nees, Lophozia ventricosa (Dicks.) Dumort., Marchantia alpestris (Nees) Burgeff, Nardia harae Amakawa, Nardia japonica Steph., Odontoschisma elongatum (Lindb.) A. Evans, Scapania subalpina (Nees ex Lindenb.) Dumort., Solenostoma subellipticum (Lindb. ex Heeg) R.M. Schust.
Peltolepis quadrata (Saut.) Müll. Frib.	597	9	Alnus fruticosa belt; moist crevice in tufa cliffs filled with fine soil near nival hollow.		1	1			*597*	*850*	K-54-2-22 (arch.)	
Pleurocladula albescens (Hook.) Grolle	597, 865, 1265, 1340, 1414, 1457	2, 4, 6, 8, 9, 19	Alnus fruticosa belt; open moist boulders near the streams, moist clayish soil in snowbed habitats, moist open to partly shaded tufa cliffs near nival hollow. Mountain tundra belt; open moist clayish soil in the cliff crevice. Pyriclastic fields: open moist to wet clayish soil near the stream, fine soil in snowbed habitats, mesic clayish soil filling crevice in old clinker.		1	1	1		*1256*, *1340*, *1457*, *1414*, *1414*, *597*, *597*, *597*, *597*, *597*, *597*, *865*, *865*, *865*, *865*, *865*	*1360*, *800*, *800*, *800*, *880*, *850*, *900*, *900*, *900*, *900*, *900*, *900*	K-50-5-22, K-51-6-22, K-52-15-22, K-53-12-22, K-53-5-22, K-54-10-22, K-54-14-22, K-54-6-22, K-54-7-22, K-54-8-22, K-54-9-22, K-63-10-22, K-63-11-22, K-63-32-22, K-63-6-22, K-63-7-22	Anthelia juratzkana (Limpr.) Trevis., Barbilophozia hatcheri (A. Evans) Loeske, Calycularia laxa Lindb. & Arnell, Cephalozia bicuspidata (L.) Dumort., Diplophyllum taxifolium (Wahlenb.) Dumort., Lophozia savicziae Schljakov, Lophozia ventricosa (Dicks.) Dumort., Marsupella apiculata Schiffn., Marsupella boeckii (Austin) Lindb. ex Kaal., Marsupella condensata (Ångstr. ex C. Hartm.) Lindb. ex Kaal., Nardia breidleri (Limpr.) Lindb., Nardia insecta Lindb., Pseudolophozia cf. debiliformis (R.M. Schust. & Damsh.) Konstant. & Vilnet, Pseudolophozia debiliformis (R.M. Schust. & Damsh.) Konstant. & Vilnet, Scapania subalpina (Nees ex Lindenb.) Dumort.
Prasanthus suecicus (Gottsche) Lindb.	1457	2	Mountain tundra belt; partly shaded moist clayish soil in the cliff crevice.			1			*1457*, *1457*		K-52-23-22, K-52-25-22	Anthelia juratzkana (Limpr.) Trevis., Cephalozia bicuspidata (L.) Dumort., Gymnomitrion commutatum (Limpr.) Schiffn., Gymnomitrion concinnatum (Lightf.) Corda, Lophozia savicziae Schljakov, Sphenolobus minutus (Schreb.) Berggr.
Preissia quadrata (Scop.) Nees	1261	18	Mountain tundra belt; open moist cliff crevice.		1	1			*1261*	*640*, *1280*	K-65-4-22 (ant., arch., spor.)	
Protochilopsis grandiretis (Lindb. ex Kaal.) A.V. Troitsky, Bakalin & Fedosov						1				*1280*		
Pseudolophozia debiliformis (R.M. Schust. & Damsh.) Konstant. & Vilnet	468, 597, 865, 1265, 1340, 1414	4, 6, 8, 9, 16, 19	Betula ermanii forest belt: open moist boulder along the stream. Alnus fruticosa belt; open moist boulders along stream. Pyroclastic fields; moist to wet fine soil along streams inclusing those in snowbeds, moist clayish soil in the niche in old clinker flow.	1	1	1	1		*1256*, *1256*, *1256*, *1340*, *1340*, *1414*, *597*, *468*, *468*, *865*, *865*	*1360*, *880*, *900*	K-50-2-22, K-50-5-22, K-50-6-22 (gemm. sparse), K-51-5-22, K-51-9-22, K-53-7-22 (gemm.), K-54-14-22 (gemm.), K-61-16-22 (ant., gemm.), K-61-18-22 (gemm.), K-63-26-22 (gemm., ant.), K-63-30-22 (gemm.; hydromorphous phase), K-51-6-22 (similar to <i>Lophoziopsis</i>, but cells are small and gemmae brownish)	Anthelia juratzkana (Limpr.) Trevis., Cephalozia bicuspidata (L.) Dumort., Diplophyllum taxifolium (Wahlenb.) Dumort., Lophozia ventricosa (Dicks.) Dumort., Marsupella tubulosa Steph., Pleurocladula albescens (Hook.) Grolle, Solenostoma sphaerocarpum (Hook.) Steph.
Pseudolophozia sudetica (Nees ex Huebener) Konstant. & Vilnet	606, 638, 865, 1457	2, 19, 22, 23	Alnus fruticosa belt; open moist boulders along streams, including those with Fe-rich waters, fine soil in snowbeds, moist scoria on steep slopes. Mountain tundra belt; open moist cliffs and their crevices, including those filled with fine soil, moist clayish soil on steep slopes.		1	1			*1457*, *1457*, *1457*, *638*, *638*, *606*, *606*, *606*, *865*, *865*	*900*, *900*, *850*, *1000*, *1000*, *800*, *800*, *880*, *900*, *900*, *900*, *900*, *900*, *900*	K-52-10-22, K-52-12-22, K-52-7-22, K-58-11-22, K-58-9-22, K-59-11-22 (gemm., per. juv.; gemmae turn to greenish), K-59-5-22, K-59-9-22 (oil boidies in seta are photographed), K-63-21-22, K-63-8-22	Anthelia juratzkana (Limpr.) Trevis., Cephalozia bicuspidata (L.) Dumort., Cryptocoleopsis imbricata Amak., Diplophyllum taxifolium (Wahlenb.) Dumort., Gymnomitrion commutatum (Limpr.) Schiffn., Gymnomitrion concinnatum (Lightf.) Corda, Nardia geoscyphus (De Not.) Lindb., Nardia japonica Steph., Schistochilopsis incisa (Schrad.) Konstant.

Ptilidium pulcherrimum (Weber) Vain.	447, 468	12, 16	Betula ermanii forest belt; open moist Betula trunk bases, mesic leaned Pinus pumila living branches.	1					*447*, *468*	*400*	K-55-1-22, K-61-1-22	Lophoziopsis longidens (Lindb.) Konstant. et Vilnet
Radula constricta Steph.	420	15	Betula ermanii forest belt; temporarily flooded humus on the slope to large pond.	1					*420*		K-62-12-22 (ant.)	
Radula polyclada A. Evans	1261	18	Mountain tundra belt; open moist cliff crevice.			1			*1261*, *1261*	*1280*, *1280*, *1280*, *1280*	K-65-1-22, K-65-2-22	Aneura pinguis (L.) Dumort., Diplophyllum taxifolium (Wahlenb.) Dumort., Sphenolobus minutus (Schreb.) Berggr.
Riccardia chamedryfolia (With.) Grolle	335, 641	14, 17	Betula ermanii forest belt; moist cliff crevice. Alnus fruticosa belt; peat in open wet hollow in mesotrophic swampy community.	1	1				*335*, *335*, *335*, *641*	*374*, *374*, *600*, *374*	K-43-4-22, K-43-5-22, K-43-6-22, K-66-15b-22	Mesoptychia rutheana (Limpr.) L. Söderstr. & Váňa, Odontoschisma elongatum (Lindb.) A. Evans, Saccobasis polita (Nees) H. Buch
Riccardia multifida (L.) Gray ssp. decrescens (Steph.) Furuki					1					*550*, *550*		
Saccobasis polita (Nees) H. Buch	641	17	Alnus fruticosa belt; peat in open wet hollow in mesotrophic swampy community.		1	1			*641*, *641*, *641*, *641*, *641*, *641*, *641*, *641*, *641*	*850*	K-66-12-22, K-66-15a-22, K-66-15b-22, K-66-15c-22, K-66-15d-22, K-66-15e-22, K-66-15f-22, K-66-2-22, K-66-7-22	Cephaloziella divaricata (Sm.) Schiffn., Harpanthus flotovianus (Nees) Nees, Odontoschisma elongatum (Lindb.) A. Evans, Riccardia chamedryfolia (With.) Grolle, Scapania cf. paludicola Loeske & Müll. Frib., Scapania paludicola Loeske & Müll. Frib.
Sauteria alpina (Nees) Nees	1457	2	Mountain tundra belt; partly shaded moist clayish soil in the cliff crevice.		1	1			*1457*, *1457*	*640*, *640*, *1280*	K-52-21-22 (ant., arch.), K-52-22-22 (ant., arch.)	
Scapania curta (Mart.) Dumort.						1				*850*		
Scapania cuspiduligera (Nees) Müll. Frib.						1				*850*		
Scapania degenii Schiffn. ex Müll. Frib.	2075	1	Alpine heathlands; moist cliff crevice in part shade in crater rim.					1	*2075*		K-49-3-22 (gemm.)	Gymnomitrion concinnatum (Lightf.) Corda
Scapania irrigua (Nees) Nees	468	16	Betula ermanii forest; moist boulder near stream in part shade.	1	1	1	1		*468*	*374*, *374*, *850*, *1360*, *460*, *550*, *880*, *900*	K-61-24-22	
Scapania mucronata H. Buch	471	10	Betula ermanii forest belt; moist tufa cliff near stream in part shade.	1					*471*, *471*		K-56-4-22 (depauperate plants), K-56-5-22 (per.)	
Scapania obcordata (Berggr.) S.W. Arnell	2075	1	Alpine heathlands; moist cliff crevice in part shade in crater rim.			1		1	*2075*	*900*	K-49-2-22 (gemm.)	Cephaloziella divaricata (Sm.) Schiffn.
Scapania paludicola Loeske & Müll. Frib.	641	17	Alnus fruticosa belt; open wet peat in the hollow of mesotrophic swamp.		1	1			*641*, *641*, *641*, *641*, *641*, *641*, *641*, *641*, *641*	*1000*	K-66-10-22, K-66-15e-22, K-66-5-22, K-66-8-22, K-66-8a-22, K-66-9-22, K-66-15c-22, K-66-15d-22, K-66-15f-22	Cephalozia bicuspidata (L.) Dumort., Cephaloziella divaricata (Sm.) Schiffn., Fuscocephaloziopsis pleniceps (Austin) Váňa & L. Söderstr., Odontoschisma elongatum (Lindb.) A. Evans, Saccobasis polita (Nees) H. Buch, Schljakovia kunzeana (Huebener) Konstant. & Vilnet
Scapania paludosa (Müll. Frib.) Müll. Frib.	594, 638	21, 23	Alnus fruticosa belt; open wet strata of degrading peat and wet boulders near the streams.	1	1	1			*638*, *594*, *594*	*550*, *880*, *900*, *900*, *600*, *850*, *850*, *850*, *1000*, *1000*, *1000*, *900*, *900*, *900*, *900*, *900*, *900*	K-58-13-22, K-60-6-22, K-60-7-22	
Scapania parvifolia Warnst.	1080	20	Alnus fruticosa belt; open moist clayish soil on a steep slope.		1				*1080*		K-64-1-22	
Scapania pseudouliginosa Potemkin, Bakalin, Vilnet & Klimova	471, 606, 865	10, 19, 22	Betula ermanii forest belt; open wet boulder near stream with Sulfur-rich water. Alnuis fruticosa belt; open moist boulders and clayish soil along streams, inclusing those with Fe-rich waters.	1	1				*471*, *606*, *865*, *865*		K-56-15-22, K-59-1-22 (mod. <i>leptoderma</i>), K-63-12-22 (mod. <i>leptoderma-viridis</i>), K-63-19-22	Calypogeia kamchatica Bakalin, A.V. Troitsky et Maltseva, Harpanthus flotovianus (Nees) Nees
Scapania subalpina (Nees ex Lindenb.) Dumort.	447, 468, 573, 606, 638, 1265, 1340	6, 8, 12, 16, 22, 23, 24	Betula ermanii forest belt; open wet humus and wet to moist boulders along streams. Alnus fruticosa belt; open wet boulders near streams including those with Fe-rich waters. Pyroclastic fields; open moist boulders and wet clayish soil near streams.	1	1	1	1		*1256*, *1340*, *447*, *638*, *606*, *468*, *468*, *468*, *1340*, *573*	*1360*, *1360*, *800*, *900*, *900*, *600*, *400*	K-50-10-22 (ant.), K-51-6-22, K-55-4-22 (gemm.), K-58-12-22, K-59-2-22, K-61-17-22 (gemm.), K-61-4-22, K-61-7-22, K-51-8-22 (leaf lobes subequal, stem blackish, with 2-3-stratose cortex, in stem features approaching <i>S. undulata</i>), K-57-10-22 (juvenile plants)	Cephalozia bicuspidata (L.) Dumort., Chiloscyphus polyanthos (L.) Corda, Conocephalum salebrosum Szweyk., Buczk. & Odrzyk., Pellia neesiana (Gottsche) Limpr., Pleurocladula albescens (Hook.) Grolle, Pseudolophozia cf. debiliformis (R.M. Schust. & Damsh.) Konstant. & Vilnet
Scapania undulata (L.) Dumort.	471	10	Betula ermanii forest belt; wet tufa cliff near stream in full shade.	1		1			*471*	*850*, *900*	K-56-2-22 (mod. <i>viridis</i>)	
Schistochilopsis incisa (Schrad.) Konstant.	573, 865, 1414, 1457	2, 7, 19, 24	Alnus fruticosa belt; open to partly shaded moist boulders along streams, incuding those Sulfur-rich, and cliff crevices. Mountain tundra belt; open to partly shaded moist cliff crevices, including those fuilled with fine soil.		1	1			*1414*, *1457*, *1457*, *1457*, *573*, *865*, *865*	*850*, *900*, *800*, *900*	K-46-1-22, K-52-12-22, K-52-13-22, K-52-18-22 (per.), K-57-13-22, K-63-18-22 (per.), K-63-32a-22	Cryptocoleopsis imbricata Amak., Diplophyllum albicans (L.) Dumort., Diplophyllum taxifolium (Wahlenb.) Dumort., Gymnomitrion concinnatum (Lightf.) Corda, Lophozia ventricosa (Dicks.) Dumort., Marsupella apiculata Schiffn., Pseudolophozia sudetica (Nees ex Huebener) Konstant. & Vilnet, Solenostoma subellipticum (Lindb. ex Heeg) R.M. Schust., Sphenolobus minutus (Schreb.) Berggr.
Schistochilopsis pacifica Bakalin	606, 641, 865	17, 19, 22	Alnus fruticosa belt; open moist boulders and clayish soil along streams (including those with Fe-rich waters), open wet peat in hollow in mesotrophic swampy community.		1	1			*606*, *865*, *641*	*900*	K-59-14-22, K-63-20-22, K-66-1-22	Anthelia juratzkana (Limpr.) Trevis., Calypogeia kamchatica Bakalin, A.V. Troitsky et Maltseva, Cephalozia bicuspidata (L.) Dumort., Diplophyllum taxifolium (Wahlenb.) Dumort., Harpanthus flotovianus (Nees) Nees, Nardia pacifica Bakalin
Schljakovia kunzeana (Huebener) Konstant. & Vilnet	641	17	Alnus fruticosa belt; open wet peat in hollow in mesotrophic swampy community.		1				*641*, *641*		K-66-5-22, K-66-8a-22 ((sol.))	Scapania paludicola Loeske & Müll. Frib.
Schljakovianthus quadrilobus (Lindb.) Konstant. & Vilnet var. quadrilobus							1			*1360*		
Schljakovianthus quadrilobus (Lindb.) Konstant. & Vilnet var. glareosa (Jørg.) Konstant. & Vilnet							1			*1360*		
Solenostoma confertissimum (Nees) Schljakov	468	16	Betula ermanii forest belt; open to partly shaded moist boulders along streams.	1					*468*, *468*		K-61-21-22 (ant., arch.), K-61-22-22 (per., ant.)	Conocephalum salebrosum Szweyk., Buczk. & Odrzyk.
Solenostoma pusillum (C.E.O. Jensen) Steph.					1					*800*		
Solenostoma rossicum Bakalin et Vilnet							1			*1360*, *1360*, *1360*, *1360*		
Solenostoma sphaerocarpum (Hook.) Steph.	1340, 1457	2, 6	Mountain tundra belt; open moist clayish soil and cliff crevices on steep slopes. Pyroclastic fields; open moist clayish soil near the stream.		1	1	1		*1340*, *1457*, *1457*, *1457*	*1360*, *1360*, *800*, *900*	K-51-5-22 ((sparse)), K-52-11-22 (per., ant., spor.), K-52-2-22, K-52-6-22	Anthelia juratzkana (Limpr.) Trevis., Cephalozia bicuspidata (L.) Dumort., Cryptocolea imbricata R.M. Schust., Gymnomitrion commutatum (Limpr.) Schiffn., Gymnomitrion concinnatum (Lightf.) Corda, Lophozia savicziae Schljakov, Nardia harae Amakawa, Nardia insecta Lindb., Pseudolophozia debiliformis (R.M. Schust. & Damsh.) Konstant. & Vilnet
Solenostoma subellipticum (Lindb. ex Heeg) R.M. Schust.	468, 471, 573, 597, 865, 1265	8, 9, 10, 16, 19, 24	Betula ermanii forest belt; moist humus and moist to wet boulders along streams, including those with Sulfur-rich waters. Alnus fruticosa belt; open moist to wet boulders along streams. Pyroclastic field; open moist boulder near the stream.	1	1	1	1		*1256*, *597*, *597*, *471*, *471*, *471*, *573*, *573*, *573*, *573*, *573*, *468*, *468*, *865*, *865*, *865*, *865*	*640*, *550*, *880*, *550*, *880*, *900*, *900*, *850*, *1000*, *900*, *900*, *900*, *900*, *900*	K-50-12-22 (ant., arch., per.), K-54-3-22 (ant., arch.), K-54-4-22, K-56-11-22, K-56-16-22 (per., ant.; paroicous; a form with deeply purple rhizoids), K-56-7-22, K-57-13-22, K-57-2-22, K-57-4-22 (ant., arch.), K-57-7-22, K-57-9-22, K-61-14-22 (ant., arch.; with some male branches), K-61-23-22 (ant., per.), K-63-13-22 (per., ant.), K-63-22-22 (per., ant.), K-63-23-22 (ant., arch.), K-63-29-22	Anthelia juratzkana (Limpr.) Trevis., Calypogeia kamchatica Bakalin, A.V. Troitsky et Maltseva, Cephalozia bicuspidata (L.) Dumort., Conocephalum salebrosum Szweyk., Buczk. & Odrzyk., Diplophyllum taxifolium (Wahlenb.) Dumort., Hygrobiella squamosa Bakalin & Vilnet, Jungermannia aff. pumila With., Jungermannia eucordifolia Schljakov, Nardia insecta Lindb., Pellia neesiana (Gottsche) Limpr., Schistochilopsis incisa (Schrad.) Konstant.

Sphenolobus minutus (Schreb.) Berggr.	471, 1261, 1457	2, 10, 18	Betula ermanii forest belt; mesic tufa conglomerate near the stream. Mountain tundra belt; moist cliff crevices including those filled with fine soil.	1		1			*1457*, *1457*, *1457*, *1457*, *1457*, *1457*, *471*, *1261*		K-52-13-22, K-52-17-22, K-52-20-22, K-52-24-22, K-52-25-22, K-52-5-22, K-56-9-22 (per., spor.), K-65-1-22	Anthelia juratzkana (Limpr.) Trevis., Cephalozia bicuspidata (L.) Dumort., Cryptocoleopsis imbricata Amak., Diplophyllum taxifolium (Wahlenb.) Dumort., Gymnomitrion concinnatum (Lightf.) Corda, Lophozia savicziae Schljakov, Marsupella apiculata Schiffn., Prasanthus suecicus (Gottsche) Lindb., Radula polyclada A. Evans, Schistochilopsis incisa (Schrad.) Konstant., Trilophozia quinquedentata (Huds.) Bakalin
Tetralophozia setiformis (Ehrh.) Schljakov	1265	8	Pyroclastic field; mesic crevice between boulders in rock field.				1		*1256*		K-50-13-22 (ant., arch., per.)	
Trilophozia quinquedentata (Huds.) Bakalin	1457	2	Mountain tundra belt; partly shaded moist cliff crevice.		1	1			*1457*	*640*, *640*, *1280*, *1280*	K-52-20-22	Diplophyllum taxifolium (Wahlenb.) Dumort., Sphenolobus minutus (Schreb.) Berggr.
