# Supplementary material for: Liverwort altitudinal patterns in East Kamchatka: dividing the indivisible
Source: Front Plant Sci. 2026 Sep 11;17:1904286. doi: 10.3389/fpls.2026.1904286 (PMC13612434; doi:10.3389/fpls.2026.1904286)
Supplement: Supplementary file 4 [file DataSheet4.pdf]

ID	Taxon	n	n_used	µ_MLE	µ_RD	σ_RD	Group	Iso. status
1	Aneura maxima (Schiffn.) Steph.	3	3	335	180	225	A	OK
2	Aneura pinguis (L.) Dumort.	11	8	412	180	245	A	needs_taxonomic_check
3	Anthelia juratzkana (Limpr.) Trevis.	30	30	1259	1672	473	C	OK
4	Barbilophozia hatcheri (A. Evans) Loeske	4	4	918	1102	293	C	OK
5	Barbilophozia lycopodioides (Wallr.) Loeske	3	3	461	180	261	A	OK
6	Blepharostoma brevirete (Bryhn & Kaal.) Vilnet & Bakalin	3	3	1280	1335	211	C	OK
7	Blepharostoma primum Vilnet et Bakalin	1	1	468	420	185	B	single_record
8	Calycularia laxa Lindb. & Arnell	3	3	611	594	202	B	OK
9	Calypogeia integristipula Steph.	1	1	638	626	198	B	single_record
10	Calypogeia kamchatica Bakalin, A.V. Troitsky et Maltseva	19	19	503	180	340	A	OK
11	Calypogeia muelleriana (Schiffn.) Müll. Frib.	2	2	850	876	182	B	OK
12	Cephalozia bicuspidata (L.) Dumort.	43	43	829	829	436	B	OK
13	Cephalozia otaruensis Steph.	1	1	865	896	180	B	single_record
14	Cephaloziella arctogena (R.M. Schust.) Konstant.	1	1	447	411	178	B	single_record
15	Cephaloziella divaricata (Sm.) Schiffn.	12	11	442	180	295	A	questionable
16	Cephaloziella rubella (Nees) Warnst.	1	1	400	387	158	B	single_record
17	Cephaloziella varians (Gottsche) Steph.	1	1	1360	1402	184	C	single_record
18	Chiloscyphus polyanthos (L.) Corda	8	8	501	180	347	A	OK
19	Conocephalum salebrosum Szweyk., Buczk. & Odrzyk.	8	8	553	180	393	A	OK
20	Cryptocolea imbricata R.M. Schust.	1	1	1457	1690	77	C	single_record
21	Cryptocoleopsis imbricata Amak.	16	15	1383	1477	218	C	questionable
22	Diplophyllum albicans (L.) Dumort.	7	6	1446	1831	127	C	questionable
23	Diplophyllum taxifolium (Wahlenb.) Dumort.	37	37	874	901	631	B	OK
24	Douinia plicata (Lindb.) Konstant. et Vilnet	3	3	1384	1473	220	C	OK

25	Endogemma caespiticia (Lindenb.) Konstant., Vilnet & A.V. Troitsky	1	1	1360	1402	184	C	single_record
26	Fossombronia alaskana Steere & Inoue	3	3	420	180	246	A	OK
27	Frullania austinii J.J. Atwood, Vilnet, Mamontov & Konstant.	3	3	240	180	198	A	OK
28	Frullania subarctica Vilnet, Borovich., Bakalin	5	5	1280	1335	211	C	OK
29	Fuscocephaloziopsis pleniceps (Austin) Váňa & L. Söderstr.	4	4	801	708	266	B	OK
30	Gymnocolea inflata (Huds.) Dumort.	8	6	1357	1424	198	C	needs_taxonomic_check
31	Gymnocolea marginata (Steph.) S. Hatt.	1	1	573	542	197	B	single_record
32	Gymnomitrion adustum Nees	1	1	865	896	180	B	single_record
33	Gymnomitrion alpinum (Gottsche ex Husn.) Schiffn.	1	1	1360	1402	184	C	single_record
34	Gymnomitrion commutatum (Limpr.) Schiffn.	6	6	1296	1690	77	C	OK
35	Gymnomitrion concinnatum (Lightf.) Corda	12	12	1521	1890	154	C	OK
36	Gymnomitrion kamchaticum Mamontov, Vilnet & Konstant.	5	5	1166	1993	148	D	OK
37	Harpanthus flotovianus (Nees) Nees	14	14	757	790	355	B	OK
38	Hygrobiella intermedia Bakalin & Vilnet	2	2	900	936	177	B	OK
39	Hygrobiella squamosa Bakalin & Vilnet	4	4	620	511	247	B	OK
40	Isopaches bicrenatus (Schmid. ex Hoffm.) H. Buch	1	1	900	936	177	B	single_record
41	Jungermannia atrovirens Dumort.	1	1	900	936	177	B	single_record
42	Jungermannia borealis Damsh. & Váňa	1	1	471	420	185	B	single_record
43	Jungermannia eucordifolia Schljakov	8	8	848	934	218	B	OK
44	Jungermannia exsertifolia Steph.	5	5	642	180	494	A	OK
45	Jungermannia aff. pumila With.	6	6	674	638	254	B	OK
46	Lophocolea heterophylla (Schrad.) Dumort.	1	1	450	411	178	B	single_record
47	Lophocolea minor Nees	5	5	422	180	247	A	OK
48	Lophozia fuscovirens Bakalin & Vilnet	1	1	1457	1690	77	C	single_record

49	Lophozia guttulata (Lindb. & Arnell) A. Evans	5	5	471	180	279	A	OK
50	Lophozia longiflora (Nees) Schiffn.	5	5	695	180	670	A	OK
51	Lophozia murmanica Kaal.	2	2	914	1000	517	B	two_record_unresolved
52	Lophozia saviczиae Schljakov	19	19	1259	2125	429	D	OK
53	Lophozia ventricosa (Dicks.) Dumort.	10	10	775	837	248	B	OK
54	Lophozia wenzelii (Nees) Steph.	2	2	1130	1223	291	C	OK
55	Lophoziopsis longidens (Lindb.) Konstant. et Vilnet	5	5	446	180	255	A	OK
56	Lophoziopsis polaris (R.M. Schust.) Konstant. & Vilnet	1	1	2075	2125	180	D	single_record
57	Marchantia alpestris (Nees) Burgeff	7	7	740	779	282	B	OK
58	Marchantia latifolia Gray	1	1	450	411	178	B	single_record
59	Marchantia polymorpha L.	1	1	597	571	197	B	single_record
60	Marsupella apertifolia Steph.	2	2	966	1074	459	B	two_record_unresolved
61	Marsupella apiculata Schiffn.	12	12	1459	2125	519	D	OK
62	Marsupella boeckii (Austin) Lindb. ex Kaal.	3	2	597	571	197	B	questionable
63	Marsupella condensata (Ångstr. ex C. Hartm.) Lindb. ex Kaal.	14	14	1380	1463	219	C	OK
64	Marsupella funckii (F.Weber et Mohr) Dumort.	1	1	900	936	177	B	single_record
65	Marsupella tubulosa Steph.	5	4	532	180	390	A	questionable
66	Mesoptychia rutheana (Limpr.) L. Söderstr. & Váňa	9	9	370	180	234	A	OK
67	Metasolenostoma ochotense (Bakalin & Vilnet) Vilnet & Bakalin	1	1	420	394	165	B	single_record
68	Metasolenostoma orientale Bakalin & Vilnet	2	2	471	180	263	A	OK
69	Moerckia flotoviana (Nees) Schiffn.	11	11	418	180	259	A	OK
70	Nardia breidleri (Limpr.) Lindb.	14	14	1150	1335	270	C	OK
71	Nardia geoscyphus (De Not.) Lindb.	6	6	1217	1514	190	C	OK
72	Nardia geoscyphus (De Not.) Lindb. var. bifida R.M. Schust.	2	2	720	733	222	B	OK
73	Nardia harae Amakawa	5	4	606	573	219	B	questionable

74	Nardia insecta Lindb.	6	5	547	417	237	B	questionable
75	Nardia japonica Steph.	23	23	766	813	243	B	OK
76	Nardia pacifica Bakalin	11	11	760	806	254	B	OK
77	Neoorthocaulis attenuatus (Mart.) L. Söderstr., De Roo & Hedd.	1	1	400	387	158	B	single_record
78	Odontoschisma elongatum (Lindb.) A. Evans	14	14	530	337	356	B	OK
79	Odontoschisma macounii (Austin) Underw.	2	2	1280	1310	186	C	OK
80	Pellia neesiana (Gottsche) Limpr.	29	29	631	431	452	B	OK
81	Peltolepis quadrata (Saut.) Müll. Frib.	2	2	724	725	244	B	OK
82	Pleurocladula albescens (Hook.) Grolle	28	28	917	969	365	B	OK
83	Prasanthus suecicus (Gottsche) Lindb.	2	2	1457	1690	77	C	OK
84	Preissia quadrata (Scop.) Nees	3	3	1060	1132	345	C	OK
85	Protochilopsis grandiretis (Lindb. ex Kaal.) A.V. Troitsky, Bakalin & Fedosov	1	1	1280	1310	186	C	single_record
86	Pseudolophozia debiliformis (R.M. Schust. & Damsh.) Konstant. & Vilnet	14	14	1019	1222	373	C	OK
87	Pseudolophozia sudetica (Nees ex Huebener) Konstant. & Vilnet	24	24	905	929	268	B	OK
88	Ptilidium pulcherrimum (Weber) Vain.	3	3	438	180	253	A	OK
89	Radula constricta Steph.	1	1	420	394	165	B	single_record
90	Radula polyclada A. Evans	6	6	1274	1325	225	C	OK
91	Riccardia chamedryfolia (With.) Grolle	8	8	421	180	304	A	OK
92	Riccardia multifida (L.) Gray ssp. decrescens (Steph.) Furuki	2	2	550	397	129	B	OK
93	Saccobasis polita (Nees) H. Buch	10	10	662	653	246	B	OK
94	Sauteria alpina (Nees) Nees	5	5	1095	1502	195	C	OK
95	Scapania curta (Mart.) Dumort.	1	1	850	876	182	B	single_record
96	Scapania cuspiduligera (Nees) Müll. Frib.	1	1	850	876	182	B	single_record
97	Scapania degenii Schiffn. ex Müll. Frib.	1	1	2075	2125	180	D	single_record
98	Scapania irrigua (Nees) Nees	9	9	691	180	509	A	OK
99	Scapania mucronata H. Buch	2	2	471	180	263	A	OK
100	Scapania obcordata (Berggr.) S.W. Arnell	2	2	1488	2125	180	D	two_record_unresolved

101	Scapania paludicola Loeske & Müll. Frib.	10	10	677	661	249	B	OK
102	Scapania paludosa (Müll. Frib.) Müll. Frib.	20	20	830	898	250	B	OK
103	Scapania parvifolia Warnst.	1	1	1080	1117	188	C	single_record
104	Scapania pseudouliginosa Potemkin, Bakalin, Vilnet & Klimova	4	4	702	694	278	B	OK
105	Scapania subalpina (Nees ex Lindenb.) Dumort.	17	17	819	180	1017	A	OK
106	Scapania undulata (L.) Dumort.	3	3	740	731	297	B	OK
107	Schistochilopsis incisa (Schrad.) Konstant.	11	11	1049	1264	349	C	OK
108	Schistochilopsis pacifica Bakalin	4	4	753	799	237	B	OK
109	Schljakovia kunzeana (Huebener) Konstant. & Vilnet	2	2	641	638	205	B	OK
110	Schljakovianthus quadrilobus (Lindb.) Konstant. & Vilnet var. quadrilobus	1	1	1360	1402	184	C	single_record
111	Schljakovianthus quadrilobus (Lindb.) Konstant. & Vilnet var. glareosa (Jørg.) Konstant. & Vilnet	1	1	1360	1402	184	C	single_record
112	Solenostoma confertissimum (Nees) Schljakov	2	2	468	180	263	A	OK
113	Solenostoma pusillum (C.E.O. Jensen) Steph.	1	1	800	825	197	B	single_record
114	Solenostoma rossicum Bakalin et Vilnet	4	4	1360	1431	186	C	OK
115	Solenostoma sphaerocarpum (Hook.) Steph.	8	8	1266	1462	244	C	OK
116	Solenostoma subellipticum (Lindb. ex Heeg) R.M. Schust.	31	31	735	716	370	B	OK
117	Sphenolobus minutus (Schreb.) Berggr.	8	7	1429	1585	151	C	questionable
118	Tetralophozia setiformis (Ehrh.) Schljakov	1	1	1256	1289	187	C	single_record
119	Trilophozia quinquedentata (Huds.) Bakalin	5	5	1059	1365	282	C	OK
