# Supplementary material for: Liverwort altitudinal patterns in East Kamchatka: dividing the indivisible
Source: Front Plant Sci. 2026 Sep 11;17:1904286. doi: 10.3389/fpls.2026.1904286 (PMC13612434; doi:10.3389/fpls.2026.1904286)
Supplement: Supplementary file 7 [file Table1.docx]

Online appendix 1. Additional information on the study area natural conditions.

The park covers the entire catchment area of the Nalycheva River, in some places going beyond it through mountain passes into the basins of adjacent river basins. The climate of the park is mild, mainly maritime humid, with very snowy winters (snow cover up to 2 m high originally, strongly redistribution due to severe winter winds) and cool, short, humid summers. However, the climate acquires continental features with colder winters and warmer summers in western macroslopes of the mountain ridges. The relief on the most extent of the part possesses the alpine appearance (steep slopes, narrow peaks and stream valleys) due to easy erosion of the stratovolcanoes formed mainly in the Quaternary period. A number of them regularly erupt, covering the surface with volcanic ash and larger fragmented pyroclastic, significantly redistributed in accordance with the elements of the mesorelief. The park is almost entirely located in a bowl, bordered on one side by the volcanoes of the Koryaksky-Avachinsky group, and on the other by the Zhupanovsky group. This bowl opens to the southeast along the valley of the Nalycheva River. The relief in the valley of the largest river, the Nalycheva, is quite smoothed with a few hills. Throughout the park, there are various thermal volcanic activity evidences, varying fumaroles to really hot and conditional thermal springs with temperatures of outgoing waters varying from 5 to 70°C. Due to hydrothermal activity, significant travertine cones and sizable shields were formed in a number of areas. In the central part of the park, in the valley of the Levaya Nalycheva River, a large swampy massif is developed on travertine deposits (thus, the seeping waters are neutral or alkaline in the reaction). Several other parts have similar mires developed over travertine shields of smaller size.

The vegetation could be generally characterized as hemiarctic. The dominants of true taiga forests (Picea, Pinus, Abies) here absent. In the wide valleys within the low elevation levels, floodplain forests of Alnus hirsuta (Spach) Rupr. predomination, often with a scattered participation of Padus avium Mill., Populus suaveolens Fisch., Chosenia arbutifolia (Pall.) A. Skvorts. are developed. They are intermingled with forb meadows and Kamchatka tall grass communities with Senecio cannabifolius Less., Filipendula camtschatica (Pall.) Maxim., Cacalia hastata L. and certain ferns as the dominants. The lower levels outside of floodplain are covered by Betula ermanii Cham. stands (actually tall variant of crooked forests) with shrub or forb cover and their intergradations, which higher up turn into thickets of Pinus pumila (Pall.) Regel and Alnus fruticosa Rupr., with A. fruticosa evidently predominating due to the humid climate. Above 900-1000 meters above sea level the mountain tundra of various compositions with dwarf shrub, dwarf shrub-lichen and lichen-moss cover and their transitional variants dominate. Usually, various types of outliers, cliffs and rock outcrops composed by tufa, their conglomerates and old lava clinkers are widespread in the tundra belt. Tundras extend to 1300-1400 m a.s.l., and above they become discontinuous. Above 1700 m a.s.l. they usually transform into alpine heathlands with scattered clumps of dwarf shrubs, grasses or moss-lichen patches. In the crooked forest belt and higher, areas of varying length on gentle slopes are occupied by meadows and meadow-like communities, often turning into mountain tundra communities. In general, the rising up the more low-grass the meadows become. On gentle slopes and flat surfaces of watersheds, scoria fields are common, as well as fields of variously sized fragmented pyroclastic and volcanic ashes. Lava flows of different ages and cliffs on the edges of volcano crater crowns are more stable substrates and are often housed the special species composition. In general, bare spaces, completely free of vegetation, are a common phenomenon in places of late snow melting (snowbed), extensive thermal springs, as well as due to volcanic activity (ashfalls) and extensive landslides of loose substrates, mainly composed of volcanic ash. High landscape diversity, the difference in altitude from sea level to 3456 m above sea level (Koryakskaya Sopka volcano) determines the taxonomic diversity, the patterns of formation of which are complicated by volcanic activity influenced disturbances and the existence of primary successions over vast areas. Three new liverwort species have been described from the park: Nardia pacifica Bakalin, Schistochilopsis pacifica Bakalin, and Calypogeia kamchatica Bakalin, A.V. Troitsky & Maltseva. All of these species have been found in many places outside the park and even outside Kamchatka further.
