# Supplementary material for: Liverwort altitudinal patterns in East Kamchatka: dividing the indivisible
Source: Front Plant Sci. 2026 Sep 11;17:1904286. doi: 10.3389/fpls.2026.1904286 (PMC13612434; doi:10.3389/fpls.2026.1904286)
Supplement: Supplementary file 8 [file Table2.docx]

**Supplementary Table 4. Sensitivity of the μ histogram to bin width and bin origin, and bin-free kernel density estimate**

Part 1 lists the interior local minima of the histogram of μ for all 119 taxa, for six bin widths and three bin origins, and reports the recovery of the adopted cutpoints under two criteria: strict (the cutpoint lies inside a minimum bin) and tolerant (a minimum bin lies within 100 m of the cutpoint). Under the strict criterion the 1100 m cutpoint is recovered in 17 of 18 configurations (in the remaining one it is bracketed by two adjacent minima) and the 1900 m cutpoint in 9 of 18; under the tolerant criterion, in 18 and 15 of 18 respectively. Part 2 gives the modes and minima of a Gaussian kernel density estimate of the same μ values, which does not depend on bin width or bin origin.

**Part 1. Histogram minima**

| **Bin width (m)** | **Bin origin (m)** | **Interior local minima (m)** | **1100 m, strict** | **1100 m, tolerant** | **1900 m, strict** | **1900 m, tolerant** |
| --- | --- | --- | --- | --- | --- | --- |
| 100 | 0 | 200–300 (n = 0); 500–600 (n = 6); 1000–1100 (n = 1); 1500–1600 (n = 3); 1700–1800 (n = 0); 2000–2100 (n = 0) | yes | yes | no | yes |
| 100 | 50 | 250–350 (n = 1); 450–550 (n = 2); 950–1050 (n = 2); 1150–1250 (n = 2); 1550–1650 (n = 1); 1750–1850 (n = 1) | no | yes | no | yes |
| 100 | 100 | 200–300 (n = 0); 500–600 (n = 6); 1000–1100 (n = 1); 1500–1600 (n = 3); 1700–1800 (n = 0); 2000–2100 (n = 0) | yes | yes | no | yes |
| 150 | 0 | 450–600 (n = 6); 1050–1200 (n = 4); 1500–1650 (n = 3); 1950–2100 (n = 1) | yes | yes | no | yes |
| 150 | 50 | 200–350 (n = 1); 500–650 (n = 9); 950–1100 (n = 3); 1700–1850 (n = 1) | yes | yes | no | yes |
| 150 | 100 | 250–400 (n = 6); 1000–1150 (n = 4); 1900–2050 (n = 1) | yes | yes | yes | yes |
| 200 | 0 | 200–400 (n = 6); 1000–1200 (n = 4); 1800–2000 (n = 3) | yes | yes | yes | yes |
| 200 | 50 | 450–650 (n = 9); 1050–1250 (n = 6); 1850–2050 (n = 2) | yes | yes | yes | yes |
| 200 | 100 | 500–700 (n = 12); 1100–1300 (n = 7); 1900–2100 (n = 1) | yes | yes | yes | yes |
| 250 | 0 | 250–500 (n = 13); 1000–1250 (n = 6); 1750–2000 (n = 3) | yes | yes | yes | yes |
| 250 | 50 | 300–550 (n = 15); 1050–1300 (n = 8); 1800–2050 (n = 3) | yes | yes | yes | yes |
| 250 | 100 | 350–600 (n = 18); 1100–1350 (n = 13); 1850–2100 (n = 2) | yes | yes | yes | yes |
| 300 | 0 | 300–600 (n = 19); 900–1200 (n = 13); 1800–2100 (n = 3) | yes | yes | yes | yes |
| 300 | 50 | 350–650 (n = 21); 950–1250 (n = 8); 1550–1850 (n = 7) | yes | yes | no | yes |
| 300 | 100 | 400–700 (n = 19); 1000–1300 (n = 8) | yes | yes | no | no |
| 400 | 0 | 800–1200 (n = 24) | yes | yes | no | no |
| 400 | 50 | 850–1250 (n = 21) | yes | yes | no | no |
| 400 | 100 | 900–1300 (n = 17); 1700–2100 (n = 3) | yes | yes | yes | yes |

**Part 2. Kernel density estimate**

| **KDE bandwidth factor (bw_method)** | **Number of modes** | **Modes (m)** | **Minima (m)** |
| --- | --- | --- | --- |
| 0.1 | 8 | 180, 406, 891, 1105, 1396, 1682, 1862, 2123 | 304, 515, 1068, 1167, 1592, 1814, 1966 |
| 0.15 | 5 | 185, 408, 864, 1388, 2115 | 341, 537, 1129, 1922 |
| 0.2 | 4 | 204, 837, 1385, 2100 | 532, 1135, 1913 |
| 0.25 | 4 | 234, 815, 1383, 2073 | 522, 1142, 1928 |
| 0.3 | 3 | 271, 793, 1372 | 526, 1157 |

Empty intervals of the ordered μ series longer than 100 m: 180–337 m (157 m), 1690–1831 m (141 m), 1890–1993 m (103 m) and 1993–2125 m (132 m). The A/B cutpoint at 300 m and the C/D cutpoint at 1900 m fall inside such intervals.
