# Supplementary material for: Liverwort altitudinal patterns in East Kamchatka: dividing the indivisible
Source: Front Plant Sci. 2026 Sep 11;17:1904286. doi: 10.3389/fpls.2026.1904286 (PMC13612434; doi:10.3389/fpls.2026.1904286)
Supplement: Supplementary file 10 [file Table4.docx]

**Supplementary Table 6. Goodness of fit of the Gaussian response**

Coefficient of determination R² between the normalized empirical range-density function g(x)/max(g) and the fitted Gaussian response, for all 87 taxa with two or more occurrence records, sorted by the number of records. R² is a descriptive measure of curve shape: neighbouring points of g(x) are strongly autocorrelated because consecutive grid positions share most of the records inside the window of radius d = 326.7 m. Median R² = 0.81; 70% of taxa exceed 0.70 and 85% exceed 0.50.

| **ID** | **Taxon** | **n** | **μ (m)** | **Group** | **R²** |
| --- | --- | --- | --- | --- | --- |
| 12 | Cephalozia bicuspidata (L.) Dumort. | 43 | 829 | B | 0.764 |
| 23 | Diplophyllum taxifolium (Wahlenb.) Dumort. | 37 | 901 | B | 0.438 |
| 116 | Solenostoma subellipticum (Lindb. ex Heeg) R.M. Schust. | 31 | 716 | B | 0.898 |
| 3 | Anthelia juratzkana (Limpr.) Trevis. | 30 | 1672 | C | 0.873 |
| 80 | Pellia neesiana (Gottsche) Limpr. | 29 | 431 | B | 0.956 |
| 82 | Pleurocladula albescens (Hook.) Grolle | 28 | 969 | B | 0.822 |
| 87 | Pseudolophozia sudetica (Nees ex Huebener) Konstant. & Vilnet | 24 | 929 | B | 0.76 |
| 75 | Nardia japonica Steph. | 23 | 813 | B | 0.864 |
| 102 | Scapania paludosa (Müll. Frib.) Müll. Frib. | 20 | 898 | B | 0.942 |
| 10 | Calypogeia kamchatica Bakalin, A.V. Troitsky et Maltseva | 19 | 180 | A | 0.922 |
| 52 | Lophozia savicziae Schljakov | 19 | 2125 | D | 0.758 |
| 105 | Scapania subalpina (Nees ex Lindenb.) Dumort. | 17 | 180 | A | 0.614 |
| 21 | Cryptocoleopsis imbricata Amak. | 16 | 1477 | C | 0.783 |
| 37 | Harpanthus flotovianus (Nees) Nees | 14 | 790 | B | 0.778 |
| 63 | Marsupella condensata (Ångstr. ex C. Hartm.) Lindb. ex Kaal. | 14 | 1463 | C | 0.85 |
| 70 | Nardia breidleri (Limpr.) Lindb. | 14 | 1335 | C | 0.748 |
| 78 | Odontoschisma elongatum (Lindb.) A. Evans | 14 | 337 | B | 0.935 |
| 86 | Pseudolophozia debiliformis (R.M. Schust. & Damsh.) Konstant. & Vilnet | 14 | 1222 | C | 0.733 |
| 15 | Cephaloziella divaricata (Sm.) Schiffn. | 12 | 180 | A | 0.914 |
| 35 | Gymnomitrion concinnatum (Lightf.) Corda | 12 | 1890 | C | 0.866 |
| 61 | Marsupella apiculata Schiffn. | 12 | 2125 | D | 0.828 |
| 2 | Aneura pinguis (L.) Dumort. | 11 | 180 | A | 0.883 |
| 69 | Moerckia flotoviana (Nees) Schiffn. | 11 | 180 | A | 0.872 |
| 76 | Nardia pacifica Bakalin | 11 | 806 | B | 0.861 |
| 107 | Schistochilopsis incisa (Schrad.) Konstant. | 11 | 1264 | C | -0.002 |
| 53 | Lophozia ventricosa (Dicks.) Dumort. | 10 | 837 | B | 0.866 |
| 93 | Saccobasis polita (Nees) H. Buch | 10 | 653 | B | 0.702 |
| 101 | Scapania paludicola Loeske & Müll. Frib. | 10 | 661 | B | 0.654 |
| 66 | Mesoptychia rutheana (Limpr.) L. Söderstr. & Váňa | 9 | 180 | A | 0.89 |
| 98 | Scapania irrigua (Nees) Nees | 9 | 180 | A | 0.473 |
| 18 | Chiloscyphus polyanthos (L.) Corda | 8 | 180 | A | 0.93 |
| 19 | Conocephalum salebrosum Szweyk., Buczk. & Odrzyk. | 8 | 180 | A | 0.934 |
| 30 | Gymnocolea inflata (Huds.) Dumort. | 8 | 1424 | C | 0.786 |
| 43 | Jungermannia eucordifolia Schljakov | 8 | 934 | B | 0.896 |
| 91 | Riccardia chamedryfolia (With.) Grolle | 8 | 180 | A | 0.951 |
| 115 | Solenostoma sphaerocarpum (Hook.) Steph. | 8 | 1462 | C | 0.779 |
| 117 | Sphenolobus minutus (Schreb.) Berggr. | 8 | 1585 | C | 0.353 |
| 22 | Diplophyllum albicans (L.) Dumort. | 7 | 1831 | C | 0.656 |
| 57 | Marchantia alpestris (Nees) Burgeff | 7 | 779 | B | 0.838 |
| 34 | Gymnomitrion commutatum (Limpr.) Schiffn. | 6 | 1690 | C | 0.17 |
| 45 | Jungermannia aff. pumila With. | 6 | 638 | B | 0.611 |
| 71 | Nardia geoscyphus (De Not.) Lindb. | 6 | 1514 | C | 0.242 |
| 74 | Nardia insecta Lindb. | 6 | 417 | B | 0.805 |
| 90 | Radula polyclada A. Evans | 6 | 1325 | C | 0.829 |
| 28 | Frullania subarctica Vilnet, Borovich., Bakalin | 5 | 1335 | C | 0.806 |
| 36 | Gymnomitrion kamchaticum Mamontov, Vilnet & Konstant. | 5 | 1993 | D | 0.854 |
| 44 | Jungermannia exsertifolia Steph. | 5 | 180 | A | 0.659 |
| 47 | Lophocolea minor Nees | 5 | 180 | A | 0.875 |
| 49 | Lophozia guttulata (Lindb. & Arnell) A. Evans | 5 | 180 | A | 0.862 |
| 50 | Lophozia longiflora (Nees) Schiffn. | 5 | 180 | A | 0.813 |
| 55 | Lophoziopsis longidens (Lindb.) Konstant. et Vilnet | 5 | 180 | A | 0.862 |
| 65 | Marsupella tubulosa Steph. | 5 | 180 | A | 0.894 |
| 73 | Nardia harae Amakawa | 5 | 573 | B | 0.643 |
| 94 | Sauteria alpina (Nees) Nees | 5 | 1502 | C | 0.195 |
| 119 | Trilophozia quinquedentata (Huds.) Bakalin | 5 | 1365 | C | 0.519 |
| 4 | Barbilophozia hatcheri (A. Evans) Loeske | 4 | 1102 | C | 0.554 |
| 29 | Fuscocephaloziopsis pleniceps (Austin) Váňa & L. Söderstr. | 4 | 708 | B | 0.086 |
| 39 | Hygrobiella squamosa Bakalin & Vilnet | 4 | 511 | B | 0.57 |
| 104 | Scapania pseudouliginosa Potemkin, Bakalin, Vilnet & Klimova | 4 | 694 | B | 0.816 |
| 108 | Schistochilopsis pacifica Bakalin | 4 | 799 | B | 0.817 |
| 114 | Solenostoma rossicum Bakalin et Vilnet | 4 | 1431 | C | 0.724 |
| 1 | Aneura maxima (Schiffn.) Steph. | 3 | 180 | A | 0.898 |
| 5 | Barbilophozia lycopodioides (Wallr.) Loeske | 3 | 180 | A | 0.845 |
| 6 | Blepharostoma brevirete (Bryhn & Kaal.) Vilnet & Bakalin | 3 | 1335 | C | 0.806 |
| 8 | Calycularia laxa Lindb. & Arnell | 3 | 594 | B | 0.714 |
| 24 | Douinia plicata (Lindb.) Konstant. et Vilnet | 3 | 1473 | C | 0.837 |
| 26 | Fossombronia alaskana Steere & Inoue | 3 | 180 | A | 0.871 |
| 27 | Frullania austinii J.J. Atwood, Vilnet, Mamontov & Konstant. | 3 | 180 | A | 0.918 |
| 62 | Marsupella boeckii (Austin) Lindb. ex Kaal. | 3 | 571 | B | 0.58 |
| 84 | Preissia quadrata (Scop.) Nees | 3 | 1132 | C | 0.491 |
| 88 | Ptilidium pulcherrimum (Weber) Vain. | 3 | 180 | A | 0.868 |
| 106 | Scapania undulata (L.) Dumort. | 3 | 731 | B | 0.684 |
| 11 | Calypogeia muelleriana (Schiffn.) Müll. Frib. | 2 | 876 | B | 0.793 |
| 38 | Hygrobiella intermedia Bakalin & Vilnet | 2 | 936 | B | 0.851 |
| 51 | Lophozia murmanica Kaal. | 2 | 1000 | B | -1.515 |
| 54 | Lophozia wenzelii (Nees) Steph. | 2 | 1223 | C | 0.7 |
| 60 | Marsupella apertifolia Steph. | 2 | 1074 | B | -1.139 |
| 68 | Metasolenostoma orientale Bakalin & Vilnet | 2 | 180 | A | 0.837 |
| 72 | Nardia geoscyphus (De Not.) Lindb. var. bifida R.M. Schust. | 2 | 733 | B | 0.768 |
| 79 | Odontoschisma macounii (Austin) Underw. | 2 | 1310 | C | 0.773 |
| 81 | Peltolepis quadrata (Saut.) Müll. Frib. | 2 | 725 | B | 0.808 |
| 83 | Prasanthus suecicus (Gottsche) Lindb. | 2 | 1690 | C | 0.408 |
| 92 | Riccardia multifida (L.) Gray ssp. decrescens (Steph.) Furuki | 2 | 397 | B | 0.32 |
| 99 | Scapania mucronata H. Buch | 2 | 180 | A | 0.837 |
| 100 | Scapania obcordata (Berggr.) S.W. Arnell | 2 | 2125 | D | 0.855 |
| 109 | Schljakovia kunzeana (Huebener) Konstant. & Vilnet | 2 | 638 | B | 0.642 |
| 112 | Solenostoma confertissimum (Nees) Schljakov | 2 | 180 | A | 0.837 |
